# Improved annotation of the insect vector of citrus greening disease: biocuration by a diverse genomics community

**DOI:** 10.1093/database/baz035

**Published:** 2019-03-01

**Authors:** Surya Saha, Prashant S Hosmani, Krystal Villalobos-Ayala, Sherry Miller, Teresa Shippy, Mirella Flores, Andrew Rosendale, Chris Cordola, Tracey Bell, Hannah Mann, Gabe DeAvila, Daniel DeAvila, Zachary Moore, Kyle Buller, Kathryn Ciolkevich, Samantha Nandyal, Robert Mahoney, Joshua Van Voorhis, Megan Dunlevy, David Farrow, David Hunter, Taylar Morgan, Kayla Shore, Victoria Guzman, Allison Izsak, Danielle E Dixon, Andrew Cridge, Liliana Cano, Xiaolong Cao, Haobo Jiang, Nan Leng, Shannon Johnson, Brandi L Cantarel, Stephen Richards, Adam English, Robert G Shatters, Chris Childers, Mei-Ju Chen, Wayne Hunter, Michelle Cilia, Lukas A Mueller, Monica Munoz-Torres, David Nelson, Monica F Poelchau, Joshua B Benoit, Helen Wiersma-Koch, Tom D'Elia, Susan J Brown

**Affiliations:** 1 Boyce Thompson Institute, Ithaca, NY; 2 Indian River State College, Fort Pierce, FL; 3 Division of Biology, Kansas State University, Manhattan, KS; 4 University of Cincinnati, Cincinnati, OH; 5 Cornell University, Ithaca, NY; 6 University of Puget Sound, Tacoma, WA, USA; 7 University of Otago, North Dunedin, Dunedin, New Zealand; 8 Plant Pathology, University of Florida/IFAS Indian River Research and Education Center, Ft. Pierce, FL; 9 Department of Biochemistry and Molecular Biology; 10 Department of Entomology and Plant Pathology, Oklahoma State University, Stillwater, OK; 11 Illumina Inc., San Diego, CA; 12 Los Alamos National Laboratory, Los Alamos, NM; 13 Department of Bioinformatics, UT Southwestern Medical Center, Bioinformatics Core Facility, Dallas, TX; 14 i5K Arthropod Genomics; 15 Human Genome Sequencing Center, Baylor College of Medicine, Houston, TX; 16 USDA ARS, U.S. Horticultural Research Laboratory, Ft. Pierce, FL; 17 USDA Agricultural Research Service, National Agricultural Library, Beltsville, MD, USA; 18 Graduate Institute of Biomedical Electronics and Bioinformatics, National Taiwan University, Taipei, Taiwan; 19 USDA ARS, Emerging Pests and Pathogens Research Unit, Ithaca, NY; 20 Plant Pathology and Plant-Microbe Biology Section; 21 Plant Breeding and Genetics Section, School of Integrative Plant Science, Cornell University, Ithaca, NY; 22 Lawrence Berkeley National Laboratory, Environmental Genomics and Systems Biology, Berkeley, CA; 23 Department of Microbiology, Immunology and Biochemistry, The University of Tennessee Health Science Center, Memphis, TN, USA

Author affiliation for Liliana Cano has been corrected to link to University of Florida/IFAS Indian River Research and Education Center, Ft. Pierce, FL 34945.

