# Supplementary material for: Improved annotation of the insect vector of citrus greening disease: biocuration by a diverse genomics community
Source: Database (Oxford). 2017 Jun 30;2017:bax032. doi: 10.1093/database/bax032 (PMC5502364; doi:10.1093/database/bax032)
Supplement: Supplementary Data [file bax032_supp.zip › Apollo Workshop - Exercises.pdf]

BERKELEY LAB
Life Sciences Division

Monica Munoz-Torres, PhD
Lead Biocurator | Bioinformatics Analyst
Lawrence Berkeley National Laboratory
Joint Genome Institute for the US Department of Energy
2800 Mitchell Dr. Ste 100. Walnut Creek, CA 94598
mcmunozt@lbl.gov | @monimunozto | (925) 296-5624

# Web Apollo Workshop - Exercises
# At Kansas State University
# 17 June, 2015
# M. Munoz-Torres
# Berkeley Bioinformatics Open Source Projects
# Lawrence Berkeley National Laboratory

# Web Apollo Workshop - KSU
# Exercise 1

It has come to our attention that the *Apis mellifera* (honey bee) ortholog of Nesprin-1 (nuclear envelope spectrin repeat proteins) or Muscle specific protein 300 is fragmented into possibly 5 different genes in the current gene set.

In *Drosophila melanogaster* MSP-300 anchors nuclei to actin, and has been reported to be essential for positioning of nurse cell nuclei during oogenesis, and thus production of mature ooctyes. More information available at http://en.wikipedia.org/wiki/Nesprin

You may use this fragment from the *Megachile rotundata* (the alfalfa leafcutter bee) model (NCBI identifier XP_003705060.1; at http://www.ncbi.nlm.nih.gov/) to pull the corresponding location in *Apis mellifera* using BLAT. The protein has over 12K amino acid residues in M. rotundata.

```
>M_rotundata nesprin-1-like (fragment)
MRIVEGRYSGSGGYWNVTILEGGDTGTWGYQKVGILERGGTRRWGYWNVGVPEGGDTGTWGYQKVGILER
GGTRRWGYWNVGVPEGGDTGTWGYQKVGILERGGTRRWGYWNVGVPEGGDTGTWGDSGAWGYWNVRILEG
GDTGTWRYQKVWSGGRTASSEELFQELDGRRNVGHFRSPTNPRPSSRASDSSFEESFERLVEEGELNGAK
VVKFEKITVRKSVREVAGTGVSHQRVLAETSRTPSEEHALEDSAYQSHSHGAPSHGSKSSSVTSFTRFPS
EESLSQRRGSSPQQHLGPDDRTPSEWYAEYHTQSFQNVAARIEYVRSKSEYDAHIAEIKDEQERVQKKTF
VNWINSYLSKRIPPLRVDDLIDDLKDGTRLLALLEVLSGEKLPVERGRNLKRPHFLSNANTALQFLQS
```

Gene models GB40006-RA and GB40007-RA have significant sequence similarity with this gene model.

Using the entire *M. rotundata* model, shows high-scoring segment pairs (hsp) across GB40006-RA, GB40007-RA, GB40008-RA, GB40009-RA, and GB40010-RA.

Clear highlight.

Drag and merge GB40006-RA, GB40007-RA, and GB40008-RA

Rename transcript to: nesprin-1-like-RA

Observing Nurse RNA-seq reads, there are no reads in support of the exon at 483,800 - 483,705, while there are many reads in support of an intron passing across the region. Delete the exon (#1 of GB40007-RA).

Closer inspection of RNA-seq reads also reveals that there are many reads in support of NOT having an exon in coordinates 494,235 - 491,660. Delete exon (#2 of GB40006-RA).

It is now time to inspect the adjacent exons at the merging of GB40007-RA and GB40008-RA. Guided by RNA-seq reads (their length, not their exact coordinates), the phase and the structure of all exons downstream (3') of 454,041 (… 453,654), you may adjust the coordinates for the adjacent exons in this region, so they may have canonical splice sites.

First, the last exon of GB40007-RA can be adjusted to the nearest canonical splice, located 5' in position 454,041.

There is a gap between 453,759 .. 453,710.

The first exon of GB40008-RA should also be adjusted to the nearest canonical site that extends the protein and is supported by RNA-seq reads. After trying a few sites, position 453,659 is a canonical splice site that conserves the phase of the rest of the product and matches the boundaries of RNAseq reads (-2 -- f 1,2,3).

There are many exons in this model; compared to the previous bee model for *A. mellifera* (in NCBI), there is an excess of about 20 exons. This newly created gene product is 7,600 amino acids in length. Use this product to query NCBI's nr for orthologs, using BLAST at http://blast.ncbi.nlm.nih.gov/Blast.cgi

The search retrieves homologs from other Apidae (*A. dorsata, A. florea, N. vitripennis, B. impatiens*) of similar length --> ~39Kbp, 12K aa.

Modify the exon coordinates at the flagged non-canonical sites at the junctions of gene models using RNAseq to guide the changes and use "color by CDS" feature to preserve the phase.

RNA-seq shows that the last exon in GB40008-RA may not be real (or perhaps, it is possible that there are more than one isoform (at 431,750)). There are 11 isoforms in *D. melanogaster*.

With RNA-seq evidence as support, use Apollo’s 'edge-matching' functionality to modify the 5' end of the first exon of GB40009-RA to match the boundary of transcripts from 431,668 to that matching RNAseq evidence at 431,683 (choose exon and RNA-seq read, right-click, 'set as 5' end'). Remember: only those reads oriented in the same sense as the transcription will work with the ‘set as x’ end’ operation.

Name this isoform (RA) and create a copy using the option "Duplicate" from the right-click menu.

In the second isoform (RB), delete exon at ~431,750 (last exon of GB40008-RA) to reveal that the phase in the rest of the peptide is restored to that of original gene model. Copy the 9009 aa residues and query the public databases again using BLAST at NCBI.

Merge the model with GB40010-RA, rename to reflect that this is isoform B.

At the junction, follow RNA-seq reads pattern to adjust the last exon of GB40009-RA to canonical splice site at 427,372, and the first exon of GB40010-RA to canonical splice site at 427,069 immediately 3' of the GAP and the longest extension supported by RNA-seq data.

You may also use additional evidence tracks to support these decisions: e.g. Forager Illumina Bee Contigs, Mixed Antennae 454 Contigs, and Fgenesh++ with RNAseq training data.

In this peptide, at position 394,197 a GC splice donor (common in insects) is visible and heavily supported by the evidence. (GC/AG instead of GT/AG). Note on the comments and finish the annotation.

A final product of 16,509aa in length is obtained.

Check integrity and accuracy:
- blastp vs Insecta (at NCBI) (-*Apis mellifera* ; i.e. without honey bee)
- add GO terms (from Drosophila entries - PAINT)
- search the literature to add PubMed IDs (http://www.ncbi.nlm.nih.gov/pubmed).

```
--------------------------------------------------------------------------

# Web Apollo Workshop - KSU
# Exercise 2
```

It has come to our attention that the honey bee ortholog of small ribonucleoprotein particle protein SmD2 is fragmented into 2 or more genes in the current gene set.

In *Drosophila melanogaster* SmD2 are involved in mRNA splicing, via spliceosome.

You may use the *Apis florea* model (XP_003697276.1) to pull the corresponding location in *Apis mellifera* using BLAT.

```
>A_florea Sm D2-like
MLNNIRNITYFSDQSSLTKPKSEMTPEELAKREEEEFNTGPLSVLTQSVKNNTQVLINCRNNKKLLGRVK
AFDRHCNMVLENVKEMWTELPRTGKGKKKAKPVNKDRFISKMFLRGDSVILVLRNPLATASGK
```

Drag the honey bee gene model you found into the 'User-created Annotation Area' and retrieve its sequence to query the public databases with it to inspect their structure. Query NCBI's nr for orthologs, using BLAST at http://blast.ncbi.nlm.nih.gov/Blast.cgi

Review conserved domains, compare the alignments with other sequences and inspect their length differences.

Find evidence in support of this model looking through the transcriptomes of Forager bees and Nurse bees as well as available ESTs. For now, please refrain from using previous bee gene models.

RNA-seq evidence will support current exons, as well as an additional exon in the 5'end.

Check integrity and accuracy:
- blastp vs Insecta (at NCBI) (-*Apis mellifera*)
- add GO terms (e.g. from Drosophila entries)
- search the literature to add PubMed IDs (http://www.ncbi.nlm.nih.gov/pubmed).

After checking the current protein product vs public databases, alignments will show a discrepancy in the 5'end.
Can you spot the difference?
Are you able to find the missing residues in the current *A. mellifera* genome assembly?
Is there enough evidence to support this hypothesis?
How many isoforms would you annotate in this case?

BERKELEY LAB
Life Sciences Division

# Web Apollo Workshop - KSU
# Exercise 3

It has come to our attention that the honey bee ortholog of FlyBase model CG31619 is fragmented into 2 or more genes in the current gene set.

In *Drosophila melanogaster* CG31619 is involved in proteolysis, and has metalloendopeptidase activity (molecular function).

We know that the homolog of CG31619 in *Apis dorsata* is similar to the ADAMTS proteins. ADAMTS = A Disintegrin And Metalloproteinase with Thrombospondin Motifs, which is a family of peptidases.

You may use the *Apis dorsata* model (XP_003697278.1) to pull the corresponding location in *Apis mellifera* using BLAT in Apollo.

```
>Apis_dorsata ADAMTS-like protein partial
RENPRYWLQPCENPSDFRSEQCAAFDDVPYSGQLLKWYPHYDPSRPCALICRGEQSVENTGNRLRQE
TSVEKTLPHDATDALQLDSEETIVVQLADKVEDGTKCYTDSMDVCINGECMKVGCDLRVGSNKNTDPCGV
CGGNGSSCQSRYSWSLESISACSKSCGGGFKIAMAVCKAIGPDESVVDDSYCDPDNRPEKTLMPCNTHPC
```

Drag each of the honey bee gene models you found into the 'User-created Annotation Area' and retrieve their sequences to query the public databases with them to inspect their structure. Query NCBI's nr database for orthologs, using BLAST at http://blast.ncbi.nlm.nih.gov/Blast.cgi

Review conserved domains, compare the alignments with other sequences and inspect their length differences.

Once you inspect the expected conserved domains, it will be apparent that the models need to be brought together. Find evidence in support of this operation looking through the transcriptomes of Forager bees and Nurse bees as well as available ESTs. For now, please refrain from using previous bee gene models. The available evidence will show support of splice site corrections and possible excess of exons.

Once you are satisfied with a final model, check integrity and accuracy:
- blastp vs Insecta (at NCBI) (-*Apis mellifera*)
- add GO terms (e.g from Drosophila entries)
- search the literature to add PubMed IDs (http://www.ncbi.nlm.nih.gov/pubmed).

Lawrence Berkeley National Laboratory

One Cyclotron Road / MS: 977 / Berkeley, California 94720 / **phone** 510-495-2981 / **fax** 510-495-2723

# Web Apollo Workshop - KSU
# Exercise 4

It has come to our attention that the honey bee ortholog of RNA Polymerase II 140 KD is fragmented into 2 or more genes in the current gene set.

In *Drosophila melanogaster* RNA Pol II is involved in transcription from RNA polymerase II promoter and has DNA binding and DNA-directed RNA polymerase activity (molecular functions).

You may use the *Apis dorsata* model (XP_006610182.1) to pull the corresponding location in *Apis mellifera* using BLAT in Apollo.

```
>Apis_dorsata RNA pol II subunit RPB2-like partial
MYSLEEDQYDDEDAEEISSKLWQEACWIVINAYFDEKGLVRQQLDSFDEFIEMSVQRIVEDSPQIDLQAE
AQHTSGEIENPVRHLLKFEQIYLSKPTHWEKDGAPSPMMPNEARLRNLTYSAPLYVDITKTIVKDGEDPI
ETQHQKTFIGKIPIMLRSKYCLLAGLSDRDLTELNECPLDPG
```

Drag each of the honey bee gene models you found into the 'User-created Annotation Area' and retrieve their sequences to query the public databases with them to inspect their structure. Query NCBI's nr for orthologs, using BLAST at http://blast.ncbi.nlm.nih.gov/Blast.cgi

Check to notice the conserved domains -- are they intact in each model?

Will bringing these models together improve the integrity of the RNA Pol II subunit RPB2 homolog in honey bee?

Re-arrange the boundaries at the joining region to be canonical splice sites on each side.

Did you spot the sequence error visible in the DNA-Track? What effect do you think this had on the automated annotation process?

Once you are satisfied with a final model, check integrity and accuracy:
- blastp vs Insecta (at NCBI) (-Apis mellifera)
- add GO terms (e.g. from Drosophila entries)
- search the literature to add PubMed IDs (http://www.ncbi.nlm.nih.gov/pubmed).

BERKELEY LAB
Life Sciences Division

```
--------------------------------------------------------------------------

# Web Apollo Workshop - KSU
# Exercise 5
```

It has come to our attention that the honey bee ortholog of Ceramidase is fragmented into 2 or more genes in the current gene set.

Ceramidase is an enzyme, which cleaves fatty acids from ceramide, producing sphingosine (SPH) which in turn is phosphorylated by a sphingosine kinase to form sphingosine-1-phosphate (S1P). Ceramide, SPH, and S1P are bioactive lipids that mediate cell proliferation, differentiation, apoptosis, adhesion, and migration.

You may use the *Bombus terrestris* model (XP_003397164.1) to pull the corresponding location in *Apis mellifera* using BLAT. The following is a fragment.

```
>B_terrestris Ceramidase-like
GTTTAAGAGTGTTCGCGCCAATTGTTCGCGGCGAGACTGGCCGTGCAGACCAGCTGTTATAGCCGCGTCT
CCGCTCTGTCTCTGCTGATCCATCGATCACCTACGCATCGATCCCTCGTTCGATCAACGTGGTCATGAGC
TGGAGCGTTTGAGCGCCGCTATCAGACTGGCGGCAGAGAAAAACTGAATGGAGGCACCGGCAGTTGGACG
CTTTAGAATCCTTGCGTTGTTGACGATATGGCTGGTCCAGCTTGCGGTGCCCGGCGCCATCGCGTCTTAC
AGCATCGGGGTGGGCAGAGCAGATGCTACAGGACCCGCCGCTGAAATTGTTTTTATGGGCTACGCGAAGA
TCGATCAAAAAGGATCAGGAATCCATCTTCGAACATTCTCCCGCGCATTCATCATCGACGATGGCGAGGA
GAGGTTCGTCTTCGTCAGCGTGGATAGCGCCATGATAGGAAACGGCGTTCGTCAAACGGTGTTGCAGAAT
CTTGAAAAGGAGTTTGGCAGCCTGTACACAGAGAAAAATGTGATGATCAGTGCAACTCACTCGCACTCCA
CACCCGGTGGATTCATGTTGCACATGTTGTTCGATATTACGACATTCGGTTTCGTTCAAGAGACCTTCGA
TGCTATGGTCAAGGGAATCACGAAGAGTATTCAACGTGCTCACTATGCCATAGTTCCAGGCAGAATATTC
ATCACCCATGGAGAAGTTCATGGTGTGAACATTAATAGAAGCCCATCCG
```

Drag the honey bee gene model(s) you found into the 'User-created Annotation Area' and retrieve its sequence(s) to query the public databases to inspect its/their structure. Query NCBI's nr for orthologs, using BLAST at http://blast.ncbi.nlm.nih.gov/Blast.cgi

Check to notice the conserved domains, if any. And if so, are they intact in each model?

After inspecting the biological evidence in the area, do you think these models should be brought together? If so, how?

Are you able to establish whether you should annotate UTRs to this gene model hypothesis?

Once you are satisfied with a final model, check integrity and accuracy:
- blastp vs Insecta (at NCBI) (-*Apis mellifera*)
- add GO terms (e.g. from Drosophila entries)
- search the literature to add PubMed IDs (http://www.ncbi.nlm.nih.gov/pubmed).

BERKELEY LAB
Life Sciences Division

Web Apollo Workshop - KSU
Exercise 6

It has come to our attention that the honey bee ortholog of adenomatous polyposis coli (APC) -like is fragmented into possibly 4 or more genes in the current gene set.

In *Drosophila melanogaster* adenomatous polyposis coli (APC) -like is involved in beta-catenin binding and microtubule binding.

APC is a negative regulator that controls Beta-catenin concentrations and interacts with E-cadherin, which are involved in cell adhesion. APC is classified as a tumor suppressor gene. Mutations in the APC gene may result in colorectal cancer.

You may use the *Camponotus floridanus* gene model (EFN68235.1) to pull the corresponding location in *Apis mellifera* using BLAT. The following is a fragment.

```
>Camponotus floridanus APC-like partial
GRVLCGSAASVGVEGVGGAWGAQRRQSPPPLASRRLESAADAVVAAAAAAAVATTAASFNPVTQRCAKKG
AVDAVAPRRIYPGPPLVAETSKEDAMEPEAKEEDERRSSTPSPEFYLRRSKRYNEDGSSEEETKQDSISL
PNHRLPSSYRGTWPIRRDVWANQQIGFPAQQSTSLAAHNDVASVMSFSSSSNSAGLDSHSVELQGHRRLG
AKVDVVYNLLGMLEKNGRDDMSTTLLSMSTSLDSCLVMRQSGCLPLLVQLIHAPRQDPDTRDRAMQALHN
VVHAKSDERAGRREARVLRFLEQLRDYCQSLRMSLERGQSMDDLERGHPAATIAALMKLSFDEAHRHAMC
QLGGLHAVAELIEMDHLAHGSESDDQNCITLRRYAGMALTNLTFGDGNNKALLCSFKEFMKALVSQLKSP
SDDLRQVTASVLRNLSWRADSSSKQTLREVGAVTGLMKAAMEGRKESTLKSILSALWNLSAHCSTNKVDI
```

Check to notice the conserved domains, if any. And if so, are they intact in each model?

After inspecting the biological evidence in the area, do you think these models should be brought together? if so, how?

Once you are satisfied with a final model, check integrity and accuracy:
- blastp vs Insecta (at NCBI) (-*Apis mellifera*)
- add GO terms (e.g. from Drosophila entries)
- search the literature to add PubMed IDs (http://www.ncbi.nlm.nih.gov/pubmed).
