# Supplementary material for: Improved annotation of the insect vector of citrus greening disease: biocuration by a diverse genomics community
Source: Database (Oxford). 2017 Jun 30;2017:bax032. doi: 10.1093/database/bax032 (PMC5502364; doi:10.1093/database/bax032)
Supplement: Supplementary Data [file bax032_supp.zip › Supp Table 1.pdf]

Supplementary Table 1

Hemiptera BUSCO markers were selected from 9 species in OTHODBv9.1 and included 3350 genes. The ana

Table 1a: Genome

Data	Sequences	Complete	Fragmented	Missing
**Diaci1.1 genome**	**161,988**	74.8%	0.3%	24.9%
**Pea aphid v2.3**	**31,391**	21.0%	0.0%	79.0%
**Bed bug v1.1**	**41,503**	83.4%	0.3%	16.3%
**White fly v1.1**	**19,761**	77.9%	0.0%	22.1%

Table 1b: Gene sets and transcriptome

Data	Sequences	Complete	Fragmented	Missing
**OGS v1.0**	**21,026**	74.5%	0.3%	25.2%
**MCOT v1.0**	**30,562**	92.9%	0.1%	7.0%
**NCBI v100**	**20,996**	74.7%	0.3%	25.0%
**Maker v1.1**	**18,242**	72.3%	0.4%	27.3%
**Egg**	**76,467**	71.5%	0.2%	28.3%
**Nymph**	**69,175**	71.6%	0.1%	28.3%
**Adult**	**62,412**	60.6%	0.2%	39.2%

alysis shows the number of markers present full-length, in fragments and missing from a given dataset.
