# Supplementary material for: Improved annotation of the insect vector of citrus greening disease: biocuration by a diverse genomics community
Source: Database (Oxford). 2017 Jun 30;2017:bax032. doi: 10.1093/database/bax032 (PMC5502364; doi:10.1093/database/bax032)
Supplement: Supplementary Data [file bax032_supp.zip › Supp Table 2.pdf]

Supplementary Table 2

Overall statistics for each annotation set and related genomes

Annotation set	Gene Models	mRNAs	Total Number of Exons
**NCBI v100**	19,311	20,966	113,609
**MCOT v1.0**	30,562	NA	NA
**Mapped MCOT v1.0**	19,744	19,744	89,423
**Maker v1.1 BCM**	18,205	18,205	94,257
**Curated genes**	530	572	3,482
**OGS1.0**	19,324	21,026	117,064
Drosophila melanogaster	13,929	30,482	77,689
Acyrthosiphon pisum (pea aphid)	20,958	30,790	238,155
Oncopeltus fasciatus	19,589	19,746	104,329
Cimex lectularius (bed bug)	14,085	14,212	89,562

Total Number of Introns	Number of Exons Per mRNA	Average mRNA length	Average Exon Length
92,613	5.42	1,317	243
NA	NA	1,996	NA
69,679	4.53	1,724	381
76,052	5.18	1,018	197
2,910	6.09	1,478	243
95,050	5.57	1,369	246
58,534	2.55	1,374	539
206,612	7.73	2,039	264
84,581	5.28	903	171
75,350	6.3	1,211	192

Total Exon length (bp)
27,605,237
60,995,906
34,029,470
18,536,849
845,582
28,784,551
41,874,371
62,788,971
17,821,062
17,206,103
