# Supplementary material for: Improved annotation of the insect vector of citrus greening disease: biocuration by a diverse genomics community
Source: Database (Oxford). 2017 Jun 30;2017:bax032. doi: 10.1093/database/bax032 (PMC5502364; doi:10.1093/database/bax032)
Supplement: Supplementary Data [file bax032_supp.zip › Supp Table 3.pdf]

Supplementary Table 3

Apollo tracks from genomics, transcriptomics and proteomics data

Category	Apollo tracks	Description
Maker pipeline	Maker	Maker gene predictions
	Augustus	Ab-initio gene predictions
	SNAP	Ab-initio gene predictions
	Repeatmasker	Repeat annotations
	Repeatrunner	Repeat annotations
NCBI v100	Genes	NCBI predicted protein coding genes
	ncRNA	NCBI predicted noncoding RNA
	Pseudogenes	NCBI Predicted pseudogenes
Transcriptomics	Adult	RNAseq from adult psyllid tissue. Mapped reads and coverage plot
	Egg	RNAseq from adult egg tissue. Mapped reads and coverage plot
	Nymph	RNAseq from adult nymph tissue. Mapped reads and coverage plot
	Healthy adult and nymph	Data from Vyas 2015. Mapped reads and coverage plot
	CLas infected adult and nymph	Data from Vyas 2015. Mapped reads and coverage plot
Proteomics	Expressed protein tags (ePSTs)	Peptides from mass spectrometry (MS) proteomics data from whole adult psyllid and percoll gradient fraction (Ramsey 2015)
	Reverse-translated peptides (RTPs)	Peptides from mass spectrometry (MS) proteomics data from whole adult psyllid and percoll gradient fraction (Ramsey 2015)
Genomics	Paired-end DNAseq reads	Mapped reads and coverage plot
