# Supplementary material for: Improved annotation of the insect vector of citrus greening disease: biocuration by a diverse genomics community
Source: Database (Oxford). 2017 Jun 30;2017:bax032. doi: 10.1093/database/bax032 (PMC5502364; doi:10.1093/database/bax032)
Supplement: Supplementary Data [file bax032_supp.zip › Supp Table 4.pdf]

Supplementary Table 4

RNAseq data used for AED analysis

	Source/SRA Sample I	Reference
Adult	ftp://ftp.citrusgreening.	Resse et al., 2013
Egg	ftp://ftp.citrusgreening.	Resse et al., 2013
Nymph	ftp://ftp.citrusgreening.	Resse et al., 2013
Candidatus Liberibacter-free adult	SAMN02716929	Vyas et al., 2015
Candidatus Liberibacter-free nymph	SAMN02716930	Vyas et al., 2015
Candidatus Liberibacter-infected adult	SAMN02716931	Vyas et al., 2015
Candidatus Liberibacter-infected nymph	SAMN02716932	Vyas et al., 2015
female terminal abdomen	SAMN04159742	Wu et al., 2016
male terminal abdomen	SAMN04159740	Wu et al., 2016
female antennal	SAMN04159737	Wu et al., 2016
male antennal	SAMN04159735	Wu et al., 2016
Candidatus Liberibacter-free Gut	NA	From personal communication
Candidatus Liberibacter-infected Gut	NA	From personal communication
