# Supplementary material for: Improved annotation of the insect vector of citrus greening disease: biocuration by a diverse genomics community
Source: Database (Oxford). 2017 Jun 30;2017:bax032. doi: 10.1093/database/bax032 (PMC5502364; doi:10.1093/database/bax032)
Supplement: Supplementary Data [file bax032_supp.zip › Supp Table 5.pdf]

Supplementary Table 5a

Quality control remark key

QC remark	Criterion
A	1. Evidence for 100%+ of exons (RNAseq or proteomics is best, blast hits may be allowed)
	2. Final protein length +- 10% of similar gene in related species
	3. Similar exon count/order to gene in related species
B	1. Evidence for 90%+ of exons (RNAseq or proteomics is best, blast hits may be allowed)
	2. Final protein length +- 25% of similar gene in related species
C	1. Evidence for less than 90% of exons (RNAseq or proteomics is best, blast hits may be allowed)
	2. Final protein length +- 50% of similar gene in related species
D	Not accepted in current form

Supplementary Table 5b

Gene naming conventions

1	Use only gene name. Do not add Dcitr to the gene name! It will be done automatically
	Criterion
a	Evidence for 100%+ of exons (RNAseq or proteomics is best, blast hits may be allowed)
b	Final protein length +- 10% of similar gene in related species
c	Similar exon count/order to gene in related species
2	Add "partial" after the gene name in all other cases, especially for misassembly
	Criterion
a	Evidence for 90%+ of exons (RNAseq or proteomics is best, blast hits may be allowed)
b	Final protein length +- 25% of similar gene in related species
