# Supplementary material for: Improved annotation of the insect vector of citrus greening disease: biocuration by a diverse genomics community
Source: Database (Oxford). 2017 Jun 30;2017:bax032. doi: 10.1093/database/bax032 (PMC5502364; doi:10.1093/database/bax032)
Supplement: Supplementary Data [file bax032_supp.zip › Supp Table 6.pdf]

**Supplementary Table 6**

Counts of responses to pre- and post- questions for University of Cincinnati students about annotation and

| No. | Question |
|---|---|
| 1 | What are some major difficulties associated with genome sequencing and assembly? |
| | **Correct answers included three or more of the following points:** |
| | A. Obtaining research funds for sequencing |
| | B. Recovery of enough DNA for inbred lines or large individuals necessarty for sequencing |
| | C. Proper sequencing depth for assembly |
| | D. Computing time and power for assembly |
| | E. Adequate assessment of genome assembly quality |
| | F. Sex-specific bias |
| | G. Assignment of genome scaffolds to chromosomes |
| | H. Accurate gene prediction. |
| | I. Removal of chimeras and/or microbial contamination |
| | difficulties associated with genome assembly |
| | |
| 2 | What information is utilized to predict genes in a newly assembled genome? |
| | **Correct answers included three or more of the following points:** |
| | A. Previously sequenced gene in NCBI |
| | B. RNA seq data |
| | C. *de novo* assemblies, such as those from Trinity or Oases |
| | D. Proteins/gene sequences in other organism |
| | E. Known characteristics of transcriptional start and stop sites. |
| | F. Known characteristics of splice sites |
| | G. ORF prediction software |
| | *Other answers are acceptable if these indicate the student understands the process of gene prediction |
| | |
| 3 | How does a sequenced genome increase tools available for research on an organism? |
| | **Correct answers included two or more of the following points:** |
| | A. Studies on transcriptional regulation through transcription factors is possible. |
| | B. DNA methylation studies can be conducted. |
| | C. Splice variants in genes can be examined. |
| | D. Development of transgenic lines can more accurate by reducing off-target effects. |
| | E. RNA interference can be developed to target specific splice variants |
| | F. Studies assessing the presence or absence of genes can be more accurate |
| | G. Potential interplay between hosts and symbionts can be examined by looking at the gene repertoire. |
| | *Other answers are acceptable if these indicate the student can justify why the genome will improve research. |

bioinformatics. Note: Not all students completed the pre- and post-test resulting on only 22 students that c

| Pre-test | Post-test |
|---|---|
| 11/22 | 22/22 |
| | |
| | |
| | |
| | |
| | |
| | |
| | |
| | |
| | |
| | |
| | |
| | |
| 6/22 | 17/22 |
| | |
| | |
| | |
| | |
| | |
| | |
| | |
| | |
| | |
| | |
| 8/22 | 20/22 |
| | |
| | |
| | |
| | |
| | |
| | |
| | |
| | |
| | |

could be assessed.
