# Supplementary material for: Improved annotation of the insect vector of citrus greening disease: biocuration by a diverse genomics community
Source: Database (Oxford). 2017 Jun 30;2017:bax032. doi: 10.1093/database/bax032 (PMC5502364; doi:10.1093/database/bax032)
Supplement: Supplementary Data [file bax032_supp.zip › Supplementary_Notes - v1.2.docx]

Gene Reports

[Supplementary Note 1: C-type Lectins 3](#_Toc476312413)

[Supplementary Note 2: Galactoside-Binding Lectins 9](#_Toc476312414)

[Supplementary Note 3: Fibrinogen related proteins 14](#_Toc476312415)

[Supplementary Note 4: PGRP and 𝛽GRP 19](#_Toc476312416)

[Supplementary Note 5: Thioester containing Proteins 23](#_Toc476312417)

[Supplementary Note 6: Toll Receptors 27](#_Toc476312418)

[Supplementary Note 7: Spaetzle proteins 31](#_Toc476312419)

[Supplementary Note 8: Tube proteins 36](#_Toc476312420)

[Supplementary Note 9: Pelle proteins 40](#_Toc476312421)

[Supplementary Note 10: MyD88 proteins 43](#_Toc476312422)

[Supplementary Note 11: Traf6 proteins 47](#_Toc476312423)

[Supplementary Note 12: Cactus proteins 51](#_Toc476312424)

[Supplementary Note 13: Dorsal proteins 54](#_Toc476312425)

[Supplementary Note 14: Imd Pathway CASPAR (FAF1), IKKB, TAK1, TAB proteins 60](#_Toc476312426)

[Supplementary Note 15: JAK/STAT pathway 64](#_Toc476312427)

[Supplementary Note 16: Lysozymes 67](#_Toc476312428)

[Supplementary Note 17: Superoxide dismutases 70](#_Toc476312429)

[Supplementary Note 18: CLIP 74](#_Toc476312430)

[Supplementary Note 19: Autophagy 82](#_Toc476312431)

[Supplementary Note 20: Dicer 84](#_Toc476312432)

[Supplementary Note 21: Drosha 88](#_Toc476312433)

[Supplementary Note 22: Pasha protein 93](#_Toc476312434)

[Supplementary Note 23: Loquacious and R2D2 proteins 97](#_Toc476312435)

[Supplementary Note 24: Argonaute and PIWI proteins 102](#_Toc476312436)

[Supplementary Note 25: Tudor Staphylococcal Nuclease protein 109](#_Toc476312437)

[Supplementary Note 26: Vasa intronic gene 112](#_Toc476312438)

[Supplementary Note 27: Armitage 114](#_Toc476312439)

[Supplementary Note 28: Fragile X Mental Retardation Protein 117](#_Toc476312440)

[Supplementary Note 29: Spindle_E (homeless) 120](#_Toc476312441)

[Supplementary Note 30: Rm62 protein 123](#_Toc476312442)

[Supplementary Note 31: Ras-related nuclear protein 126](#_Toc476312443)

[Supplementary Note 32: Glycolysis pathway 129](#_Toc476312444)

[Supplementary Note 33: Aquaporin 132](#_Toc476312445)

[Supplementary Note 34: Cathepsins and Cysteine Proteases 135](#_Toc476312446)

[Supplementary Note 35: Early developmental genes (Gap and Pair Rule genes) 141](#_Toc476312447)

[Supplementary Note 36: Cuticle proteins 149](#_Toc476312448)

[Supplementary Note 37: Secretome 153](#_Toc476312449)

[Supplementary Note 38: Heat shock proteins 171](#_Toc476312450)

[Supplementary Note 39: Rab proteins 178](#_Toc476312451)

# Supplementary Note 1: C-type Lectins

**Introduction**

C-type Lectins (CTLs) are carbohydrate binding receptors which connect to mostly a wide range of carbohydrate structures (Cambi and Figdor 2003) and proteins as well (Dodd and Drickamer 2001). This gives them the ability to recognize pathogen associated molecular patterns (PAMPs) detecting different pathogens and possibly cancerous cells (Cambi Figdor 2003). They can be involved in transmission of signals after binding to a ligand. Some of them are also known as cell adhesion molecules (CAMs), which enable the transport of immune cells. These are involved in cells traveling to the site of infection through tissue proliferation during the immune response. The C-type Lectins can be either bound to the membrane by a transmembrane region or free-roaming (Weis et al. 1998). The silencing of these genes could lead to the death of an organism, since it will have difficulty preparing an immune response since a C-type Lectin might not be able to detect an organism through carbohydrates usually found on their cell surface, and the absence of CAMs will prevent immune cells from breaking down pathogens as they infect the organism.

**Materials and Methods**

The C-type Lectin sequences for the following insects were collected and used to search the genome of *Diaphorina citri*: *A. pisum, D. melanogastor, O. fasciatus, A. gambiae, A. aegypti,* *C. quinquefasciaitus* and *C. lectularius*. These sequences were used in the BLAT search tool in Web Apollo and BLAST from I5K to find gene model predictions. A total of 10 models were found and annotated, correcting anything that was not supported by evidence tracks like RNA sequence data, genomics, and proteomics tracks. Pairwise alignments using BLASTp were performed using the sequences of the completed models to find orthologues in other insects for additional support. Table 1 contains a list of the top results found in closely related insects as well as their ID, Query coverage, and bit score. The listed insects include: *Acyrthosiphon pisum, Cimex lectularius, Anopheles gambiae, Aedes aegypti,* and *Culex quinquefasciatus*.

The sequences from the annotated models were collected in FASTA format. Sequences of the same genes from other insects were found in NCBI and collected as FASTA files. Using a total of 38 sequences a multiple sequence alignment was made with default MUSCLE settings within MEGA 7. The phylogenetic tree was made with sequences from the following organisms: *Apis mellifera*, *Apis Dorsata*, *Bactrocera cucurbitae*, *Ceratitis capitate*, *Culex quinquefasciatus*, *Cimex lectularius*, *Acyrthosiphon pisum*, *Bombus impatiens*, *Nicrophorus vespilloides*, *Stomoxys calcitrans*, *Athalia rosae*, *Plutella xylostella*, *Homo sapiens*, *Pediculus humanus corporis*, and *Bombyx mori*. The names of most of the genes were simplified to abbreviations, and the species names to the first letter of the genus name and the whole species name. A neighbor joining tree was then made, based on the previous alignment, with 1000 replicates for bootstrap, Poisson model, and pairwise deletion also within MEGA 7.

**Results and Discussion**

Ten C-type lectin gene models were located and annotated after using orthologs from closely related insects including *A. pisum, Drosophila melanogastor, Anopheles gambiae, Aedes aegypti,* *Culex quinquefasciaitus* and *Cimex lectulius*. Most of the models had a C-type Lectin domain that BLAST was able to detect. A multiple alignment was constructed which illustrated sections of conserved sequence. The phylogenetic tree shows that I have indeed found and annotated CTLs, like oxidized low- density lipoprotein receptor, due to their location on the tree in relation to orthologs (Figure 1). Even though the CTLs are conserved from metazoans to humans, there are little to no similarities between CTLs of vertebrates and invertebrates (Dodd and Drickamer 2001). SVEP had not paired with the orthologs found in *H.* sapiens. The lack of accurate pairing possibly indicates that there is a need for more sequences of more closely related organisms to illustrate relations, which was not available. 10 CTL sequences were found and annotated in the *Diaphorina citri* genome while 34 have been found in *Drosophila melanogaster,* 25 in *Anopheles gambiae,* 10 in *Apis mellifera,* and 16 in *Tribolium castaneum* (Tanaka et al. 2008)*.* A comparison to the numbers of CTLs found in those insects show that possibly with better sequence and expression data there might be more CTLs found in the citrus psyllid’s genome. Since these sequences have been supported as probable CTLs as more sequence data is available they could be knocked out with RNAi to determine if it could reduce the citrus psyllid population and prevent the spread of citrus greening.

Table 1: A comparison table between *D.citri* and *A. pisum, D. melanogastor, A. gambiae, A. aegypti,* *C. quinquefasciaitus* and *C. lectulius* derived from the BLAST results. Lists amino acid length of *D.citri* genes, their domains, query coverage, identity, and bit score.

***Diaphorina citri* gene comparison**

Figure1: Phylogenetic tree consisting of *Diaphorina citri* C-type Lectins and the same genes from other insects. The C-type Lectins found in *D.citri* are the following: oxidized low density lipoprotein receptor (OLR), C-type Lectin 3 (CTL3), C-type Lectin 5 (CTL5), E selectin (ESEL), Perlucin, Agglucetin subunit alpha, and selectin like osteoblast derived protein (SVEP). The species names of the additional organisms are shown next to the gene name. An ortholog from another insect could not be found for Agglucetin. Only a human ortholog could be found for SVEP. High bootstrap values show that some genes like OLR are closely related to those in other insects. OLR is also known as CTL8A.

**References**

1. Cambi A and Figdor CG. 2003. Dual function of c-type lectin-like receptors in the immune system. Curr Opin Cell Biol. 15:539-546. PMID: 14519388
2. Dodd RB and Drickamer K. 2001. Lectin-like proteins in model organisms: implications for evolutions of carbohydrate- binding activity. Glycobiology 11(5):71R-79R. PMID: 11425795
3. Tanaka H, Ishibashi J, Fujita K, Nakajima Y, Sagisaka A, Tomimoto, K, Suzuki, N, Yoshiyama, M, Kaneko, Y, Iwasaki, T, Sunagawa, T, Yamaji, K, Asaoka, A, Mita, K, Yamakawa, M. 2008. A genome-wide analysis of genes and gene families involved in innate immunity of Bombyx mori. Insect Biochem Mol Biol 38(12): 1087-1110. PMID: 18835443
4. Weis WI, Taylor ME, and Drickamer K. 1998. The c-type lectin superfamily in the immune system. Immunological Reviews 163:19-34. PMID: 9700499

# Supplementary Note 2: Galactoside-Binding Lectins

**Introduction**

Galactoside-binding lectins (galectins) bind β-galactose with their structurally similar carbohydrate-recognition domains (CRDs) (Cummings and Liu 2009). CRDs can function alone or in clusters creating a β-sandwich structure without Ca^2+^ binding sites (Leffler et al., 2004; Mitchell et al., 2001; Wang et al., 2012).

In the invertebrate *Clavelina picta*, it has been shown that galectin may be an effector molecule in the innate immune system (Vasta et al., 1999), whereas it can be similar in insects. *Anopheles* mosquito galectins were upregulated in the gut and salivary glands after infection of both malaria and bacteria (Dimopoulos et al., 1996, 1998, 1988). It was suggested that galectins may function as a pattern recognition receptor (PRR) by binding saccharides (β-galactose) on the pathogen surface to trigger the innate immune response or agglutinate bacteria in the midgut following blood-feeding (Dimopoulos et al., 1998).

**Methods**

Insecta galectins orthologs were collected from Immunodb and were used to blat against the *Diaphorina citri*, Asian citrus psyllid (ACP), genome. Identification and annotation of galectins was performed in WebApollo where RNA sequence data (adult, nymph, and egg) supported the manually curated gene models. BLASTp was used to confirm accuracy of the annotated galectins and determine the number of CRDs each galectin contains.

Galectin orthologs were collected from a limited NCBI search to include different insect orders. The conserved domains database in NCBI was used to identify and retrieve the CRD amino acid sequences of each collected galectin orthologs. ACP CRDs were globally pairwise aligned with *Acyrthosiphon pisum, Cimex lectularius, Diuraphis noxia,* and *Drosophila melanogaster* using NCBI Needleman-Wunsch tool. Multiple sequence alignments of ACP and orthologous insect galectins CRDs was performed in MEGA6 using ClustalW (Tamura et al 2003). The phylogenetic analysis of the CRDs included, constructing a midpoint rooted neighbor-joining tree in MEGA6 using p-distance to show evolutionary distance and 1,000 bootstrap replicates.

**Results and Discussion**

A total of three galectins and one partial galectin were identified and manually curated within the ACP genome (Table 1). *Acyrthosiphon pisum,* ACP closest relative, only has two galectin paralogs (Gerardo et al., 2010) (Table 3), which are isoforms (NCBI accession: XP_008186241 and XP_001943769). The naming of the ACP galectins are based on NCBI Gnomon. Galectin-5 and 32 kDa beta-galactoside-binding lectin were identified in the same scaffold (WebApollo accession: gi|645507215|ref|NW_007377695.1|). Blastp showed that galectin-4, 32 kDa beta-galactoside-binding lectin, galectin-5, and galectin-4 partial has two CRDs, two CRDs, one CRD, and one CRD, respectively. ACP galectins containing two CRDs, like that of mammalian galectins, may mediate cell-cell communication or trigger signal cascades, as also suggested in *Drosophila melanogaster* (Pace et al., 2002; Liu, 2000; Hadari et al., 2000; Pace et al., 2000; Baum et al., 1995).

In the pairwise alignment analysis (Table 2), the ACP CRDs have an identity of 17% or greater compared to other related organisms. *D. citri* gal-4 CRD1, 32 kDa gal CRD2, and gal-5 CRD1 were analyzed with related insects CRDs from the orders Hymenoptera, and Coleoptera (Figure 1). The tree shows that the ACP CDRs have evolved from each other but still grouped with other hemipterans, further supporting the manually curated models. Coleoptera and Hymenoptera CRDs have also grouped with themselves independently.

Table 1: All the galectins found in the *D. citri* genome. Table shows all galectins with names based on NCBI Gnomon with BLASTp matches, and corresponding protein length, number of CRDs, and domains.

| ***D. citri* galectins** | **BLASTP match**  **(organism, bit score)** | **Protein Length** | **Number of CRDs** | **Domains** |
| --- | --- | --- | --- | --- |
| **Galectin 4** | Galectin 4 *(Zootermopsis nevadensis*, 101) | 286 | 2 | Dimerization interface, sugar binding pocket, dimerization swap strand, Putative alternate dimerization interface, Gal-bind_lectin (CRD), Gal-bind_lectin (CRD), two Gal-bind_lectin, two GLECT, two GLECT superfamily |
| **Galectin 4, partial** | Galectin 9  (*Habropoda laboriosa*, 52.4) | 142 | 1 | Putative alternate dimerization interface, sugar binding pocket, Gal-bind_lectin (CRD), GLECT, Gal-bind­_lectin, GLECT superfamily |
| **Galectin 5** | Galectin 8 *(Zootermopsis nevadensis*, 116) | 218 | 1 | Dimerization swap strand, sugar binding pocket, dimerization interface, putative alternate dimerization interface, Gal-bind_lectin (CRD), Gal-bind_lectin, GLECT, GLECT superfamily |
| **32 kDa beta-galactoside-binding lectin** | Galectin 8 *(Zootermopsis nevadensis*, 184) | 494 | 2 | Two sugar binding pocket, dimerization interface, dimerization swap strand, two putative alternate dimerization interface, two Gal-bind_lectin (CRD), two Gal-bind_lectin, two GLECT, two GLECT superfamily |

Table 2: Global pairwise alignment of *D. citri* CRDs with related organisms displaying percent identity and (Needleman-Wunsch score).

|  | Galectin-4 | | 32 kDa galectin | | Galectin-5 |
| --- | --- | --- | --- | --- | --- |
| *Diaphorina citri* | CRD1 | CRD2 | CRD1 | CRD2 | CRD1 |
| *Acyrthosiphon pisum*  (XP_008186241.1) | 33%  (146) | 22%  (-27) | 28%  (104) | 20%  (7) | 28%  (104) |
| *Cimex lectularius*  (XP_014259365.1) | 17%  (12) | 19%  (-32) | 31%  (147) | 18%  (-29) | 31%  (147) |
| *Diuraphis noxia*  (XP_015371421.1) | 32%  (139) | 22%  (-28) | 27%  (91) | 19%  (2) | 27%  (91) |
| *Drosophila melanogaster*  (NP_001245813.1) | 23%  (52) | 23%  (-24) | 31%  (155) | 20%  (25) | 31%  (155) |

Table 3: Number of galectins in *D. citri* and related organisms.

| Organism | # of galectins |
| --- | --- |
| *Diaphorina citri* | 3 |
| *Acyrthosiphon pisum* | 1 |
| *Cimex lectularius* | 1 |
| *D. melanogaster* | 1 |
| *Aedes aegypti* | 12 |
| *Anopheles gambiae* | 15 |
| *Culex quinquefasciatus* | 5 |

*Numbers based on Ensembl and NCBI, does not include isoforms/partial.


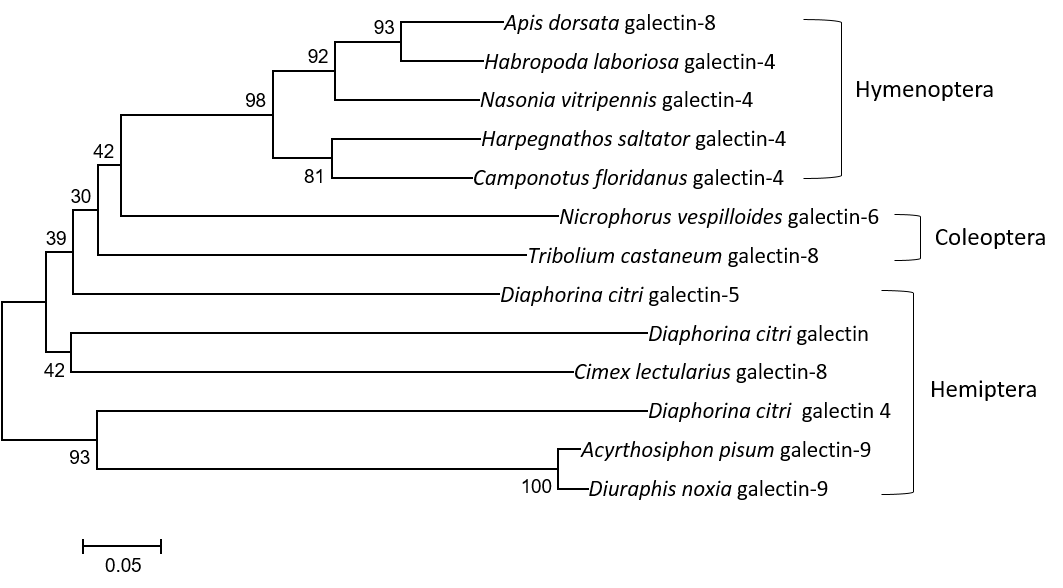


Figure 1: Neighbor-joining tree of 13 galectin CRDs was constructed in MEGA6 using 1000 bootstrap replications. The p-distance method was used to show evolutionary distance. A total of 60 amino acid positions were analyzed for the tree. NCBI accessions: *A. dorsata* [XP_006607868.1], *H. laboriosa* [XP_017794332.1], *N. vitripennis* [XP_008211704.1], *H. saltator* [EFN82261.1], *C. floridanus* [EFN73449.1], *N. vespilloides* [XP_017773770.1], *T. castaneum* [XP_015834566.1], *C. lectularius* [XP_014259365.1], *A. pisum* [XP_008186241.1], *D. noxia* [XP_015371421.1].

**References**

1. Cummings RD, Liu FT. 2009. Galectins. Essentials of Glycobiology 2^nd^ edition.
2. Vasta GR, Quesenberry M, Ahmed H, O’Leary N. 1999. C-type lectins and galectins mediate innate and adaptive immune functions: their roles in the complement activation pathway. Developmental & Comparative Immunology [Internet]. 23(4-5):401-420. PMID:[10426431](http://www.ncbi.nlm.nih.gov/pubmed/10426431)
3. Dimopoulos G, Richman A, della Torre A, Kafatos FC, Louis C, Proc Natl Acad Sci USA 93, 13066–71 (1996).
4. Dimopoulos G, Seeley D, Wolf A, Kafatos FC, Embo J 17, 6115– 23. (1998).
5. Dimopoulos G, Muller HM, Levashina EA, Kafatos FC,Curr Opin Immunol 13, 79–88 (1998).
6. Gerardo NM, et al. 2010 Feb. Immunity and other defenses in pea aphids, *Acyrthosiphon pisum.* Genome Biology [Internet]. [cited 2016 May 17];1-17. PMID: 20178569
7. Leffler, H., Carlsson, S., Hedlund, M., Qian, Y., Poirier, F., 2004. Introduction to galectins. Glycoconj. J. 19, 433e440.
8. Mitchell, D.A., Fadden, A.J., Drickamer, K., 2001. A novel mechanism of carbohydrate recognition by the C-type lectins DC-SIGN and DC-SIGNR. Subunit organization and binding to multivalent ligands. J. Biol. Chem. 276, 28939e28945
9. Wang, L., Wang, L., Yang, J., Zhang, H., Huang, M., Kong, P., Zhou, Z., Song, L., 2012. A multi-CRD C-type lectin with broad recognition spectrum and cellular adhesion from Argopectenirradians. Dev. Comp. Immunol. 36, 591e601.
10. Tamura K., Stecher G., Peterson D., Filipski A., and Kumar S. (2013). MEGA6: Molecular Evolutionary Genetics Analysis version 6.0. *Molecular Biology and Evolution*30: 2725-2729.
11. Pace KE, Lebestky T, Hummel T, Arnoux P, Kwan K, Baum LG. 2002. Characterization of a Novel Drosophila melanogaster Galectin. THE JOURNAL OF BIOLOGICAL CHEMISTRY. 227(13):13091-13098
12. Liu, F.-T. (2000) Clin. Immunol. 97, 79–88
13. Hadari, Y. R., Arbel-Goren, R., Levy, Y., Amsterdam, A., Alon, R., Zakut, R., and Zick, Y. (2000) J. Cell Sci. 113, 2385–2397
14. Pace, K. E., Hahn, H. P., Pang, M., Nguyen, J. T., and Baum, L. G. (2000)J. Immunol. 165, 2331–2334
15. Baum, L. G., Pang, M., Perillo, N. L., Wu, T., Delegeane, A., Uittenbogaart,C. H., Fukuda, M., and Seilhammer, J. (1995) J. Exp. Med. 181, 877–887

# Supplementary Note 3: Fibrinogen related proteins

**Introduction**

Fibrinogen related proteins (FREPS) include a highly conserved fibrinogen like domain (FBG domain) found universally in vertebrates and invertebrates (Wang et al, 2005). In invertebrates the FBG domains are predicted to function in innate immunity by recognizing carbohydrates and their derivatives on the surface of microorganisms. The FBG domain includes approximately 200 amino acids with high sequence similarity to the C terminus of fibrinogen, however some FREPs include a truncated domain (Middah and Wang, 2008). A number of N-terminal domains can be joined to the highly conserved FBG domain allowing for variability and antibodies against mammalian fibrinogen can be used to find proteins with FBG domains in invertebrates (Hanington and Zhang, 2010). FREP genes include angiopoietin, tenascin, scabrous, aslectin, microfibril associated proteins (MAP), ficolin, and tachylectin; all of which were searched for in the *D. citri* genome.

**Methods**

Fibrinogen related protein genes were collected from NCBI, i5k, Ensembl and FlyBase. BLAT searches were then performed at the i5k workspace to identify homologs in the *D. citri* genome. Potential hits within the *D. citri* genome were explored further using the WebApollo genome browser. NCBI Gnomon annotation gene models were manually annotated within WebApollo using BLAST and RNA-Seq evidence tracks. Completed gene models were analyzed by BlastP to confirm accuracy and completeness of the annotation.

Several models were found when searching the genome for the fibrinogen C-terminal domain. Sequences from completed annotated genes were used to perform BlastP searches against related insects and model organisms. Potential models found in this manner were used in BLASTp searches to verify. This includes gene models named angiopoietin, tenascin, and three fibrillin models.

Pairwise amino acid sequence comparisons were performed between *D citri*, *Acyrthosiphon pisum*, *Drosophila melanogaster*, *C. lectularius*, *Anopheles gambiae*, *A. aegypti*, and *C. quinquefasciatus*. Collected orthologs and annotated *D. citri* FREP sequences were used to generate a multiple sequence alignment using ClustalW in MEGA6. Sequences were chosen to identify close relationships to proteins that include the FBG domain. The *D. citri* proteins were named per the NCBI BLAST results. A neighbor joining phylogenetic tree was produced from the multiple sequence alignment to analyze the evolutionary relationships between the *D. citri* FREPS and well characterized sequences. (Figure 1) The unrooted Neighbor-Joining phylogenetic tree was constructed with the MEGA6 program using the bootstrap method (with 1000 replications) as a test of phylogeny and p-distance method as the substitution model. The analysis includes 38 amino acid sequences with 53 positions in the data set of FREP genes proposed by NCBI.

**Results and Discussion**

Three FREPS were found within the *D. citri* genome. Several potential gene models had insufficient RNA sequence data and/or domains to facilitate an approved gene model. *Anopheles gambiae* has been shown to have 53 FREPS and *Drosophila melanogaster* to have 20 FREPS (Wang et al, 2005). (Table 1) It is likely the *D. citri* will have a closer amount to *D. melanogaster*, as it has been shown the FREPs have expanded in *A. gambiae* due to recognizing parasites from blood feeding.

One gene model is attributed to the ImmunoDB FREP ortholog search, scabrous protein, which includes the fibrinogen C-terminal domain. Scabrous protein has been shown in Drosophila melanogaster to be involved in the regulation of neurogenesis and (along with Gp150) as an endosomal protein that regulates Notch activity (Li et al, 2003). A search for the protein tenascin revealed one gene model although there are several members in the tenascin family. Tenascins are extracellular matrix proteins, which are associated with organogenetic processes, with neuronal and axon development, and mediate cell adhesion (Wang et al, 2005). A search for the protein angiopoietin revealed one gene model. Angiopoietin is involved with embryonic and postnatal angiogenesis. Other FREPS that were not found in the genome include ficolin, tachylectins and aslectin, which are likely involved in parasite detection within the innate immune system (Wang et al, 2005). Ficolin and tachylectin are involved with N-acetylglucosamine (GlcNAc) binding activity (Middah and Wang, 2008). Aslectin can also bind bacteria and GlcNAc.

The multiple sequence alignment and phylogenetic tree position the scabrous model in a separate clade alongside the other hemipteran scabrous proteins. The angiopoietin and tenascin models were also in the proper clades. (Figure 1)

Table 1: The numbers of named FREP gene copies in *Diaphorina citri*, *Acyrthosiphon pisum*, *Drosophila melanogaster*, *Cimex lectularius*, *Anopheles gambiae*, *Aedes aegypti*, and *Culex quinquefasciatus*. An extensive search was performed through NCBI search, ENSEMBL, and www.supfam.org to obtain final figures for numbers of FREP genes in other hemipterans.

| **Fibrinogen Related Proteins** | **Scabrous** | **MAP** | **Ficolin** | **Angiopoietin** | **Tenascin** | **Other** |
| --- | --- | --- | --- | --- | --- | --- |
| ***D. citri* a** | 1 | 0 | 0 | 1 | 1 | 0 |
| ***A. pisum* a** | 1 | 1 | 0 | 1 | 1 | 0 |
| ***C. lectularius* a** | 1 | 1 | 0 | 1 | 0 | N/A |
| ***D. melanogaster* a c** | 1 | 1 | 0 | 0 | 2 | 16 |
| ***A. gambiae* b c** | 1 | 0 | 2 | 1 | 0 | 55 |
| ***A. aegypti* b c** | 1 | 1 | 0 | 0 | 0 | 33 |
| ***C. quinquefasciatus* a c** | 1 | 1 | 3 | 3 | 2 | 83 |

**a** NCBI search, **b** ENSEMBL, **c** [www.supfam.org](http://www.supfam.org)

Table 2: FREP sequence table. The protein sequences used for the phylogenetic analysis with their corresponding BLAST match results for specific species, with the bit scores followed by the coverage %, ID%, and accession numbers.

| ***D. citri* Gene Named** | ***D. citri* AA Length** | ***A. pisum* BLAST Match Bit Score** | ***BLAST Query Percent*** | ***BLAST ID Percent*** | ***A. pisum* Acc #** | ***D. melanogaster*  BLAST Match Bit Score** | ***BLAST Query Percent*** | ***BLAST ID Percent*** | ***D. melanogaster* Acc #** |
| --- | --- | --- | --- | --- | --- | --- | --- | --- | --- |
| Scabrous | 897 | 434 | 86 | 83 | XP_001951011.3 | 419 | 87 | 78 | NP_001188463.1 |
| Angiopoietin | 567 | 337 | 99 | 32 | XP_001944698.3 | 337 | 95 | 33 | AAA28880.1 |
| Tenascin | 977 | 313 | 72 | 31 | XP_008178803.2 | 57.8 | 9 | 33 | NP_609691.6 |

MAP

Tenascin

Fibrillin

Protein Scabrous

Angiopoietin / Ficolin

Figure 1: Phylogenetic analysis of Hemipteran FREP family protein sequences

The unrooted Neighbor-Joining phylogenetic tree was constructed with the MEGA6 program using the bootstrap method (with 1000 replications) as a test of phylogeny and p-distance method as the substitution model. The analysis includes 38 amino acid sequences with 53 positions in the data set of FREP genes proposed by NCBI. All bootstrap values are shown near the nodes. The *D. citri* proteins are numbered in the order found. The *A. pisum* proteins were named according to their EST numbers*.* The *D. Melanoaster, A gambiae,* and proteins were renamed from their accession number name. Abbreviations: *Diaphorina citri* Dc; *Acyrthosiphon pisum* Ap; *Anopheles gambiae* Agb; *Drosophila melanogaster* Dme; *Cimex lectularius* Cle, *Aedes aegypti* Aae, and *Culex quinquefasciatus* Cq.

**References**

1. Identification and characterization of the fibrinogen-like domain of fibrinogen-related proteins in the mosquito, Anopheles gambiae, and the fruitfly, Drosophila melanogaster, genomes. Xinguo Wang, Qin Zhao, and Bruce M Christensen. BMC Genomics20056:114 Wang et al; licensee BioMed Central Ltd. 2005. DOI: 10.1186/1471-2164-6-114
2. Scabrous and Gp150 are endosomal proteins that regulate Notch activity. Yanxia Li, Michael Fetchko, Zhi-Chun Lai, Nicholas E. Baker. Development 2003 130: 2819-2827; doi: 10.1242/dev.00495
3. The Scabrous protein can act as an extracellular antagonist of Notch signaling in the Drosophila wing. Lee, E-Chiang et al. Current Biology , Volume 10 , Issue 15 , 931 – 934. DOI: <http://dx.doi.org/10.1016/S0960-9822(00)00622-9>
4. Hanington P, C, Zhang S, -M. The Primary Role of Fibrinogen-Related Proteins in Invertebrates Is Defense, Not Coagulation. J Innate Immun 2011;3:17-27 DOI: 10.1159/000321882
5. Evolution and potential function of fibrinogen-like domains across twelve Drosophila species. Sumit Middha and Xinguo Wang. BMC Genomics20089:260 DOI: 10.1186/1471-2164-9-260

# Supplementary Note 4: PGRP and 𝛽GRP

**Introduction**

PGRPs are receptors that recognize and bind to peptidoglycan molecules which can be found in the cell walls of bacteria (Sun *et al.*, 2014). They have been conserved from insects to mammals as they are important in the detection of pathogens. The PGRP gene family is split into different size groups, two being PGRP long (30-90kDA) and PGRP small (20kDA) (Hoffmann, 2003). PGRP-S genes make up about half of the PGRP gene family present in the genome of *Drosophila melanogaster*, 7 out of 13 genes (Hoffmann, 2003). PGRP-S is an extracellular protein that bind to pathogen associated molecular patterns (PAMPs) which will cause the production of an immune response directing immune cells to degrade a pathogen or leading to the transcription of antimicrobial peptides via signaling pathways like Toll or IMD (Hoffmann, 2003). So far there has been one small PGRP found in the citrus psyllid’s genome, but with the PGRPs being a first line of defense in the insect immune response, targeting them could result in a lack of cellular function that could dampen the *Diaphorina citri’s* immune system and response.

**Materials and Methods**

The PGRP sequences from the following insects were used to search the genome of *Diaphorina citri*: *Drosophila melanogaster, Anopheles gambiae, Aedes aegypti,* *Culex pipiens* and *Cimex lectularius*. These sequences were used in the BLAT search tool in Web Apollo, BLAST from I5K, and the MCOT database of the citrus psyllid to find gene model predictions. The resulting hit was annotated using transcript and RNA sequences tracks as support.

Blastp was used to perform a pairwise alignment with other insects. The top hits from the previously mentioned insects plus the pea aphid were recorded along with accession, query, identity, and bit score information. The ExPasy Compute pl/MW tool was utilized to calculate the theoretical molecular weight of the annotated PGRP sequence. PGRP gene numbers were collected from Table 2 of Viljakainen, 2015.

The *D. citri* PGRP sequence and those from the following insects: *Culex quinquefasciatus* (XP_001849091.1, XP_001848058.1), Halyomorpha halys (XP_014294583.1), *Anopheles gambiae* (XP_001688526.1, XP_310547.4), *Drosophila melanogaster* (NP_573078.1, NP_572727.1), *Aedes Aegypti* (XP_001660103.2, XP_001661789.2), and *Cimex lectularius* (XP_014247856.1) were used to construct a multiple sequences alignment in MEGA7 with default MUSCLE settings. Subsequently, a neighbor-joining tree was made from the alignment with p-distance, 1000 bootstrap replicates, and complete deletion.

**Results and Discussion**

In the search for the PGRPs in the citrus psyllid’s genome one model was found after searches were conducted using BLAT, I5K BLAST, and MCOT search. The sequence collected from ACP contained one PGRP domain. The compute pl/MW tool was used to calculate the molecular weight of the PGRPS protein and result was about 23.85 kDa consistent with previous knowledge on PGRPs.

It was a surprise that there was only one gene found in version 1 of the psyllid genome (Table 1). On the other hand, it has been shown that the PGRPs are missing in the pea aphid, which is a closely related hemipteran to ACP (Table 1; Gerardo *et al.*, 2010). A Blastp pairwise alignment showed the sequence top matches were small PGRPs having two of the highest bit scores (Table 2). The neighbor-joining tree distance shows that the psyllid PGRP is closer to the small PGRPs (Figure 1). In the future this gene could be targeted as a means of possibly disrupting the insect’s immunity.

Table 1: PGRP family member gene number in *D. citri, D. melanogaster, A. gambiae, A. pisum, P. humuculus, B. mori*, and *T. castanium* (Gerardo et al., 2010; Viljakainen 2015).

| **PGRP gene count in *D. citri* and other insects** |  |
| --- | --- |
|  | PGRP |
| ***Drosophila melanogaster*** | 13 |
| ***Anopheles gambiae*** | 11 |
| ***Acyrthosiphon pisum*** | 0 |
| ***Bombyx mori*** | 12 |
| ***Pediculus humanus*** | 1 |
| ***Tribolium castaneum*** | 7 |

Table 2: Top Blastp hit found when alignment the PGRP from *D. citri* with *D. melanogaster, A. aegypti, C. quiquefasciatus, A. gambiae,* and *C. lectularius.* Information includes: match description, accession, query, identity, and bit score.

| **Blastp results for PGRP-S in *D. citri*** | | | | | |
| --- | --- | --- | --- | --- | --- |
| **Organism** | **Match** | **Accession** | **Query** | **ID** | **Bit score** |
| *Drosophila melanogaster* | peptidoglycan recognition protein SB1 | CAD89128.1 | 47% | 38% | 222 |
| *Aedes aegypti* | peptidoglycan recognition protein (Long) | XP_001654275.1 | 48% | 40% | 217 |
| *Culex quinquefasciatus* | peptidoglycan recognition protein sb2 | XP_001849091.1 | 55% | 38% | 217 |
| *Anopheles gambiae* | peptidoglycan recognition protein (long) | XP_558600.3 | 70% | 27% | 175 |
| *Cimex lectularius* | PREDICTED: peptidoglycan-recognition protein SB2-like isoform X4 | XP_014247859.1 | 47% | 30% | 136 |

**
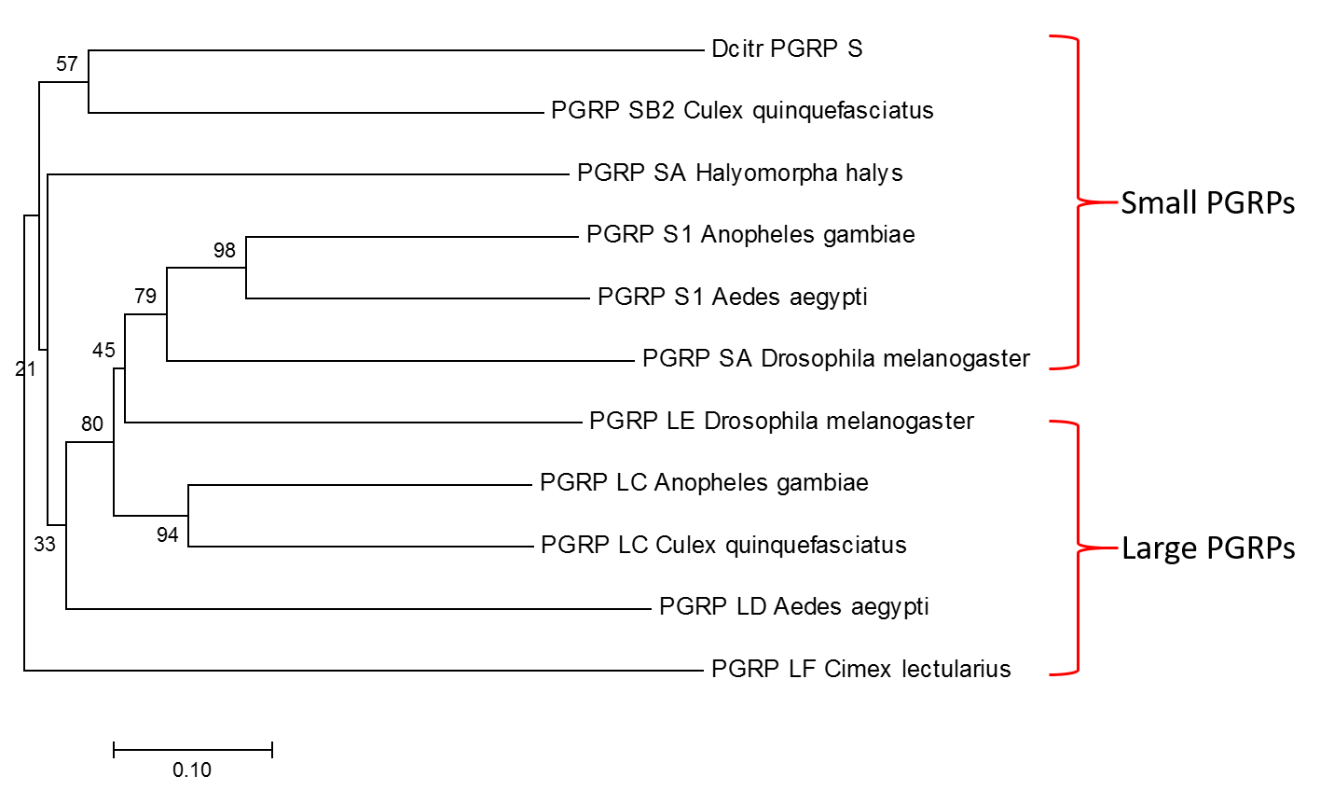
**

Figure 1: Neighbor-Joining tree constructed using small and large PGRP sequences from *D. citri* and other insects. The branches shown that the D. citri PGRP is closer to the other PGRPs, especially *C. quinquefasiatus*. Bootstrap values based on 1000 replicates are shown at the nodes.

**References**

1. Dukhanina E, Lukyanova T, Romanova E, Guerriero V, Gnuchev N, Georgiev G, Yashin D, Sashchenko L. 2015. A new role for PGRP-S (Tag7) in immune defense: lymphocyte migration is induced by a chemoattractant complex of Tag7 with Mts1. Cell Cycle 14(22):3635-3643.
2. Dziarski R, Gupta D. 2006. The peptidoglycan recognition proteins (PGRPs). Genome biology 7(8):1.
3. Gerardo NM, Altincicek B, Anselme C, Atamian H, Barribeau SM, De Vos M, Duncan EJ, Evans JD, Gabaldón T, Ghanim M. 2010. Immunity and other defenses in pea aphids, Acyrthosiphon pisum. Genome biology 11(2):1.
4. Hoffmann JA. 2003. The immune response of Drosophila. Nature 426(6962):33-38.
5. Paredes JC, Welchman DP, Poidevin M, Lemaitre B. 2011. Negative regulation by amidase PGRPs shapes the Drosophila antibacterial response and protects the fly from innocuous infection. Immunity 35(5):770-779.
6. Sharma P, Dube D, Yadav SP, Sinha M, Kaur P, Sharma S, Singh TP. 2014. Structural and functional studies of mammalian peptidoglycan recognition protein, PGRP-S. Journal of the Indian Institute of Science 94(1):109-118.
7. Sun Q, Xu X-X, Freed S, Huang W-J, Zheng Z, Wang S, Ren S-X, Jin F-L. 2014. Molecular characterization of a short peptidoglycan recognition protein (PGRP-S) from Asian corn borer (Ostrinia furnacalis) and its role in triggering proPO activity. World Journal of Microbiology and Biotechnology 30(1):263-270.
8. Tydell CC, Yuan J, Tran P, Selsted ME. 2006. Bovine peptidoglycan recognition protein-S: antimicrobial activity, localization, secretion, and binding properties. The Journal of Immunology 176(2):1154-1162.
9. Viljakainen L. 2015. Evolutionary genetics of insect innate immunity. Briefings in functional genomics:elv002.

# Supplementary Note 5: Thioester containing Proteins

**Introduction**

Thioester containing Proteins (TEPs) are members of an ancient protein family that includes vertebrate C complement and alpha-2-macroglobulin proteins (2). Insect TEPs seem to play a similar role to their vertebrate homologs, binding to invaders such as parasites or microbes, marking them for degradation (1). These proteins may be upregulated by the JAK/STAT pathway during innate immune response (1). In some TEPs, the interaction between invader and TEP occurs via a conserved thioester motif (GCGEQ), specifically TEP 1-4, but other TEPs lack this domain (2). TEPs also include a long C-terminal cysteine signature (2). Previous studies indicate that insect TEPs fall into three clades, with one clade being specific to mosquitos (3). Potential TEPs in *Diaphorina citri* were identified and investigated in comparison to sequences from other insects, including mosquito species, *Drosophila* species, and the pea aphid.

**Methods**

Investigation into potential TEPs in *Diaphorina citri* was initiated by first collecting known TEP sequences from other insects, including *Anopheles gambiae, Aedes aegypti, Culex quinquefasciatus, Drosophila melanogaster, Tribolium castaneum* and *Acrythosiphon pisum* (4) (7)*.* Using the gathered sequences, individual searches were conducted for each of the 46 orthologs provided using the protein blat and blast functions in i5k and NCBI, respectively. Two predicted proteins were implicated as those most closely related to the known TEPs of related organisms. The corresponding sequences were then extracted from NCBI, and searched within the online annotation program, WebApollo. In WebApollo, the NCBI predicted sequence, the RNA seq data, and the Maker predicted genes were used as reference for manual annotation. Using the NCBI predicted sequences as a base, exons were edited based on the evidence provided by the RNAseq data. After ensuring that the NCBI model corresponded as precisely as possible to the RNAseq data, a multiple sequence alignment was created through MUSCLE to determine whether the constructed *D. citri* model was similar to the known sequences from the previously listed organisms. MEGA 7 was used to perform a local alignment on all of these sequences, from which a proposed phylogenetic relationship was created, using a neighbor-joining phylogeny, as illustrated in Figure 1.

**Results and Discussion**

Based on the current gene assembly, only two TEPs were able to be identified within the psyllid, *Diaphorina citri.* This number of TEPs is comparable to that of *Acrythosiphon pisum* and *Nasonia vitripennis,* indicating that these are mostly likely the only two TEP genes in the psyllid’s genome, although isoforms may be present.

| **Organism** | **Number of TEPs** |
| --- | --- |
| *Drosophila melanogaster* | 6 |
| *Anopheles gambiae* | 13 |
| *Tribolium castaneum* | 4 |
| *Apis mellifera* | 4 |
| *Nasonia vitripennis* | 3 |
| *Acyrthosiphon pisum* | 2 |
| *Diaphorina citri* | 2 |

Table 1 : TEP in *D. citri* and related organisms (5)(6)

The first gene TEP3 (Thioester_containing_protein 3) is 28,982 base pairs in length. It includes 24 exons, which are typically short and evenly spread between long introns. It was found to contain the distinct GCGEQ motif towards the

C terminal.The second gene TEP6 (Thioester_containing_protein 6) is 30,776 base pairs in length. Within this gene there are 19 exons in three distinct clusters. The GCGEQ motif was not found within this protein, giving more evidence to its identity as an ortholog of TEP6. RNAseq data suggests that this model is one of many possible isoforms. Due to the length and the number of exons, determining the number of isoforms present was not possible at this time, and requires further investigation.

|  |  | ***A. pisum*** | ***D. melanogaster*** | ***T. castaneum*** |
| --- | --- | --- | --- | --- |
| Thioester containing protein 3 | Bit Score (name) | 1400 (CD109 antigen) | 704 (macroglobulin complement-related) | 981 (CD 109 antigen) |
|  | Query (Identity) | 95% (65%) | 95% (38%) | 96% (46%) |
|  | E value | 0 | 0 | 0 |
| Thioester containing protein 6 | Bit Score (name) | 1000 (CD109 antigen) | 805 (macroglobulin complement-related) | 834 (CD109 antigen) |
|  | Query (Identity) | 96% (58%) | 93% (67%) | 94% (69%) |
|  | E value | 0 | 0 | 0 |

Table 2: Blast matches of ACP TEP proteins to sequenced arthropods

Figure 1: Proposed phylogenetic relationship between Diaphorina citri (Dcitr), Acrythosiphon pisum (Apis), Tribolium castaneum (Tcas), Anopheles gambiae (Agam), Aedes aegypti (Aaeg), Culex quinquefasciatus (Cpip) and Drosophila melanogaster (Dmel)

**References**

1. Agaisse, H. and Perrimon, N. “The roles of JAK/STAT signaling in Drosophila immune responses.” Immunological Reviews (2004), 198: 72–82. doi:10.1111/j.0105-2896.2004.0133.x
2. Blandlin, Stephanie et al “Thioester-containing proteins and insect immunity.” *Molecular Immunology Vol 40* (2004) 903-908
3. Bou Aoun, Richard et al. “Analysis of Thioester-Containing Proteins during the Innate Immune Response of *Drosophila Melanogaster*.” *Journal of Innate Immunity* 3.1 (2010): 52–64. *PMC*. Web. 28 Sept. 2016.
4. Gerardo et al. “Immunity and other defenses in pea aphids” *Genome Biology* (2010)
5. Lagueux, Marie et al. “Constitutive Expression of a Complement-like Protein in Toll and JAK Gain-of-Function Mutants of *Drosophila*.” *Proceedings of the National Academy of Sciences of the United States of America* 97.21 (2000): 11427–11432. Print.
6. Viljakainen, Lumi “Evolutionary genetics of insect innate immunity” *Briefings in Functional Genomics* (2015)
7. Zou, Zhen et al. “Comparative genomic analysis of the Tribolium immune system” *Genome Biology* (2007)

# Supplementary Note 6: Toll Receptors

**Introduction**

The Toll pathway is widely conserved in animals and plays a role in innate immunity in both vertebrates and insects. Toll receptors are transmembrane proteins characterized by extracellular leucine-rich repeats (LRRs) and cysteine-rich clusters, as well as a cytoplasmic Toll/Interleukin-1R (TIR) domain (see Imler and Zheng, 2004). Vertebrate Toll receptors, known as Toll-like receptors (TLRs) act as pattern recognition receptors and are directly activated by microbial components. In contrast, insect Toll receptors are indirectly activated by microbial components. Secreted pattern recognition receptors bind microbial molecules and then initiate a protease cascade that results in the cleavage of Spätzle. The cleaved form of Spätzle is able to bind to the Toll receptor, which then activates a downstream signal transduction pathway leading to the transcription of antimicrobial peptides (see Valanne et al. 2011)

The founding member of the Toll receptor family was the *Drosophila melanogaster* gene *Toll*, which was initially identified because of its role in embryonic development (Nüsslein-Volhard and Wieschaus, 1980). Eight more Toll receptor genes (*Toll-2* through *Toll-*9) have been found in the *D. melanogaster* genome*.* Comparison of the Toll receptors found in various insects suggests that there were only six ancestral Toll receptors: *Toll-1*, *Toll-6*, *Toll-2/7*, *Toll-8*, *Toll-9* and *Toll-10* (Evans et al. 2006; Benton et al. 2016). Extant insects have different numbers of Toll receptors due to lineage-specific duplications and losses (see Table 1). Most hemipterans whose genomes have been sequenced have one member of each ancestral class except *Toll-*9 (Gerardo et al. 2010, Bao et al. 2013, Wang et al. 2015, Benoit et al. 2016). The only hemipteran in which *Toll-9* has been found is the milkweed bug, *Oncopeltus fasciatus* (Benton et al. 2016). The only Toll receptor expansion reported in a hemipteran is in the pea aphid (*Acyrthosiphon pisum*), which has three *Toll-1* class genes (Gerardo et al. 2010).

**Methods**

Toll receptor genes from other insects were used to query the predicted *D. citri* protein sets (Diacit_International_psyllid_consortium_proteins_v1 and Diacit_RefSeq_proteins_Release_100) at i5k@NAL. The loci encoding the matching proteins were identified and manually annotated in Web Apollo. BLAST searches to compare *D. citri* proteins to other insect proteins were performed at NCBI and Flybase. We also performed BLAST searches of the *D. citri* MCOT set to compare our gene models to *de novo* assembled transcripts when possible and to search evidence of genes not found in the current genome assembly. Multiple alignments of the predicted *D. citri* isoforms and their homologs were performed in Muscle (<http://www.ebi.ac.uk/Tools/msa/muscle/>). We used MEGA7 to construct a phylogenetic tree via the neighbor-joining method. Sequences for multiple alignment and phylogenetic analysis were obtained from NCBI, FlyBase, ImmunoDB and the Bordenstein Lab (NSF DEB-1046149).

**Results and Discussion**

Using Toll receptor proteins from other insects to BLAST against *D. citri* predicted proteins, we identified four Toll receptors encoded by the *D. citri* genome. After manual annotation of these genes, we used the edited models to BLAST the MCOT database. When possible, we used *de novo* assemblies from MCOT as independent tests of our models. In one case (*Toll-1*), this method revealed repeated exons in the gene model due to improper contig assembly. We were able to improve the model by removing the repeated exons, although the model is still missing sequence that is present in the MCOT transcript but is not found in the assembled contig.

Figure 1. Neighbor-joining tree of Toll receptor proteins. Dm=*Drosophila melanogaster*, Of=*Oncopeltus fasciatus*, Nv=*Nilaparvata lugens*, Dcitr=*Diaphorina citri*

We were able to determine the apparent orthology of each of the Toll receptor genes by reciprocal BLAST searches (Table 2) and phylogenetic analysis (Fig. 1). Based on these results, we named the genes *Toll-1*, *Toll-6*, *Toll-7* and *Toll-8*. No genes from the *Toll-9* or *Toll-10* classes were identified in the current assembly. We also searched the MCOT database for genes from these missing classes, but did not find any candidates.

In addition to the four typical Toll receptor genes, our BLAST searches also identified an ortholog of *Toll-13*, which has been found in other hemipteran species (Bao et al. 2013, Oncopeltus fasciatus Official Gene set v1.1. (Ag Data Commons <http://dx.doi.org/10.15482/USDA.ADC/1173142>). The protein encoded by *Toll-13* lacks the transmembrane region and the cytoplasmic Toll Interleukin-1 Receptor (TIR) domain, suggesting it might be secreted (Bao et al. 2013).

**References**

1. Bao, Y.-Y., Qu, L.-Y., Zhao, D., Chen, L.-B., Jin, H.-Y., Xu, L.-M., Cheng, J.-A., and Zhang, C.-X. (2013). The genome- and transcriptome-wide analysis of innate immunity in the brown planthopper, Nilaparvata lugens. Bmc Genomics *14*, 1–23.
2. Benoit, J., Adelman, Z., Reinhardt, K., Dolan, A., Poelchau, M., Jennings, E., Szuter, E., Hagan, R., Gujar, H., Shukla, J., et al. (2016). Unique features of a global human ectoparasite identified through sequencing of the bed bug genome. Nat Commun *7*, 10165.
3. Benton, M.A., Pechmann, M., Frey, N., Stappert, D., Conrads, K.H., Chen, Y.-T., Stamataki, E., Pavlopoulos, A., and Roth, S. (2016). Toll Genes Have an Ancestral Role in Axis Elongation. Curr Biol *26*, 1609–1615.
4. Evans, J., Aronstein, K., Chen, Y., Hetru, C., Imler, J.-L., Jiang, H., Kanost, M., Thompson, G., Zou, Z., and Hultmark, D. (2006). Immune pathways and defence mechanisms in honey bees Apis mellifera. Insect Mol Biol *15*, 645–656.
5. Gerardo, N., Altincicek, B., Anselme, C., Atamian, H., Barribeau, S., de Vos, M., Duncan, E., Evans, J., Gabaldón, T., Ghanim, M., et al. (2010). Immunity and other defenses in pea aphids, Acyrthosiphon pisum. Genome Biology *11*, R21.
6. Imler, J.-L., and Zheng, L. (2004). Biology of Toll receptors: lessons from insects and mammals. J Leukocyte Biol *75*, 18–26.
7. Nüsslein-Volhard, C., and Wieschaus, E. (1980). Mutations affecting segment number and polarity in Drosophila. Nature *287*, 795–801.
8. Valanne, S., Wang, J.-H., and Rämet, M. (2011). The Drosophila Toll Signaling Pathway. J Immunol *186*, 649–656.
9. (dataset) Vargas Jentzsch, Iris M.; Hughes, Daniel S. T.; Poelchau, Monica; Robertson, Hugh M.; Benoit, Joshua B.; Rosendale, Andrew J.; Armisén, David; Duncan, Elizabeth J.; Vreede, Barbara M. I. ; Jacobs, Chris G. C.; Berger, Chloe; Burnett, Denielle L.; Chang, Chun-che; Chen, Yen-Ta; Chipman, Ariel D.; Andrew Cridge; Crumière, Antonin Jean Johan; Peter Dearden; Erezyilmaz, Deniz F.; Cassandra Extavour; Friedrich, Markus; Horn, Thorsten; Hsiao, Yi-min; Jones, Jeffery W.; Jones, Tamsin E.; Khila, Abderrahman; Leask, Megan; Lovegrove, Mackenzie; Lu, Hsiao-ling; Lu, Yong; Nair, Ajay; Palli, Subba R.; Pick, Leslie; Porter, Megan L.; Refki, Peter; Rivera Pomar, Rolando; Roth, Siegfried; Sachs, Lena; Santos, Emilia; Seibert, Jan; Sghaier, Essia; Shukla, Jayendra N.; Suzuki, Yuichiro; Tidswell, Olivia; Traverso, Lucila; van der Zee, Maurijn; Viala, Séverine; Richards, Stephen; Panfilio, Kristen A. (2015). Oncopeltus fasciatus Official Gene set v1.1. Ag Data Commons. <http://dx.doi.org/10.15482/USDA.ADC/1173142>
10. Wang, L., Tang, N., Gao, X., Guo, D., Chang, Z., Fu, Y., Akinyemi, I., and Wu, Q. (2016). Understanding the immune system architecture and transcriptome responses to southern rice black-streaked dwarf virus in Sogatella furcifera. Sci Reports *6*, 36254.

# Supplementary Note 7: Spaetzle proteins

**Introduction**

Spaetzle proteins serve as ligands for Toll receptors. When a proper developmental or immune signal is received, Spaetzle is cleaved. This allows it to bind to the Toll receptor causing activation of downstream components of the Toll pathway (see Valanne et al 2011).

In Drosophila, there are six *spaetzle* genes, but the number of genes in other insects varies (see Viljakainen 2015 and Table 1). Phylogenetic analysis has shown that there are six major classes of *spaetzle* genes with one Drosophila *spaetzle* gene in each class (An et al. 2010, Sun et al. 2016).

| **Organism** | **Order** | **# of spätzle genes** |
| --- | --- | --- |
| *Tribolium castaneum* | Coleoptera | 7 |
| *Anopheles gambiae* | Diptera | 6 |
| *Drosophila melanogaster* | Diptera | 6 |
| *Acyrthosiphon pisum* | Hemiptera | 6 |
| *Nilaparvata lugens* | Hemiptera | 8 |
| *Apis mellifera* | Hymenoptera | 2 |
| *Nasonia vitripennis* | Hymenoptera | 9 |
| *Linepithsma humile* | Hymenoptera | 5 |
| *Bombyx mori* | Lepidoptera | 3 |

Table 1. Gene counts of *spätzle* orthologs in representative insects.

**Methods**

At least one ortholog from each of the six classes of *spätzle* genes was used to query the predicted *D. citri* protein sets (Diacit_International_psyllid_consortium_proteins_v1 and Diacit_RefSeq_proteins_Release_100) at i5k@NAL. Putative *D. citri* orthologs were identified and manually annotated in Web Apollo. The predicted proteins were BLASTed against Insecta with NCBI BLAST to verify orthology. We also performed BLAST searches of the *D. citri* MCOT set at citrusgreening.org to compare the predicted proteins to proteins encoded by *de novo*-assembled transcripts. We used MEGA7 to construct a phylogenetic tree via the neighbor-joining method. Sequences for phylogenetic analysis were obtained from NCBI, FlyBase, ImmunoDB and the Bordenstein Lab (NSF DEB-1046149).


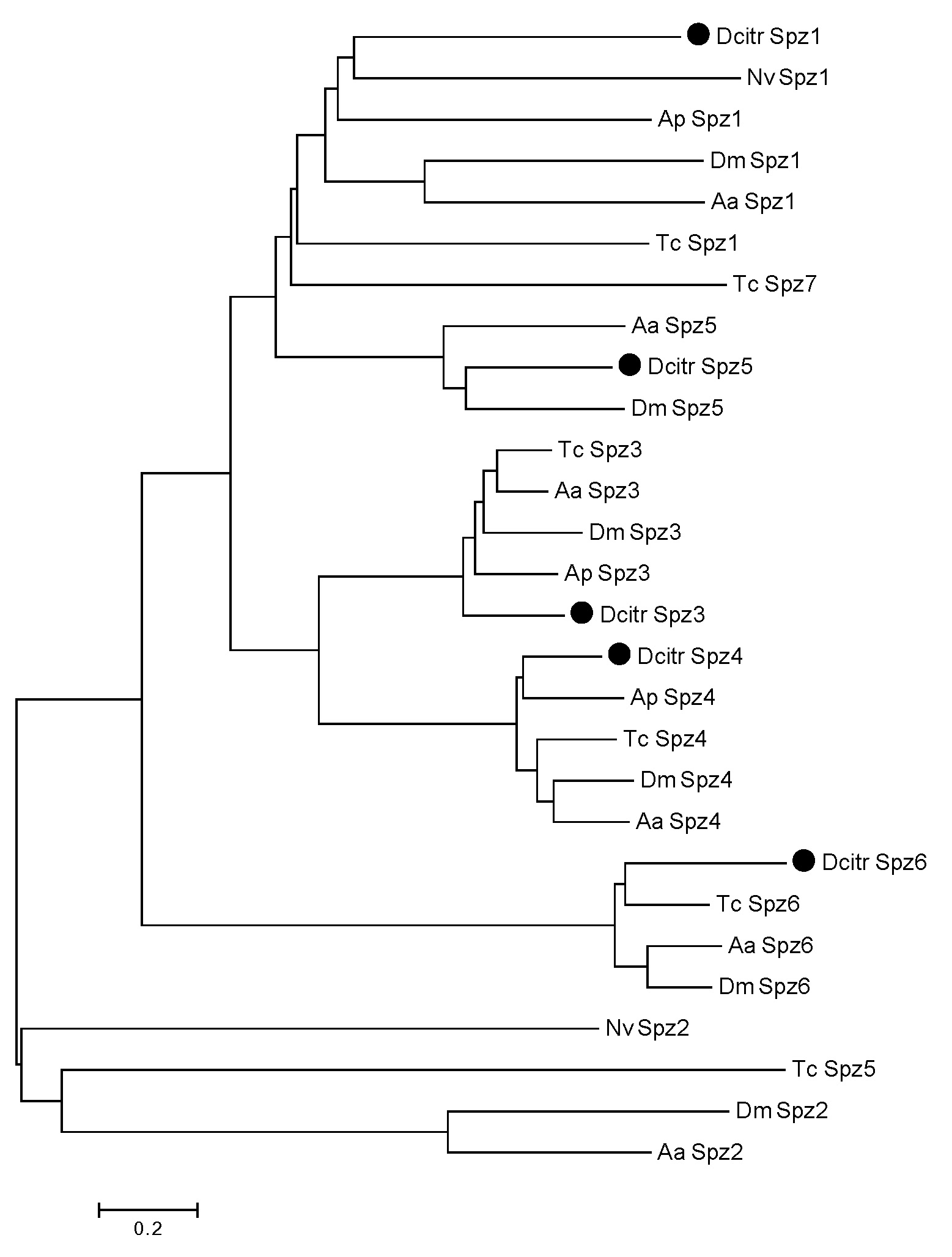


Figure 1. Phylogenetic tree of Spaetzle orthologs from *Diaphorina citri* (Dc), *Nasonia vitripennis* (Nv), *Acyrthosiphon pisum* (Ap), *Drosophila melanogaster* (Dm), *Aedes aegypti* (Aa) and *Tribolium castaneum* (Tc). *D. citri* proteins are marked with a black dot.

| **Predicted Protein** | **Top BLASTp hit** | **Bitscore** | **E value** | **Percent Identity** | **Top D.melanogaster BLASTp hit** | **Bitscore** | E value | Percent Identity |
| --- | --- | --- | --- | --- | --- | --- | --- | --- |
| Spaetzle1 (MCOT15644.0.CT) | PREDICTED: LOW QUALITY PROTEIN: uncharacterized protein LOC100871556 [Apis florea] | 110 | 9e-27 | 40% | spatzle, isoform I | 82.8 | 2e-18 | 31% |
| Spaetzle3  (MCOT16194.0.CT) | hypothetical protein g.16433 [Cuerna arida] | 395 | 8e-134 | 65% | spatzle 3 | 373 | 9e-124 | 54 |
| Spaetzle4  (MCOT04678.2.CT) | spaetzle 4 precursor [Acyrthosiphon pisum] | 335 | 2e-111 | 51% | spatzle 4 | 214  (316 total) | 8e-64 | 70% |
| Spaetzle5  (MCOT21079.2.CO) | hypothetical protein g.5364, partial [Clastoptera arizonana] | 267 | 8e-87 | 48% | spatzle 5 | 142  (217 total) | 3e-39 | 62% |
| Spaetzle6  (MCOT04452.0.CT) | PREDICTED: uncharacterized protein LOC109036501 [Bemisia tabaci] | 512 | 4e-179 | 60% | spatzle 6 | 392 | 1e-133 | 51% |

Table 2. BLAST results for *D. citri* Spaetzle proteins.

**Results and Discussion**

The six Drosophila Spaetzle proteins and, when possible, their pea aphid orthologs were used to BLAST *Diaphorina citri* (*D.* citri) predicted protein sets. These searches identified potential orthologs for Spaetzle 1, 3, 4, 5, and 6. The *D. citri* proteins were then used to BLAST the MCOT protein set. For each Spaetzle class, at least one protein encoded by a de novo-assembled transcript was identified. These provided an assembly-independent alternative to the gene models.

A single Spaetzle1 ortholog was found in *D. citri*. The most complete sequence was found as MCOT15644.0.CT*.*  This sequence mapped to scaffold gi|645505476|ref|NW_007378110.1, but problems with the assembly made it impossible to create a single accurate transcript. Some of the exons in the middle of the transcript were either missing or out of order. Therefore, two partial transcripts were created: spaetzle1 partial 5’ and spaetzle1 partial 3’.

No Spaetzle2 ortholog was identified in either the predicted proteins or in MCOT.

We identified one Spaetzle3 ortholog in *D. citri*. The most complete protein sequence comes from MCOT16194.0.CT. Because of incomplete genome assembly, the sequences encoding this protein are found on three different scaffolds. Therefore, there are three partial transcripts associated with *spaetzle3*. spaetzle3 partial 5’ is located on gi|645502920|ref|NW_007378693.1, spaetzle3 partial middle is on gi|645507426|ref|NW_007377648.1 and spaetzle3 partial 3’ is on gi|645506656|ref|NW_007377820.1.

A single ortholog of Spaetzle4 was identified in *D. citri*. The MCOT04678.2.CT protein has a slightly longer C-terminus than the protein predicted by the best gene model. The exon encoding this additional C-terminal sequence is located in the wrong position in the assembly, so it was not included in the annotated transcript spaetzle4 partial.

One ortholog of Spaetzle5 was found in *D. citri*. Two MCOT proteins (MCOT21079.2.CO and MCOT21079.1.CO) matched the predicted protein from the Spaetzle5 gene model. These proteins differed in one stretch of amino acids, apparently as a result of the presence or absence of an alternative exon. We created two alternative models, one containing the alternative exon and one without it. The MCOT proteins also had additional N-terminal sequence compared to the gene model. This N-terminal sequence could not be located in the gi|645504497|ref|NW_007378346.1 scaffold. Therefore, we believe our annotated transcripts to be partial and named them spaetzle5 partial-RA and spaetzle5 partial-RB.

A single ortholog of Spaetzle6 was identified in *D. citri*. MCOT04452.0.CT appears to represent the most complete sequence available. The exons encoding the MCOT04452.0.CT protein map to gi|645504930|ref|NW_007378241.1, but the order is not consistent with the MCOT protein. This appears to be a result of improper assembly. Only the exons from the 3’ portion of the gene could be included in the gene model, so we named the transcript spaetzle6 partial.

The MCOT Spaetzle proteins from *D. citri* were used to BLAST against insect proteins and against *Drosophila melanogaster* proteins. The results confirmed the orthology of the *D. citri* proteins to the expected *Drosophila* proteins. (Table 2). A phylogenetic tree was also constructed using the *D. citri* MCOT Spaetzle proteins, as well as Spaetzle proteins from several other insects (Figure 1). In all cases, the *D. citri* Spaetzle proteins clustered with the expected class.

**References**

1. An, C., Jiang, H., and Kanost, M. (2010). Proteolytic activation and function of the cytokine Spätzle in the innate immune response of a lepidopteran insect, Manduca sexta. Febs J *277*, 148–162.
2. Sun, Y., Jiang, Y., Wang, Y., Li, X., Yang, R., Yu, Z., and Qin, L. (2016). The Toll Signaling Pathway in the Chinese Oak Silkworm, Antheraea pernyi: Innate Immune Responses to Different Microorganisms. Plos One *11*, e0160200.
3. Valanne, S., Wang, J.-H., and Rämet, M. (2011). The Drosophila Toll Signaling Pathway. J Immunol *186*, 649–656.
4. Viljakainen, L. (2015). Evolutionary genetics of insect innate immunity. Briefings Funct Genom *14*, 407–412.

# Supplementary Note 8: Tube proteins

**Introduction**

Tube is a component of the Toll pathway, which is important for development and innate immunity in insects. In *Drosophila*, Tube acts as the central component in a complex with MyD88 and Pelle, each of which binds to a separate site in Tube’s death domain (Sun et al. 2002). This interaction, which occurs when Toll is activated, allows Pelle to phosphorylate the inhibitory protein Cactus, targeting it for degradation (Daigneault et al 2013). Degradation of Cactus allows the Nf-kappa B transcription factors Dorsal and/or Dif to enter the nucleus and activate target genes (Wu and Anderson, 1998)

The presence of a single ortholog of *tube* appears to be the general rule in insect genomes (see Viljakainen 2015 and Table 1). However, many of these orthologs differ from *Drosophila* Tube in that they have a protein kinase domain in addition to the conserved death domain (Towb et al. 2009). This discovery allowed Towb et al. (2009) to determine that Tube and its binding partner Pelle are actually paralogous genes, which arose from a very ancient duplication. They also concluded that Tube is the ortholog of vertebrate IRAK4, while Pelle is orthologous to vertebrate IRAK1.

| **Organism** | **Order** | **# of tube genes** |
| --- | --- | --- |
| *Tribolium castaneum* | Coleoptera | 1 |
| *Anopheles gambiae* | Diptera | 1 |
| *Drosophila melanogaster* | Diptera | 1 |
| *Acyrthosiphon pisum* | Hemiptera | 1 |
| *Nilaparvata lugens* | Hemiptera | 1 |
| *Apis mellifera* | Hymenoptera | 1 |
| *Nasonia vitripennis* | Hymenoptera | 1 |
| *Linepithsma humile* | Hymenoptera | 1 |
| *Bombyx mori* | Lepidoptera | 1 |

Table 1. Gene counts of *tube* orthologs in representative insects.

**Methods**

Tube orthologs were used to query the predicted *D. citri* protein sets (Diacit_International_psyllid_consortium_proteins_v1 and Diacit_RefSeq_proteins_Release_100) at i5k@NAL. A locus encoding a putative Tube ortholog was identified and manually annotated in Web Apollo. The predicted protein was BLASTed against Insecta with NCBI BLAST to verify its identity. We also performed a BLAST search of the *D. citri* MCOT set to compare our gene model to *de novo* assembled transcripts. Multiple alignments were performed in Muscle (<http://www.ebi.ac.uk/Tools/msa/muscle/>). We used MEGA7 to construct a phylogenetic tree via the neighbor-joining method. Sequences for multiple alignment and phylogenetic analysis were obtained from NCBI, FlyBase, ImmunoDB and the Bordenstein Lab (NSF DEB-1046149).

**Results**

We used the *Tribolium castaneum* Tube ortholog (Towb et al. 2009) to perform a BLAST search of the *Diaphorina citri* (*D. citri*) predicted proteins. Several almost identical predicted proteins encoded by loci on several different scaffolds were identified. Most of these scaffolds are rather short and they appear to be artifacts of incomplete assembly rather than separate loci. We chose the locus on the longest scaffold (gi|645501093|ref|NW_007379137.1) for annotation. The NCBI model (XM_008481952.1) was a good match with the RNA-seq data, so it was renamed *tube*.

The Tube predicted protein was then used to BLAST the *D. citri* MCOT protein set. Two proteins encoded by de novo-assembled transcripts were nearly exact matches. MCOT03326.3.CO has four amino acid differences scattered throughout the protein. MCOT03326.1.CT appears to be truncated at the N-terminus and also has hree of the four amino acid differences seen in MCOT03326.3.CO. Finding only minor differences between the MCOT proteins and the gene model protein increases our confidence in the accuracy of the gene model.

We next used Tube to BLAST against other insect proteins. The top hit is annotated as a putative plant-type serine-threonine protein kinase from the body louse *Pediculus humanus corporis*  (Table 2). Moving down the list were several interleukin-1 receptor-associated kinase 4-like (IRAK-4-like) proteins from other Hemipterans. Since Tube is orthologous to vertebrate IRAK4, it appears that these are Tube orthologs. However, the list also includes several good matches that are annotated as Pelle orthologs.

| **Predicted Protein** | **Top Blastp hit** | **Bitscore** | **E value** | **Percent Identity** |
| --- | --- | --- | --- | --- |
| Tube | serine-threonine protein kinase, plant-type, putative [Pediculus humanus corporis] | 295 | 3e-93 | 36% |

Table 2. BLAST analysis of the predicted Tube protein.

*D. citri* has a separate *pelle* ortholog (see *pelle* gene report), but we wanted to be sure that *tube* was really *tube* and not another *pelle* gene. In most insects, Tube and Pelle both have a death domain and a protein kinase domain. However, there are a few diagnostic residues that distinguish the two groups. Tube orthologs have previously been shown to share several conserved death domain residues that are required for interaction with MyD99 and Pelle (Towb et al. 2009). Multiple alignment with *Anopheles gambiae* Tube indicates that the three residues required for MyD99 interaction are perfectly conserved in Tube, but the residues involved in Pelle interaction are only weakly conserved. Most strikingly, a GP-A motif in the Pelle interaction site that is conserved in previously described Tube orthologs (Towb et al. 2009), is not found in Tube. The significance of these differences for interaction of Tube and Pelle in *D. citri* is not clear. Another diagnostic residue is found in the protein kinase domain. Tube orthologs have an arginine (R) preceding an aspartate (D) in sequence subdomain VI, putting them in the RD class of protein kinases (Towb et al. 2009). Pelle orthologs have a glycine (G) instead. Tube has an R in that position, as would be expected for a Tube ortholog. Taken together, these observations suggest that the Tube is, in fact, a Tube ortholog.

We constructed a phylogenetic tree to compare Tube and Pelle to Tube and Pelle orthologs from several other insects. As expected, Dcitri_Tube clustered with other Tube orthologs, while Dcitri_Pelle grouped with other Pelle orthologs (Fig. 1)

Figure 1. A phylogenetic tree constructed with Pelle and Tube orthologs from several insects.

**References**

1. Daigneault, J., Klemetsaune, L., and Wasserman, S. (2013). The IRAK Homolog Pelle Is the Functional Counterpart of IκB Kinase in the Drosophila Toll Pathway. Plos One *8*, e75150.
2. Sun, H., Bristow, B., Qu, G., and Wasserman, S. (2002). A heterotrimeric death domain complex in Toll signaling. Proc Natl Acad Sci *99*, 12871–12876.
3. Towb, Sun, and Wasserman, S.A. (2009). Tube Is an IRAK-4 Homolog in a Toll Pathway Adapted for Development and Immunity. J Innate Immun *1*, 309–321.
4. Viljakainen, L. (2015). Evolutionary genetics of insect innate immunity. Briefings Funct Genom *14*, 407–412.
5. Wu, L., and Anderson, K. (1998). Regulated nuclear import of Rel proteins in the Drosophila immune response. Nature *392*, 93–97.

# Supplementary Note 9: Pelle proteins

**Introduction**

Pelle is a protein kinase that is a component of the Toll pathway. In *Drosophila*, it is responsible for the phosphorylation of the inhibitory protein Cactus (Daigneault et al 2013), targeting it for degradation so that the transcription factor Dorsal and/or Dif can translocate to the nucleus and activate target genes (Wu and Anderson, 1998). Pelle activity requires the formation of a heterotrimeric complex with MyD88 and Tube (Sun et al. 2002)

Tube and Pelle are paralogs, arising from an ancient duplication before the separation of the vertebrate and invertebrate lineages. They are orthologs of vertebrate IRAK4 and IRAK1, respectively (Towb et al. 2009). In Drosophila, Pelle and Tube are easily distinguished because Tube has no protein kinase domain. However, in many other insects both proteins have a death domain and a protein kinase domain (Towb et al. 2009. *pelle* is a single-copy gene in those insects that have been sequenced (see Viljakainen 2015 and Table 1).

| **Organism** | **Order** | **# of tube genes** |
| --- | --- | --- |
| *Tribolium castaneum* | Coleoptera | 1 |
| *Anopheles gambiae* | Diptera | 1 |
| *Drosophila melanogaster* | Diptera | 1 |
| *Acyrthosiphon pisum* | Hemiptera | 1 |
| *Nilaparvata lugens* | Hemiptera | 1 |
| *Apis mellifera* | Hymenoptera | 1 |
| *Nasonia vitripennis* | Hymenoptera | 1 |
| *Linepithsma humile* | Hymenoptera | 1 |
| *Bombyx mori* | Lepidoptera | 1 |

Table 1. Gene counts of *pelle* orthologs in representative insects.

**Methods**

Pelle orthologs were used to query the predicted *D. citri* protein sets (Diacit_International_psyllid_consortium_proteins_v1 and Diacit_RefSeq_proteins_Release_100) at i5k@NAL. The best hits was BLASTed against Insecta with NCBI BLAST to check their orthology. The locus encoding Pelle was identified and manually annotated in Web Apollo. We also performed a BLAST search of the *D. citri* MCOT set at citrusgreening.org. We used MEGA7 to construct a phylogenetic tree via the neighbor-joining method. Sequences for BLAST searches and phylogenetic analysis were obtained from NCBI, FlyBase, ImmunoDB and the Bordenstein Lab (NSF DEB-1046149).

**Results**

We used *Acyrthosiphon pisum* Pelle to BLAST against the *Diaphorina citri* (*D. citri*) predicted proteins at i5K. This identified a locus on gi|645505624|ref|NW_007378073.1 that was the clear best match. The next best hit (which had a much lower bitscore) was the Tube ortholog (see *tube* gene report), suggesting that there is only one *pelle* gene in the current *D. citri* assembly.

When the NCBI-predicted Pelle protein was used to BLAST against other insect proteins, the best matches were Pelle orthologs from other hemipterans (Table 2). These proteins show identity to the predicted Pelle protein in all regions except the C-terminus of the predicted protein. However, RNA-seq data indicates that the region encoding the C-terminus appears in the majority of the transcripts. Therefore, we accepted the NCBI annotation as the gene model for *pelle*. We then used Pelle to BLAST the MCOT predicted proteins. However, the only match was to another assembly-predicted protein, so we were unable to find independent verification of the gene model.

| **Predicted Protein** | **Top Blastp hit** | **Bitscore** | **E value** | **Percent Identity** |
| --- | --- | --- | --- | --- |
| Serine threonine-protein kinase pelle | PREDICTED: serine/threonine-protein kinase pelle [Acyrthosiphon pisum] | 435 | 6e-142 | 48% |

Table 2. BLAST analysis of the predicted Pelle protein

We constructed a phylogenetic tree containing Pelle and Tube orthologs from several insects. Pelle clearly clustered with other Pelle orthologs (Fig. 1), supporting our conclusion that the locus encodes a Pelle ortholog.

Figure 1. A phylogenetic tree constructed with Pelle and Tube orthologs from several insects.

**References**

1. Daigneault, J., Klemetsaune, L., and Wasserman, S. (2013). The IRAK Homolog Pelle Is the Functional Counterpart of IκB Kinase in the Drosophila Toll Pathway. Plos One *8*, e75150.
2. Sun, H., Bristow, B., Qu, G., and Wasserman, S. (2002). A heterotrimeric death domain complex in Toll signaling. Proc Natl Acad Sci *99*, 12871–12876.
3. Towb, Sun, and Wasserman, S.A. (2009). Tube Is an IRAK-4 Homolog in a Toll Pathway Adapted for Development and Immunity. J Innate Immun *1*, 309–321.
4. Viljakainen, L. (2015). Evolutionary genetics of insect innate immunity. Briefings Funct Genom *14*, 407–412.
5. Wu, L., and Anderson, K. (1998). Regulated nuclear import of Rel proteins in the Drosophila immune response. Nature *392*, 93–97.

# Supplementary Note 10: MyD88 proteins

**Introduction**

MyD88 is a component of the Toll pathway in both vertbrates and invertebrates. *Drosophila* MyD88 has a Toll/Interleukin 1-R (TIR) domain that binds to the Toll TIR (Horng and Medzhitov, 2001; Tauszig-Delamasure et al 2002) and a death domain that interacts with Tube (Sun et al 2002). The formation of a trimeric complex containing MyD88, Tube and Pelle (Sun et al. 2002) allows Pelle to phosphorylate Cactus (Daigneault et al 2013). Once Cactus is phosphorylated, it is degraded, releasing its inhibition of the transcription factors Dorsal and/or Dif and allowing them to move to the nucleus to activate their target genes (Wu and Anderson, 1998).

*MyD88* is found in a single copy in almost all insect genomes that have been examined (see Viljakainen 2015 and Table 1). Exceptions include the brown planthopper *Nilaparvata lugens*, which has two *MyD88* genes (Bao et al. 2015), and the diamondback moth *Plutella xylostella* where *MyD88* has not been found (Xia et al. 2015).

| **Organism** | **Order** | **# of MyD88 genes** |
| --- | --- | --- |
| *Tribolium castaneum* | Coleoptera | 1 |
| *Anopheles gambiae* | Diptera | 1 |
| *Drosophila melanogaster* | Diptera | 1 |
| *Acyrthosiphon pisum* | Hemiptera | 1 |
| *Nilaparvata lugens* | Hemiptera | 2 |
| *Apis mellifera* | Hymenoptera | 1 |
| *Nasonia vitripennis* | Hymenoptera | 1 |
| *Linepithsma humile* | Hymenoptera | 1 |
| *Bombyx mori* | Lepidoptera | 1 |
| *Plutella xylostella* | Lepidoptera | 0? |

Table 1. Gene counts of *MyD88* orthologs in representative insects. The question mark indicates that the absence of *MyD88* has not been proven.

**Methods**

*Oncopeltus fasciatus* MyD88 was used to query the predicted *D. citri* protein sets (Diacit_International_psyllid_consortium_proteins_v1 and Diacit_RefSeq_proteins_Release_100) at i5k@NAL. A locus encoding a putative MyD88 ortholog was identified and manually annotated in Web Apollo. The predicted protein was BLASTed against Insecta with NCBI BLAST to verify its identity. We also performed a BLAST search of the *D. citri* MCOT set at citrusgreening.org. We used MEGA7 to construct a phylogenetic tree via the neighbor-joining method. Sequences for phylogenetic analysis were obtained from NCBI, FlyBase, ImmunoDB and the Bordenstein Lab (NSF DEB-1046149).

**Results**

We used *Oncopeltus fasciatus* MyD88 to BLAST against *Diaphorina citri* (*D. citri*) predicted proteins at i5k. There were only two predicted proteins that showed significant identity to *Oncopeltus* MyD88, and both mapped to a single locus on gi|645507901|ref|NW_007377556.1. The two predicted proteins were encoded by NCBI gene model (XM_008489802.1) and maker gene model (maker-s119-augustus-gene-0.138-mRNA-1). The two models differed primarily at the 5’ end, with the maker model having additional 5’ exons. BLAST searches with the two predicted proteins showed that the maker model was a fusion of two genes. Comparison of the NCBI model to RNA-seq data suggested that it was missing one exon near the 3’ end, so we manually merged that exon from the maker model with the NCBI model. The new model was named *MyD88*. We performed a BLAST with MyD88 against MCOT to try to obtain independent verification for our model, but the only match was to another predicted from the genome assembly.

When MyD88 is used as a query against other insect proteins, the top hit is bed bug MyD88 (Table 2). The domain analysis from NCBI BLAST indicates that, as expected, MyD88 has both a death domain and a TIR domain.

| **Predicted Protein** | **Top BLASTp hit** | **Bitscore** | **E value** | **Percent Identity** |
| --- | --- | --- | --- | --- |
| MyD88 | PREDICTED: myeloid differentiation primary response protein MyD88 [Cimex lectularius] | 117 | 2e-27 | 31% |

Table 2. BLAST analysis of the predicted MyD88 protein.

We constructed a phylogenetic tree using MyD88 proteins from a variety of insects. Surprisingly, MyD88 appears to be a outgroup to all of the other proteins rather than clustering with the hemipteran MyD88 orthologs (Fig. 1). To understand this result, we compared BLAST alignments using either MyD88 or its closest match, bed bug (*Cimex lectularius*) MyD88, as the query sequence. The results suggest that MyD88 has diverged more rapidly than other MyD88 orthologs. While bed bug MyD88 is the best hit for MyD88, the converse is not true. MyD88 does not even appear on the list of top bed bug MyD88 matches. The stretches of identity shared between MyD88 and bed bug MyD88 are noticeably shorter than those shared by bed bug MyD88 with other insect orthologs. The significance of the rapid divergence of MyD88 is unclear, but it could have implications for Toll signaling in *D. citri*.

Figure 1. A phylogenetic tree constructed with MyD88 orthologs from *Aedes aegypti* (Aa), *Culex pipiens* (Cp), *Anopheles gambiae* (Ag), *Nasonia vitripennis* (Nv), *Oncopeltus fasciatus* (Of), *Cimex lectularius* (Cl), *Tribolium castaneum* (Tc), *Drosophila melanogaster* (Dm), and *Diaphorina citri* (Dcitr).

**References**

1. Bao, Y.-Y., Qu, L.-Y., Zhao, D., Chen, L.-B., Jin, H.-Y., Xu, L.-M., Cheng, J.-A., and Zhang, C.-X. (2013). The genome- and transcriptome-wide analysis of innate immunity in the brown planthopper, Nilaparvata lugens. Bmc Genomics *14*, 1–23.
2. Daigneault, J., Klemetsaune, L., and Wasserman, S. (2013). The IRAK Homolog Pelle Is the Functional Counterpart of IκB Kinase in the Drosophila Toll Pathway. Plos One *8*, e75150.
3. Horng, T., and Medzhitov, R. (2001). Drosophila MyD88 is an adapter in the Toll signaling pathway. Proc Natl Acad Sci *98*, 12654–12658.
4. Sun, H., Bristow, B., Qu, G., and Wasserman, S. (2002). A heterotrimeric death domain complex in Toll signaling. Proc Natl Acad Sci *99*, 12871–12876.
5. Tauszig-Delamasure, S., Bilak, H., Capovilla, M., Hoffmann, J., and Imler, J.-L. (2001). Drosophila MyD88 is required for the response to fungal and Gram-positive bacterial infections. Nat Immunol *3*, 91–97.
6. Viljakainen, L. (2015). Evolutionary genetics of insect innate immunity. Briefings Funct Genom *14*, 407–412.
7. Wu, L., and Anderson, K. (1998). Regulated nuclear import of Rel proteins in the Drosophila immune response. Nature *392*, 93–97.
8. Xia, X., Yu, L., Xue, M., Yu, X., Vasseur, L., Gurr, G., Baxter, S., Lin, H., Lin, J., and You, M. (2015). Genome-wide characterization and expression profiling of immune genes in the diamondback moth, Plutella xylostella (L.). Sci Reports *5*, 9877.

# Supplementary Note 11: Traf6 proteins

**Introduction**

TRAF6 is an adaptor protein that acts in several signaling pathways in the immune system of vertebrates (see Walsh et al. 2015 for review). This multifunctionality stems from its ability to interact with many different proteins. TRAF6 also acts as a non-conventional E3 ubiquitin ligase. In the Toll pathway, TRAF6 interacts with the IRAK complex to activate the TAK1 complex by ubiquitination (reviewed by Valanne et al. 2011).

There have been mixed reports as to whether the Drosophila ortholog of TRAF6, also known as dTRAF2, functions in Drosophila innate immunity. Shen et al. (2001) showed that TRAF6 can interact with Pelle *in vitro* and that the two proteins act synergistically to activate Dorsal when transfected into Schneider cells. Moreover, a null mutant of TRAF6 appears to have a deficient immune system, showing reduced transcription of the antimicrobial peptide genes *diptericin and drosomycin* in response to infection (Cha et al. 2003). In contrast, Sun et al. (2002) reported that RNAi against TRAF6 in cultured cells with constitutively active Toll receptors had no effect on the expression of a *drosomycin* reporter construct, suggesting that TRAF6 is not required for Toll signaling.

As in vertebrates, *Drosophila* TRAF6 has been shown to function in other pathways besides Toll. There have been reports of TRAF6 acting in JNK (Tang et al. 2013) and Notch (Mishra et al. 2014) signaling.

Most insects seem to have a single TRAF6 ortholog, although duplications have occasionally been described (Table 1).

| **Organism** | **Order** | **# of TRAF6 genes** |
| --- | --- | --- |
| *Tribolium castaneum* | Coleoptera | 1 |
| *Drosophila melanogaster* | Diptera | 1 |
| *Plutella xylostella* | Lepidoptera | 2 |
| *Bombyx mori* | Lepidoptera | 1 |
| *Cimex lectularius* | Hemiptera | 1 |

Table 1. Gene counts of *TRAF6* orthologs in several insects.

**Methods**

*Drosophila* TRAF6 was used to query the predicted *D. citri* protein sets (Diacit_International_psyllid_consortium_proteins_v1 and Diacit_RefSeq_proteins_Release_100) at i5k@NAL. A locus encoding a putative TRAF6 ortholog was identified and manually annotated in Web Apollo. The predicted protein was BLASTed against Insecta with NCBI BLAST to verify its identity. We also performed a BLAST search of the *D. citri* MCOT set at citrusgreening.org. Multiple alignments were performed using MUSCLE (<http://www.ebi.ac.uk/Tools/msa/muscle/>). We used MEGA7 to construct a phylogenetic tree via the neighbor-joining method. Sequences for phylogenetic analysis were obtained from NCBI, FlyBase, ImmunoDB and the Bordenstein Lab (NSF DEB-1046149).

**Results**

We used *Drosophila melanogaster* TRAF6 to BLAST against *Diaphorina citri* (*D. citri*) predicted protein sets. Matching proteins were encoded on two separate scaffolds, gi|645498610|ref|NW_007379755.1and gi|645508310|ref|NW_007377463.1. Multiple alignments showed that the proteins were virtually identical (data not shown), making it unlikely that they were actually separate loci. Both scaffolds had duplications of portions of the TRAF6-encoding sequence, probably due to assembly problems. The longest predicted protein on the longest scaffold (XP_008472878.1 on gi|645508310|ref|NW_007377463.1) was used to BLAST the MCOT data set. A protein encoded by a *de novo*-assembled transcript, MCOT13375.2.CT, differed by only one amino acid, suggesting that our gene model is accurate. We also BLASTed the predicted protein from our gene model against insect proteins at NCBI. The top matches were TRAF6 proteins (Table 2.) We therefore named our gene model TRAF6.

| **Predicted Protein** | **Top BLASTp hit** | **Bitscore** | **E value** | **Percent Identity** |
| --- | --- | --- | --- | --- |
| TRAF6 | TNF receptor-associated factor 6 [Papilio xuthus] | 157 | 3e-40 | 27% |

Table 2. BLAST analysis of the predicted TRAF6 protein.

We constructed a phylogenetic tree using predicted TRAF6 proteins from *D. citri* and other insects (Figure 1). Traf6 groups with TRAF6 from the bed bug, *Cimex lectularius.*

Figure 1. A phylogenetic tree constructed with TRAF6 orthologs from *Bombyx mori* (Bm), *Cimex lectularius* (Cl), *Tribolium castaneum* (Tc), *Drosophila melanogaster* (Dm), and *Diaphorina citri* (Dcitr).

**References**

1. Cha, G.-H., Cho, K., Lee, J., Kim, M., Kim, E., Park, J., Lee, S., and Chung, J. (2003). Discrete Functions of TRAF1 and TRAF2 in Drosophila melanogaster Mediated by c-Jun N-Terminal Kinase and NF-κB-Dependent Signaling Pathways. Mol Cell Biol*23*, 7982–7991.
2. Mishra, A., Sachan, N., Mutsuddi, M., and Mukherjee, A. (2014). TRAF6 is a novel regulator of Notch signaling in Drosophila melanogaster. Cell Signal *26*, 3016–3026.
3. Shen, B., Liu, Skolnik, E., and Manley, J. (2001). Physical and functional interactions between Drosophila TRAF2 and Pelle kinase contribute to Dorsal activation. Proc Natl Acad Sci *98*, 8596–8601.
4. Sun, H., Bristow, B., Qu, G., and Wasserman, S. (2002). A heterotrimeric death domain complex in Toll signaling. Proc Natl Acad Sci *99*, 12871–12876.
5. Tang, H.-W., Liao, H.-M., Peng, W.-H., Lin, H.-R., Chen, C.-H., and Chen, G.-C. (2013). Atg9 Interacts with dTRAF2/TRAF6 to Regulate Oxidative Stress-Induced JNK Activation and Autophagy Induction. Developmental Cell 489–503.
6. Valanne, S., Wang, J.-H., and Rämet, M. (2011). The Drosophila Toll Signaling Pathway. J Immunol *186*, 649–656.
7. Walsh, M., Lee, J., and Choi, Y. (2015). Tumor necrosis factor receptor‐ associated factor 6 (TRAF6) regulation of development, function, and homeostasis of the immune system. Immunological Reviews 72–92.

# Supplementary Note 12: Cactus proteins

**Introduction**

Cactus is an I kappa B factor that acts in the Toll pathway in insects (Geisler et al, 1992). In *Drosophila*, Cactus binds the NF-kappa B transcription factors Dorsal and Dif, keeping them in the cytoplasm. When the Toll pathway is activated by the presence of an infectious agent (usually Gram-positive bacteria or fungi), Cactus is phosphorylated and then degraded. This releases Dorsal or Dif to translocate to the nucleus to activate target genes such as antimicrobial peptides (see Valanne et al. 2011)

Most insects whose genomes have been sequenced have a single ortholog of Cactus (see Viljakainen 2015 and Table 1), although the honeybee *Apis mellifera* has three (Evans et al 2006) and the Argentine ant *Linepithsma* *humile* has two (Smith et al 2011).

| **Organism** | **Order** | **# of cactus genes** |
| --- | --- | --- |
| *Tribolium castaneum* | Coleoptera | 1 |
| *Anopheles gambiae* | Diptera | 1 |
| *Drosophila melanogaster* | Diptera | 1 |
| *Acyrthosiphon pisum* | Hemiptera | 1 |
| *Nilaparvata lugens* | Hemiptera | 1 |
| *Apis mellifera* | Hymenoptera | 3 |
| *Nasonia vitripennis* | Hymenoptera | 1 |
| *Linepithsma humile* | Hymenoptera | 2 |
| *Bombyx mori* | Lepidoptera | 1 |

Table 1. Gene counts of *cactus* orthologs in various insects

| **Predicted Protein** | **Top BLASTp hit** | **Bitscore** | **E value** | **Percent Identity** |
| --- | --- | --- | --- | --- |
| Cactus  (MCOT19824.0.CT) | PREDICTED: NF-kappa-B inhibitor cactus [Bemisia tabaci] | 212 | 6e-63 | 39% |

Table 2. BLAST analysis of the predicted Cactus protein.

**Methods**

*Acyrthosiphon pisum* Cactus was used to query the predicted *D. citri* protein sets (Diacit_International_psyllid_consortium_proteins_v1 and Diacit_RefSeq_proteins_Release_100) at i5k@NAL. The best hit was BLASTed against Insecta with NCBI BLAST to verify its identity. The locus encoding this protein was identified and manually annotated in Web Apollo. We also performed a BLAST search of the *D. citri* MCOT set to compare our gene model to *de novo* assembled transcripts. Multiple alignments were performed in Muscle (<http://www.ebi.ac.uk/Tools/msa/muscle/>). We used MEGA7 to construct a phylogenetic tree via the neighbor-joining method. Sequences for multiple alignment and phylogenetic analysis were obtained from NCBI, FlyBase, ImmunoDB and the Bordenstein Lab (NSF DEB-1046149).

**Results**

Using *Acyrthosiphon pisum* Cactus as a BLAST query, we identified a single *D. citri cactus* gene. An existing NCBI gene model was manually edited to include additional exons encoding motifs shared with other Cactus orthologs. However, comparing the predicted protein to orthologous proteins reveals that these motifs do not occur in the expected order, suggesting a possible problem with the genome assembly in this region. We then used the predicted Cactus protein to BLAST the MCOT predicted protein set. We identified a single MCOT protein (MCOT19824.0.CT), which perfectly matched several stretches of the predicted Cactus protein. Again, these regions were not in the same order. The region corresponding to the C-terminal portion of the MCOT protein was located in the middle of the assembly-predicted protein. Since the MCOT protein is a *de novo-*assembled transcript from Trinity and the order of its sequence matches that found in Cactus genes from other insects, we conclude that the genome is incorrectly assembled in this region causing the *cactus* exons to be in the wrong order. Therefore, we used the MCOT Cactus protein for the remainder of our analysis.

Using the MCOT Dcitri_Cactus protein to BLAST against insect proteins retrieves Cactus orthologs from many insects. The top hit is to the Cactus ortholog of another hemipteran, *Bemisia tabaci*, the silverleaf whitefly (Table 2). In a phylogenetic tree, MCOT Dcitri_Cactus also clusters with another hemipteran Cactus ortholog (Fig. 1)

Figure 1. A neighbor-joining tree of Cactus proteins from *Drosophila melanogaster* (Dm), *Tribolium castaneum* (Tc), *Acyrthosiphon pisum* (Ap), *Diaphorina citri* (Dcitr) and *Oncopeltus fasciatus* (Of).

**References**

1. Evans, J., Aronstein, K., Chen, Y., Hetru, C., Imler, J.-L., Jiang, H., Kanost, M., Thompson, G., Zou, Z., and Hultmark, D. (2006). Immune pathways and defence mechanisms in honey bees Apis mellifera. Insect Mol Biol *15*, 645–656.
2. Geisler, R., Bergmann, A., Hiromi, Y., and Nüsslein-Volhard, C. (1992). cactus, a gene involved in dorsoventral pattern formation of Drosophila, is related to the IκB gene family of vertebrates. Cell *71*, 613–621.
3. Smith, C., Zimin, A., Holt, C., Abouheif, E., Benton, R., Cash, E., Croset, V., Currie, C., Elhaik, E., Elsik, C., et al. (2011). Draft genome of the globally widespread and invasive Argentine ant (Linepithema humile). Proc Natl Acad Sci *108*, 5673–5678.
4. Valanne, S., Wang, J.-H., and Rämet, M. (2011). The Drosophila Toll Signaling Pathway. J Immunol *186*, 649–656.
5. Viljakainen, L. (2015). Evolutionary genetics of insect innate immunity. Briefings Funct Genom *14*, 407–412.

# Supplementary Note 13: Dorsal proteins

**Introduction**

Rel/NFκB transcription factors are widely conserved in the animal kingdom. These proteins are defined by the presence of a Rel Homology Domain (RHD), which includes a DNA binding domain and a protein dimerization domain. In insects, Rel proteins play important roles in both development and immunity. *Drosophila melanogaster*, where these genes have been best studied, has three Rel genes: *dorsal* (*dl*), *Dorsal-related immunity factor* (*Dif*) and *Relish* ([Ganesan et al., 2011](#_ENREF_5)). Dorsal and DIF both respond to Toll signaling ([Belvin and Anderson, 1996](#_ENREF_1); [Meng et al., 1999](#_ENREF_12)), while Relish responds to IMD signaling ([Hedengren et al., 1999](#_ENREF_8); [Khush et al., 2001](#_ENREF_11)). Relish also differs in that it contains ankyrin repeats ([Dushay et al., 1996](#_ENREF_4)).

**Methods**

Rel genes from other insects were used to query the predicted *D. citri* protein sets (Diacit_

International_psyllid_consortium_proteins_v1 and Diacit_RefSeq_proteins_Release_100) at i5k@NAL. The locus encoding the matching protein was identified and manually annotated in Web Apollo using RNA-Seq data to deduce the structure of transcripts. Multiple alignments of the predicted *D. citri* isoforms and their homologs were performed in Muscle (<http://www.ebi.ac.uk/Tools/msa/muscle/>). A phylogenetic tree was constructed using the neighbor-joining method in MEGA7. All sequences for multiple alignment and phylogenetic analysis were obtained from NCBI, FlyBase, ImmunoDB and the Bordenstein Lab (NSF DEB-1046149).

**Results and Discussion**


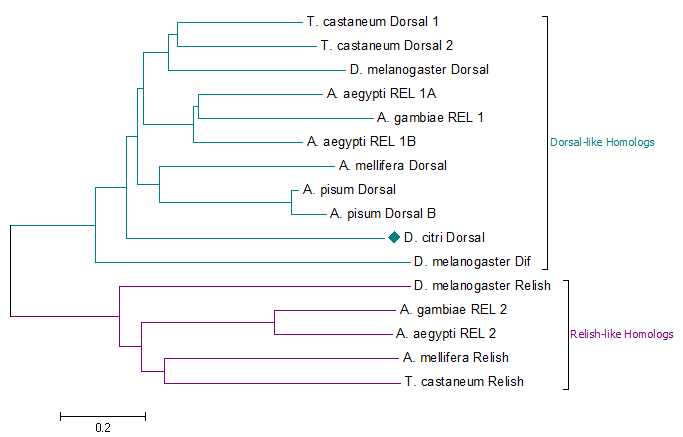


Figure 1: Phylogenetic tree depicting clustering of insect Rel proteins

In *D. citri*, BLASTp searches with known Rel proteins identified only one locus encoding a predicted Rel family member. The predicted RHD is much more similar to Dorsal than to either DIF or Relish. Moreover, the locus does not seem to encode the ankyrin repeats that are found in Relish orthologs. Consistent with these observations, phylogenetic analysis shows that the *D. citri* protein clusters with Dorsal proteins from other insects. Note that *dorsal* and *Dif* seem to be the result of a duplication in the *Drosophila* lineage ([Meng et al., 1999](#_ENREF_12); [Christophides et al., 2004](#_ENREF_3)). *Dif* has diverged more rapidly (hence its position as an outgroup), so homologs in other insects have generally been named *dorsal* ([Chen et al., 2000](#_ENREF_2); [Ursic-Bedoya et al., 2009](#_ENREF_18); [Gerardo et al., 2010](#_ENREF_6)). Following this convention, we will refer to this gene as *D. citri* *dorsal* (*dorsal*).

Table 1: Bit score, query coverage, identity and e-value results from blast analysis of predicted dorsal_RA and dorsal_RB proteins

In several insect species, *dorsal* genes produce alternative transcripts that generate different protein isoforms ([Gross et al., 1999](#_ENREF_7); [Shin et al., 2005](#_ENREF_16); [Zou et al., 2007](#_ENREF_19)). Therefore, it was not surprising that RNA-Seq data suggests that *dorsal* produces at least two alternative transcripts (*dorsal_RA* and *dorsal_RB*) that are very similar in structure to those seen in other insects.

Table 2: Rel/NFkB transcription factors in *D. citri* and related organisms

No ortholog of *Relish* was found in the *D. citri* genome. While it is possible that we were unable to find *Relish* due to gaps in the *D. citri* genome, the fact that *Relish* is missing in the pea aphid genome ([Gerardo et al., 2010](#_ENREF_6)) suggests that it could be absent in *D. citri* as well. It is interesting to note that both the pea aphid and the Asian citrus psyllid, two organisms known for their ability to host a variety of bacterial symbionts, appear to be lacking multiple members of the IMD pathway (including *Relish*) ([Gerardo et al., 2010](#_ENREF_6)). The IMD pathway is known to be involved in the detection and elimination of Gram negative bacteria ([Hedengren et al., 1999](#_ENREF_8)). Several Gram negative bacteria have been identified as *D. citri* symbionts, including the bacteria *Wolbachia*, *Candidatus* Carsonella, *Candidatus* Profftella armature, an as yet unidentified enteric bacteria closely related to *Klebsiella variicola* and *Salmonella enterica*, and *Candidatus* Liberibacter asiaticus, which causes citrus greening disease ([Jagoueix et al., 1994](#_ENREF_10); [Nakabachi et al., 2006](#_ENREF_14); [Hilgenboecker et al., 2008](#_ENREF_9); [Saha et al., 2012](#_ENREF_15); [Sloan and Moran, 2012](#_ENREF_17); [Nakabachi et al., 2013](#_ENREF_13)). Given this information it is tempting to speculate that a reduction in IMD pathway genes may in fact aid in *D. citri’s* ability to acquire and harbor these Gram negative symbionts.

**Reference**

1. Belvin, M. P. and Anderson, K. V. (1996) 'A conserved signaling pathway: the Drosophila toll-dorsal pathway', *Annu Rev Cell Dev Biol* 12: 393-416.
2. Chen, G., Handel, K. and Roth, S. (2000) 'The maternal NF-kappaB/dorsal gradient of Tribolium castaneum: dynamics of early dorsoventral patterning in a short-germ beetle', *Development* 127(23): 5145-56.
3. Christophides, G. K., Vlachou, D. and Kafatos, F. C. (2004) 'Comparative and functional genomics of the innate immune system in the malaria vector Anopheles gambiae', *Immunol Rev* 198: 127-48.
4. Dushay, M. S., Asling, B. and Hultmark, D. (1996) 'Origins of immunity: Relish, a compound Rel-like gene in the antibacterial defense of Drosophila', *Proc Natl Acad Sci U S A* 93(19): 10343-7.
5. Ganesan, S., Aggarwal, K., Paquette, N. and Silverman, N. (2011) 'NF-kappaB/Rel proteins and the humoral immune responses of Drosophila melanogaster', *Curr Top Microbiol Immunol* 349: 25-60.
6. Gerardo, N. M., Altincicek, B., Anselme, C., Atamian, H., Barribeau, S. M., de Vos, M., Duncan, E. J., Evans, J. D., Gabaldon, T., Ghanim, M. et al. (2010) 'Immunity and other defenses in pea aphids, Acyrthosiphon pisum', *Genome Biol* 11(2): R21.
7. Gross, I., Georgel, P., Oertel-Buchheit, P., Schnarr, M. and Reichhart, J. M. (1999) 'Dorsal-B, a splice variant of the Drosophila factor Dorsal, is a novel Rel/NF-kappaB transcriptional activator', *Gene* 228(1-2): 233-42.
8. Hedengren, M., Asling, B., Dushay, M. S., Ando, I., Ekengren, S., Wihlborg, M. and Hultmark, D. (1999) 'Relish, a central factor in the control of humoral but not cellular immunity in Drosophila', *Mol Cell* 4(5): 827-37.
9. Hilgenboecker, K., Hammerstein, P., Schlattmann, P., Telschow, A. and Werren, J. H. (2008) 'How many species are infected with Wolbachia?--A statistical analysis of current data', *FEMS Microbiol Lett* 281(2): 215-20.
10. Jagoueix, S., Bove, J. M. and Garnier, M. (1994) 'The phloem-limited bacterium of greening disease of citrus is a member of the alpha subdivision of the Proteobacteria', *Int J Syst Bacteriol* 44(3): 379-86.
11. Khush, R. S., Leulier, F. and Lemaitre, B. (2001) 'Drosophila immunity: two paths to NF-kappaB', *Trends Immunol* 22(5): 260-4.
12. Meng, X., Khanuja, B. S. and Ip, Y. T. (1999) 'Toll receptor-mediated Drosophila immune response requires Dif, an NF-kappaB factor', *Genes Dev* 13(7): 792-7.
13. Nakabachi, A., Ueoka, R., Oshima, K., Teta, R., Mangoni, A., Gurgui, M., Oldham, N. J., van Echten-Deckert, G., Okamura, K., Yamamoto, K. et al. (2013) 'Defensive bacteriome symbiont with a drastically reduced genome', *Curr Biol* 23(15): 1478-84.
14. Nakabachi, A., Yamashita, A., Toh, H., Ishikawa, H., Dunbar, H. E., Moran, N. A. and Hattori, M. (2006) 'The 160-kilobase genome of the bacterial endosymbiont Carsonella', *Science* 314(5797): 267.
15. Saha, S., Hunter, W. B., Reese, J., Morgan, J. K., Marutani-Hert, M., Huang, H. and Lindeberg, M. (2012) 'Survey of endosymbionts in the Diaphorina citri metagenome and assembly of a Wolbachia wDi draft genome', *PLoS One* 7(11): e50067.
16. Shin, S. W., Kokoza, V., Bian, G., Cheon, H. M., Kim, Y. J. and Raikhel, A. S. (2005) 'REL1, a homologue of Drosophila dorsal, regulates toll antifungal immune pathway in the female mosquito Aedes aegypti', *J Biol Chem* 280(16): 16499-507.
17. Sloan, D. B. and Moran, N. A. (2012) 'Genome reduction and co-evolution between the primary and secondary bacterial symbionts of psyllids', *Mol Biol Evol* 29(12): 3781-92.
18. Ursic-Bedoya, R., Buchhop, J. and Lowenberger, C. (2009) 'Cloning and characterization of Dorsal homologues in the hemipteran Rhodnius prolixus', *Insect Mol Biol* 18(5): 681-9.
19. Zou, Z., Evans, J. D., Lu, Z., Zhao, P., Williams, M., Sumathipala, N., Hetru, C., Hultmark, D. and Jiang, H. (2007) 'Comparative genomic analysis of the Tribolium immune system', *Genome Biol* 8(8): R177.

# Supplementary Note 14: Imd Pathway CASPAR (FAF1), IKKB, TAK1, TAB proteins

**Introduction**

Fas-associated factor-1 (FAF1) is a component of the death-inducing signing complex (DISC) and is able to enhance Fas-induced apoptosis when overexpressed. FAF1 is characterized by containing two ubiquitin-homologous domains in its N-terminus and a UX domain (Ryu et al., 2001, 2003). FAF1 is a homolog of *Drosophila* caspar (Chu et al., 1995; Ryu et al., 2003; Park et al., 2004). It has been shown that caspar suppresses the immune response by inhibiting nuclear localization of Relish induced by bacterial infection (Kim et al., 2006). Disruption of the IKK complex can occur when FAF1 binds to I-kappaB kinase-beta (IKKB) (Park et al., 2007). IKKB is the catalytic subunit of the IKK complex (~400 kDa) in IMD signaling (Silverman and Takashi, 2005; Silverman and Maniatis, 2001). In *Drosophila*, the IKK complex is responsible for phosphorylating Relish, which triggers cleavage of Relish mediated by DREDD. TAK1 (Transforming growth factor beta-activated kinase 1) activates the IKK complex and is required for expression of anti-microbial peptide genes through Relish activation (Silverman and Takashi, 2005).

**Methods**

Dipteran caspar, IKKB, and TAK1 orthologs were collected from Immunodb and were used to blat against the *Diaphorina citri*, Asian citrus psyllid (ACP), genome. Identification of the genes were performed in WebApollo where RNA sequence data (adult, nymph, and egg) supported the manually curated gene models. BLASTp was used to confirm accuracy of the annotated the gene models.

FAS-associated factor 1, IKKB, and TAK1 orthologs were collected through an NCBI search for the phylogenetic analysis. Multiple sequence alignments (MSA) of ACP and orthologous insect FAF1 protein sequences was performed in MEGA6 using ClustalW (Tamura et al 2003). MSA was repeated for IKKB and TAK1. The phylogenetic analysis of FAF1, IKKB, and TAK1 included, constructing two midpoint rooted neighbor-joining trees in MEGA6 using p-distance to show evolutionary distance and 1,000 bootstrap replicates.

**Results**

In the ACP genome, one FAF1 was identified along with two partials (Table 1). The two partials were located on small contigs, ~4,000 bp and below. Between hemipterans and dipterans, only one TAK1 and IKKB genes were found, including in the ACP (Table 1 & 2). TAB was found in the ACP, however, the N-terminus and C-terminus of the gene are on separate contigs. TAK1 is activated by overexpression of TAK1 interacting proteins, TAB (Shibuya et al. 1996). *D. citri* is missing many components of the IMD pathway, including DREDD, FADD, IMD, and IKKG (kenny). The pea aphid also have many IMD members missing (Gerardo et al., 2010). In *Drosophila*, this pathway is crucial to elicit an immune response when infected with Gram-negative bacteria (Boutros et al., 2002; Lemaitre et al., 1995).

Table 1: IMD pathway members found in the *D. citri* genome. Table shows members with BLASTp matches, corresponding protein length, and domains.

| **IMD members** | **BLASTP match**  **(organism, bit score)** | **Protein Length** | **Domains** |
| --- | --- | --- | --- |
| Fas-associated factor-1 | FAF1  (Zootermopsis nevadensis, 171) | 862 | UAS family, FAF1 subfamily, Faf1 UBX domain, UAS domain, UBX domain, UBA-like domain, thioredoxin (TRX)-like superfamily, hydrophobic patch, charged pocket |
| FAF1, partial | FAF1  (Locusta migratoria manilensis, 98.6) | 198 | UBA like domain, UBA like domain |
| FAF1, partial | FAF1  (Zootermopsis nevadensis, 117) | 152 | UAS family, FAF1 subfamily, thioredoxin (TRX)-like superfamily |
| TAK1 | TAK1  (Zootermopsis nevadensis, 367) | 257 | STKc_TAK1, KIND, PKc like superfamily, STYKc, Pkinase_Tyr, SPS1, PTZ00267, TOMM_kin_cyc |
| IKKB | IKKB  (Halyomorpha halys, 508) | 765 | DCX (6), XLIS mutations, SBH mutations, hydrophobic patch, charged pocket, active site, ATP binding site (2), polypeptide substrate binding site, UBQ superfamily (2), PKc like superfamily (2), STKc_DCKL, pk1, STYKc1, Pkinase, SPS1, Pkinase_Tyr, TOMM_kin_cyc (2), |

Table 2: Number of IMD pathway members in *D. citri* and related organisms.

| Organism | FAF1/caspar | TAK1 | IKKB |
| --- | --- | --- | --- |
| *Diaphorina citri* | 1 | 1 | 1 |
| *Acyrthosiphon pisum* | 1 | 1 | 1 |
| *Cimex lectularius* | 2 | 1 | 1 |
| *D. melanogaster* | 1 | 1 | 1 |
| *Aedes aegypti* | 2 | 1 | 1 |
| *Anopheles gambiae* | 2 | 1 | 1 |
| *Culex quinquefasciatus* | 1 | 1 | 1 |

**
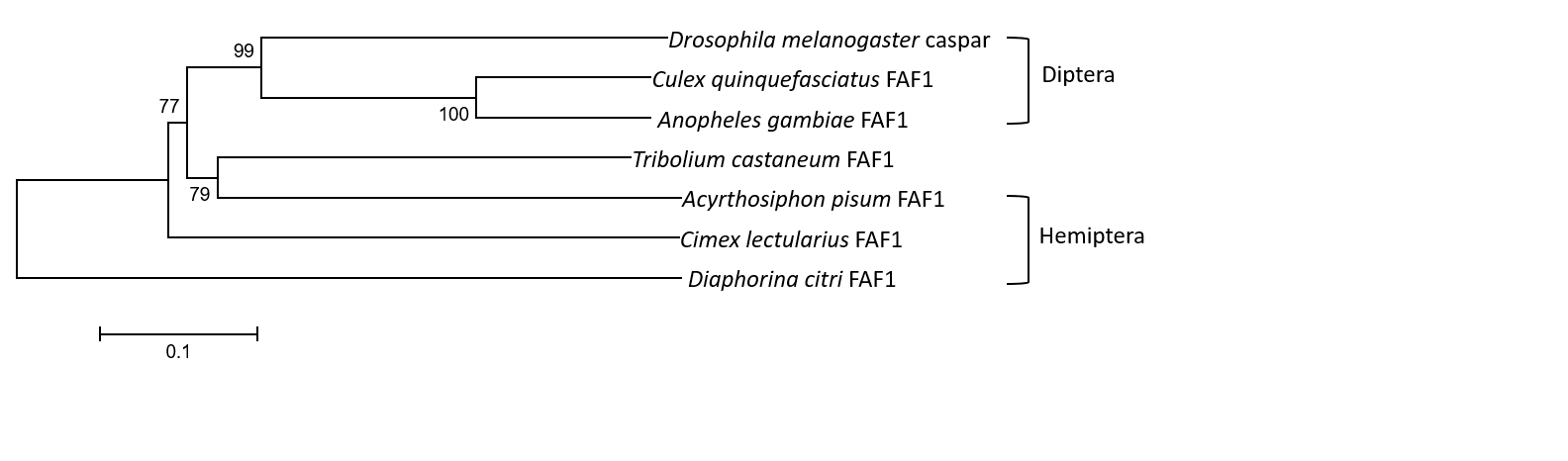
**Figure 1: Neighbor-joining tree of 7 FAF1 was constructed in MEGA6 using 1000 bootstrap replications. The p-distance method was used to show evolutionary distance. A total of 546 amino acid positions were analyzed for the tree. NCBI accessions: *A. pisum* [XP_008188590.1], *T. castaneum* [XP_008195902.1], *A. gambiae* [XP_316513.4], *C. quinquefasciatus* [EDS36965.1], *C. lectularius* [XP_014254010.1], *D. melanogaster* [NP_611080.1].


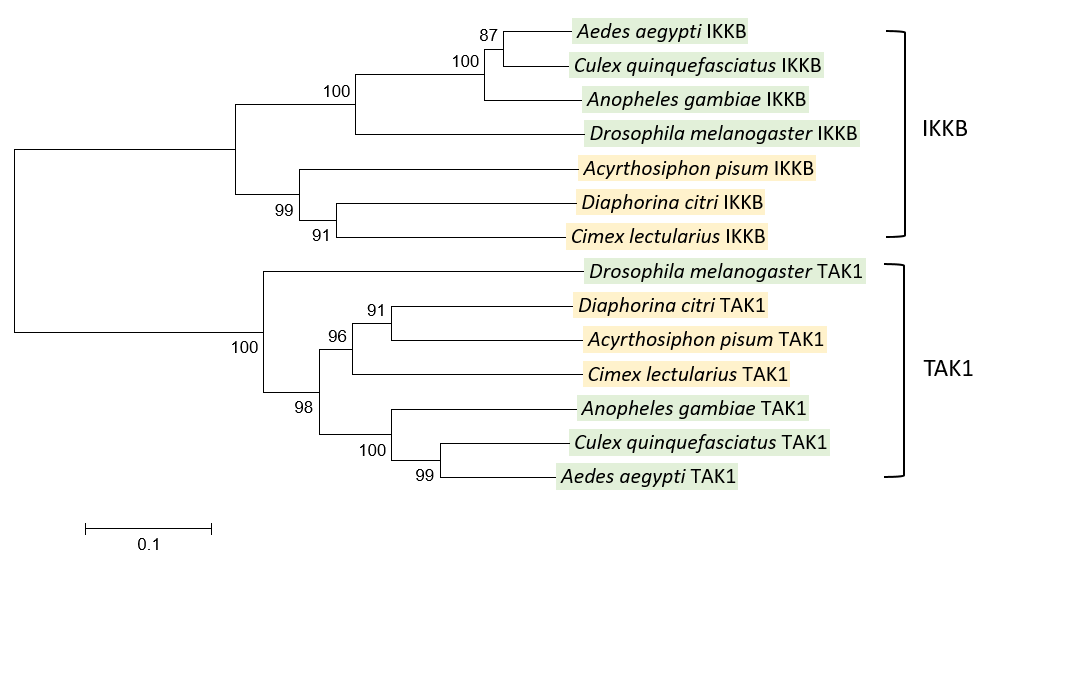


Figure 2: Neighbor-joining tree of 14 FAF1 was constructed in MEGA6 using 1000 bootstrap replications. The p-distance method was used to show evolutionary distance. A total of 237 amino acid positions were analyzed for the tree. Organisms highlighted in yellow and green belong to orders Hemipteran and Diptera, respectively.

The phylogenetic analysis (Figure 1) shows the clustering of ACP FAF1 with other hemipterans and the relationship between*. T. castaneum* (Order: Coleoptera) and Dipterans. Since, TAK1 and IKKB are both kinases, a neighbor-joining tree (Figure 2) was constructed showing the evolution of these proteins. Table 1 shows that TAK1 and IKKB share some domains.

**References**

1. Ryu, S. W., and Kim, E. (2001) Biochem. Biophys. Res. Commun. 286, 1027–1032
2. Ryu, S. W., Lee, S. J., Park, M.-Y., Jun, J. I., Jung, Y. K., and Kim, E. (2003) J. Biol. Chem. 278, 24003–24010
3. Kim M, Lee JH, Lee SY, Kim E, Chung J. (2006) Caspar, a suppressor of antibacterial immunity in Drosophila. PNAS. 103(44) 16358-16363
4. Chu K, Niu X, Williams LT (1995) Proc Natl Acad Sci USA 92:11894–11898.
5. Ryu SW, Lee SJ, Park MY, Jun JI, Jung YK, Kim E (2003) J Biol Chem 278:24003–24010.
6. Park MY, Jang HD, Lee SY, Lee KJ, Kim E (2004) J Biol Chem 279:2544–2549
7. Park MY, Moon JH, Lee KS, Choi HI, Chung J, Hong HJ, Kim E. FAF1 suppresses IkappaB kinase (IKK) activation by disrupting the IKK complex assembly. J Biol Chem. 2007; 282:27572–27577. [PubMed: 17684021]
8. Silverman N, Maniatis T. NF-kB signaling pathways in mammalian and insect innate immunity. 2001. Genes Dev. 2001 15: 2321-2342
9. Silverman N, Kaneko T. Bacterial recognition and signalling by the Drosophila IMD pathway. Cel Micro. 2005. 7(4):461-469
10. Boutros M, Agaisse H, Perrimon N: Sequential activation of signaling pathways during innate immune responses in Drosophila. Dev Cell 2002, 3:711-722.
11. Lemaitre B, Kromermetzger E, Michaut L, Nicolas E, Meister M, Georgel P, Reichhart JM, Hoffmann JA: A recessive mutation, immume-deficiency (IMD), defines 2 distinct control pathways in the Drosophila hostdefense. Proc Natl Acad Sci USA 1995, 92:9465-9469.
12. Gerardo NM, et al. 2010 Feb. Immunity and other defenses in pea aphids, *Acyrthosiphon pisum.* Genome Biology [Internet]. [cited 2016 May 17];1-17. PMID: 20178569
13. Shibuya H., Yamaguchi, K., Shirakabe, K., Tonegawa, A., Gotoh, Y., Ueno, N., Irie, K., Nishida, E., and Matsumoto, K. 1996. TAB1: An activator of the TAK1 MAPKKK in TGF-signal transduction. *Science* **272**: 1179–1182.

# Supplementary Note 15: JAK/STAT pathway

**Introduction**

The Janus kinase/signal transducer of activators of transcription (JAK/STAT) pathway is a somewhat-simple, signaling pathway that provides direct communication between the membrane and nucleus (O'shea *et al.*, 2015). A variety of molecules could signal this pathway, two being cytokines and growth factors (O'shea *et al.*, 2015). Janus kinase and signal transducer of activators of transcription are the mammalian names given for these genes. They have been called hopscotch and marelle or STAT92E in insects, specifically *Drosophila melanogaster* (Hou *et al., 1996).* Domeless is the third component of this pathway, and it is similar to the cytokine receptor in mammals (Brown *et al.*, 2001). They still carry out, generally, the same purposes like development and immunity. In the genome of *Diaphorina citri* a search for orthologs of these genes resulted in finding one of each, establishing the presence of the pathway in the Asian citrus psyllid.

**Materials and Methods**

To find JAK/HOP, STAT, and DOME in the psyllid’s genome orthologs from five organisms were obtained: *Acyrthosiphon pisum* (pea aphid), *Aedes aegypti, Anopheles gambiae, Drosophila melanogaster* (fruit fly), and *Culex pipiens*. The sequences were used in the BLAT tool in Web Apollo, the BLAST tool at I5K, and the MCOT tool in the psyllid database. GNOMON predicted models were manually annotated using supporting evidence tracks. The sequences of the models were used to perform comparisons in Blastp at NCBI.

The *D. citri* sequences were used with sequences from other insects collected from NCBI to make a multiple sequence alignment using default MUSCLE settings in MEGA7. A Neighbor- Joining tree was then made using a 1000 bootstrap test, complete deletion, and Poisson model.

Table 1: Blastp top results for DOME, HOP, and STAT genes in *D. citri.* Includes Name, accession, query, identity, and bit score.

| **Top Blastp Hit for JAK/STAT pathway members in *D. citri*** | | | | | |
| --- | --- | --- | --- | --- | --- |
| **Gene** | **Match** | **Accession** | **Query** | **ID** | **Bit score** |
| DOME | Cytokine receptor [Zootermopsis nevadensis] | KDR09708.1 | 82% | 37% | 1120 |
| HOP | PREDICTED: LOW QUALITY PROTEIN: tyrosine-protein kinase hopscotch-like [Atta cephalotes] | XP_012054793.1 | 83% | 33% | 922 |
| STAT | PREDICTED: signal transducer and activator of transcription 5B isoform X3 [Megachile rotundata] | XP_012146903.1 | 97% | 46% | 1053 |

Table 2: Gene numbers of the JAK/STAT pathway members present in the listed insects, including *D. citri* (Viljakainen, 2015)*.*

| **JAK/STAT member count in *D. citri* vs. other insects** | | | |
| --- | --- | --- | --- |
|  | DOME | HOP | STAT |
| *Drosophila melanogaster* | 1 | 1 | 1 |
| *Anopheles gambiae* | 1 | 1 | 2 |
| *Acyrthosiphon pisum* | 4 | 1 | 2 |
| *Bombyx mori* | 1 | 1 | 1 |
| *Pediculus humanus* | 1 | 1 | 1 |
| *Tribolium castaneum* | 1 | 1 | 1 |


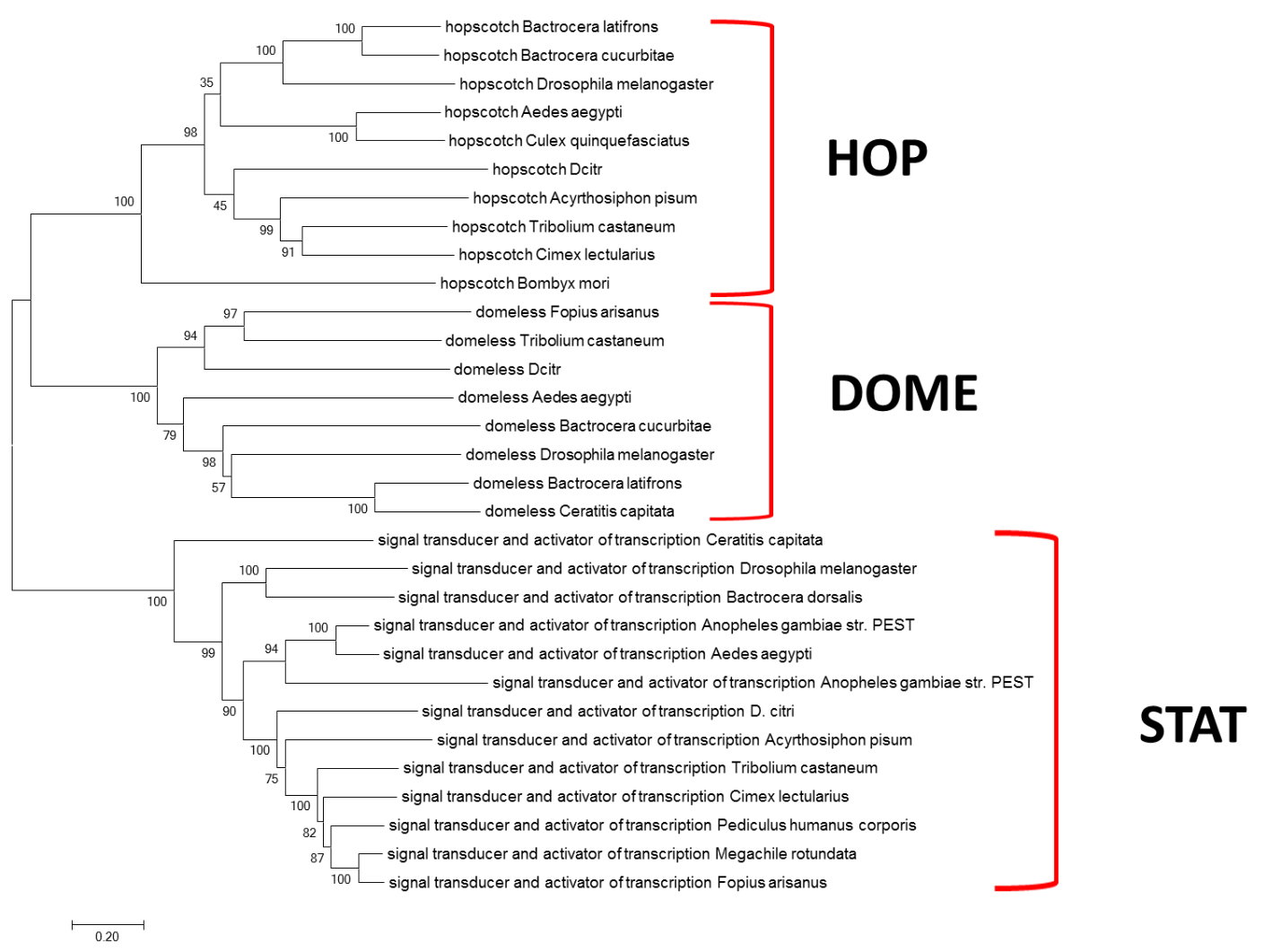


Figure1: Neighbor- Joining tree for JAK/STAT genes in *D. citri* using 1000 bootstrap replicates, Poisson model, and complete deletion. *D. citri* consistently appears close to fellow hemipteran *A. pisum* and beetle *T. castaneum*

**Results and Discussion**

Annotation revealed one ortholog for each component of the JAK/STAT signaling pathway. Blastp gives insect matches with high query and bit score values (Table 2). The gene numbers found in the psyllid is consistent with what has been seen in other insects (Table 2). The phylogenetic tree shows clades formed for each gene, along the psyllid gene being close to pea aphid and *T. castaneum*. The JAK/STAT pathway members have been successfully found and annotated. The next step would be to use them as targets in treatments.

**References**

1. Brown S, Hu N, Hombrı́a JC-G. 2001. Identification of the first invertebrate interleukin JAK/STAT receptor, the Drosophila gene domeless. Current Biology 11(21):1700-1705.Hoffmann JA. 2003. The immune response of Drosophila. Nature 426(6962):33-38.
2. Hou XS, Melnick MB, Perrimon N. 1996. Marelle acts downstream of the Drosophila HOP/JAK kinase and encodes a protein similar to the mammalian STATs. Cell 84(3):411-419.
3. O'Shea JJ, Schwartz DM, Villarino AV, Gadina M, McInnes IB, Laurence A. 2015. The JAK-STAT pathway: impact on human disease and therapeutic intervention*. Annual review of medicine 66:311-328.
4. Viljakainen L. 2015. Evolutionary genetics of insect innate immunity. Briefings in functional genomics:elv002.

# Supplementary Note 16: Lysozymes

**Introduction**

Lysozymes (LYS) hydrolyze the β-(1,4)-glycosidic linkage between *N*-acetylmuramic acid and *N*-acetylglucosamine of bacterial peptidoglycan. Many insects will produce lysozymes, particularly c-type, and secrete them into the hemolymph, following bacterial infection. LYS c-type have been reported in many different insect orders, such as Diptera, Hemiptera, and Lepidoptera (Callewaert and Michiels, 2010). Gram-positive bacteria are detected by these LYS, while only a few insect LYS are antibacterial against Gram-negative bacteria (Abraham et al. 1995; Yu et al. 2002). LYS i-type has isopeptidase (destabilase) activity, it hydrolyzes fibrin molecules in coagulated blood (Callewaert and Michiels, 2010). Studies analyzing whether LYS i-type have muramidase and/or isopeptidase activity in insects are negligible (Paskewitz et al. 2008). Although, *Anopheles gambiae* have shown increased overexpression of i-type LYS in the midgut after blood-feeding, indicating a possible isopeptidase activity (Paskewitz et al. 2008).

**Methods**

Lysozyme orthologs were collected from Immunodb and were used to blat against the *Diaphorina citri*, Asian citrus psyllid (ACP), genome. Identification of LYS was performed in WebApollo where RNA sequence data (adult, nymph, and egg) supported the manually curated gene models. BLASTp was used to confirm accuracy of the annotated LYS. Further analysis of LYS were done through the MCOT database found on citrusgreening.org. Lysozyme orthologs were collected through an NCBI search for the phylogenetic analysis. Multiple sequence alignments of ACP and orthologous insect LYS protein sequences was performed in MEGA6 using ClustalW (Tamura et al 2003). The phylogenetic analysis of LYS included, constructing a midpoint rooted neighbor-joining tree in MEGA6 using p-distance to show evolutionary distance and 1,000 bootstrap replicates.

Table 1: All the LYS found in the *D. citri* genome. Table shows all LYS with names based on NCBI Gnomon with BLASTp matches, corresponding protein length, and domains.

| ***D citri* LYS** | **BLASTP match**  **(organism, bit score)** | **Protein Length** | **Domains** |
| --- | --- | --- | --- |
| **LYS i-type 1** | i-type LYS  (Nilaparvata lugens, 137) | 165 | Destabilase, Destabilase superfamily |
| **LYS i-type 2** | i-type LYS 3  (Nilaparvata lugens, 100) | 131 | Destabilase, Destabilase superfamily |
| **LYS i-type 3** | LYS i-type  (Acyrthosiphon pisum, 169) | 159 | Destabilase, Destabilase superfamily |
| **LYS c-type 1** | Lysozyme  (Tribolium castaneum, 167) | 349 | Lys catalytic site, lys catalytic cleft, Ca^2+^ binding site (2), lysozyme like superfamily, LYZ1, Lys |
| **LYS c-type 2** | Lysozyme  (Papilio polytes, 92.8) | 181 | Lys catalytic site, Ca^2+^ binding site, LYZ1, Lys, lysozyme like superfamily |

Table 2: Number of LYS in *D. citri* and related organisms.

| Organism | LYS c-type | LYS i-type |
| --- | --- | --- |
| *Diaphorina citri* | 2 | 3 |
| *Acyrthosiphon pisum* | 0 | 3 |
| *Cimex lectularius* | 8 | 2 |
| *D. melanogaster* | 12 | 9 |
| *Aedes aegypti* | 6 | 2 |
| *Anopheles gambiae* | 7 | 2 |
| *Culex quinquefasciatus* | 5 | 2 |

**
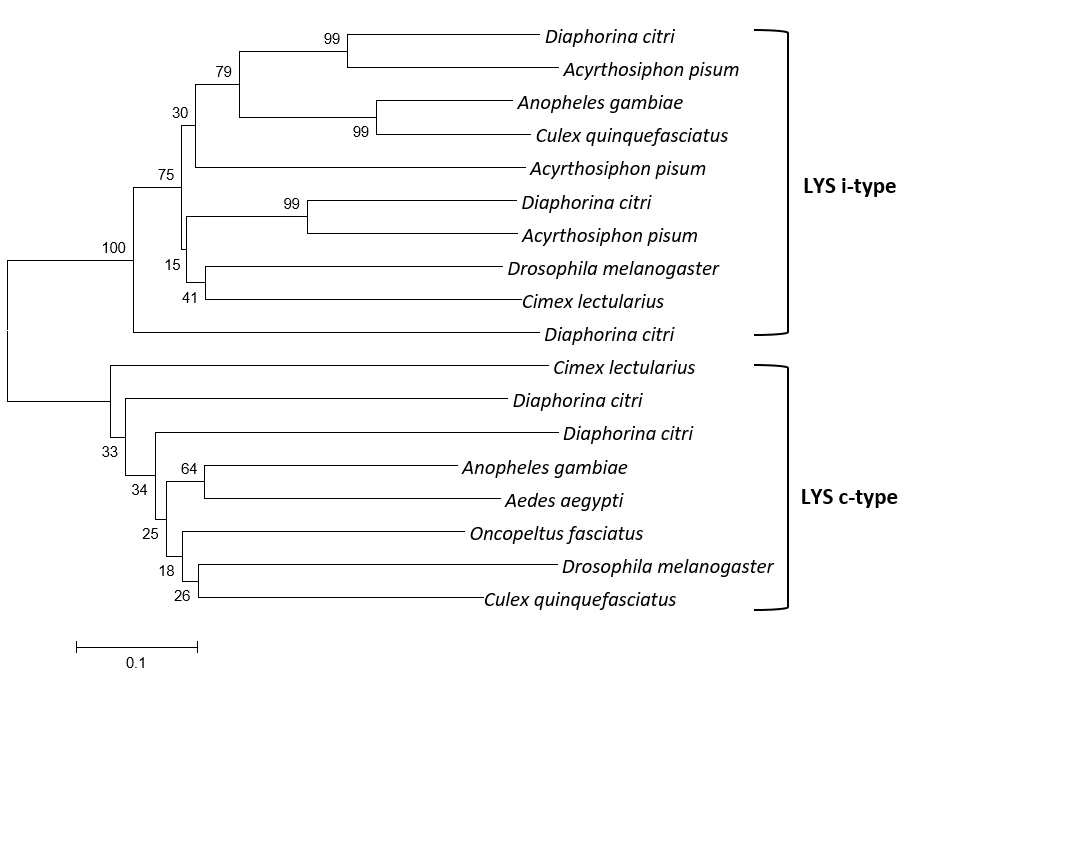
**

Figure 1: Neighbor-joining tree of 18 LYS was constructed in MEGA6 using 1000 bootstrap replications. The p-distance method was used to show evolutionary distance. A total of 75 amino acid positions were analyzed for the tree.

**Results and Discussion**

Within the ACP genome, three LYS i-type and two c-type have been identified. LYS c-type was not found initially with a blat search. The MCOT database predicted two c-type genes and was then found on Webapollo. All LYS i-type found contains the destabilase domain, as shown in Table 1. The function of this destabilase activity is not clear in many insects (Callewaert and Michiels, 2010), therefore it can be inferred no known function in the ACP. *Acyrthosiphon pisum* (aphid)*,* ACP closest relative, lacks the c-type lysozyme but contains three LYS i-type (Table 2) (Gerardo et al., 2010). Bacterioltyic activity assay were performed on immune-challenged aphids suggesting that aphid hemolymph has little to no lysozyme activity (Altincicek et al., 2008).

The neighbor-joining tree of the LYS reveal the divergence of i-type and c-type (Figure 1). It is shown that *D. citri* LYS are evolutionary closer to other hemipterans than dipterans.

**References:**

1. Yu K H, Kim K N, Lee J H, Lee H S, Kim S H, Cho K Y, Nam M H and Lee I H. 2002 Comparative study on characteristics of lysozymes from the hemolymph of three lepidopteran larvae, Galleria mellonella, Bombyx mori, Agrius convolvuli; Dev. Comp. Immunol. 26 707–713
2. Abraham E G, Nagaraju J, Salunke D, Gupta H M and Datta R K. 1995 Purifi cation and partial characterization of an induced antibacterial protein in the silkworm, Bombyx mori; J. Invertebr. Pathol. 65 17–24
3. Callewaert L, and Michiels C W 2010 Lysozymes in the animal kingdom; J. Biosci. 35 127–160] DOI 10.1007/s12038-010-0015-5
4. Paskewitz S M, Li B and Kajla K M 2008 Cloning and molecular characterization of two invertebrate-type lysozymes from Anopheles gambiae; Insect Mol. Biol. 17 217–225
5. Gerardo NM, et al. 2010 Feb. Immunity and other defenses in pea aphids, *Acyrthosiphon pisum.* Genome Biology. 1-17. PMID: 20178569
6. Altincicek B, Gross J, Vilcinskas A: Wounding-mediated gene expression and accelerated viviparous reproduction of the pea aphid Acyrthosiphon pisum. Insect Mol Biol 2008, 17:711-716.

# Supplementary Note 17: Superoxide dismutases

**Introduction**

Superoxide dismutases (SODs) combat reactive oxygen species (ROS) by converting negatively charged oxygen molecules to hydrogen peroxide that is then destroyed by catalase, as an enzymatic defense in aerobic metabolism (Parker et al., 2004). ROS are harmful to cellular components as evidence implicates ROS as a major cause of aging (Perez-Campo et al., 1998; Finkel & Holbrook, 2000; Arking et al., 2002; Barja, 2002; Sohal et al., 2002; Hasty et al., 2003; Hekimi & Guarente, 2003).

In animals, there are three distinct SODs: CuZn SOD (SOD1), CuZn SOD (SOD3), and Mn SOD (SOD2) (Bordo et al., 1994; Colinet et al., 2011). SOD1 is located primarily in the cytoplasm and found in small amounts in the mitochondrial inter-membrane space (Okado and Fridovich, 2001). SOD3 is similar to SOD1 in structure but contains an N-terminal signal peptide and a C-terminal region involved in extracellular binding (Stenlund et al., 2002). SOD2 is completely different from SOD1 in protein sequence and structure. SOD2 is located in the inner matrix of the mitochondria (Weisiger and Fridovich, 1973).

**Methods**

Insecta SOD orthologs were collected from Immunodb and were used to Blat against the *Diaphorina citri*, Asian citrus psyllid (ACP), genome. Identification of the SODs was performed in WebApollo where RNA sequence data (adult, nymph, and egg) supported the manually curated gene models. BLASTp was used to confirm accuracy of the annotated SODs.

Superoxide dismutase orthologs were collected through an NCBI search for the phylogenetic analysis. Multiple sequence alignments of ACP and orthologous insect SODs protein sequences was performed in MEGA6 using ClustalW (Tamura et al 2003). The phylogenetic analysis of SODs included, constructing a midpoint rooted neighbor-joining tree in MEGA6 using p-distance to show evolutionary distance and 1,000 bootstrap replicates.

Table 1: All the SODs found in the *D. citri* genome. Table shows all SODs with names based on NCBI Gnomon with BLASTp matches, corresponding protein length, and domains.

| ***D. citri* SODs** | **BLASTP match**  **(organism, bit score)** | **Protein Length** | **Domains** |
| --- | --- | --- | --- |
| **SOD [Mn-Fe]** | Mn superoxide dismutase  (*Bemisia tabaci*, 296) | 224 | Sod_Fe_N, Sod_Fe_N superfamily, SodA, PLN02471, Sod_Fe_C, Sod_Fe_C superfamily |
| **SOD [Cu-Zn]** | Cu/Zn superoxide dismutase  (*Coptotermes formosanus*, 239) | 155 | Active site, Cu^2+^ binding site, Zn^2+^ binding site, E-class dimer interface, P-class dimer interface, Cu-Zn_Superoxide_Dismutase, Sod_Cu, SodC, Cu-Zn_Superoxide_Dismutase superfamily, PLN02386, PLN02957 |
| **SOD [Cu-Zn]** | superoxide dismutase [Cu-Zn]  (*Acyrthosiphon pisum,* 178) | 214 | Active site, Zn^2+^ binding site, P-class dimer interface, Cu-Zn_Superoxide_Dismutase, Sod_Cu, SodC, Cu-Zn_Superoxide_Dismutase superfamily, PLN02957 |
| **SOD [Cu-Zn], partial** | Superoxide dismutase [Cu-Zn]  (*Acyrthosiphon pisum*, 87.8) | 69 | Cu-Zn_Superoxide_Dismutase, Sod_Cu, SodC, Cu-Zn_Superoxide_Dismutase superfamily, PLN02386, PLN02957 |

Table 2: Number of SODs in *D. citri* and related organisms.

| Organism | # of SODs |
| --- | --- |
| *Diaphorina citri* | 3 |
| *Acyrthosiphon pisum* | 3 |
| *Cimex lectularius* | 5 |
| *D. melanogaster* | 8 |
| *Aedes aegypti* | 7 |
| *Anopheles gambiae* | 6 |
| *Culex quinquefasciatus* | 11 |

*Numbers based on Ensembl and NCBI, does not include isoforms/partial.


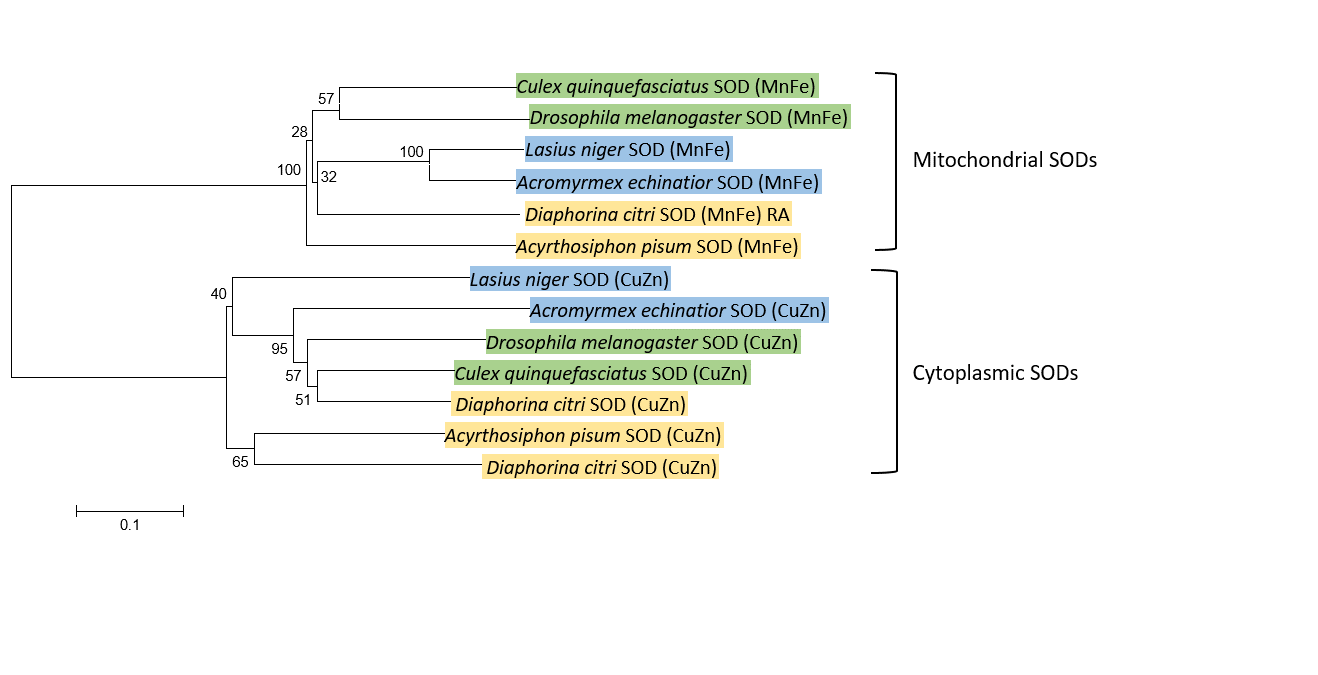
Figure 1: Neighbor-joining tree of 13 CuZn and MnFe SODs was constructed in MEGA6 using 1000 bootstrap replications. The p-distance method was used to show evolutionary distance. A total of 132 amino acid positions were analyzed for the tree. Organisms highlighted in green, blue, and yellow, belong to orders: Diptera, Hymenoptera, and Hemiptera, respectively. NCBI accessions: *D. melanogaster* [NP_001286503.1 & NP_001261700.1], *C. quinquefasciatus* [EDS43182.1 & EDS44530.1], *A. echinatior* [EGI68366.1 & EGI70961.1], *L. niger* [KMQ93269.1 & KMQ95190.1], *A. pisum* [BAH70786.1 & NP_001156153.1].

**Results and Discussion**

It was identified that *D. citri* has a total of four SODs (Table 1). Three of them represent SOD CuZn, and one SOD2. It was shown in *Drosophila melanogaster* that both Cu/Zn SOD and Mn SOD were found to increase life span without effecting metabolism, when induced (Sune et al. 2002). However, transgenic fruit flies with an extra copy of Mn SOD did not exhibit the same effect (Mockett et al. 1999). These results suggest that overexpression/silencing of SOD can increase/decrease life span in other related organisms, including the ACP.

Insect mitochondrial and cytoplasmic SODs are clearly distinct from each other as shown in the phylogenetic tree (Figure 1). These SODs share the same function, but there are many reasons to argue against a common ancestor. SOD1 and SOD2 have completely different crystal structures, differ in metal cofactors, and distinctive catalytic mechanism (Zelko et al., 2002).

**References**

1. Parker JD, Parker KM, Keller L. 2004. Molecular phylogenetic evidence for an extracellular Cu Zn superoxide dismutase gene in insects. Insect Molecular Biology [Internet]. 13(6);587-594.
2. Perez-Campo, R., López-Torres, M., Cadenas, E., Rojas, C. and Barja, G. (1998) The rate of free radical production as a determinant of the rate of aging: evidence from the comparative approach. J Comp Physiol B 168: 149–158.
3. Finkel, T. and Holbrook, N.J. (2000) Oxidants, oxidative stress and the biology of ageing. Nature 408: 239–247.
4. Arking, R., Buck, S., Hwangbo, D. and Lane, M. (2002) Metabolic alterations and shifts in energy allocations are corequisites for the expression of extended longevity genes in Drosophila. Ann NY Acad Sci 959: 251–262
5. Barja, G. (2002) Endogenous oxidative stress: relationship to aging, longevity and caloric restriction. Ageing Res Rev 1: 397–411.
6. Sohal, R.S., Mockett, R.J. and Orr, W.C. (2002) Mechanisms of aging: An appraisal of the oxidative stress hypothesis. Free Radic Biol Med 33: 575–586.
7. Hasty, P., Campisi, J., Hoeijmakers, J., van Steeg, H. and Vijg, J. (2003) Aging and genome maintenance: lessons from the mouse. Science 299: 1355–1359.
8. Hekimi, S. and Guarente, L. (2003) Genetics and the specificity of the aging process. Science 299: 1351–1354.
9. Bordo, D., Djinovic, K. and Bolognesi, M. (1994) Conserved patterns in the Cu, Zn superoxide dismutase family. J Mol Biol 238: 366–386.
10. Colinet D, Cazes D, Belghazi M, Gatti JL, Poirie M. 2011. Extracellular Superoxide Dismutase in Insects CHARACTERIZATION, FUNCTION, AND INTERSPECIFIC VARIATION IN PARASITOID WASP VENOM. The Journal of Biological Chemistry [Internet]. 286(46);40110-40121.
11. Okado-Matsumoto, A., and Fridovich, I. (2001) J. Biol. Chem. 276, 38388–38393
12. Stenlund, P., Lindberg, M. J., and Tibell, L. A. (2002) Biochemistry 41, 3168–3175
13. Weisiger, R. A., and Fridovich, I. (1973) J. Biol. Chem. 248, 4793–4796
14. Mockett R. J., Orr W. C., Rahmandar J. J., Benes J. J., Radyuk S. N., et al., 1999 Overexpression of Mn-containing superoxide dismutase in transgenic Drosophila melanogaster. Arch. Biochem. Biophys. 371: 260–269.
15. Sun J, Folk D, Bradley TJ, Tower J. 2002. Induced overexpression of Mitochondrial Mn-Superoxide Dismutase Extends the Life Span of Adult Drosophila melanogaster. Genetics. 161(2):661-672.
16. Zelko IN, Mariani TJ, Folz RJ. 2002. SUPEROXIDE DISMUTASE MULTIGENE FAMILY: A COMPARISON OF THE CuZn-SOD (SOD1), Mn-SOD (SOD2), AND EC-SOD (SOD3) GENE STRUCTURES, EVOLUTION, AND EXPRESSION. Free Radical Biology & Medicine. 33(3):337-349.

# Supplementary Note 18: CLIP

**Introduction**

Exclusive to the hemolymph of arthropods, Clip-domain serine proteases are “paper clip”-like non-digestive proteases that are composed of one or more N-terminal clip domains (Jang IH, Nam HJ, and Lee WJ 2008). Clip-domains are followed by a linker sequence and a carboxyl-terminal s1A family serine protease domain (Jiang H and Kanost MR 2000). CLIP protease genes have evolved as four clades (CLIPA, CLIPB, CLIPC, and CLIPD) that are present as multigene families in insect genomes and function as hemolymphs in innate immune responses (Jiang H and Kanost MR 2015). In immune pathways that have been characterized, CLIP proteases are involved via a proteinase cascade method (Whaley and Lemercier, 1993). CLIPC is activated by stimulation of an initiating modular serine protease by microbial surface molecules. CLIPC then activates CLIPB which, in turn, cleaves and activates effector molecules proSpätzle or prophenoloxidase. As CLIPA proteins lack proteolytic activity (pseudoprotease), some CLIPA proteins can function as regulators of the other CLIP proteases, forming immune complexes with high molecular weight. The functions of CLIPD and most other CLIP proteases are currently unknown.

**Methods**

The orthologs of CLIPs were collected from NCBI and their scaffolds were screened for predicted gene models using BLAT from Web Apollo and i5k BLAST, where necessary. Gene models were manually annotated and verified using RNAseq data. Further analysis was also performed using NCBI’s BLASTP program.

Once the genes models had been annotated, pairwise alignments between amino acid sequences *Diaphorina citri* and *Acyrthosiphon pisum* were performed using the ClustalW program in MEGA6 software (Tamura, Stecher, Peterson, Filipski, and Kumar 2013).

**Table 1.** Current CLIP domain proteases found in *D.citri* genome (CLIPD annotation is incomplete). Table shows names based on NCBI Gnomon (*) with Blastp matchs, location and accession number, and protein length

| ***D. citri* CLIP Serine Protease** | **LOCATION [Accession #]** | **Protein Length** | **Domains** |
| --- | --- | --- | --- |
| **CLIPA7-trypsin 1 like protease** | gi\|645506705\|ref\|NW_007377811.1  [XP_008470938.1] | 368 bp | **Tryp_SpC** |
| **CLIPA10 -Kallikrein-15-like** | gi\|645505574\|ref\|NW_007378085.1  [XP_008473780.1] | 143 bp |  |
| **CLIPA6-serine proteinase stubble** | gi\|645499856\|ref\|NW_007379449.1  [XP_017303128.1] | 667 bp |  |
| **ClIPA15-serine proteinase stubble** | gi\|645507848\|ref\|NW_007377568.1  [XP_008487216.1] | 690 bp | **Tryp_SpC** |
| **CLIPB1** | gi\|645502691\|ref\|NW_007378736.1  [XP_017302078.1] | 514 bp |  |
| **CLIPB2** | gi\|645506135\|ref\|NW_007377957.1  [XP_008472575.1] | 310 bp |  |
| **CLIPB3**  **(Partial)** | [XP_017302079.1] | - |  |
| **CLIPB4**  **(Partial)** | - | - |  |
| **CLIPB5** | gi\|645508403\|ref\|NW_007377443.1  [XP_017303373.1] | 254 bp |  |
| **CLIPB6** | gi\|645507848\|ref\|NW_007377568.1  [XP_008488270.1] | 485 bp |  |
| **CLIPB7** | gi\|645506135\|ref\|NW_007377957.1  [XP_017299783.1] | 496 bp |  |
| **CLIPC11-serine protease snake-like** | gi\|645507171\|ref\|NW_007377706.1  [XP_008469523.1] | 384 bp |  |
| **CLIPC3 -Serine protease snake-like** | gi\|645504563\|ref\|NW_007378328.1  [XP_008475910.1] | 184 bp |  |
| **CLIPD- serine protease (Partial)** | - | - |  |

**Table 2**. Represents Query Coverage (Identity) and E-value of the annotated gene models pairwise aligned to orthologues in other species. Pairwise alignment was performed using NCBI blast.

|  | **CLIPA6**  **QC (ID)  E-value** | **CLIPA7**  **QC (ID)  E-value** | **CLIPA10**  **QC (ID)  E-value** | **CLIPA15**  **QC (ID)  E-value** | **CLIPC3**  **QC (ID)  E-value** | **CLIPC11**  **QC (ID)  E-value** |
| --- | --- | --- | --- | --- | --- | --- |
| ***Acyrthosiphon pisum*** | 34% (37%)  1e-43 | 65% (46%)  1e-66 | 36% (60%)  2e-19e | 96% (58%)  0.0 | 86% (47%)  2e-39 | 95% (44%)  1e-60 |
| ***Halyomorpha halys*** | 45% (68%)  6e-140 | 66% (57%)  4e-82 | 37% (68%)  3e-24 | 36% (37%)  0.0 | 80% (43%)  2e-35 | 90% (37%)  1e-47 |
| ***Cimex lectularius*** | 32% (33%)  9e-32 | 66% (61%)  1e-93 | 35% (59%)  1e-18 | 35% (39%)  0.0 | 80% (40%)  4e-33 | 100%(39%)  8e-53 |
| ***Anopheles gambiae*** | 31% (40%)  4e-50 | 59% (59%)  2e-99 | 79% (78%)  1e-26 | 86% (64%)  5e-169 | 97% (42%)  2e-39 | 90% (34%)  9e-54 |
| ***Aedes aegypti*** | 95% (47%)  9e-166 | 66% (58%)  1e-94 | 37% (79%)  2e-27 | 93%(59%)  0.0 | 80% (43%)  1e-35 | 96% (40%)  1e-54 |

| ***Diaphorina citri*** | **CLIPB1**  **QC (ID)  E-value** | **CLIPB2**  **QC (ID)  E-value** | **CLIPB5**  **QC (ID)  E-value** | **CLIPB6**  **QC (ID)  E-value** | **CLIPB7**  **QC (ID)  E-value** |
| --- | --- | --- | --- | --- | --- |
| ***Acyrthosiphon pisum*** | 81%(36%)  5e46 | 98%(57%)  2e-124 | 100%(45%)  6e-78 | 90%(56%)  0.0 | 91%(61%)  2e-67 |
| ***Halyomorpha halys*** | 86%(37%)  1e-60 | 87%(60%)  7e-131 | 99%(47%)  4e-69 | 90%(53%)  5e-163 | 95%(57%)  2e-80 |
| ***Cimex lectularius*** | 82%(37%)  3e-46 | 85%(64%)  6e-139 | 99%(43%)  2e-70 | 89%(57%)  1e-180 | 84%(73%)  8e-80 |
| ***Anopheles gambiae*** | 82%(41%)  2e-61 | 85%(66%)  6e-140 | 100%(38%)  1e-58 | 89%(48%)  3e-117 | 77%(75%)  2e-69 |
| ***Aedes aegypti*** | 81%(40%)  7e-55 | 80%(68%)  9e-137 | 100%(41%)  1e-65 | 69%(49%)  3e-122 | 77%(73%)  7e-66 |

**Table 3**. This table shows the number of CLIP domain proteases within *D.citri, A. pisum, H. halys, C. Lectularius, A. gambiae* and *A. aegypti*.

|  | **Number of CLIP Genes** |
| --- | --- |
| ***Diaphorina citri*** | 11 found |
| ***Acyrthosiphon pisum*** | 11 |
| ***Halyomorpha halys*** | 18 |
| ***Cimex lectularius*** | 7 |
| ***Anopheles gambiae*** | 41 |
| ***Aedes aegypti*** | 79 |

**Figure 1.** Neighbor joining tree of 11 annotated CLIP domain proteases was constructed using standard parameters.


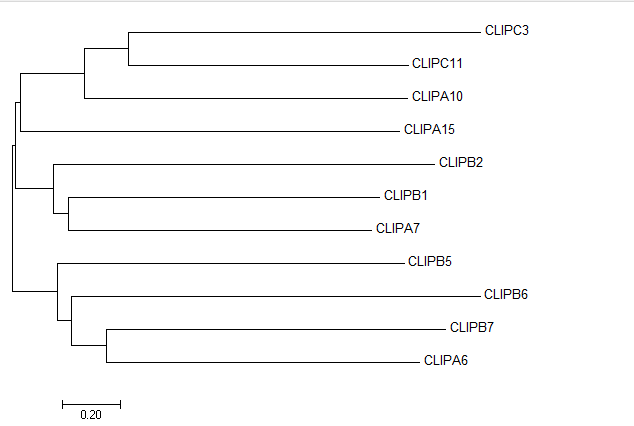


**Figure 2.** Neighbor joining tree constructed using standard parameters. Eleven annotated CLIP domain proteases phylogenetic relationships were compared within various insect species. 4 Tribolium (Tc), 4 Drosophila (Dm), 3 Manduca (Ms), 7 Anopeles (Ag), Aedes (Aa), and 5 Acrythosiphon (Ap).


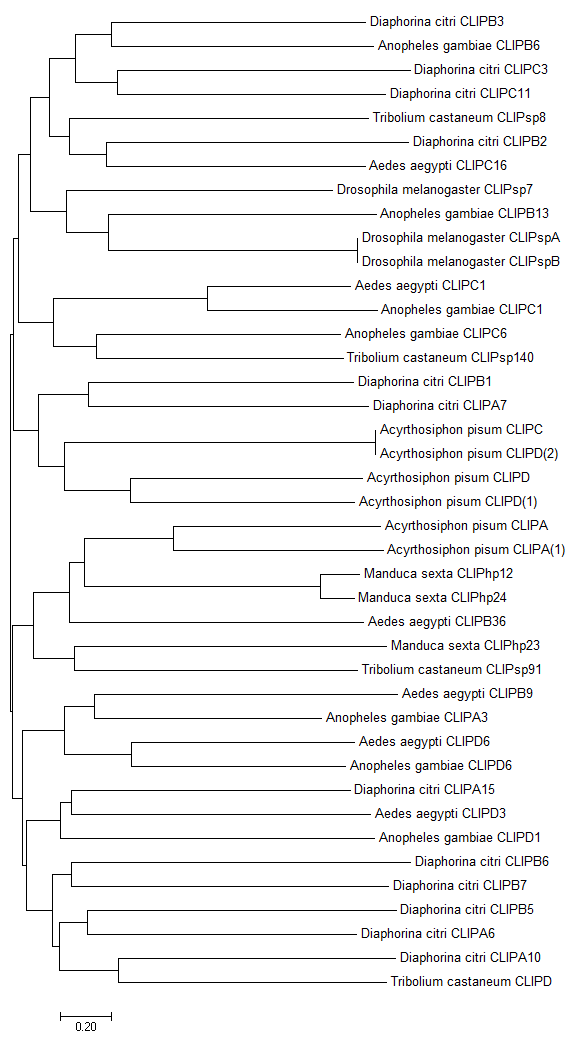


**Table 3. MCOT blasts of annotated CLIP sequences.** Sequences were blasted against the *Diaphorina citri* MCOT protein database using blastp.

|  | **SubjectId** | **id%** | **Aln** | **evalue** | **Score** |
| --- | --- | --- | --- | --- | --- |
| **CLIPA7** | [MCOT03907.0.CO](https://citrusgreening.org/tools/blast/show_match_seq.pl?blast_db_id=26;id=MCOT03907.0.CO;hilite_coords=223-440) | 99.54 | 217/218 | e-155 | 448 |
| **CLIPA10** | [MCOT02226.0.CT](https://citrusgreening.org/tools/blast/show_match_seq.pl?blast_db_id=26;id=MCOT02226.0.CT;hilite_coords=651-738) | 100 | 88/88 | 3.00E-51 | 177 |
| **CLIPA6** | [MCOT05567.0.CT](https://citrusgreening.org/tools/blast/show_match_seq.pl?blast_db_id=26;id=MCOT05567.0.CT;hilite_coords=323-961) | 96.06 | 634/660 | 0 | 1149 |
| **CLIPA15** | [MCOT01310.0.CT](https://citrusgreening.org/tools/blast/show_match_seq.pl?blast_db_id=26;id=MCOT01310.0.CT;hilite_coords=229-762) | 72.57 | 471/649 | 0 | 810 |
| **CLIPC11** | [MCOT00897.2.CO](https://citrusgreening.org/tools/blast/show_match_seq.pl?blast_db_id=26;id=MCOT00897.2.CO;hilite_coords=147-389) | 89.67 | 243/271 | e-170 | 484 |
| **CLIPC3** | [MCOT12281.1.CO](https://citrusgreening.org/tools/blast/show_match_seq.pl?blast_db_id=26;id=MCOT12281.1.CO;hilite_coords=12-195) | 94.02 | 173/184 | e-126 | 360 |
| **CLIPB1** | [MCOT23305.0.CC](https://citrusgreening.org/tools/blast/show_match_seq.pl?blast_db_id=26;id=MCOT23305.0.CC;hilite_coords=27-360) | 100 | 334/334 | 0 | 676 |
| **CLIPB2** | [MCOT16598.0.CC](https://citrusgreening.org/tools/blast/show_match_seq.pl?blast_db_id=26;id=MCOT16598.0.CC;hilite_coords=1-310) | 100 | 310/310 | 0 | 643 |
| **CLIPB5** | [MCOT05156.0.CT](https://citrusgreening.org/tools/blast/show_match_seq.pl?blast_db_id=26;id=MCOT05156.0.CT;hilite_coords=146-379) | 92.13 | 234/254 | e-167 | 471 |
| **CLIPB6** | [MCOT19384.1.CT](https://citrusgreening.org/tools/blast/show_match_seq.pl?blast_db_id=26;id=MCOT19384.1.CT;hilite_coords=35-550) | 78.67 | 413/525 | 0 | 799 |
| **CLIPB7** | [MCOT16016.2.CC](https://citrusgreening.org/tools/blast/show_match_seq.pl?blast_db_id=26;id=MCOT16016.2.CC;hilite_coords=1-212) | 99.53 | 211/212 | e-153 | 443 |

**Table 3 Cont.**

|  | **Description** |
| --- | --- |
| **CLIPA7** | \| Serine protease H2 \| Similar to D6WBT0 \| ***- \| PANTHER PTHR24256 \| PANTHER PTHR24256:SF203 \| Pfam PF00089 Length = 440 |
| **CLIPA10** | \| Serine proteinase stubble \| Similar to A0A034V6F0 \| *-*- \| PANTHER PTHR24258 \| PANTHER PTHR24258:SF96 \| Pfam PF00089 |
| **CLIPA6** | \| Serine proteinase stubble \| Similar to A0A034V6F0 \| *-*- \| PANTHER PTHR24258 \| PANTHER PTHR24258:SF96 \| Pfam PF00089 Length = 1010 |
| **CLIPA15** | \| Serine protease 42 \| Similar to W8C5K3 \| *-*- \| PANTHER PTHR24258 \| PANTHER PTHR24258:SF105 \| Pfam PF00089 Length = 762 |
| **CLIPC11** | \| Trypsin-like serine protease \| Similar to Q9XY63 \| ***- \| PANTHER PTHR24256 \| Pfam PF00089 Length = 391 |
| **CLIPC3** | \| Trypsin-like serine protease \| Similar to Q9XY63 \| ***- \| PANTHER PTHR24256 \| Pfam PF00089 Length = 308 |
| **CLIPB1** | \| Serine protease \| Similar to Q6RX66 \| ***- \| PANTHER PTHR24256 \| Pfam PF00089 Length = 360 |
| **CLIPB2** | \| Serine protease \| Similar to W5JJ65 \| ***- \| PANTHER PTHR24256 \| PANTHER PTHR24256:SF218 \| Pfam PF00089 Length = 310 |
| **CLIPB5** | \| Serine protease \| Similar to Q8MP08 \| ***- \| PANTHER PTHR24256 \| Pfam PF00089 \| Pfam PF12032 Length = 379 |
| **CLIPB6** | \| Trypsin-like serine protease \| Similar to A8QL65 \| ***- \| PANTHER PTHR24258 \| PANTHER PTHR24258:SF99 \| Pfam PF00089 Length = 550 |
| **CLIPB7** | \| Trypsin-like serine protease \| Similar to A8QL65 \| ***- \| PANTHER PTHR24256 \| PANTHER PTHR24256:SF158 \| Pfam PF00089 Length = 335 |

**Results and Discussion**

Eleven CLIPs (excluding partial models), from four subfamilies, within the *D. citri* genome were manually annotated using Web Apollo software (Table 1). A neighbor-joining tree was constructed using standard parameters (Fig 1). Phylogenetic relationships of the annotated CLIP domain proteases were compared with various insect species [Tribolium (Tc) XP_015839950.1, XP_008193916.1, EFA07452.2, EFA11600.2, Drosophila (Dm) 2XXL_A, NP_001097020.1, Manduca (Ms) AAZ91695.1, AAZ91694.1, AAZ91697.1, Anopeles (Ag) EAA03300.4, XP_307757.4, CAO83683.1, XP_552698.3, XP_001687772.2, XP_312523.4, XP_312099.2, Aedes (Aa) XP_001657092.1, XP_011492919.1, XP_001655815.2, XP_001662819.1, XP_001662898.2, XP_001654732.1, and Acrythosiphon (Ap) XP_001943192.2, XP_016663806.1, NP_001155379.1, BAH71307.1, XP_008178923.2 ].

A pairwise alignment (Table 2) identified several *A. pisum* CLIPA orthologs (CLIPA6, CLIPA7, CLIPA10, CLIPA15) with 37–60% identity, and 34–65% query coverage for CLIPA6, CLIPA7, and CLIPA10, while a query coverage of 96% was displayed in CLIPA15. Higher identities and query coverages (qc) for CLIPA6, CLIPA7, and CLIPA10 were found in *Aedes aegypti* (47% ident, 95% qc), *Cimex lectularius* (61% ident, 66% qc), and *Anopheles gambiae* (78% ident, 79% qc) respectively. Orthologs of CLIPC (3 and 11) and CLIPB (1, 2, 5, 6, and 7) in *A. pisum* were also found in *D. citri* with 36–61% identity and 81–100% query coverage.

**References**

1. Jang I-H, Nam H-J, Lee W-J. CLIP-domain serine proteases in Drosophila innate immunity. BMB Reports. 2008;41(2):102–107.
2. Jiang H, Kanost MR. The clip-domain family of serine proteinases in arthropods. Insect Biochemistry and Molecular Biology. 2000;30(2):95–105.
3. Kanost MR, Jiang H. Clip-domain serine proteases as immune factors in insect hemolymph. Current
4. Opinion in Insect Science. 2015;11:47–55.
5. Whaley, K., Lemercier, C., 1993. The complement system. In: Sim, E. (Ed.) The Natural Immune System, Humoral Factors. IRL Press, New York, pp. 121–150.
6. Zou Z, et al. Transcriptome analysis of Aedes aegypti transgenic mosquitoes with altered immunity. PLoS Pathog. 2011. 7(11):e1002394.
7. Wang L, et al. Understanding the immune system architecture and transcriptome responses to southern rice black-streaked dwarf virus in Sogatella furcifera. Sci. Rep. 2016 . 36254.
8. Christophides GK, et al. Immunity-related genes and gene families in Anopheles gambiae. Science 2002;298:159–165.

# Supplementary Note 19: Autophagy

**Introduction**

Autophagy is the regulated breakdown of unnecessary or dysfunctional components of the cell. This process is critical and is highly conserved among all animals and is critical to the regulation of cell degradation and recycling of cellular components. The main pathway is marcoautophagy, where specific cytoplasmic components are isolated from the remaining cell in a double-membraned vesical called the autophagosome^1-3^.

**Materials and Methods**

*Drosophila* *melanogaster* autophagy-related genes (Twenty total) were used to identify putative autophagy genes within the ACP genome and MCOT datasets. Blastx (e value = 10^-10^ limit) was used to compared the *Drosophila* autophagy-related genes to ACP predicted and MCOT protein sets. The protein sequence for each corrected model was then subjected to a blastp at NCBI to ensure the correct autophagy gene had been identified.

Table 1. A comparison table between *D.citri* and *D. melanogastor* autophagy genes derived

from the BLAST results.

Table 2. BLAST-based analyses of *D*. *citri* autophagy-associated genes comapred to those from the fruit fly (*Drosophila* *melanogaster*), bed bug (*Cimex* *lectularius*), and pea aphid (*Acyrthosiphon* *pisum*).

**Results and Discussion**

Based upon the blast results, we were able to identify 17/20 autophagy-related genes from the ACP genome and MCOT gene sets (Table 1; Table 2). There is only a single autophagy-related 8 gene in ACP gene sets compared to two for the *Drosophila* gene set, but this is common for non-dipteran insects^2^. Thus, as expected, psyllids have the required repertoire of autophagy-related genes to undergo marcoautophagy. This suggests that utilized of the gene sets from the genome and MCOT can identify conserved genes for ACP by comparison to other insects such as *Drosophila*.

**References**

1. Chang, Y.Y. & Neufeld, T.P. Autophagy takes flight in Drosophila. *FEBS Lett* **584**, 1342-9 (2010).

2. Malagoli, D. *et al.* Autophagy and its physiological relevance in arthropods: Current knowledge and perspectives. *Autophagy* **6**, 575-588 (2010).

3. Zirin, J. & Perrimon, N. Drosophila as a model system to study autophagy. *Semin Immunopathol* **32**, 363-72 (2010).

# Supplementary Note 20: Dicer

**Introduction**

RNase III enzymes are molecules which exhibit specificity for dsRNA. Three subclasses of RNase III enzymes have been identified to date; the *Escherichia coli* RNase III class (class I), the Drosha class (class II) and the Dicer-like class (class III). Typically, enzymes in the Dicer-like class contain an N-terminal DEXH-box RNA helicase domain, a domain of unknown function known as DUF283, a PAZ (PIWI-Argonaute-Zwille) domain, two RNase III domains and a dsRNA binding domain. The presence of these domains allow Dicer (Dcr) to function as a ribonuclease, cleaving long dsRNA molecules into small effector molecules, in both the miRNA and RNAi pathways (reviewed in ([Carmell and Hannon, 2004](#_ENREF_2)). In vertebrates and some invertebrates, such as *Caenorhabditis elegans*, one protein (Dcr-1) is involved in both pathways ([Bernstein et al., 2001](#_ENREF_1); [Ketting et al., 2001](#_ENREF_3); [Knight and Bass, 2001](#_ENREF_4)). In other organisms, such as *Drosophila melanogaster*, different Dcr proteins function in each pathway. In *D. melanogaster* Dm-Dcr-1 functions in the miRNA pathway while Dm-Dcr-2 functions in the RNAi pathway ([Lee et al., 2004](#_ENREF_5)).

**Methods**

Insect Dicer protein sequences were obtained from NCBI, ImmunoDB and the i5k workspace. BLASTp analysis was used to query the predicted *Diaphorina citri* protein sets (Diacit_

International_psyllid_consortium_proteins_v1 and Diacit_RefSeq_proteins_Release_100) at i5k@NAL. Loci encoding putative homologs were identified and manually annotated in Web Apollo using RNA-Seq data as evidence for transcript structure. EMBL EBI’s online InterPro program was used for domain identification. MEGA7 software was used to produce multiple sequence alignments of insect Dicer and RNase III enzyme homologs (ClustalW). Neighbor-joining phylogenetic trees were constructed in MEGA7 using multiple sequence alignments of predicted protein sequences.

Table 1: Number of Dicer homologs in insect species.

| **Species** | **Dicer 1** | **Dicer 2** |
| --- | --- | --- |
| *D. melanogaster* | 1 | 1 |
| *A. gambiae* | 1 | 1 |
| *A. aegypti* | 1 | 1 |
| *C. quinquefasciatus* | 1 | 1 |
| *T. castaneum* | 1 | 1 |
| *C. lectularius* | 1 | 1 |
| *D. citri* | 1 or 2 | 1 or 2 |

Table 2: Bit score, query coverage, identity and e-value results from BLAST analysis of predicted Dicer1, Dicer2RA, Dicer2RB, Dicer like partial and Endonuclease Dicer to insect Dcr homologs. The scores given are the scores from the named ortholog in each insect species. In the cases of Dicer like partial and Endonuclease Dicer whether the hit was to Dicer1 or Dicer2 has been denoted in the Bit Score column.

|  | **Top Blastp Hit** |  | *D. melanogaster* | *A. gambiae* | *A. aegypti* | *C. quinquefasciatus* | *T. castaneum* | *C. lectularius* |
| --- | --- | --- | --- | --- | --- | --- | --- | --- |
| *D. citri* Dicer 1 | Dcr 1 *C. costatus* | **Bit score** | 639 | 688 | 724 | 668 | 639 | 1145 |
|  |  | **QC (Identity)** | 92% (59%) | 90% (35%) | 90% (36%) | 89% (61%) | 79% (65%) | 94% (49%) |
|  |  | **E value** | 0.00E+00 | 0.00E+00 | 0.00E+00 | 0.00E+00 | 0.00E+00 | 0.00E+00 |
| *D. citri* Dicer 2RA | Dicer 2 *N. lugens* | **Bit score** | 94 | 91.3 | 99 | 92.8 | 97.4 | 113 |
|  |  | **QC (Identity)** | 15% (41%) | 16% (39%) | 63% (41%) | 68% (42%) | 42% (41%) | 40% (41%) |
|  |  | **E value** | 1.00E-19 | 5.00E-19 | 2.00E-21 | 2.00E-19 | 1.00E-20 | 8.00E-26 |
| *D. citri* Dicer 2RB | Dicer 2 *N. lugens* | **Bit score** | 94.7 | 91 | 99 | 92.8 | 97.4 | 113 |
|  |  | **QC (Identity)** | 15% (41%) | 16% (39%) | 63% (41%) | 68% (42%) | 42% (41%) | 40% (41%) |
|  |  | **E value** | 1.00E-19 | 5.00E-19 | 2.00E-21 | 2.00E-19 | 1.00E-20 | 8.00E-26 |
| *D. citri* Dicer like partial | Dicer like *N. vespilloides* | **Bit score** | 166 (Dcr1) | 180 (Dcr1) | 187 (Dcr2) | 191 (Dcr2) | 208 (Dcr2) | 191 (Dcr1) |
|  |  | **QC (Identity)** | 45% (39%) | 52% (37%) | 59% (41%) | 48% (42%) | 69% (43%) | 61% (48%) |
|  |  | **E value** | 2.00E-42 | 1.00E-47 | 5.00E-50 | 4.00E-51 | 2.00E-56 | 1.00E-60 |
| *D. citri* Endonuclease Dicer | Endoribonuclease Dicer *C. floridanus* | **Bit score** | 82 (Dcr1) | 84 (Dcr1) | 97.1 (Dcr2) | 87.8 (Dcr2) | 80.9 (Dcr2) | 83.2 (Dcr2) |
|  |  | **QC (Identity)** | 61% (34%) | 61% (34%) | 64% (39%) | 63% (34%) | 55% (39%) | 66% (29%) |
|  |  | **E value** | 1.00E-17 | 8.00E-19 | 2.00E-23 | 4.00E-20 | 2.00E-17 | 2.00E-18 |

**Results and Discussion**

In *D. citri*, initial BLASTp searches with known insect Dcr proteins identified four loci encoding predicted Dcr proteins (Table 1). InterPro analysis of the first Dcr identified (Dicer1) revealed that the predicted protein contained a DEXH box RNA helicase domain, RNase III domains and a PAZ domain validating its identification as a Dcr protein. However, incomplete assembly of the *D. citri* genome has made analysis of the final three *Dcr* loci more difficult. Dicer2, as currently annotated, contains both a helicase and PAZ domain but seems to be lacking the RNase III domain. Whether this represents a true domain loss or is an artifact of inaccurate assembly or incomplete annotation is unknown. RNA-Seq data suggests that at least two isoforms of this gene are produced, they have been designated *Dicer2A* and *Dicer2B*. The third *Dcr* loci identified (*endoribonuclease Dicer*) is a fragment of DNA on a very short contig (~3,000bp) suggesting that this portion of the genome may not be assembled correctly. Analysis of this fragment revealed that this sequence only encodes a PAZ domain. As PAZ domains are only found in Dcr and Argonaute (AGO) proteins ([Carmell and Hannon, 2004](#_ENREF_2)) it is possible that this fragment (*endoribonuclease Dicer*) may in fact belong to one of the previously identified Dcr or AGO proteins but has been incorrectly assembled resulting in a partial gene fragment (although multiple sequence alignments of *D. citri* AGO and Dcr proteins does not support this idea). Alternatively, it could represent a fragment of a duplicated *Dicer* gene as yet unidentified. The final *Dcr* loci identified (*Dicer like partial*) is at the end of a contig and is thus incomplete. Domain analysis of this fragment indicates a helicase domain and RNase III domain are present in the available sequence. Reciprocal blast analysis of the four identified loci all indicate that these proteins share homology with other insect Dcr proteins but determining true orthology for Dicer like partial and Endonuclease Dicer is not possible at this time (Table 2).

Figure 1: Phylogenetic tree of inset Dicer proteins. Dicer 1 is represented in blue Dicer 2 is represented in purple. *D. citri* proteins have been identified with a taxon marker.


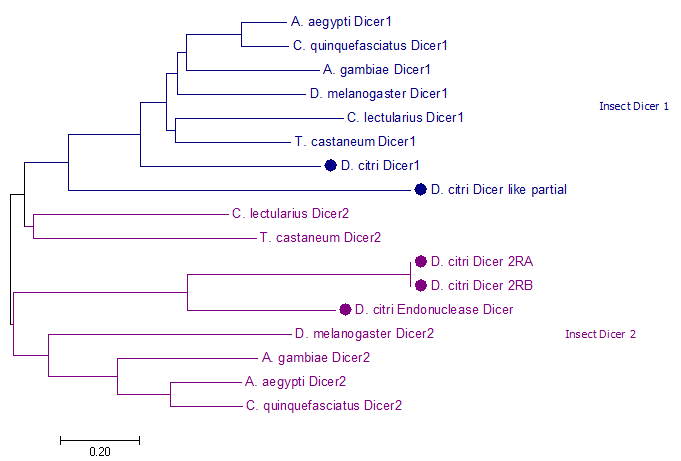


Due to incomplete gene models we are unable to make a definitive determination of *Dcr* gene number in *D. citri* but there are likely three and perhaps four *Dcr* genes in *D. citri* (Table 1). Incomplete sequences may influence phylogenetic tree analysis but based on the data we have Dicer1 is orthologous to other insect Dcr1 proteins (Figures 1 and 2). The isoforms Dicer 2RA and Dicer 2RB, as currently annotated, cluster with other Dicer2 proteins (as does the Endoribonuclease Dicer fragment) (Figure 1). Finally, Dicer like partial shows similarity with both Dcr-1 (Figure 1) and Dcr-2 (Figure 2). More complete gene models or complete sequences from experimental data is necessary to determine the exact relationship between *D. citri* Dcr proteins and other insect Dcrs.


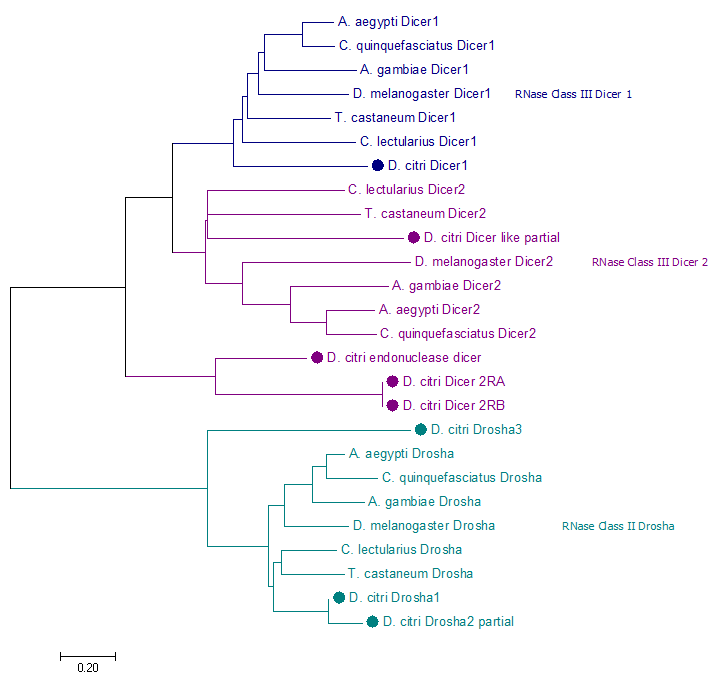


Figure 2: Phylogenetic tree depicting clustering of insect Class II (Drosha) and Class III (Dicer) RNase III enzymes. Blue represents likely Dicer1 homologs. Purple represents likely Dicer2 homologs. Teal represents Drosha homologs. *D. citri* proteins have been marked with a taxon marker.

**References**

1. Bernstein, E., Caudy, A. A., Hammond, S. M. and Hannon, G. J. (2001) 'Role for a bidentate ribonuclease in the initiation step of RNA interference', *Nature* 409(6818): 363-6.
2. Carmell, M. A. and Hannon, G. J. (2004) 'RNase III enzymes and the initiation of gene silencing', *Nat Struct Mol Biol* 11(3): 214-8.
3. Ketting, R. F., Fischer, S. E., Bernstein, E., Sijen, T., Hannon, G. J. and Plasterk, R. H. (2001) 'Dicer functions in RNA interference and in synthesis of small RNA involved in developmental timing in C. elegans', *Genes Dev* 15(20): 2654-9.
4. Knight, S. W. and Bass, B. L. (2001) 'A role for the RNase III enzyme DCR-1 in RNA interference and germ line development in Caenorhabditis elegans', *Science* 293(5538): 2269-71.
5. Lee, Y. S., Nakahara, K., Pham, J. W., Kim, K., He, Z., Sontheimer, E. J. and Carthew, R. W. (2004) 'Distinct roles for Drosophila Dicer-1 and Dicer-2 in the siRNA/miRNA silencing pathways', *Cell* 117(1): 69-81.

# Supplementary Note 21: Drosha

**Introduction**

Drosha belongs to a class (class II) of RNase III enzymes. Drosha proteins characteristically contain two tandem RNase III domains and a C-terminal dsRNA binding domain. One of Drosha’s primary roles in the cell is in the processing of microRNAs (miRNAs). Drosha’s catalytic activity allows it to cleave primary miRNA (pri-miRNA) molecules, which have been transcribed by the cell, into precursor miRNA (pre-miRNA) molecules which are transported from the nucleus via Exportin-5 and then are further processed by Dicer (a class III RNase III enzyme) into their mature form (reviewed in ([Ha and Kim, 2014](#_ENREF_3); [Connerty et al., 2015](#_ENREF_2)).

Drosha is conserved in animals, and most insects with a sequenced genome (and annotated RNAi and miRNA genes) have been shown to have 1 Drosha homolog ([Campbell et al., 2008](#_ENREF_1); [Tomoyasu et al., 2008](#_ENREF_8); [Jaubert-Possamai et al., 2010](#_ENREF_5); [Xu et al., 2013](#_ENREF_9)).

**Methods**

Insect Drosha protein sequences were obtained from NCBI, ImmunoDB and the i5k workspace. BLASTp analysis was used to query the predicted *Diaphorina citri* protein sets (Diacit_

International_psyllid_consortium_proteins_v1 and Diacit_RefSeq_proteins_Release_100) at i5k@NAL. Loci encoding putative homologs were identified and manually annotated in Web Apollo using RNA-Seq data as evidence for transcript structure. EMBL EBI’s online InterPro program was used for domain identification. MEGA7 software was used to produce multiple sequence alignments of insect Drosha and RNase III homologs (ClustalW). Neighbor-joining phylogenetic trees were constructed in MEGA7 using multiple sequence alignments of predicted protein sequences.

Table 1: Number of Drosha homologs in insect species

| **Species** | **Drosha** |
| --- | --- |
| *D. melanogaster* | 1 |
| *A. gambiae* | 1 |
| *A. aegypti* | 1 |
| *C. quinquefasciatus* | 1 |
| *T. castaneum* | 1 |
| *C. lectularius* | 1 |
| *D. citri* | 2 or 3 |

Table 2: Bit score, query coverage, identity and e-value results from BLAST analysis of predicted Drosha1, Drosha2 partial and Drosha3 to insect Drosha homologs.

|  |  | ***D. melanogaster*** | ***A. gambiae*** | ***A. aegypti*** | ***C. quinquefasciatus*** | ***T. castaneum*** | ***C. lectularius*** |
| --- | --- | --- | --- | --- | --- | --- | --- |
| *D. citri* Drosha 1 | **Bit score** | 567 | 588 | 587 | 42 | 522 | 592 |
|  | **QC (Identity)** | 95% (72%) | 96% (73%) | 96% (73%) | 83.2% (16%) | 94% (67%) | 955 (73%) |
|  | **E value** | 0.00E+00 | 0.00E+00 | 0.00E+00 | 4.00E-04 | 2.00E-176 | 0.00E+00 |
| *D. citri* Drosha 2 partial | **Bit score** | 917 | 967 | 974 | 731 | 1060 | 1044 |
|  | **QC (Identity)** | 78% (57%) | 77% (58%) | 81% (57%) | 64% (56%) | 77% (64%) | 81% (62%) |
|  | **E value** | 0.00E+00 | 0.00E+00 | 0.00E+00 | 0.00E+00 | 0.00E+00 | 0.00E+00 |
| *D. citri* Drosha 3 | **Bit score** | 347 | 366 | 370 | 379 | 443 | 443 |
|  | **QC (Identity)** | 61% (45%) | 60% (45%) | 67% (45%) | 51% (48%) | 59% (54%) | 59% (54%) |
|  | **E value** | 2.00E-100 | 3.00E-107 | 7.00E-109 | 2.00E-116 | 1.00E-136 | 1.00E-136 |

Figure 1: Phylogenetic tree of insect Drosha homologs. Insect species are depicted with differing colors. *D. citri* proteins are marked with a taxon marker.


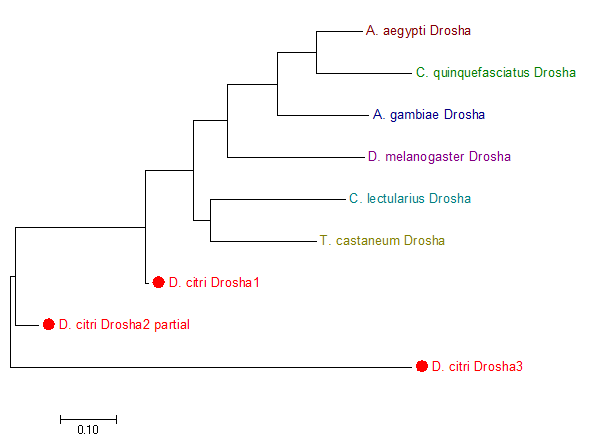


Figure 2: Phylogenetic tree depicting clustering of insect Class II (Drosha) and Class III (Dicer) RNase III enzymes. Blue represents likely Dicer1 homologs. Purple represents likely Dicer2 homologs. Teal represents Drosha homologs. *D. citri* proteins have been marked by a taxon marker.


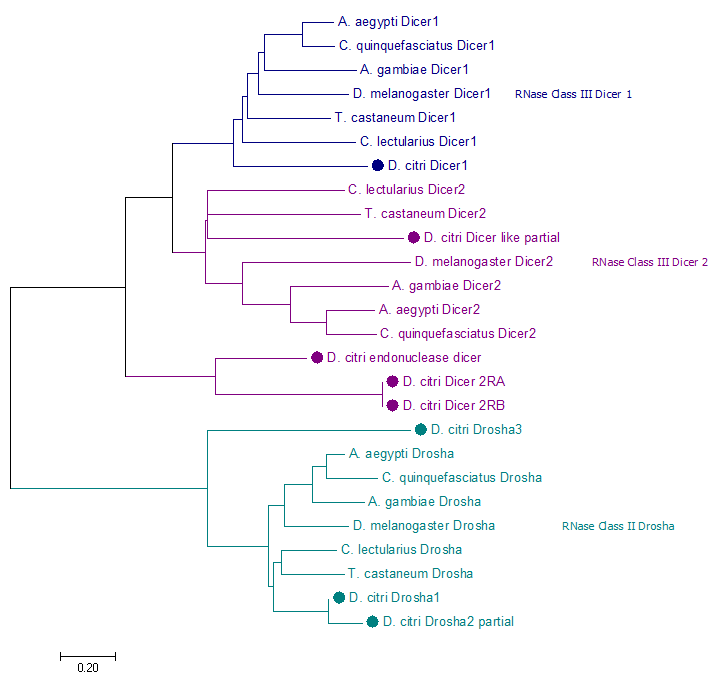


**Results and Discussion**

In *D. citri*, initial BLASTp searches with known insect Drosha proteins identified three loci encoding predicted Drosha proteins (Table 1). While all three genes identified show homology to Drosha, as shown by reciprocal BLAST analysis (Table 2), their position in the current *D. citri* genome assembly has made their annotation and analysis difficult. The first gene identified, currently named *D. citri drosha2 partial* (*drosha2 partial*), is located at the end of a contig, causing an artificial truncation of the gene. The second gene, currently named *D. citri drosha1* (*drosha1*) is near the end of another contig and was found within an intron of another annotated gene, *D. citri transmembrane protein 179*, making analysis of RNA-Seq data difficult. The third gene, currently named *D. citri drosha 3* (*drosha3*) is also near the end of a contig and, of the three, shares the least similarity to other known Drosha proteins.

EMBL EBI’s InterPro analysis of Drosha1 revealed that despite its small size this predicted protein is the most complete of all the *D. citri* Drosha proteins as it contains two RNase III domains and the dsRNA binding domain. InterPro analysis of Drosha2 partial identified one full and one truncated RNase III domain. This result is no doubt the result of *drosha2 partial’s* position at the end of the contig, resulting in the loss of the C-terminal end of the protein sequence, as opposed to a true loss of the second RNase III domain and dsRNA binding domain. InterPro analysis of Drosha3 only identified a dsRNA binding domain in this sequence. This result could indicate that this third *drosha* gene has become highly diverged over time or this may not be a complete gene and may represent the 3’ end of *drosha2 partial*.

Phylogenic analysis of insect Drosha proteins (Figure 1), and of RNase III class II and class III proteins (Figure 2) indicate that the three loci identified are homologous to other insect Drosha proteins. However, conclusions about the exact number of *D. citri* homologs and the relationship between Drosha proteins are unable to be drawn due to incomplete sequence information and genome fragmentation.

At least two and possibility three *drosha* genes have been identified in *D. citri*. Two *pasha* genes (the partner of *drosha*) have also been identified. This increase in miRNA processing components may increase the ability of *D. citri* cells to produce miRNAs but it is important to note the Drosha and Pasha have been shown to have other cellular functions within the cell ([Han et al., 2009](#_ENREF_4); [Karginov et al., 2010](#_ENREF_6); [Macias et al., 2012](#_ENREF_7)). Therefore, their duplication and subsequent maintenance in the *D. citri* genome may be unrelated to their function in the miRNA pathway.

**References**

1. Campbell, C. L., Black, W. C. th, Hess, A. M. and Foy, B. D. (2008) 'Comparative genomics of small RNA regulatory pathway components in vector mosquitoes', *BMC Genomics* 9: 425.
2. Connerty, P., Ahadi, A. and Hutvagner, G. (2015) 'RNA Binding Proteins in the miRNA Pathway', *Int J Mol Sci* 17(1).
3. Ha, M. and Kim, V. N. (2014) 'Regulation of microRNA biogenesis', *Nat Rev Mol Cell Biol* 15(8): 509-24.
4. Han, J., Pedersen, J. S., Kwon, S. C., Belair, C. D., Kim, Y. K., Yeom, K. H., Yang, W. Y., Haussler, D., Blelloch, R. and Kim, V. N. (2009) 'Posttranscriptional crossregulation between Drosha and DGCR8', *Cell* 136(1): 75-84.
5. Jaubert-Possamai, S., Rispe, C., Tanguy, S., Gordon, K., Walsh, T., Edwards, O. and Tagu, D. (2010) 'Expansion of the miRNA pathway in the hemipteran insect Acyrthosiphon pisum', *Mol Biol Evol* 27(5): 979-87.
6. Karginov, F. V., Cheloufi, S., Chong, M. M., Stark, A., Smith, A. D. and Hannon, G. J. (2010) 'Diverse endonucleolytic cleavage sites in the mammalian transcriptome depend upon microRNAs, Drosha, and additional nucleases', *Mol Cell* 38(6): 781-8.
7. Macias, S., Plass, M., Stajuda, A., Michlewski, G., Eyras, E. and Caceres, J. F. (2012) 'DGCR8 HITS-CLIP reveals novel functions for the Microprocessor', *Nat Struct Mol Biol* 19(8): 760-6.
8. Tomoyasu, Y., Miller, S. C., Tomita, S., Schoppmeier, M., Grossmann, D. and Bucher, G. (2008) 'Exploring systemic RNA interference in insects: a genome-wide survey for RNAi genes in Tribolium', *Genome Biol* 9(1): R10.
9. Xu, H. J., Chen, T., Ma, X. F., Xue, J., Pan, P. L., Zhang, X. C., Cheng, J. A. and Zhang, C. X. (2013) 'Genome-wide screening for components of small interfering RNA (siRNA) and micro-RNA (miRNA) pathways in the brown planthopper, Nilaparvata lugens (Hemiptera: Delphacidae)', *Insect Mol Biol* 22(6): 635-47.

# Supplementary Note 22: Pasha protein

**Introduction**

Pasha is the dsRNA binding protein (dRBP) partner of Drosha ([Denli et al., 2004](#_ENREF_1)). dRBPs have a motif allowing them to interact with dsRNA and have been shown to perform a variety of cellular functions within the cell ([Saunders and Barber, 2003](#_ENREF_5)). Pasha is known to be specifically involved in the microRNA (miRNA) pathway through interactions with its RNase III protein partner Drosha ([Denli et al., 2004](#_ENREF_1)). When miRNA precursors are transcribed from non-coding genes within a cell these primary transcripts form imperfect dsRNA hairpins which are bound by Pasha and processed by Drosha before leaving the nucleus where they are further processed by Dicer into mature miRNAs (reviewed in ([Ouellet et al., 2006](#_ENREF_4); [Lucas and Raikhel, 2013](#_ENREF_3)).

**Methods**

Pasha homolog sequences from *Drosophila melanogaster*, *Anopheles gambiae*, *Aedes aegypti*, *Culex quinquefasciatus*, *Tribolium castaneum* and *Cimex lectularius* were used to query the predicted *Diaphorina citri* protein sets (Diacit_International_psyllid_consortium_proteins_v1 and Diacit_RefSeq_proteins_Release_100) at i5k@NAL via BLASTp analysis. Putative homologs were identified, renamed, and manually annotated in Web Apollo using RNA-Seq data as evidence for transcript structure. MEGA 7 software was used to create multiple sequence alignments (ClustalW) and build neighbor-joining phylogenic trees from full length Pasha homologs.

Table 1: Number of Pasha homologs in insect species

| **Species** | **Pasha** |
| --- | --- |
| *D. melanogaster* | 1 |
| *A. gambiae* | 1 |
| *A. aegypti* | 1 |
| *C. quinquefasciatus* | 1 |
| *T. castaneum* | 1 |
| *C. lectularius* | 1 |
| *D. citri* | 2 |

Table 2: Bit score, query coverage, identity and e-value results from BLAST analysis of predicted Pasha 1 and Pasha 2 to insect Pasha homologs.

|  |  | ***D. melanogaster*** | ***A. gambiae*** | ***A. aegypti*** | ***C. quinquefasciatus*** | ***T. castaneum*** | ***C. lectularius*** |
| --- | --- | --- | --- | --- | --- | --- | --- |
| *D. citri* Pasha 1 | **Bit score** | 434 | 327 | 398 | 410 | 423 | 409 |
|  | **QC (Identity)** | 79%  (44%) | 78%  (46%) | 81% (41%) | 83%  (41%) | 77%  (46%) | 80%  (45%) |
|  | **E value** | 9.00E-146 | 3.00E-103 | 2.00E-132 | 3.00E-135 | 1.00E-141 | 3.00E-136 |
| *D. citri* Pasha 2 | **Bit score** | 340 | 312 | 303 | 301 | 326 | 301 |
|  | **QC (Identity)** | 86%  (37%) | 79%  (34%) | 84% (34%) | 84%  (33%) | 81%  (38%) | 85%  (33%) |
|  | **E value** | 6.00E-110 | 3.00E-98 | 5.00E-96 | 4.00E-94 | 2.00E-104 | 6.00E-95 |

Figure 1: Phylogenetic tree depicting clustering of insect Pasha homologs. Blue represents insects in the order Diptera. Teal represents insects in the order Coleoptera. Purple represents insects in the order Hemiptera. *D. citri* proteins are designated with a taxon marker.


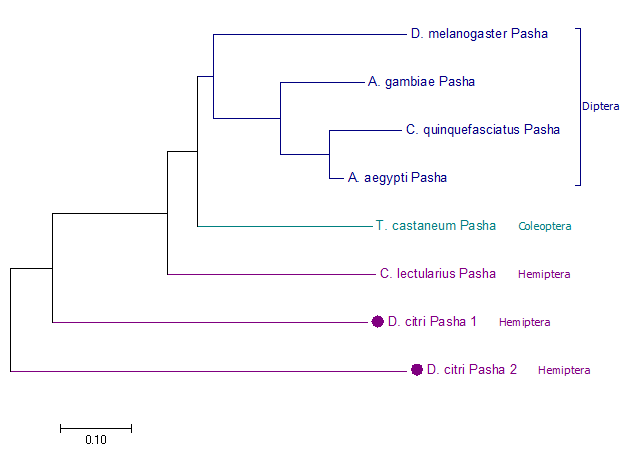


**Results and Discussion**

BLASTp analysis of the *D. citri* genome revealed two putative Pasha homologs (Table 1). We have named these genes *D. citri pasha 1* (*pasha 1*) and *D. citri pasha 2* (*pasha 2*). Both of these identified proteins have dsRNA binding domains and show homology to fly, mosquito, beetle and bedbug Pasha homologs (Table 2).

Phylogenetic analysis illustrates appropriate clustering of insect Pasha homologs, with the Diptera forming one clade and the Hemiptera proteins located more basally (Figure 1). While a single Pasha has been identified in the *D. melanogaster*, *A. gambiae*, *A. aegpti*, *C. quinquefasciatus*, *T. castaneum* and *C. lectularius* genomes, two have been found in the *D. citri* genome. This could be the result of gene loss of one Pasha homolog in studied model insects or, more likely, could be the result of gene duplication and rapid divergence of Pasha paralogs in *D. citri*. Interestingly, multiple Drosha proteins have also been identified in *D. citri*.

Figure 2: Phylogenetic tree depicting clustering of insect dRBPs. Loquacious homologs are shown in blue. R2D2 homologs are shown in teal. Pasha homologs are shown in purple. *D. citri* proteins are designated with a taxon marker.


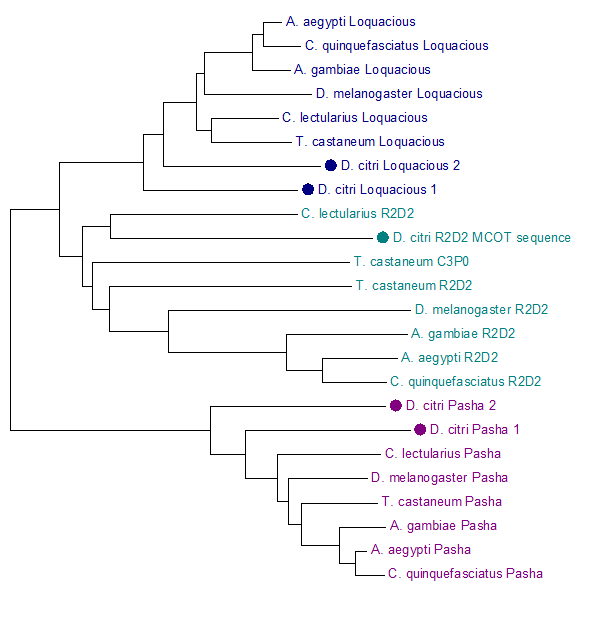


It is important to note that, to our knowledge, extensive functional analysis has not been performed on RNAi and miRNA core machinery genes in insects outside of *Drosophila*. However, if we assume that as in *Drosophila* Pasha 1 and Pasha 2 act as partners to Drosha one must speculate whether a specific *D. citri* Pasha protein partners with a specific Drosha protein, or whether these proteins participate in more of a free-for-all approach with available Pasha proteins partnering with any available Drosha protein. Further phylogenetic analysis of all known dRBPs involved in RNA silencing demonstrates Pasha 1 and Pasha 2 are more similar to other insect Pasha’s than other dRBPs (Loquacious and R2D2) (Figure 2) confirming the results of our reciprocal BLAST analysis (Table 2). It has been reported that the pea aphid *Acyrthosiphon pisum* has seen a duplication of genes involved in the miRNA pathway with 4 *pasha* genes, 2 *loquacious* genes, 2 *Dicer-1* genes, and 2 *Argonaute 1* genes. In contrast, the number of genes involved in the RNAi pathway in *A. pisum* is similar to the number reported in other insects with sequenced genomes ([Jaubert-Possamai et al., 2010](#_ENREF_2)). This first genome assembly in *D. citri* seems to show a similar trend with duplicated *drosha*, *pasha*, *loquacious* and possibly *Dicer-1* genes. Determining the function of these additional miRNA machinery genes, which have diverged but been maintained over evolutionary time, would be an enticing endeavor.

**References**

1. Denli, A. M., Tops, B. B., Plasterk, R. H., Ketting, R. F. and Hannon, G. J. (2004) 'Processing of primary microRNAs by the Microprocessor complex', Nature 432(7014): 231-5.
2. Jaubert-Possamai, S., Rispe, C., Tanguy, S., Gordon, K., Walsh, T., Edwards, O. and Tagu, D. (2010) 'Expansion of the miRNA pathway in the hemipteran insect Acyrthosiphon pisum', Mol Biol Evol 27(5): 979-87.
3. Lucas, K. and Raikhel, A. S. (2013) 'Insect microRNAs: biogenesis, expression profiling and biological functions', Insect Biochem Mol Biol 43(1): 24-38.
4. Ouellet, D. L., Perron, M. P., Gobeil, L. A., Plante, P. and Provost, P. (2006) 'MicroRNAs in gene regulation: when the smallest governs it all', J Biomed Biotechnol 2006(4): 69616.
5. Saunders, L. R. and Barber, G. N. (2003) 'The dsRNA binding protein family: critical roles, diverse cellular functions', FASEB J 17(9): 961-83.

# Supplementary Note 23: Loquacious and R2D2 proteins

**Introduction**

Loquacious (Loqs) and R2D2 are dsRNA binding proteins (dRBP) which play an essential role in the miRNA and RNAi pathways. These dsRBPs partner with Dicer during cleavage of dsRNA into small effector molecules (miRNAs and siRNAs) and, in the case of R2D2, aid in loading these small RNA molecules into the RNA-induced silencing complex (RISC) ([Liu et al., 2003](#_ENREF_5); [Leuschner et al., 2005](#_ENREF_4); [Saito et al., 2005](#_ENREF_11); [Liu et al., 2006](#_ENREF_6); [Liu et al., 2007](#_ENREF_7); [Okamura et al., 2011](#_ENREF_8)). In *Drosophila melanogaster* these two proteins have subfunctionalized so that Loqs interacts specifically with Dicer1 in the miRNA pathway and R2D2 interacts specifically with Dicer2 in the RNAi pathway (reviewed in ([Paroo et al., 2007](#_ENREF_9)).

Thus far there are limited functional studies of the core RNAi and miRNA machinery genes outside of the leading insect model *D. melanogaster*. However, the few studies which have been performed suggest that perhaps this subfunctionalization of dsRBPs may be a relatively recent event in the dipteran lineage. In *Aedes aegypti* an isoform of Loqs has recently been found to interact with siRNA components and influences the quantity of siRNAs present in mosquito cells ([Haac et al., 2015](#_ENREF_2)). In *Tribolium castaneum* knockdown of Loqs via RNAi did not result in the loss of miRNA phenotype seen in Ago1 knocked down individuals, suggesting the dsRBPs may act redundantly in small RNA pathways in *T. castaneum* ([Tomoyasu et al., 2008](#_ENREF_12)). Furthermore, more extensive studies in *D. melanogaster* indicate that in some situations Loqs can also associate with Dicer2 in the siRNA pathway ([Czech et al., 2008](#_ENREF_1)).

**Methods**

Loqs and R2D2 homolog sequences based on peer-reviewed literature were collected from NCBI and were used to query the predicted *Diaphorina citri* protein sets (Diacit_International_psyllid_ consortium_proteins_v1 and Diacit_RefSeq_proteins_Release_100) at i5k@NAL with the BLASTp tool. Plausible homologs were run through the Web Apollo genome browser in order to identify *D. citri* putative homologs. These homologs were manually annotated in Web Apollo using RNA-Seq data as evidence for transcript structure. Resulting protein sequences were run through a BLAST search on NCBI to confirm homology and domain conservation was confirmed through the domain database on NCBI and by EMBL EBI’s online InterPro program. MEGA 7 software was used to create multiple sequence alignments (ClustalW) and build neighbor-joining phylogenic trees from full length homologs.

Table 1: Number of Loquacious and R2D2 homologs in insect species. * Indicates evidence for this homolog was found in MCOT

| **Species** | **Loquacious** | **R2D2** |
| --- | --- | --- |
| *D. melanogaster* | 1 | 1 |
| *A. gambiae* | 1 | 1 |
| *A. aegypti* | 1 | 1 |
| *C. quinquefasciatus* | 1 | 1 |
| *T. castaneum* | 1 | 2 |
| *C. lectularius* | 1 | 1 |
| *D. citri* | 2 | 1* |

Table 2: Bit score, query coverage, identity and e-value results from BLAST analysis of predicted Loq1 and Loqs2 to insect Loqs Homologs

|  | **Top Blastp Hit** |  | ***D. melanogaster*** | ***A. gambiae*** | ***A. aegypti*** | ***C. quinquefasciatus*** | ***T. castaneum*** |
| --- | --- | --- | --- | --- | --- | --- | --- |
| *D. citri* Loqs1 | *M. destructor* Loqs | **Bit score** | 223 | 208 | 208 | 202 | 233 |
|  |  | **QC (Identity)** | 80%  (40%) | 84% (41%) | 85% (41%) | 92%  (38%) | 84%  (43%) |
|  |  | **E value** | 1.00E-68 | 1.00E-64 | 1.00E-64 | 9.00E-62 | 1.00E-73 |
| *D. citri* Loqs2 | *M. destructor* Loqs | **Bit score** | 213 | 202 | 222 | 207 | 224 |
|  |  | **QC (Identity)** | 98%  (40%) | 75% (46%) | 75% (49%) | 97%  (44%) | 96%  (43%) |
|  |  | **E value** | 1.00E-65 | 2.00E-63 | 8.00E-71 | 6.00E-65 | 2.00E-71 |

**Results and Discussion**

Most insects used in our BLASTp analysis have been shown to have one *loqs* ortholog and one *r2d2* ortholog. The one exception to this is *T. castaneum* which has been shown to have a second, highly diverged, *r2d2* paralog named *C3P0* ([Tomoyasu et al., 2008](#_ENREF_12)) (Table 1). BLASTp analysis of the *D. citri* genome identified two loci similar to both Loqs and R2D2. Reciprocal BLAST analysis of these two putative homologs identified them both as Loqs orthologs (Table 2).

Figure 1: Phylogenetic tree depicting clustering of insect dRBPs. Loquacious homologs are shown in blue. R2D2 homologs are shown in teal. Pasha homologs are shown in purple. *D. citri* proteins are designated with a taxon marker.


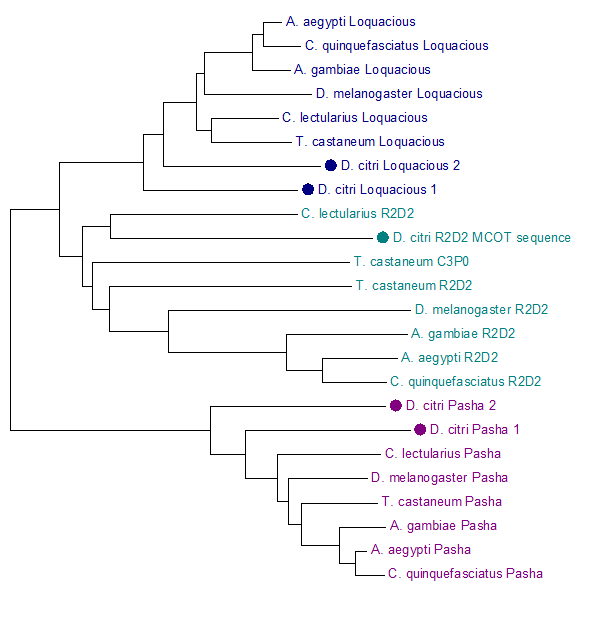


Furthermore, phylogenetic analysis of all three dsRBPs involved in RNA silencing mechanisms show that these *D. citri* genes are found in the insect Loqs clade (Figure 1). Therefore, we have tentatively named these two genes *D. citri loqs1* (*loqs1*) and *D. citri loqs2* (*loqs2*). The presence of multiple *loqs* paralogs is not unprecedented as two *loqs* paralogs have also been identified in the pea aphid *Acyrthosiphon pisum* ([Jaubert-Possamai et al., 2010](#_ENREF_3)). Initially we are unable to identify a true R2D2 ortholog in the *D. citri* genome, therefore, we used insect R2D2 orthologs to search the MCOT transcriptome. This analysis identified a gene showing homology to insect R2D2 proteins and while fragments of this gene could be found in the *D. citri* genome, fragmentation and possible misassembly of the genome at this location made annotation impossible. Future genome assemblies will need to be examined to determine if a true *r2d2* ortholog exists in the *D. citri* genome. If *D. citri* is in fact lacking an *r2d2* ortholog this is not unprecedented as the recent publication of the grasshopper *Shirakiacris shirakii* transcriptome suggests that it may lack an *r2d2* ortholog ([Qiu et al., 2016](#_ENREF_10)) and, as suggested in the introduction, dsRBPs may be capable of functioning outside of their canonical pathway. If this is the case in *D. citri*, the loss of R2D2 would not necessarily be catastrophic, as one of the two Loqs proteins could potentially act in the RNAi pathway. Loqs and R2D2 are very similar proteins and domain analysis of insect Loqs and R2D2 orthologs indicate that Loqs typically contains three dsRNA binding domains, at least one interferon-inducible dsRNA-dependent protein kinase activator A domain and a Staufen domain while R2D2 contains only two dsRNA binding domains. Domain analysis of the Loqs orthlogs found in the *D. citri* genome and the R2D2 ortholog found in the MCOT transcriptome showed similar trends with Loqs2 containing three dsRNA binding domains, 2 interferon-inducible dsRNA-dependent protein kinase activator A domains and a Staufen domain, Loqs1 containing two dsRNA binding domains and an interferon-inducible dsRNA-dependent protein kinase activator A domain and the potential *D. citri* R2D2 sequence containing two dsRNA binding domains. Future experimental and functional analysis is required to provide further insights into the evolutionary origin and history of the *loqs/r2d2* homologs in *D. citri* and other species.

**References**

1. Czech, B., Malone, C. D., Zhou, R., Stark, A., Schlingeheyde, C., Dus, M., Perrimon, N., Kellis, M., Wohlschlegel, J. A., Sachidanandam, R. et al. (2008) 'An endogenous small interfering RNA pathway in Drosophila', *Nature* 453(7196): 798-802.
2. Haac, M. E., Anderson, M. A., Eggleston, H., Myles, K. M. and Adelman, Z. N. (2015) 'The hub protein loquacious connects the microRNA and short interfering RNA pathways in mosquitoes', *Nucleic Acids Res* 43(7): 3688-700.
3. Jaubert-Possamai, S., Rispe, C., Tanguy, S., Gordon, K., Walsh, T., Edwards, O. and Tagu, D. (2010) 'Expansion of the miRNA pathway in the hemipteran insect Acyrthosiphon pisum', *Mol Biol Evol* 27(5): 979-87.
4. Leuschner, P. J., Obernosterer, G. and Martinez, J. (2005) 'MicroRNAs: Loquacious speaks out', *Curr Biol* 15(15): R603-5.
5. Liu, Q., Rand, T. A., Kalidas, S., Du, F., Kim, H. E., Smith, D. P. and Wang, X. (2003) 'R2D2, a bridge between the initiation and effector steps of the Drosophila RNAi pathway', *Science* 301(5641): 1921-5.
6. Liu, X., Jiang, F., Kalidas, S., Smith, D. and Liu, Q. (2006) 'Dicer-2 and R2D2 coordinately bind siRNA to promote assembly of the siRISC complexes', *RNA* 12(8): 1514-20.
7. Liu, X., Park, J. K., Jiang, F., Liu, Y., McKearin, D. and Liu, Q. (2007) 'Dicer-1, but not Loquacious, is critical for assembly of miRNA-induced silencing complexes', *RNA* 13(12): 2324-9.
8. Okamura, K., Robine, N., Liu, Y., Liu, Q. and Lai, E. C. (2011) 'R2D2 organizes small regulatory RNA pathways in Drosophila', *Mol Cell Biol* 31(4): 884-96.
9. Paroo, Z., Liu, Q. and Wang, X. (2007) 'Biochemical mechanisms of the RNA-induced silencing complex', *Cell Res* 17(3): 187-94.
10. Qiu, Z., Liu, F., Lu, H., Yuan, H., Zhang, Q. and Huang, Y. (2016) 'De Novo Assembly and Characterization of the Transcriptome of Grasshopper Shirakiacris shirakii', *Int J Mol Sci* 17(7).
11. Saito, K., Ishizuka, A., Siomi, H. and Siomi, M. C. (2005) 'Processing of pre-microRNAs by the Dicer-1-Loquacious complex in Drosophila cells', *PLoS Biol* 3(7): e235.
12. Tomoyasu, Y., Miller, S. C., Tomita, S., Schoppmeier, M., Grossmann, D. and Bucher, G. (2008) 'Exploring systemic RNA interference in insects: a genome-wide survey for RNAi genes in Tribolium', *Genome Biol* 9(1): R10.

# Supplementary Note 24: Argonaute and PIWI proteins

**Introduction**

Argonaute (AGO) proteins play an essential role in RNA-silencing mechanisms and have been implicated in the RNA interference (RNAi), micro RNA (miRNA), and transcriptional gene silencing (rasiRNA/piRNA) pathways (reviewed in ([Parker and Barford, 2006](#_ENREF_19); [Meister, 2013](#_ENREF_14)). They function to present small RNA guide molecules to their complementary targets through the RNA-induced silencing complex (RISC) or the RNA-induced initiation of transcription gene silencing (RITS) complex ([Hammond et al., 2001](#_ENREF_6); [Verdel et al., 2004](#_ENREF_24)). Additionally, AGO proteins provide the ‘Slicer’ catalytic activity that is required for mRNA cleavage in some RNA silencing pathways ([Liu et al., 2004](#_ENREF_11); [Meister et al., 2004](#_ENREF_15)). AGO proteins are varied with respect to sequence and can be divided into subfamilies including the AGO and PIWI subfamilies. Despite their variability AGO proteins contain four conserved domains, an amino terminal (N) domain, a PAZ (PIWI-Argonaute-Zwille) domain, a MID domain, and a C-terminal PIWI domain (reviewed in ([Parker and Barford, 2006](#_ENREF_19); [Meister, 2013](#_ENREF_14)). Structural studies of these domains have revealed that the PAZ domain functions in binding the 3’ end of small RNA guides ([Lingel et al., 2003](#_ENREF_9); [Song et al., 2003](#_ENREF_21); [Yan et al., 2003](#_ENREF_27); [Ma et al., 2004](#_ENREF_13)), the N domain is responsible for small RNA loading ([Kwak and Tomari, 2012](#_ENREF_8)), the MID domain is responsible for anchoring the 5’ end of the guide RNA ([Jinek and Doudna, 2009](#_ENREF_7)), and the PIWI domain provides the ‘Slicer’ enzymatic activity of the protein ([Liu et al., 2004](#_ENREF_11); [Rivas et al., 2005](#_ENREF_20); [Parker and Barford, 2006](#_ENREF_19); [Meister, 2013](#_ENREF_14)).

In many model organisms, including *Arabidopsis thaliana*, *Caenorhabditis elegans* and *Drosophila melanogaster*, AGO proteins have subfunctionalized with specific AGO proteins operating in specific RNA-silencing pathways ([Cikaluk et al., 1999](#_ENREF_3); [Lynn et al., 1999](#_ENREF_12); [Tabara et al., 1999](#_ENREF_22); [Grishok et al., 2001](#_ENREF_4)). In *D. melanogaster*, the insect model for which the RNAi core machinery genes have been best studied, there are five AGO family members. Dm-AGO1 is involved in the miRNA pathway, Dm-AGO2 is involved in the RNAi pathway ([Hammond et al., 2000](#_ENREF_5); [Okamura et al., 2004](#_ENREF_17)) while Dm-AGO3, Dm-PIWI and Dm-Aub (all members of the PIWI subfamily) are involved in transcriptional silencing ([Kennerdell et al., 2002](#_ENREF_10); [Pal-Bhadra et al., 2002](#_ENREF_24); [Aravin et al., 2004](#_ENREF_1)) (reviewed in ([Lippman and Martienssen, 2004](#_ENREF_10); [Meister and Tuschl, 2004](#_ENREF_16); [Parker and Barford, 2006](#_ENREF_19); [Meister, 2013](#_ENREF_14)). Functional studies in *Tribolium castaneum* indicate that similar subfunctionalization exists in beetles as well with Tc-Ago1 functioning in the miRNA pathway and Tc-Ago 2a and Tc-Ago 2b functioning in the RNAi pathway ([Tomoyasu et al., 2008](#_ENREF_23)).

**Methods**

AGO homolog sequences from a variety of insects were obtained from NCBI, ImmunoDB and the i5k workspace. These sequences were then used to query the predicted *Diaphorina citri* protein sets (Diacit_International_psyllid_consortium_proteins_v1and Diacit_RefSeq_

proteins_Release_100) at i5k@NAL. Loci encoding putative homologs were identified and manually annotated in Web Apollo using RNA-Seq data as evidence for transcript structure. EMBL EBI’s online InterPro program was used for domain identification. Multiple sequence alignments of insect Ago homologs were performed using the ClustalW program (MEGA7 software). A neighbor-joining phylogenetic tree was constructed using full-length protein sequences in MEGA7.

Table 1: Argonaute family genes in *D. citri* and related organisms. Note: Many mosquito genes are given the PIWI nomenclature even though they are Aub-like.

| **Species** | **AGO1** | **AGO2** | **AGO3** | **PIWI/Aub** |
| --- | --- | --- | --- | --- |
| *D. melanogaster* | 1 | 1 | 1 | 2 |
| *A. gambiae* | 1 | 1 | 1 | 2 |
| *A. aegypti* | 2 | 1 | 1 | 7 |
| *C. quinquefasciatus* | 1 | 2 | 1 | 6 |
| *T. castaneum* | 1 | 2 | 1 | 1 |
| *C. lectularius* | 2 | 2 | 1 | 4 |
| *D. citri* | 1 | 1 | 1 | 1 |

Table 2: Bit score, query coverage, identity and e-value results from BLAST analysis of predicted AGO1, AGO2, AGO3, Aub RA and Aub RB proteins to their presumptive orthologs.

| **Species** |  | ***D. citri* AGO1** | ***D. citri***  **AGO2** | ***D. citri***  **AGO3** | ***D. citri***  **Aub RA** | ***D. citri***  **Aub RB** |
| --- | --- | --- | --- | --- | --- | --- |
| *D. melanogaster* | **Bit score** | 1245 | 214 | 629 | 376 | 368 |
|  | **QC (Identity)** | 91%  (90%) | 98%  (47%) | 87%  (41%) | 82%  (35%) | 82%  (34%) |
|  | **E value** | 0 | 2.00E-59 | 0 | 6.00E-121 | 4.00E-118 |
| *A. gambiae* | **Bit score** | 1248 | 238 | 696 | 349 | 363 |
|  | **QC (Identity)** | 91%  (93%) | 94%  (45%) | 92%  (43%) | 94%  (36%) | 93%  (35%) |
|  | **E value** | 0 | 4.00E-68 | 0 | 5.00E-110 | 5.00E-115 |
| *A. aegypti* | **Bit score** | 1258 | 274 | 728 | 408 | 396 |
|  | **QC (Identity)** | 99%  (86%) | 100%  (50%) | 96%  (43%) | 93%  (37%) | 93%  (36%) |
|  | **E value** | 0 | 2.00E-80 | 0 | 4.00E-132 | 5.00E-128 |
| *C. quinquefasciatus* | **Bit score** | 1257 | 261 | 533 | 401 | 386 |
|  | **QC (Identity)** | 96%  (87%) | 99%  (51%) | 76%  (40%) | 93%  (37%) | 93%  (36%) |
|  | **E value** | 0 | 6.00E-76 | 4.00E-177 | 4.00E-130 | 1.00E-124 |
| *T. castaneum* | **Bit score** | 320 | 289 | 657 | 381 | 362 |
|  | **QC (Identity)** | 18%  (96%) | 99%  (56%) | 94%  (41%) | 92%  (38%) | 92%  (36%) |
|  | **E value** | 7.00E-105 | 2.00E-86 | 0 | 2.00E-121 | 2.00E-114 |
| *C. lectularius* | **Bit score** | 301 | 300 | 664 | 456 | 450 |
|  | **QC (Identity)** | 85%  (33%) | 99%  (59%) | 100%  (39%) | 91%  (42) | 91%  (41%) |
|  | **E value** | 1.00E-89 | 2.00E-89 | 0 | 3.00E-154 | 6.00E-149 |

**Results and Discussion**

In *D. citri*, BLASTp searches with known insect AGO proteins identified four loci encoding predicted AGO proteins (Table 1). Following the naming convention from previously studied insects these genes have been named *D. citri Argonaute 1* (*AGO1*), *D. citri Argonaute 2* (*AGO2*), *D. citri Argonaute 3* (*AGO3*) and *D. citri aubergine* (*aub*). RNA-Seq data suggests that *aub* may have at least two isoforms and they have been designated *aubRA* and *aubRB* (Table 2). This result is not surprising as isoforms of the PIWI/Aub class of Ago genes have been identified in other insects.

Domain analysis using EMBL-EBI’s InterPro indicate AGO1 contains the N, Mid, PAZ and PIWI domains characteristic of AGO proteins. In contrast, AGO2, AGO3, and Aub were only found to contain the PAZ and PIWI domains. Phylogenetic analysis shows that *D. citri* AGO1, AGO2, AGO3 and Aub cluster with representative genes from other insect taxa (Figure 1).

It is interesting to note that *D. citri* appears to have a minimal number of AGO proteins as has also been observed in other insects such as *Bombyx mori (*[*Wang et al., 2013*](#_ENREF_25)*)*, *Nilaparvata lugens (*[*Xu et al., 2013*](#_ENREF_26)*)*, *T. castaneum (*[*Tomoyasu et al., 2008*](#_ENREF_23)*),* and *D. melanogaster*. It does not appear to have undergone expansions in the PIWI class of AGO proteins, as seen in several species of mosquitoes ([Campbell et al., 2008](#_ENREF_2)) nor does it have AGO proteins showing homology to the expanded class of secondary AGO proteins seen in *C. elegans* ([Yigit et al., 2006](#_ENREF_28)).

Figure 1: Phylogenetic tree depicting clustering of insect Argonaute family proteins. Purple clades represent the canonical AGO subclass of proteins typically involved in post transcriptional silencing (RNAi and miRNA pathways). Blue clades represent the PIWI subclass of proteins typically involved in transcriptional silencing (rasiRNA/piRNA pathways). *D. citri* proteins are marked with a taxon marker.


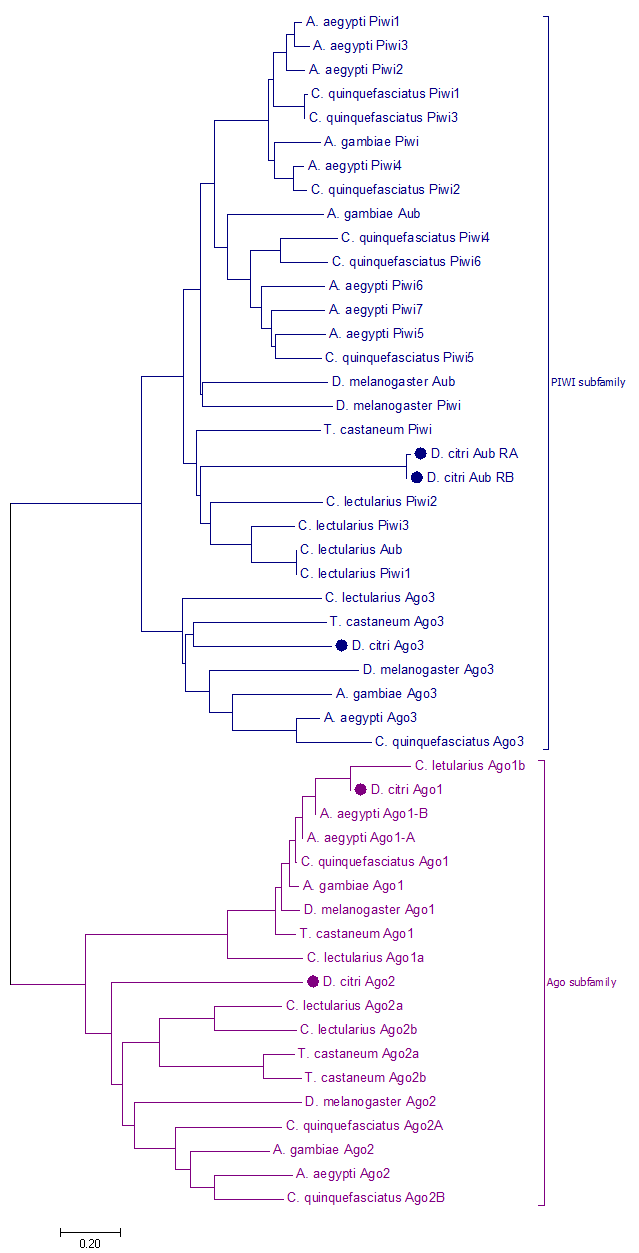


**References**

1. Aravin, A. A., Klenov, M. S., Vagin, V. V., Bantignies, F., Cavalli, G. and Gvozdev, V. A. (2004) 'Dissection of a natural RNA silencing process in the Drosophila melanogaster germ line', *Mol Cell Biol* 24(15): 6742-50.
2. Campbell, C. L., Black, W. C. th, Hess, A. M. and Foy, B. D. (2008) 'Comparative genomics of small RNA regulatory pathway components in vector mosquitoes', *BMC Genomics* 9: 425.
3. Cikaluk, D. E., Tahbaz, N., Hendricks, L. C., DiMattia, G. E., Hansen, D., Pilgrim, D. and Hobman, T. C. (1999) 'GERp95, a membrane-associated protein that belongs to a family of proteins involved in stem cell differentiation', *Mol Biol Cell* 10(10): 3357-72.
4. Grishok, A., Pasquinelli, A. E., Conte, D., Li, N., Parrish, S., Ha, I., Baillie, D. L., Fire, A., Ruvkun, G. and Mello, C. C. (2001) 'Genes and mechanisms related to RNA interference regulate expression of the small temporal RNAs that control C. elegans developmental timing', *Cell* 106(1): 23-34.
5. Hammond, S. M., Bernstein, E., Beach, D. and Hannon, G. J. (2000) 'An RNA-directed nuclease mediates post-transcriptional gene silencing in Drosophila cells', *Nature* 404(6775): 293-6.
6. Hammond, S. M., Boettcher, S., Caudy, A. A., Kobayashi, R. and Hannon, G. J. (2001) 'Argonaute2, a link between genetic and biochemical analyses of RNAi', *Science* 293(5532): 1146-50.
7. Jinek, M. and Doudna, J. A. (2009) 'A three-dimensional view of the molecular machinery of RNA interference', *Nature* 457(7228): 405-12.
8. Kennerdell, J. R., Yamaguchi, S. and Carthew, R. W. (2002) 'RNAi is activated during Drosophila oocyte maturation in a manner dependent on aubergine and spindle-E', *Genes Dev* 16(15): 1884-9.
9. Kwak, P. B. and Tomari, Y. (2012) 'The N domain of Argonaute drives duplex unwinding during RISC assembly', *Nat Struct Mol Biol* 19(2): 145-51.
10. Lingel, A., Simon, B., Izaurralde, E. and Sattler, M. (2003) 'Structure and nucleic-acid binding of the Drosophila Argonaute 2 PAZ domain', *Nature* 426(6965): 465-9.
11. Lippman, Z. and Martienssen, R. (2004) 'The role of RNA interference in heterochromatic silencing', *Nature* 431(7006): 364-70.
12. Liu, J., Carmell, M. A., Rivas, F. V., Marsden, C. G., Thomson, J. M., Song, J. J., Hammond, S. M., Joshua-Tor, L. and Hannon, G. J. (2004) 'Argonaute2 is the catalytic engine of mammalian RNAi', *Science* 305(5689): 1437-41.
13. Lynn, K., Fernandez, A., Aida, M., Sedbrook, J., Tasaka, M., Masson, P. and Barton, M. K. (1999) 'The PINHEAD/ZWILLE gene acts pleiotropically in Arabidopsis development and has overlapping functions with the ARGONAUTE1 gene', *Development* 126(3): 469-81.
14. Ma, J. B., Ye, K. and Patel, D. J. (2004) 'Structural basis for overhang-specific small interfering RNA recognition by the PAZ domain', *Nature* 429(6989): 318-22.
15. Meister, G. (2013) 'Argonaute proteins: functional insights and emerging roles', *Nat Rev Genet* 14(7): 447-59.
16. Meister, G., Landthaler, M., Patkaniowska, A., Dorsett, Y., Teng, G. and Tuschl, T. (2004) 'Human Argonaute2 mediates RNA cleavage targeted by miRNAs and siRNAs', *Mol Cell* 15(2): 185-97.
17. Meister, G. and Tuschl, T. (2004) 'Mechanisms of gene silencing by double-stranded RNA', *Nature* 431(7006): 343-9.
18. Okamura, K., Ishizuka, A., Siomi, H. and Siomi, M. C. (2004) 'Distinct roles for Argonaute proteins in small RNA-directed RNA cleavage pathways', *Genes Dev* 18(14): 1655-66.
19. Pal-Bhadra, M., Bhadra, U. and Birchler, J. A. (2002) 'RNAi related mechanisms affect both transcriptional and posttranscriptional transgene silencing in Drosophila', *Mol Cell* 9(2): 315-27.
20. Parker, J. S. and Barford, D. (2006) 'Argonaute: A scaffold for the function of short regulatory RNAs', *Trends Biochem Sci* 31(11): 622-30.
21. Rivas, F. V., Tolia, N. H., Song, J. J., Aragon, J. P., Liu, J., Hannon, G. J. and Joshua-Tor, L. (2005) 'Purified Argonaute2 and an siRNA form recombinant human RISC', *Nat Struct Mol Biol* 12(4): 340-9.
22. Song, J. J., Liu, J., Tolia, N. H., Schneiderman, J., Smith, S. K., Martienssen, R. A., Hannon, G. J. and Joshua-Tor, L. (2003) 'The crystal structure of the Argonaute2 PAZ domain reveals an RNA binding motif in RNAi effector complexes', *Nat Struct Biol* 10(12): 1026-32.
23. Tabara, H., Sarkissian, M., Kelly, W. G., Fleenor, J., Grishok, A., Timmons, L., Fire, A. and Mello, C. C. (1999) 'The rde-1 gene, RNA interference, and transposon silencing in C. elegans', *Cell* 99(2): 123-32.
24. Tomoyasu, Y., Miller, S. C., Tomita, S., Schoppmeier, M., Grossmann, D. and Bucher, G. (2008) 'Exploring systemic RNA interference in insects: a genome-wide survey for RNAi genes in Tribolium', *Genome Biol* 9(1): R10.
25. Verdel, A., Jia, S., Gerber, S., Sugiyama, T., Gygi, S., Grewal, S. I. and Moazed, D. (2004) 'RNAi-mediated targeting of heterochromatin by the RITS complex', *Science* 303(5658): 672-6.
26. Wang, G. H., Jiang, L., Zhu, L., Cheng, T. C., Niu, W. H., Yan, Y. F. and Xia, Q. Y. (2013) 'Characterization of Argonaute family members in the silkworm, Bombyx mori', *Insect Sci* 20(1): 78-91.
27. Xu, H. J., Chen, T., Ma, X. F., Xue, J., Pan, P. L., Zhang, X. C., Cheng, J. A. and Zhang, C. X. (2013) 'Genome-wide screening for components of small interfering RNA (siRNA) and micro-RNA (miRNA) pathways in the brown planthopper, Nilaparvata lugens (Hemiptera: Delphacidae)', *Insect Mol Biol* 22(6): 635-47.
28. Yan, K. S., Yan, S., Farooq, A., Han, A., Zeng, L. and Zhou, M. M. (2003) 'Structure and conserved RNA binding of the PAZ domain', *Nature* 426(6965): 468-74.
29. Yigit, E., Batista, P. J., Bei, Y., Pang, K. M., Chen, C. C., Tolia, N. H., Joshua-Tor, L., Mitani, S., Simard, M. J. and Mello, C. C. (2006) 'Analysis of the C. elegans Argonaute family reveals that distinct Argonautes act sequentially during RNAi', *Cell* 127(4): 747-57.

# Supplementary Note 25: Tudor Staphylococcal Nuclease protein

**Introduction**

Tudor Staphylococcal Nuclease protein (Tudor SN or TSN) has been found to be a component of the holo-RISC complex in *Drosophila melanogaster*, *Caenorhabditis elegans* and mammals. TSN in *D. melanogaster* contains five Staphylococcal/Micrococcal nuclease domains and a Tudor domain ([Caudy et al., 2003](#_ENREF_1)).

**Methods**

TSN protein sequences were used to query the predicted *Diaphorina citri* protein sets (Diacit_International_psyllid_consortium_proteins_v1and Diacit_RefSeq_proteins_Release

_100) at i5k@NAL via the BLASTp tool. Loci encoding putative homologs were identified and manually annotated in Web Apollo using RNA-Seq data as evidence for transcript structure. EMBL EBI’s online InterPro program was used for domain identification. NCBI databases were used for reciprocal blast analysis of predicted proteins. Multiple sequence alignments of insect homologs were performed using the ClustalW program (MEGA7 software). A neighbor-joining phylogenetic tree was constructed using full-length protein sequences in MEGA7.

Table 1: Number of TSN homologs in insect species

|  | **TSN** |
| --- | --- |
| *D. melanogaster* | 1 |
| *A. gambiae* | 1 |
| *A. aegypti* | 1 |
| *C. quinquefasciatus* | 1 |
| *D. citri* | 2 |

Table 2: Bit score, query coverage, identity and e-value results from BLAST analysis of predicted TSN1 and TSN2 to TSN insect orthologs.

|  |  | ***D. melanogaster*** | ***A. gambiae*** | ***A. aegypti*** | ***C. quinquefasciatus*** |
| --- | --- | --- | --- | --- | --- |
| *D. citri* TSN1 | **Bit score** | 402 | 409 | 429 | 429 |
|  | **QC (Identity)** | 96% (45%) | 95% (45%) | 98% (46%) | 96% (46%) |
|  | **E value** | 2.00E-130 | 5.00E-136 | 1.00E-141 | 2.00E-141 |
| *D. citri* TSN2 | **Bit score** | 256 | 246 | 266 | 268 |
|  | **QC (Identity)** | 96% (38%) | 95% (39%) | 95% (41%) | 95% (40%) |
|  | **E value** | 9.00E-77 | 1.00E-73 | 8.00E-81 | 2.00E-81 |

**Results and Conclusions**

Initial genome searches identified four loci with significant BLAST hits to TSN insect homologs. However, reciprocal BLAST analysis suggests that only two of these genes are likely orthologous to fly and mosquito TSN (Table 2). We have named these genes *D. citri* *TSN1* (*TSN1*) and *D. citri TSN2* (*TSN2*) (Table 1).

Domain analysis reveals that *D. citri* TSN1 contains 3 staphylococcal nuclease domains and one tudor domain. *D. citri* TSN 2 contains 3 staphylococcal nuclease domains and does not contain a tudor domain. However, *D. citri* TSN2 is located at the end of a contig and the predicted protein is likely missing C-terminal sequence information. Both *D. citri* TSN proteins have fewer staphylococcal nuclease domains than is seen in *D. melanogaster* but this is not surprising as the number of staphylococcal domains in TSN homologs varies across species (4 in *Bombyx mori* and humans, 5 in mosquitoes) ([Li et al., 2008](#_ENREF_2); [Zhu et al., 2013](#_ENREF_3)). Phylogenetic analysis depicting the relationship between insect TSN homologs is shown below.

Figure 1: Phylogenetic tree of insect TSN homologs. Each species is depicted with a different color. *D. citri* is marked with a taxon marker.


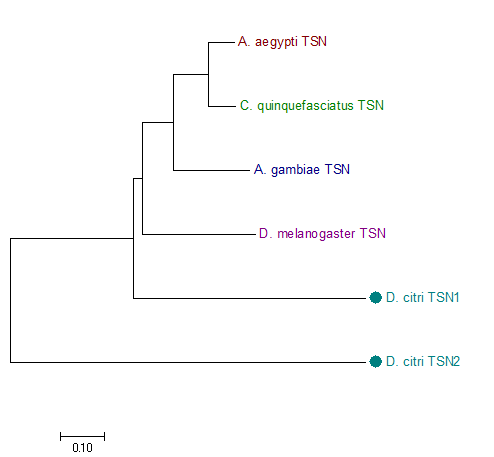


**References**

1. Caudy, A. A., Ketting, R. F., Hammond, S. M., Denli, A. M., Bathoorn, A. M., Tops, B. B., Silva, J. M., Myers, M. M., Hannon, G. J. and Plasterk, R. H. (2003) 'A micrococcal nuclease homologue in RNAi effector complexes', *Nature* 425(6956): 411-4.
2. Li, C. L., Yang, W. Z., Chen, Y. P. and Yuan, H. S. (2008) 'Structural and functional insights into human Tudor-SN, a key component linking RNA interference and editing', *Nucleic Acids Res* 36(11): 3579-89.
3. Zhu, L., Tatsuke, T., Mon, H., Li, Z., Xu, J., Lee, J. M. and Kusakabe, T. (2013) 'Characterization of Tudor-sn-containing granules in the silkworm, Bombyx mori', *Insect Biochem Mol Biol* 43(8): 664-74.

# Supplementary Note 26: Vasa intronic gene

**Introduction**

When the RNA-induced silencing complex (RISC) was first identified in *Drosophila melanogaster* S2 cells, two RNA binding proteins, Fragile X Mental Retardation (FMR) and Vasa Intronic Gene (VIG) were found to be components of the complex ([Caudy et al., 2002](#_ENREF_3)). VIG has since been shown to play an important role in RNAi in *Caenorhabditis elegans* as well, as inactivation of Ce VIG-1 resulted in decreased RNAi activity ([Caudy et al., 2003](#_ENREF_2)), however the function of VIG in the RISC is still poorly understood.

**Methods**

VIG sequences were used to query the predicted *Diaphorina citri* protein sets (Diacit_International_psyllid_consortium_proteins_v1and Diacit_RefSeq_proteins_Release

_100) at i5k@NAL via the BLASTp tool. Loci encoding putative homologs were identified and manually annotated in Web Apollo using RNA-Seq data as evidence for transcript structure. NCBI databases were used for reciprocal blast analysis of the predicted protein. Multiple sequence alignments of insect homologs were performed using the ClustalW program (MEGA7 software). A neighbor-joining phylogenetic tree was constructed using full-length protein sequences in MEGA7.

Table 1: Number of VIG homologs seen in related insect species

|  | **VIG** |
| --- | --- |
| *D. melanogaster* | 2 |
| *A. gambiae* | 1 |
| *A. aegypti* | 1 |
| *D. citri* | 1 |

Table 2: Bit score, query coverage, identity and e-value results from BLAST analysis of predicted VIG to insect VIG homologs.

|  |  | ***D. melanogaster*** | ***A. gambiae*** | ***A. aegypti*** |
| --- | --- | --- | --- | --- |
| *D. citri* VIG | **Bit score** | 116 | 114 | 104 |
|  | **QC (Identity)** | 70% (43%) | 70% (39%) | 74% (40%) |
|  | **E value** | 1.00E-29 | 2.00E-29 | 5.00E-26 |

**Results and Conclusions**

BLASTp analysis of the *D. citri* genome revealed one putative VIG ortholog (Table 1). We have named this gene *D. citri VIG* (*VIG*). Blast analysis indicates that *D. melanogaster* has two VIG genes, although to our knowledge VIG2 has not been implicated in RNAi. The mosquito species *Anopheles gambiae* and *Aedes aegypti* each have one VIG ortholog, although only the *A. aegypti* gene appears to be annotated (Table 1). Reciprocal BLAST analysis with the predicted *D. citri* VIG protein sequence confirms that *D. citri* VIG is orthologous to fly and mosquito VIG (Table 2).


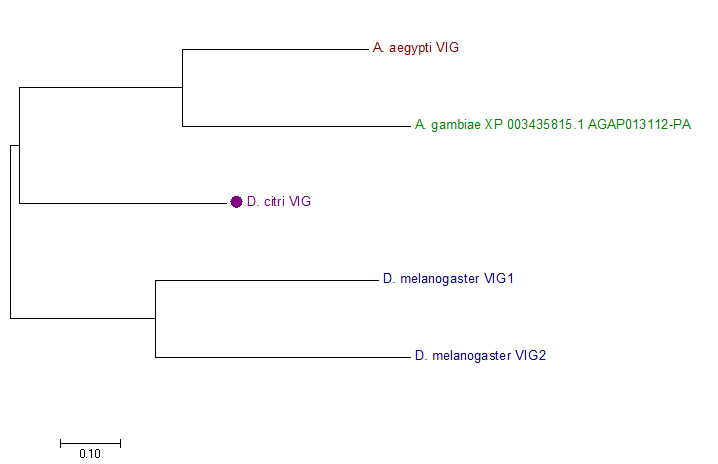


Phylogenetic analysis of fly and mosquito VIG homologs indicate that the two *D. melanogaster* genes are likely paralogs, with *D. citri* *VIG* showing more similarity to mosquito *VIG* genes (Figure 1).

**References**

1. Caudy, A. A., Ketting, R. F., Hammond, S. M., Denli, A. M., Bathoorn, A. M., Tops, B. B., Silva, J. M., Myers, M. M., Hannon, G. J. and Plasterk, R. H. (2003) 'A micrococcal nuclease homologue in RNAi effector complexes', *Nature* 425(6956): 411-4.
2. Caudy, A. A., Myers, M., Hannon, G. J. and Hammond, S. M. (2002) 'Fragile X-related protein and VIG associate with the RNA interference machinery', *Genes Dev* 16(19): 2491-6.

# Supplementary Note 27: Armitage

**Introduction**

Armitage (Armi) is a homolog of the *Arabidopsis thaliana* gene SDE3. SDE3 has been identified as an ATP dependent RNA helicase involved in RNAi in plants ([Dalmay et al., 2001](#_ENREF_5); [Willmann, 2001](#_ENREF_17)) and has been shown, in a variety of studies, to be required for RNAi in the fruit fly *Drosophila melanogaster*. Armi is necessary for silencing select mRNAs involved in oogenesis ([Cook et al., 2004](#_ENREF_4)) and *armi* mutants have been shown to be defective in the production of RISC ([Tomari et al., 2004](#_ENREF_16)). Furthermore, Armi has been shown to be one of many factors essential for primary piwiRNA (piRNA) biogenesis ([Malone et al., 2009](#_ENREF_10); [Haase et al., 2010](#_ENREF_7); [Olivieri et al., 2010](#_ENREF_11)).

**Methods**

Armi protein sequences were used to query the predicted *Diaphorina citri* protein sets (Diacit_International_psyllid_consortium_proteins_v1and Diacit_RefSeq_proteins_Release

_100) at i5k@NAL via the BLASTp tool. Loci encoding putative homologs were identified and manually annotated in Web Apollo using RNA-Seq data as evidence for transcript structure. NCBI databases were used for reciprocal blast analysis of the predicted protein. Multiple sequence alignments of insect homologs were performed using the ClustalW program (MEGA7 software). A neighbor-joining phylogenetic tree was constructed using full-length protein sequences in MEGA7.

Table 1: Number of Armitage homologs seen in related insect species

| Species | Armitage |
| --- | --- |
| *D. melanogaster* | 1 |
| *A. gambiae* | 1 |
| *A. aegypti* | 2 |
| *C. quinquefasciatus* | 2 |
| *D. citri* | 1 |

Table 2: Bit score, query coverage, identity and e-value results from BLAST analysis of predicted Armitage to insect Armitage homologs.

|  |  | ***D. melanogaster*** | ***A. gambiae*** | ***A. aegypti*** | ***C. quinquefasciatus*** |
| --- | --- | --- | --- | --- | --- |
| *D. citri* Armitage | **Bit score** | 289 | 247 | 266 | 158 |
|  | **QC (Identity)** | 51% (41%) | 52% (41%) | 54% (38%) | 43% (35%) |
|  | **E value** | 3.00E-81 | 1.00E-67 | 2.00E-74 | 2.00E-39 |

**Results and Conclusions**

BLASTp analysis of the *D. citri* genome revealed one putative Armi ortholog (Table 1).

We have named this gene *D. citri armitage* (*armitage*). Reciprocal BLAST analysis with the predicted *D. citri* Armi protein sequence to fly and mosquito genome databases confirm that *D. citri* Armi is orthologous to fly and mosquito Armi (Table 2). Phylogenetic analysis of insect Armi homologs show clustering of *Aedes aegypti* and *Culex quinquefasciatus* paralogs and *D. citri’s* Armi is positioned as the outgroup (Figure 1).

Figure 1: Phylogenetic tree of related insect Armitage proteins. Individual insect species are depicted with different colors. *D. citri* is marked with a taxon marker.


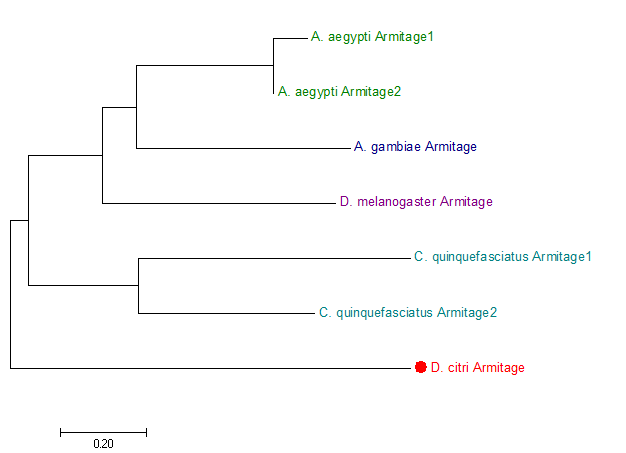


**References**

1. Cook, H. A., Koppetsch, B. S., Wu, J. and Theurkauf, W. E. (2004) 'The Drosophila SDE3 homolog armitage is required for oskar mRNA silencing and embryonic axis specification', *Cell* 116(6): 817-29.
2. Dalmay, T., Horsefield, R., Braunstein, T. H. and Baulcombe, D. C. (2001) 'SDE3 encodes an RNA helicase required for post-transcriptional gene silencing in Arabidopsis', *EMBO J* 20(8): 2069-78.
3. Haase, A. D., Fenoglio, S., Muerdter, F., Guzzardo, P. M., Czech, B., Pappin, D. J., Chen, C., Gordon, A. and Hannon, G. J. (2010) 'Probing the initiation and effector phases of the somatic piRNA pathway in Drosophila', *Genes Dev* 24(22): 2499-504.
4. Malone, C. D., Brennecke, J., Dus, M., Stark, A., McCombie, W. R., Sachidanandam, R. and Hannon, G. J. (2009) 'Specialized piRNA pathways act in germline and somatic tissues of the Drosophila ovary', *Cell* 137(3): 522-35.

# Supplementary Note 28: Fragile X Mental Retardation Protein

**Introduction**

Fragile X syndrome is a major cause of autism and mental retardation in humans. The mutation responsible for this syndrome is an expansion of a CGG trinucleotide repeat which suppresses expression of the *fragile X mental retardation 1* (FMR1) gene found on the X chromosome in humans. This loss of protein expression causes a loss of neuronal connectivity resulting in the syndrome’s symptoms (reviewed in ([Lin, 2015](#_ENREF_3))). In insects FMR1 was identified as playing a role in the RNAi response when it was identified as part of the RNA-induced silencing complex (RISC) in *Drosophila melanogaster* S2 cells ([Caudy et al., 2002](#_ENREF_1); [Ishizuka et al., 2002](#_ENREF_2)). This data indicating FMR1’s role in RISC has led to further investigations identifying FMR1’s role in gene expression regulation through the miRNA pathway (reviewed in ([Lin, 2015](#_ENREF_3)).

**Methods**

FMR1 sequences were used to query the predicted *Diaphorina citri* protein sets (Diacit_International_psyllid_consortium_proteins_v1and Diacit_RefSeq_proteins_Release

_100) at i5k@NAL via the BLASTp tool. Loci encoding putative homologs were identified and manually annotated in Web Apollo using RNA-Seq data as evidence for transcript structure. EMBL EBI’s online InterPro program was used for domain identification. NCBI databases were used for reciprocal blast analysis of the predicted protein. Multiple sequence alignments of insect homologs were performed using the ClustalW program (MEGA7 software). A neighbor-joining phylogenetic tree was constructed using full-length protein sequences in MEGA7.

Table 1: Number of FMR1 homologs in insect species

|  | **FMR1** |
| --- | --- |
| *D. melanogaster* | 1 |
| *A. gambiae* | 0 |
| *A. aegypti* | 1 |
| *C. quinquefasciatus* | 1 |
| *D. citri* | 1 |

Table 2: Bit score, query coverage, identity and e-value results from BLAST analysis of predicted FMR1 to FMR1 insect orthologs.

|  |  | ***D. melanogaster*** | ***A. gambiae*** | ***A. aegypti*** | ***C. quinquefasciatus*** |
| --- | --- | --- | --- | --- | --- |
| *D. citri* FMR1 | **Bit score** | 202 | No significant hits | 245 | 234 |
|  | **QC (Identity)** | 54% (44%) |  | 50% (50%) | 54% (49%) |
|  | **E value** | 3.00E-57 |  | 2.00E-73 | 2.00E-69 |

**Results and Conclusions**

BLASTp analysis indicates that *D. citri* has one *fragile x mental retardation* ortholog (Table 1 and 2). Following conventional nomenclature, we have named this gene *D. citri* *FMR1* (*FMR1*). RNA-Seq data suggests that alternative splicing does occur at this loci and multiple isoforms for this gene probably exist. This is not a surprising result as at least 9 isoforms of *FMR1* have been identified in *Drosophila*. As currently annotated the *D. citri* FMR1 protein has two Agenet-like domains and a K homology domain but does not contain the Fragile X related 1 protein C terminal core as seen in flies and mosquitoes.

Whether this domain is missing due to inaccurate annotation or true loss is unknown. Phylogenetic analysis shows expected relationships between fly, mosquito and psyllid FMR1 proteins (Figure 1).

Figure 1: Phylogenetic tree of insect FMR1 homologs. Each species is depicted with a different color. *D. citri* is marked with a taxon marker.


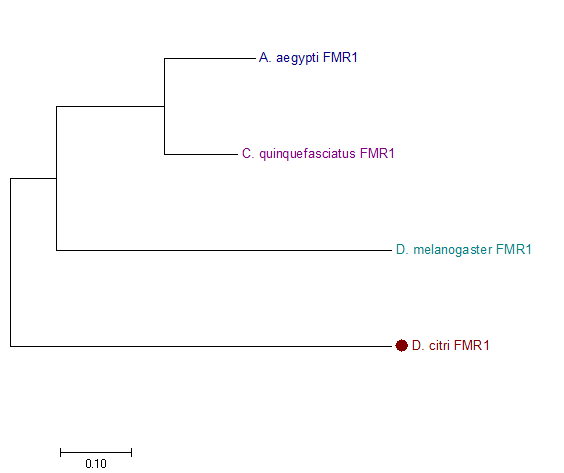


**References**

1. Caudy, A. A., Myers, M., Hannon, G. J. and Hammond, S. M. (2002) 'Fragile X-related protein and VIG associate with the RNA interference machinery', Genes Dev 16(19): 2491-6.
2. Ishizuka, A., Siomi, M. C. and Siomi, H. (2002) 'A Drosophila fragile X protein interacts with components of RNAi and ribosomal proteins', Genes Dev 16(19): 2497-508.
3. Lin, S. L. (2015) 'microRNAs and Fragile X Syndrome', Adv Exp Med Biol 888: 107-21.

# Supplementary Note 29: Spindle_E (homeless)

**Introduction**

Spindle-E (homeless) mutations were first characterized by the role the protein product played in fertility in *Drosophila melanogaster* ([Stapleton et al., 2001](#_ENREF_14); [Aravin et al., 2004](#_ENREF_1)). Soon after this work was preformed *spindle-E* was identified as having a role in RNA silencing pathways when *spindle-E* mutants were shown to prevent RNAi activation during egg maturation and prevent translation control during oogenesis ([Kennerdell et al., 2002](#_ENREF_8)). Later, *spindle-E* mutants were shown to be defective in heterochromatic silencing indicating *spindle-E* has a role in transcriptional silencing in germline cells ([Pal-Bhadra et al., 2004](#_ENREF_12)).

**Methods**

Spindle-E protein sequences were used to query the predicted *Diaphorina citri* protein sets (Diacit_International_psyllid_consortium_proteins_v1and Diacit_RefSeq_proteins_Release

_100) at i5k@NAL via the BLASTp tool. Loci encoding putative homologs were identified and manually annotated in Web Apollo using RNA-Seq data as evidence for transcript structure. NCBI databases were used for reciprocal blast analysis of predicted proteins. Multiple sequence alignments of insect homologs were performed using the ClustalW program (MEGA7 software). A neighbor-joining phylogenetic tree was constructed using full-length protein sequences in MEGA7.

Table 1: Number of Spindle-E homologs in insect species

| **Species** | **Spindle_E** |
| --- | --- |
| ***D. melanogaster*** | 1 |
| ***A. gambiae*** | 1 |
| ***A. aegypti*** | 1 |
| ***C. quinquefasciatus*** | 1 |
| ***D. citri*** | 2 |

Table 2: Bit score, query coverage, identity and e-value results from BLAST analysis of predicted Spindle-E1 and Spindle-E2 to insect Spindle-E homologs.

|  |  | ***D. melanogaster*** | ***A. gambiae*** | ***A. aegypti*** | **C. quinquefasciatus** |
| --- | --- | --- | --- | --- | --- |
| *D. citri* Spindle-E1 | **Bit score** | 242 | 243 | 250 | 244 |
|  | **QC (Identity)** | 54% (52%) | 89% (49%) | 60% (52%) | 80% (50%) |
|  | **E value** | 7.00E-68 | 5.00E-68 | 2.00E-70 | 2.00E-68 |
| *D. citri* Spindle-E2 | **Bit score** | 175 | 158 | 161 | 171 |
|  | **QC (Identity)** | 26% (36%) | 72% (33%) | 74% (33%) | 78% (33%) |
|  | **E value** | 2.00E-47 | 4.00E-39 | 3.00E-40 | 3.00E-45 |

**Results and Conclusions**

BLASTp analysis of the *D. citri* genome has identified at least two Spindle-E orthologs (Table 1). These two genes have been named *D. citri spindle-E1* (*spindle-E1*) and *D. citri spindle-E2* (*spindle-E2*). Reciprocal BLAST analysis confirms these putative orthologs (Table 2) and phylogenetic analysis shows appropriate clustering of insect Spindle-E proteins (Figure 1). In the case of *spindle-E2* RNA-Seq data suggests alternative splicing may occur producing a variety of different isoforms.

Putative isoforms have been named *spindle-E2 RA*, *spindle-E2* *RB*, *spindle-E2 RC* and *spindle-E2* *RD*. *spindle-E2 RA* was used as the representative isoform for BLAST and phylogenetic analysis.

Figure 1: Phylogenetic tree of related insect Spindle-E proteins. Individual insect species are depicted with different colors. *D. citri* is marked with a taxon marker.


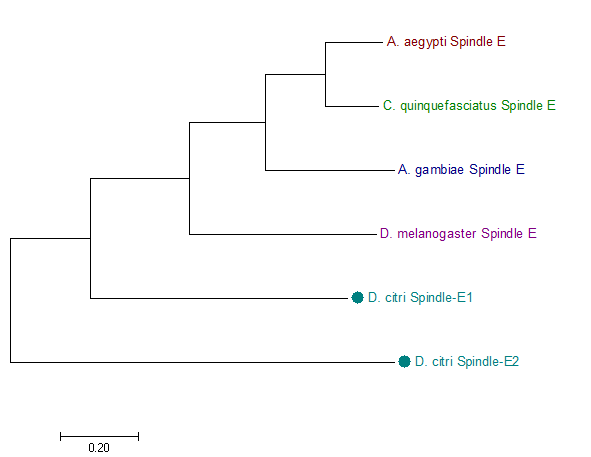


**References**

1. Aravin, A. A., Klenov, M. S., Vagin, V. V., Bantignies, F., Cavalli, G. and Gvozdev, V. A. (2004) 'Dissection of a natural RNA silencing process in the Drosophila melanogaster germ line', *Mol Cell Biol* 24(15): 6742-50.
2. Kennerdell, J. R., Yamaguchi, S. and Carthew, R. W. (2002) 'RNAi is activated during Drosophila oocyte maturation in a manner dependent on aubergine and spindle-E', *Genes Dev* 16(15): 1884-9.
3. Pal-Bhadra, M., Leibovitch, B. A., Gandhi, S. G., Chikka, M. R., Bhadra, U., Birchler, J. A. and Elgin, S. C. (2004) 'Heterochromatic silencing and HP1 localization in Drosophila are dependent on the RNAi machinery', *Science* 303(5658): 669-72.
4. Stapleton, W., Das, S. and McKee, B. D. (2001) 'A role of the Drosophila homeless gene in repression of Stellate in male meiosis', *Chromosoma* 110(3): 228-40.

# Supplementary Note 30: Rm62 protein

**Introduction**

Rm62 (also known as Lip) is the ortholog of human p68. It is an RNA helicase which has been shown to be involved in the RNA interference (RNAi) pathway ([Huang and Liu, 2002](#_ENREF_2); [Ishizuka et al., 2002](#_ENREF_3)), antiviral defense ([Zambon et al., 2006](#_ENREF_5)) and in retrotransponson maintenance in *Drosophila melanogaster* ([Lei and Corces, 2006](#_ENREF_4)). While only Rm62 has been linked to RNAi in *Drosophila* six other RNA helicase genes have also been identified (NM165103, NM141510, NM1401752, NM132792, NM139805, NM132196) ([Campbell et al., 2008](#_ENREF_1)). Furthermore, in the mosquito species *Anopheles gambiae*, *Aedes aegypti* and *Culex quinquefasciatus* six, nine and ten Rm62-like genes have been respectively identified and expression has been confirmed for many of these genes ([Campbell et al., 2008](#_ENREF_1)).

**Methods**

Rm62 sequences were used to query the predicted *Diaphorina citri* protein sets (Diacit_International_psyllid_consortium_proteins_v1and Diacit_RefSeq_proteins_Release

_100) at i5k@NAL via the BLASTp tool. Loci encoding putative homologs were identified and manually annotated in Web Apollo using RNA-Seq data as evidence for transcript structure. NCBI databases were used for reciprocal blast analysis of the predicted protein. Multiple sequence alignments of insect homologs were performed using the ClustalW program (MEGA7 software). A neighbor-joining phylogenetic tree was constructed using full-length protein sequences in MEGA7.

Table 1: Number of Rm62 homologs in insect species. Note: While there is only 1 Rm62 in *Drosophila* there are 6 more Rm62 like proteins. Therefore, the mosquito homologs listed here are not necessarily orthologous to Rm62 but could be orthologs of other *Drosophila* RNA helicases.

|  | **Rm62** |
| --- | --- |
| *D. melanogaster* | 1 |
| *A. gambiae* | 6 |
| *A. aegypti* | 9 |
| *C. quinquefasciatus* | 10 |
| *D. citri* | 1 |

Table 2: Bit score, query coverage, identity and e-value results from BLAST analysis of predicted Rm62 to Rm62 insect homologs. For the mosquito species the top BLAST hit has been identified in the Bit Score column.

|  |  | ***D. melanogaster*** | ***A. gambiae*** | ***A. aegypti*** | ***C. quinquefasciatus*** |
| --- | --- | --- | --- | --- | --- |
| *D. citri* Rm62 | **Bit score** | 516 | 506 (Rm62A) | 505 (Rm62C) | 506 (Rm62H) |
|  | **QC (Identity)** | 78% (58%) | 81% (56%) | 92% (57%) | 83% (56%) |
|  | **E value** | 8.00E-180 | 6.00E-174 | 1.00E-175 | 2.00E-174 |

**Results and Conclusions**

The RNA helicase family in Diptera is clearly a large, complex family. Because Rm62 is currently the only RNA helicase in this family known to be involved in RNAi we limited our search of the *D. citri* genome to orthologs of this particular gene. We found one putative ortholog to Rm62 which we have named *D. citri Rm62* (*Rm62*) and included in our analysis (Table 1). It is important to note, however, that other RNA helicase family members could also exist in the *D. citri* genome which have not been annotated. Reciprocal BLASTp analysis of Rm62 indicates that this gene is orthologous to *Drosophila’s* Rm62. However, the top BLASTp hits to mosquito databases suggest that this gene may be more closely related to *A. gambiae’s* Rm62A, *A. aegypti’s* Rm62C and *C. quinquefasciatus’* Rm62H than to other mosquito *Rm62* genes (Table 2).

Figure 1: Phylogenetic tree of insect Rm62 homologs. Each species is depicted with a different color. *D. citri* is marked with a circular taxon marker. Proteins that received the top BLAST score on a reciprocal BLAST analysis are marked with a diamond.


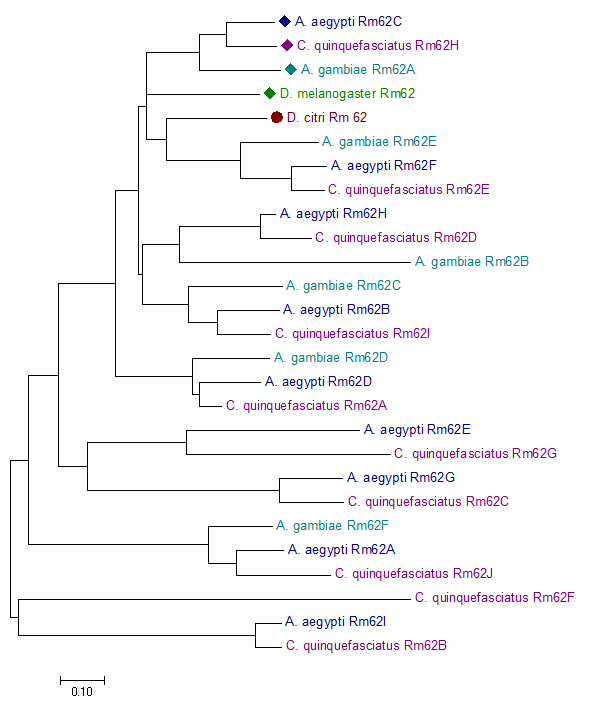


Exact relationships between these RNA helicase family members is difficult to determine as bootstrap scores at some nodes are quite low. However, Rm62, *Drosophila* Rm62 and mosquito top BLASTp hits do fall into the same clade (top reciprocal BLAST hits are depicted by the colored diamonds) (Figure 1).

**References**

1. Campbell, C. L., Black, W. C. th, Hess, A. M. and Foy, B. D. (2008) 'Comparative genomics of small RNA regulatory pathway components in vector mosquitoes', *BMC Genomics* 9: 425.
2. Huang, Y. and Liu, Z. R. (2002) 'The ATPase, RNA unwinding, and RNA binding activities of recombinant p68 RNA helicase', *J Biol Chem* 277(15): 12810-5.
3. Ishizuka, A., Siomi, M. C. and Siomi, H. (2002) 'A Drosophila fragile X protein interacts with components of RNAi and ribosomal proteins', *Genes Dev* 16(19): 2497-508.
4. Lei, E. P. and Corces, V. G. (2006) 'RNA interference machinery influences the nuclear organization of a chromatin insulator', *Nat Genet* 38(8): 936-41.
5. Zambon, R. A., Vakharia, V. N. and Wu, L. P. (2006) 'RNAi is an antiviral immune response against a dsRNA virus in Drosophila melanogaster', *Cell Microbiol* 8(5): 880-9.

# Supplementary Note 31: Ras-related nuclear protein

**Introduction**

Ran (Ras-related nuclear protein) is a GTPase which regulates many cellular functions related to the cell cycle, including spindle assembly and nuclear membrane assembly (reviewed in ([Chen et al., 2015](#_ENREF_2)). In addition to its role in the cell cycle Ran has been implicated in the nucleocytoplasmic transport of various molecules ([Shibata et al., 2006](#_ENREF_4)). With respect to RNA silencing Ran plays an essential role in miRNA biogenesis as Exportin-5 functions to transport primary miRNA precursors from the nucleus to the cytoplasm in a Ran-dependent manner ([Yi et al., 2003](#_ENREF_6); [Bohnsack et al., 2004](#_ENREF_1); [Lund et al., 2004](#_ENREF_3); [Wang et al., 2011](#_ENREF_5))

**Methods**

Ran sequences were used to query the predicted *Diaphorina citri* protein sets (Diacit_International_psyllid_consortium_proteins_v1and Diacit_RefSeq_proteins_Release

_100) at i5k@NAL via the BLASTp tool. Loci encoding putative homologs were identified and manually annotated in Web Apollo using RNA-Seq data as evidence for transcript structure. EMBL EBI’s online InterPro program was used for domain identification. NCBI databases were used for reciprocal blast analysis of the predicted protein. Multiple sequence alignments of insect homologs were performed using the ClustalW program (MEGA7 software). A neighbor-joining phylogenetic tree was constructed using full-length protein sequences in MEGA7.

Table 1: Number of Ran homologs in insect species

|  | **Ran** |
| --- | --- |
| *D. melanogaster* | 1 |
| *A. gambiae* | 1 |
| *A. aegypti* | 1 |
| *C. quinquefasciatus* | 1 |
| *D. citri* | 1 |

Table 2: Bit score, query coverage, identity and e-value results from BLAST analysis of predicted Dcitr_Ran to Ran insect orthologs.

|  |  | ***D. melanogaster*** | ***A. gambiae*** | ***A. aegypti*** | ***C. quinquefasciatus*** |
| --- | --- | --- | --- | --- | --- |
| *D. citri* Ran | **Bit score** | 418 | 424 | 423 | 423 |
|  | **QC (Identity)** | 100% (91%) | 99% (93%) | 99% (93%) | 99% (93%) |
|  | **E value** | 5.00E-151 | 7.00E-154 | 2.00E-153 | 2.00E-153 |

**Results and Conclusions**

BLASTp analysis confirms the *D. citri* genome contains one Ran GTPase orthologous to *Drosophila* Ran (Table 1). We have named this gene *D. citri ran* (*ran*). Reciprocal BLASTp analysis to fly and mosquito databases show orthologous Ran proteins to be the only significant hit to Ran (Table 2) and domain analysis confirms *D. citri* Ran as a Ran GTPase belonging to the small GTPase superfamily. Phylogenetic analysis shows expected relationships between fly, mosquito and psyllid Ran proteins (Figure 1).

Figure 1: Phylogenetic tree of insect Ran homologs. Each species is depicted with a different color. *D. citri* is marked with a taxon marker.


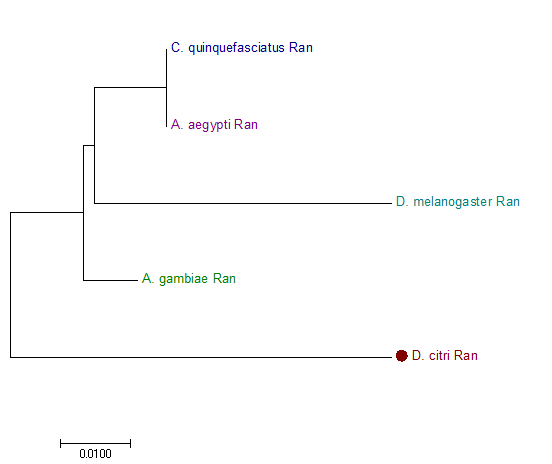


**References**

1. Bohnsack, M. T., Czaplinski, K. and Gorlich, D. (2004) 'Exportin 5 is a RanGTP-dependent dsRNA-binding protein that mediates nuclear export of pre-miRNAs', *RNA* 10(2): 185-91.
2. Chen, J. W., Barker, A. R. and Wakefield, J. G. (2015) 'The Ran Pathway in Drosophila melanogaster Mitosis', *Front Cell Dev Biol* 3: 74.
3. Lund, E., Guttinger, S., Calado, A., Dahlberg, J. E. and Kutay, U. (2004) 'Nuclear export of microRNA precursors', *Science* 303(5654): 95-8.
4. Shibata, S., Sasaki, M., Miki, T., Shimamoto, A., Furuichi, Y., Katahira, J. and Yoneda, Y. (2006) 'Exportin-5 orthologues are functionally divergent among species', *Nucleic Acids Res* 34(17): 4711-21.
5. Wang, X., Xu, X., Ma, Z., Huo, Y., Xiao, Z., Li, Y. and Wang, Y. (2011) 'Dynamic mechanisms for pre-miRNA binding and export by Exportin-5', *RNA* 17(8): 1511-28.
6. Yi, R., Qin, Y., Macara, I. G. and Cullen, B. R. (2003) 'Exportin-5 mediates the nuclear export of pre-microRNAs and short hairpin RNAs', *Genes Dev* 17(24): 3011-6.

# Supplementary Note 32: Glycolysis pathway

**Introduction**

The glycolysis pathway for three carbon compounds is an essential pathway for eukaryotic life. We have attempted to annotate 15 genes involved in this pathway in the Asian citrus psyllid (*Diaphorina citri*), an agrarian pest species, by comparison to the genome of a model insect species: *Drosophila melanogaster*.

**Methods**

Gene Identification

A list of genes associated with glycolysis in *D. melanogaster* were gathered using the Kyoto Encyclopedia of Genes and Genomes (KEGG) pathway database (http://www.genome.jp/kegg/) (2, 3). The sequences for these genes were downloaded from FlyBase (1), and the putative homologs in *D. citri* were identified by BLASTing these sequences against the transcriptome of *D. citri* using CLC Genomics Workbench 7.0 **(https://www.qiagenbioinformatics.com), using E-value to determine best matches; genes with non-unique matches were assigned unique matches based on next lowest E-value.**

Gene Annotation

Gene annotations were performed using the JBrowse/Web Apollo *Diaphorina citri* on the i5k Workspace@NAL (http://i5k.nal.usda.gov/) (4). A search was performed for each gene sequence and gene models were created from various evidence tracks. The sequence of each newly created model was BLASTed at NCBI to check for congruence.

Table 1: List of genes involved in the core module of glycolysis involving three-carbon compounds (2, 3)

Table 2: Results from CLC Genomics Workspace 7.0 BLAST of D. melanogaster genes against D. citri RNA.

| **Query** | **Lowest E-value** | **Accession (E-value)** | **Next Best Match** | **Resulting E-value** |
| --- | --- | --- | --- | --- |
| FBgn0250906 | 0.00 | XM_008475651 | - | - |
| FBgn0001092 | 0.00 | XM_008481620 | - | - |
| FBgn0000579 | 0.00 | XM_008483244 | - | - |
| FBgn0001091 | 1.01E-175 | XM_008481620 | XM_08481619 | 1.01E-175 |
| FBgn0031451 | 3.76E-145 | XM_008475651 | XM_008475652 | 2.33E-143 |
| FBgn0034173 | 2.76E-138 | XM_008481620 | XM_008474604 | 3.79E-138 |
| FBgn0267385 | 1.63E-119 | XM_008482433 | - | - |
| FBgn0031462 | 7.46E-79 | XM_008482433 | XM_008476979 | 2.47E-69 |
| FBgn0014869 | 1.67E-69 | XM_008489682 | - | - |
| FBgn0011270 | 5.23E-68 | XM_008474451 | - | - |
| FBgn0038952 | 6.55E-67 | XM_008482433 | XM_008482434 | 7.84E-49 |
| FBgn0086355 | 3.66E-62 | XM_008476234 | - | - |
| FBgn0038258 | 1.20E-47 | XM_008482433 | XM_008483412 | 3.17E-4 |
| FBgn0037115 | 1.23E-7 | XM_008482433 | XM_008479557 | 0.36 |
| FBgn0036723 | 2.05 | XM_008481206 | - | - |

**Table 3:** Results from BLAST at NCBI of gene model

**Results**

Out of fifteen genes identified as potential matches for the glycolysis pathway in *D. citri* only five unique best matches based on E-value were found, with one predicted gene (XM_008482433) being the best match for five genes in the pathway. After reassignment, twelve unique predicted genes had an E-value below E-10, which was used as an arbitrary cutoff. In all cases for the remaining 12 models the subsequent check using NCBI BLAST confirmed the model, though low Max Score/Total Score ration was observed in one case involving transcript variants.

**Discussion**

Despite being an integral pathway for metabolism, there was amount of variation in the length of similar sequences, with some *D. citri* sequences only matching a stretch totaling less than 1/8th of the query gene. There could be a case made against the use of solely E-value as a determinant of best matches in this case, especially considering the number of non-unique matches. The number of transcript variants and related proteins in this pathway was also no doubt a contributing factor to difficulty in distinguishing its related components.

**References**

1. Attrill H, Falls K, Goodman JL, Millburn GH, Antonazzo G, Rey AJ, Marygold SJ; the FlyBase Consortium. (2016) FlyBase: establishing a Gene Group resource for Drosophila melanogaster. Nucleic Acids Res. 44(D1):D786-D792.

2. Kanehisa, M. and Goto, S.; KEGG: Kyoto Encyclopedia of Genes and Genomes. Nucleic Acids Res. 28, 27-30 (2000).

3. Kanehisa, M., Sato, Y., Kawashima, M., Furumichi, M., and Tanabe, M.; KEGG as a reference resource for gene and protein annotation. Nucleic Acids Res. 44, D457-D462 (2016).

4. Poelchau M.,Childers C., Moore G. et al. (2015) The i5k Workspace@NAL–enabling genomic data access, visualization and curation of arthropod genomes. .,43, D714–D719.

# Supplementary Note 33: Aquaporin

**Introduction**

Aquaporins are integral membrane proteins primarily responsible for transporting water and, depending on the protein subset, small solutes across biological membranes^1-3^. A part of the major intrinsic protein (MIP) family, this subset of proteins has a common motif of 6 membrane-spanning elements, which form a selective pore allowing water or other molecules to pass through the cell membrane. Thirteen of these proteins have been identified in mammalian samples, and greater or fewer numbers in lower species including, plants, amphibians, insects, and bacteria^1,4^. Insects typically have 6-8 aquaporins^2,5^ with higher flies have 8-12 aquaporins^6^

**Methods and Results**

A quick GO term search of the FlyBase Drosophila melanogaster gene sets generated a list of eight genes with water transmembrane transporter activity proteins (flybase.org). Using the tblastx function in the CLC Genomics Workbench 9.0 software searching against a local Diaphorina citri RNA gene sets, eight aquaporin genes were identified that match those from D. melanogaster. The best matches of those genes, determined by E-value and bit scores (Table 1) were targeted for annotation using the WebApollo.

In cases where matches were uncertain or redundant, the next closest gene targets were annotated. Using the MAKER predicted gene set, the NCBI predicted gene set, and RNA-seq read mapping data, gene models were aligned and adjusted in the assembled *D. citri* genome. The final curated gene model was then confirmed using the NCBI BLAST search of the predicted protein.

For the most part, gene models were readily identified, and at least partially corrected with little issue. However, in the case of three closely related *D. melanogaster* proteins (see Table 1), psyllid AQP genes were difficult to pinpoint, were found to be closely related to more than one predicted gene, and/or difficult to correct. These incorrect models prevented the generation of a phylogenetic tree for *D. citri* AQPs. To determine correct AQP sequences the assembled MCOT was utilized to supplement the genome-based analyses. The combination of the MCOT and genome analyses revealed that there are eight unique aquaporin genes (Figure 1, Table 2) and provided the complete sequences for each of these genes. From this combinatory analysis, we were able to confirm that we have identified eight aquaporin genes that includes the typical water transporters (Drip, AQP2), AQP4/AQP5/Eglps (three sequences), AQP6 (unorthodox), and Bib. This number falls within the range of most insects (6-8)^2,5^.

Figure 1. Comparison of predicted aquaporins from *D. melanogaster* and *D. citri*. Neighbor-joining tree was produced using MEGA6 using Dayhoff Model and pairwise matching; branch values indicate support following 1500 bootstraps; values below 50% are omitted.


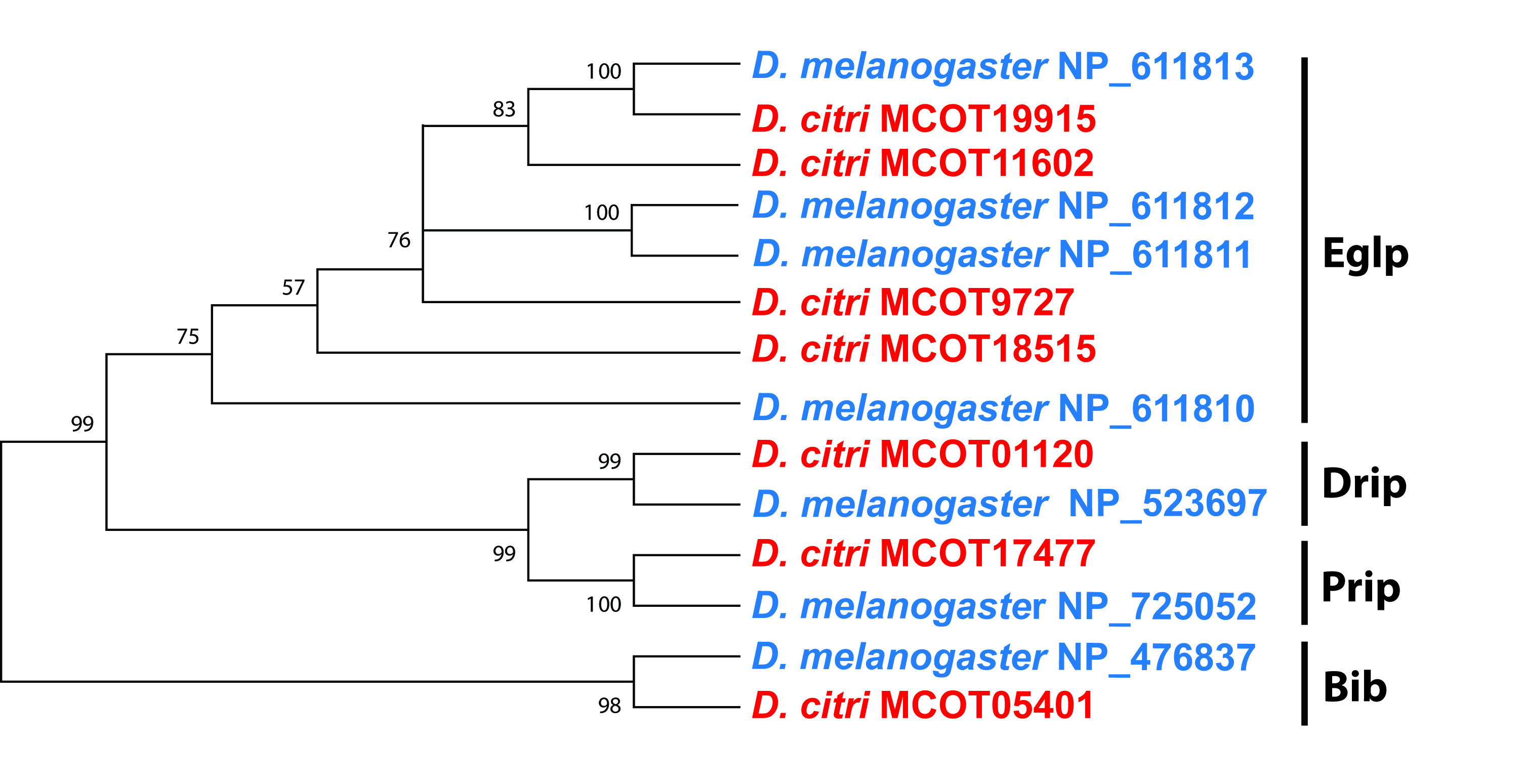


| Table 1: *D. citri* annotated gene list. Homologous blast search from eight *D. melagonaster* aquaporin genes (flybase.org) against *D. citri* genome were mostly successful in identifying genes to annotate. In cases marked with (*), the next closest match from the BLAST search was annotated. | | | |
| --- | --- | --- | --- |
|  |  |  |  |
| **mRNA:** | **Symbol (Predicted):** | **Scaffold:** | ***D. melanogaster*** |
| XM_008476342 | bib (big brain) | NW_007378164.1 | FBgn0000180 |
| XM_008471675 | Aquaporin AQPA.e_1 | NW_007377733.1 | FBgn0033635 |
| XM_008486010 | Aquaporin AQPAe.a_2 (drip) | NW_007381649.1 | FBgn0015872 |
| XM_008486171 | Aquaporin 12-like | NW_007381869.1 | FBgn0033807 |
| XM_008489052 | Aquaporin-4 like | NW_007459679.1 | FBgn0034882 |
| XM_008475207* | Aquaporin AQPAe.a-like* | NW_007378044.1* | FBgn0034885 |
| XM_008489051* | Aquaporin-2-like* | NW_007459679.1* | FBgn0034884 |
| XM_008483237* | Aquaporin-4 like* | NW_007379533.1* | FBgn0034883 |

Table 2. BLAST-based analyses of *D*. *citri* aquaporin genes compared to those from the fruit fly (*Drosophila* *melanogaster*), bed bug (*Cimex* *lectularius*), and pea aphid (*Acyrthosiphon* *pisum*).

**References**

1. Verkman, A.S. More than just water channels: unexpected cellular roles of aquaporins. *J Cell Sci* **118**, 3225-32 (2005).

2. Benoit, J.B. *et al.* Emerging roles of aquaporins in relation to the physiology of blood-feeding arthropods. *J Comp Physiol B* (2014).

3. Campbell, E.M., Ball, A., Hoppler, S. & Bowman, A.S. Invertebrate aquaporins: a review. *J Comp Physiol B* **178**, 935-55 (2008).

4. Verkman, A.S. *et al.* Water transport across mammalian cell membranes. *Am J Physiol* **270**, C12-30 (1996).

5. Finn, R.N., Chauvigne, F., Stavang, J.A., Belles, X. & Cerda, J. Insect glycerol transporters evolved by functional co-option and gene replacement. *Nat Commun* **6**(2015).

6. Benoit, J.B. *et al.* Aquaporins are critical for provision of water for lactation and progeny hydration to maintain tsetse fly reproductive success. . *PLoS Negl Trop Dis* **In press** (2014).

# Supplementary Note 34: Cathepsins and Cysteine Proteases

**Introduction**

Cysteine cathepsins are usually found in lysozymes where they are optimally active due to the slightly acidic environment (Turk et al, 2012). Cathepsins are found in vertebrates and invertebrates; in insects, cathepsins function in digestion, growth, and embryological development. There are several forms of cathepsins, designated by letters that can be divided into functional groups. Cathepsin B has its own group, cathepsins L, O, H, K, and S are in another group, and cathepsins W and F potentially make a third group (Wang et al, 1998). Normal organismal functioning depends on the different cathepsins; for example, cathepsin F is involved with MHC class II antigen presentation in macrophages (Turk et al, 2001). Cathepsin B has been attributed to several biological processes including apoptosis during metamorphosis, in embryogenesis with mobilization and degradation of yolk proteins, and digestion in the midgut (Kutsukake et al, 2004).

**Methods**

Cathepsin genes were collected from NCBI, i5k, Ensembl and FlyBase. BLAT searches were then performed at the i5k workspace to identify homologs in the ACP genome. Potential hits within the ACP genome were explored further using the WebApollo genome browser. NCBI Gnomon annotation gene models were manually annotated within WebApollo using BLAST and RNA-Seq evidence tracks. Completed gene models were analyzed by BlastP to confirm accuracy and completeness of the annotation.

Sequences from completed annotated genes were used to perform BlastP searches against related insects and model organisms. Pairwise amino acid sequence comparisons were performed between ACP, *Acyrthosiphon pisum* and *Drosophila melanogaster.* Collected orthologs and annotated ACP Cathepsin B protein sequences were used to generate a multiple sequence alignment using ClustalW in MEGA6. Sequences were chosen to identify close relationships to *A. pisum*. The ACP proteins were numbered in the order found. A neighbor joining phylogenetic tree was produced from the multiple sequence alignment to analyze the evolutionary relationships between the ACP cathepsins and well characterized sequences. (Figure 1) The unrooted Neighbor-Joining phylogenetic tree was constructed with the MEGA6 program using the bootstrap method (with 1000 replications) as a test of phylogeny and Poisson correction as the substitution model. The analysis includes 53 amino acid sequences of cathepsin genes proposed by NCBI and Ensembl, 607 positions with the ambiguous positions removed for each sequence pair in the data set.

**Results and Discussion**

Within the Asian citrus psyllid genome, a total of 34 cathepsins and/or cysteine proteinase orthologs were found. (Table 1) Manual curation of NCBI Gnomon annotation gene models allowed for evidence-based gene associations. The NCBI gene models included several partial genes that included peptidase domains within Apollo. Areas flanking the partial models were investigated, but did not reveal the absent sequences. Annotation of full length cathepsin B genes contained 5-6 exons, as found by Rispe et al in 2008. Several *A. pisum* cathepsin B orthologs were identified in the ACP with 32-65% amino acid identity, and 71-100% query coverage. (Table 2) Two *D. melanogaster* cathepsin B orthologs were identified in the ACP with 31% to 69% amino acid identity. Cathepsin H, L, K, O, and W also have orthologs in ACP. Two clades were identified through phylogenetic analysis within the cathepsin B orthologs, B-1 and B-2.

The evolutionary process of gene duplication can contribute to development of new biological functions or phenotypic traits, and is important to study within the cathepsin family. There was research that previously found many cathepsin B-like genes in the pea aphid (Table 1), and 11 were found in the psyllid genome (Rispe et al, 2008). Pairwise alignments and multiple sequence alignments were created to analyze the evolutionary relationships between the cathepsin B’s, which showed a similar expansion from 2 B-like genes in most model organisms, to the 28 in the pea aphid (Figure 1). Cathepsin B and cathepsin F have undergone genetic expansion, including 11 cathepsin B and B like proteins, and 9 cathepsin F and F like proteins. This is similar to the pea aphid, whom was shown to have a genetic expansion of cathepsin B proteins likely due to their phloem feeding lifestyle. RNAi inhibition of psyllid cathepsin B is considered a potential approach to control psyllid vectored disease.

Table 1: Cathepsin gene copies. The numbers of named gene copies in *Diaphorina citri*, *Acyrthosiphon pisum*, *Drosophila melanogaster*, *Cimex lectularius*, *Anopheles gambiae*, *Aedes aegypti*, and *Culex quinquefasciatus*, and *Mus musculus*. The letters designate the type of cathepsin.

| **Cathepsin** | **B** | **F** | **H** | **L / L1** | **K** | **Other** |
| --- | --- | --- | --- | --- | --- | --- |
| ***D. citri*** | 11 | 9 | 1 | 5 | 0 | 8 |
| ***A. pisum*** | 28 | 1 | 0 | 2 | 1 | 8 |
| ***C. lectularius*** | 8 | 0 | 0 | 9 | 0 | 6 |
| ***D. melanogaster*** | 2 | 1 | 0 | 1 | 1 | 0 |
| ***A. gambiae*** | 6 | 2 | 0 | 2 | 0 | 4 |
| ***A. aegypti*** | 7 | 0 | 0 | 1 | 0 | 4 |
| ***C. quinquefasciatus*** | 5 | 0 | 0 | 1 | 0 | 2 |
| ***M. musculus*** | 2 | 2 | 2 | 2 | 1 | 21 |

Table 2: Cathepsin B sequence table. The protein sequences used for the phylogenetic analysis with their corresponding BLAST match results for specific species, with the bit scores followed by the coverage %, ID%, and accession numbers. The *D. citri* proteins are numbered in the order found. The A. pisum proteins were named according to their EST numbers. The D. melanogaster proteins were renamed from their accession number name.

| ***D. citri* Gene Named** | ***D. citri* AA Length** | ***A. pisum* BLAST Match Name and Bit Score** | ***BLAST Query Percent*** | ***BLAST ID Percent*** | ***A. pisum* Acc #** | ***D. melanogaster*  BLAST Match Name and Bit Score** | ***BLAST Query Percent*** | ***BLAST ID Percent*** | ***D. melanogaster* Acc #** |
| --- | --- | --- | --- | --- | --- | --- | --- | --- | --- |
| B Prot | 131 | B348 77.4 | 90 | 32 | NP001119608 | B Iso A 53.5 | 29 | 49 | NP572920 |
| B Prot 1 | 253 | B5880 133 | 84 | 34 | NP001119619 | B Iso A 105 | 82 | 31 | NP572920 |
| B Prot 1 Iso 2 | 344 | B348 229 | 99 | 38 | NP001119608 | B Iso A 126 | 96 | 37 | NP572920 |
| B Prot 2 | 180 | B348 240 | 97 | 65 | NP001119608 | B Iso A 256 | 98 | 69 | NP572920 |
| B-like iso X1 prot 3 | 399 | B348 355 | 91 | 55 | NP001119608 | B Iso A 340 | 91 | 57 | NP572920 |
| B-like iso X2 prot 4 | 399 | B348 356 | 91 | 55 | NP001119608 | B Iso A 345 | 91 | 57 | NP572920 |
| B-like cp 5 | 374 | B91418 231 | 71 | 42 | NP001119614 | B2 3074 211 | 69 | 31 | NP726176 |
| B-like cp 6 | 362 | B84 242 | 95 | 37 | NP001119618 | B Iso A 227 | 89 | 39 | NP572920 |
| B-like prot 7 part | 143 | B1852 123 | 100 | 40 | NP001119610 | B Iso A 116 | 99 | 40 | NP572920 |
| B-like prot 8 part | 110 | B3074 47.8 | 88 | 34 | DAA06100 | B Iso A 46.6 | 75 | 37 | NP572920 |
| B-like cp 4 prot 9 part | 90 | B10270 67 | 84 | 42 | NP001128401 | B Iso A 27.7 | 86 | 31 | NP572920 |

Hm B

Mm B

100

Aae B-9637

Agb B-10992

100

Dme B-10992

Dme B10992

100

93

Dc B-2

52

Ap B-348

63

Dc B-4

Dc B-3

100

59

Cl B-like

57

70

Cq B-2340

Aae B1-9642

99

Agb B-1

100

Agb B-2

Cq B-2908

85

Cq B-6023

Cq B-6022

98

Aae B2-7599

Aae B2-5312

99

100

91

47

44

Cl Cyst Prot B

Cl B-like isoform X2

80

Ap B-5880

Ap B-1874

84

Ap B-3483

Ap B-10270

100

9

Dc B-5

Of B-7979

Dc B-7

62

Nlugens B

Dc B-10

77

45

Ap B-1674

Ap B-1852

Ap B-84

Ap B-16A

Ap B-912

Ap B-3098

Ap B-6207

Ap B-16D1

Ap B-16D2

100

100

76

85

100

97

99

80

10

11

Ap B-3738

Ap B-1418

94

Dc B-6

Dc B-1

85

Dc B-8

Dc B-9

61

58

Ap B-2744

Of B-1454

Dme B2-3074b

Dme B2-3074a

100

Ap B2-3074

100

96

23

13

12

1

8

20

Mm B2-3074

Of B-4538

51

0.2

Cathepsin B

Cathepsin B-2

*A. pisum*

Cathepsin B

Expansion

*D. citri* &

*A. pisum*

Cathepsin B

Expansion

Figure 1: Phylogenetic analysis of *Diaphorina citri* cathepsin B family protein sequences

The unrooted Neighbor-Joining phylogenetic tree was constructed with the MEGA6 program using the bootstrap method (with 1000 replications) as a test of phylogeny and Poisson correction as the substitution model. The analysis includes 53 amino acid sequences with 607 positions in the data set of cathepsin genes proposed by NCBI. Bootstrap values are shown near the nodes. The *D. citri* proteins are numbered in the order found. The *A. pisum* proteins were named according to their EST numbers. *Cimex lectularius,* *Aedes aegypti, Culex quinquefasciatus* were renamed using the last four digits of their accession numbers, and *D. Melanoaster* proteins were renamed from their accession number name. Abbreviations: *Diaphorina citri* Dc; *Acyrthosiphon pisum* Ap; *Anopheles gambiae* Agb; *Drosophila melanogaster* Dme; *Cimex lectularius Cl;* *Aedes aegypti Aae;* *Culex quinquefasciatus* Cq; *Homo sapien* Hm; *Mus musculus* Mm; and *Nilaparvata lugens* Nlugens.

**References**

1. Claude Rispe, Mayako Kutsukake, Vincent Doublet, Sylvie Hudaverdian, Fabrice Legeai, Jean-Christophe Simon, Denis Tagu, and Takema Fukatsu Large Gene Family Expansion and Variable Selective Pressures for Cathepsin B in Aphids Mol Biol Evol (2008) 25 (1): 5-17. PMID: 17934209
2. Ferrara TFdS, Schneider VK, Kishi LT, Carmona AK, Alves MFM, Belasque-Júnior J, et al. (2015) Characterization of a Recombinant Cathepsin B-Like Cysteine Peptidase from Diaphorina citri Kuwayama (Hemiptera: Liviidae): A Putative Target for Control of Citrus Huanglongbing. PLoS ONE 10(12): e0145132. doi:10.1371/journal.pone.0145132
3. Kutsukake M, Shibao H, Nikoh N, et al. Venomous protease of aphid soldier for colony defense. Proceedings of the National Academy of Sciences of the United States of America. 2004;101(31):11338-11343. doi:10.1073/pnas.0402462101.
4. Vito Turk, Boris Turk, Dušan Turk. Lysosomal cysteine proteases: facts and opportunities. The EMBO Journal Sep 2001, 20 (17) 4629-4633; DOI: 10.1093/emboj/20.17.4629
5. Turk Vito, Stoka Veronika, Vasiljeva Olga, Renko Miha, Sun Tao, Turk Boris, Turk Dušan. Cysteine cathepsins: From structure, function and regulation to new frontiers. Biochimica et Biophysica Acta (BBA) - Proteins and Proteomics, Volume 1824, Issue 1, January 2012, Pages 68-88, ISSN 1570-9639, http://dx.doi.org/10.1016/j.bbapap.2011.10.002. (http://www.sciencedirect.com/science/article/pii/S1570963911002706)
6. Bruce Wang, Guo-Ping Shi, Pin Mei Yao, Zhenqiang Li, Harold A. Chapman, and Dieter Brömme. Human Cathepsin F: MOLECULAR CLONING, FUNCTIONAL EXPRESSION, TISSUE LOCALIZATION, AND ENZYMATIC CHARACTERIZATIONJ. Biol. Chem. 1998 273: 32000-. doi:10.1074/jbc.273.48.32000
7. Xiaoduan Fang, Huilin Lu, Gecheng Ouyang, Yulu Xia, Mingfang Guo, Weinan Wu. Effectiveness of to predatory mite species (Acari: Phytoseiidae) in controlling Diaphorina citri (Hemiptera: Lividae) The Florida Entomologist, [Internet] [cited 2015 Sept 10]; Vol. 96, No. 4 (December, 2013), pp. 1325-1333 Florida Entomological Society Stable URL: http://www.jstor.org/stable/23609169

# Supplementary Note 35: Early developmental genes (Gap and Pair Rule genes)

**Introduction**

Early developmental genes are responsible for determining anterior/posterior (A/P) and the dorsal/ventral (D/V) axis of the embryo. The establishment of the axes enables the embryo to undergo segmentation. Segmentastion, or the subdivision of the developing embryo into serially homologous units, is one of the hallmarks of arthropods development. Arthropod segmentation is best understood in the fly *D. melanogaster*. *D. melanogaster* is different from most arthropods in that all segments are formed from the early blastoderm and segments are formed simultaneously (so called long-germ developmental mode). In most other arthropods only the anterior segments are formed in a similar way to *D. melanogaster* with the posteria sections added one at a time or in pairs of two from cell material derived from a posterior growth zone (so called short-germ developmental mode). Insects, show a range of short to long germ development however segmentation mechanisms are not universally conserved and only little is known about the genetic patterning of the anterior segments in *D. citri,* hence the need to identify early development genes in *D. citri*.

**Methods**

The choice of early developmental genes to annotate was informed on the basis of GO term annotations in *D. melanogaster*. Protein sequences for developmental genes for *D. melanogaster* were obtained from http://flybase.org/ [[3](#_ENREF_3)]. Contig sequences were searched for homology to the selected protein sequences using tbastn. Gene models that aligned with the regions of highest homology identified by tbastn search were selected for further analysis. RNAseq mapped reads were compared with the gene models to determine the transcribed regions. The transcribed regions were used to determine protein sequences of the gene. Protein sequences were used in reciprocal blast (blastx NCBI) to confirm the homology of the orthologs. Gene models were edited to resolve conflicts between RNAseq, blastx and homology data. Many of these were partial models and some had frameshifts and/or internal stop codons. A list of most (but not all) of the sequences is provided in the Psyllid annotation workbook. *Diaphorina citri* gene models identified by MCOT generated from a comparison of maker and cufflinks models with de novo assemblies by Trinity and Oases were searched for homology to known *D. melanogaster* early development genes. MCOT Gene models that aligned with the regions of highest homology identified by tbastn search were selected for further analysis by reciprocal blast against model insect genomes.

**Results**

One of the main reasons for choosing to sequence the *Diaphorina citri* genome was to identify novel targets for biological control. For this reason, it was of special interest to analyse its developmental gene complement. As developmental genes are a potential targets for gene silencing by RNA interference (RNAi), which is emerging as a promising tool to control *D. citri* through targeting nymphy development pathways [[1](#_ENREF_1)]. In total, we annotated ~10 genes that are known, in other insects, to be involved in developmental processes. We identified a further ~15 gene models in MCOT data base that also mapped to proteins involved in early developmental processes in insects. These include both genes encoding transcription factors and members of signaling pathway. These genes are identified and named as distinct development genes in accordance with the nomenclature from *Drosophila melanogaster*. There are also numerous developmental gene fragments, incomplete and putative models/genes predicted from the assembly. The genes identified indicate that *D. citri* utilizes conical insect developmental pathway, including Hox genes, and can be expected to contain components require to establish a normal anterior/posterior axis pattern. Normally the number of early developmental genes does not vary in arthropod species due to the conserved nature of development in this order [[2](#_ENREF_2)]. In confirmation of this the early patterning genes of *D. citri,* appear mainly conserved relative to what is known from other insects. No gene duplication in the early development genes was observed. However a number of gap and pair rule genes know from *Drosophila* were not found in *D. citri* however we conclude that the absence from the genome is evidence of the incomplete coverage of the sequencing effort, rather than a true absence.

| **Genes** | **Species** | |
| --- | --- | --- |
|  | *D. melanogaster* | *D. citri* |
| *crocodile* | **✓** | **✓** |
| *knirps* | **✓** | **✓** |
| *even-skipped* | **✓** | **✓** |
| *hairy* | **✓** | **✓** |
| *odd-paired* | **✓** | **✓** |
| *runt* | **✓** | **✓** |
| *runxA/lozenge* | **✓** | **✓** |
| *runxB* | **✓** | **✓** |
| *antennapedia* | **✓** | **✓** |
| *sex combs reduced* | **✓** | **✓** |

**Table 1** Presence/absence of early patterning genes in the genomes of Hemiptera *Drosophila melanogaster* and *Diaphorina citri*

| ***D. citri* Gap Genes** | **LOCATION [Accession #]** | **Protein Length** | **Domains** |
| --- | --- | --- | --- |
| crocodile | gi\|645504644\|ref\|NW_007378308.1  [XP_008475713.1] | 322 aa | FH Superfamily |
| knirps | gi\|645504350\|ref\|NW_007378383.1  [XP_008476344.1] | 344 aa | zinc finger C4 |
| ***D. citri* Pair Rule Genes** | **LOCATION [Accession #]** | **Protein Length** | **Domains** |
| even-skipped | gi\|645499728\|ref\|NW_007379481.1  [XP_008481312.1] | 165 aa |  |
| hairy | gi\|645499404\|ref\|NW_007379550.1  [XP_008481515.1] | 297 aa | HLH Superfamily  Hairy-orange Superfamily |
| odd-paired | gi\|645497036\|ref\|NW_007380134.1  [XP_008482718.1] | 316 aa | zinc finger M2C2 |
| runt | gi\|645490800\|ref\|NW_007381688.1  [XP_008484261.1] | 155 aa | runt superfamily |
| runxA/lozenge | gi\|645501180\|ref\|NW_007379113.1  [XP_008480084.1] | 258 aa | runt superfamily |
| runxB | gi\|645501995\|ref\|NW_007378890.1  [XP_008479194.1] | 130 aa | runt superfamily |
| ***D. citri* Hox Genes** | **LOCATION [Accession #]** | **Protein Length** | **Domains** |
| antennapedia | gi\|645505921\|ref\|NW_007378005.1  [XP_008481317.1] | 317 aa | Homeobox |
| sex combs reduced | gi\|645504043\|ref\|NW_007378448.1.1  [XP_008476811.1] | 313 aa | Homeobox |

**Table 2.** Current Early Developmental Genes identified in the *D. citri* genome.

The table lists Gap Genes, Pair Rule Genes and Hox Genes models, model location and accession number, protein length, and protein domain identified in the model

| ***Diaphorina citri*** | **crocodile**  **QC (ID)  Bit Score** | **knirps**  **QC (ID)  Bit Score** | **even skipped**  **QC (ID)  E-value** | **hairy**  **QC (ID)  E-value** | **odd paired**  **QC (ID)  Bit Score** |
| --- | --- | --- | --- | --- | --- |
| ***Acyrthosiphon pisum*** | 64% (63%)  246 | 31%  (87%)  207 | 35%  (95%)  52 | 100%  (45%)  192 | 37%  (76%)  202 |
| ***Halyomorpha halys*** | 79%  (55%)  233 | 43%  (74%)  216 | 26%  (51%)  47 | 62%  (47%)  160 | 32%  (44%)  98 |
| ***Cimex lectularius*** | 97% (50%)  248 | 36%  (80%)  204 | 29%  (52%)  51 | 61%  (48%)  144 | 37%  (81%)  206 |
| ***Anopheles gambiae*** | 67%  (54%)  227 | 28%  (94%)  202 | 20%  (83%)  60 | 73%  (50%)  195 | 37%  (66%)  209 |
| ***Aedes aegypti*** | 94%  (74%)  244 | 40%  (73%)  199 | 20%  (83%)  60 | 100%  (45%)  232 | 93%  (44%)  230 |
| ***Drosophila melanogaster*** | 69%  (56%)  231 | 27%  (86%)  196 | 20%  (93%)  60 | 100%  (40%)  170 | 40%  (73%)  209 |

| ***Diaphorina citri*** | **runt**  **QC (ID)  Bit Score** | **RunxA**  **QC (ID)  E-value** | **RunxB**  **QC (ID)  E-value** | **antennapedia QC (ID)  E-value** | **sex combs reduced**  **QC (ID)  E-value** |
| --- | --- | --- | --- | --- | --- |
| ***Acyrthosiphon pisum*** | 100%  (66%)  211 | 62%  (82%)  284 | 89%  (84%)  206 | 89%  (80%)  190 | 94%  (81%)  68 |
| ***Halyomorpha halys*** | 100%  (70%)  233 | 55%  (89%)  274 | 78%  (85%)  191 | 100%  67%  326 | 97%  (46%)  89 |
| ***Cimex lectularius*** | 92%  (78%)  245 | 58%  (89%)  292 | 82%  (83%)  198 | 100%  (66%)  322 | 94%  (51%)  104 |
| ***Anopheles gambiae*** | 99%  (72%)  243 | 48%  (80%)  233 | 81%  (67%)  154 | 99%  (47%)  219 | 57%  (81%)  54 |
| ***Aedes aegypti*** | 89%  (73%)  220 | 59%  (87%)  283 | 90%  (76%)  189 | 99%  (48%)  218 | 57%  (81%)  53 |
| ***Drosophila melanogaster*** | 94%  (75%)  244 | 64%  (80%)  293 | 82%  (82%)  194 | 95%  (61%)  218 | 25%  (58%)  50 |

**Table 3**. Represents Query Coverage (Identity) and E-value of the annotated gene models pairwise aligned to orthologues in other species. Pairwise alignment was performed using NCBI blast.

| ***D. citri* Genes** | **MCOT Model Number** |
| --- | --- |
| cap ‘n’ collar | MCOT01547.1.CT |
| caudal | MCOT08625.0.OO |
| collier/knot | MCOT22397.0.CO |
| crocodile | MCOT13724.0.CC |
| empty spiracles | MCOT04329.0.CO |
| hunchback | MCOT05092.1.CC |
| kruppel | MCOT03100.0.CO |
| orthodenticle | MCOT05861.1.CO |
| sloppy paired | MCOT19895.0.CT |
| tailless | MCOT23139.2.CC |
| even skipped | MCOT05103.0.CT |
| hairy | MCOT00149.0.CT |
| odd paired | MCOT12397.2.CT |
| runt | MCOT14329.0.CT |

**Table 4.** Current Early Developmental Genes identified in the *D. citri* MCOT database. The table lists Gap Genes, and Pair Rule Genes model number

Gap Genes

| ***Diaphorina citri*** | **cap ‘n’ collar**  **QC (ID)  E-value** | **caudal**  **QC (ID)  E-value** | **collier**  **QC (ID)  E-value** | **crocodile**  **QC (ID)  E-value** | **empty spiracles**  **QC (ID)  Bit Score** |
| --- | --- | --- | --- | --- | --- |
| ***Acyrthosiphon pisum*** | 39%  (63%)  234 | 43%  (71%)  141 | 51%  (77%)  471 | 61%  (62%)  244 | 63%  (79%)  238 |
| ***Halyomorpha halys*** | 66%  (44%)  381 | 90%  (49%)  154 | 82%  (87%)  797 | 76%  (55%)  233 | 46%  (87%)  194 |
| ***Cimex lectularius*** | 66%  (43%)  417 | 79%  (58%)  162 | 82%  (87%)  792 | 93%  (49%)  248 | 60%  (75%)  214 |
| ***Anopheles gambiae*** | 24%  (72%)  230 | 54%  (71%)  169 | 16%  (78%)  36 | 51%  (68%)  223 | 30%  (79)  174 |
| ***Aedes aegypti*** | 35%  (44%)  249 | 61%  (66%)  168 | 82%  (83%)  792 | 89%  (46%)  239 | 47%  (57%)  177 |
| ***Drosophila melanogaster*** | 23%  (66%)  207 | 37%  (76%)  134 | 82%  (84%)  798 | 65%  (57%)  230 | 46%  (60%)  183 |

| ***Diaphorina citri*** | **hunchback**  **QC (ID)  E-value** | **kruppel**  **QC (ID)  E-value** | **orthodenticle**  **QC (ID)  E-value** | **sloppy paired**  **QC (ID)  Bit Score** | **tailless**  **QC (ID)  E-value** |
| --- | --- | --- | --- | --- | --- |
| ***Acyrthosiphon pisum*** | 15%  (51%)  148 | 27%  (67%)  164 | 22%  (89%)  116 | 20%  (76%)  147 | 95%  (42%)  263 |
| ***Halyomorpha halys*** | 17%  (58%)  187 | 50%  (48%)  223 | 49%  (65%)  172 | 77%  (51%)  187 | 94%  (51%)  338 |
| ***Cimex lectularius*** | 18%  (53%)  184 | 51%  (57%)  265 | 44%  (72%)  171 | 82%  (51%)  208 | 98%  (51%)  360 |
| ***Anopheles gambiae*** | 16%  (55%)  174 | 56%  (51%)  265 | 32%  (66%)  129 | 41%  (69%)  174 | 82% (78%)  201 |
| ***Aedes aegypti*** | 15%  (51%)  148 | 57%  (59%)  279 | 23%  (89%)  133 | 41%  (69%)  174 | 95%  (44%)  295 |
| ***Drosophila melanogaster*** | 16%  (55%)  187 | 53%  (56%)  271 | 30%  (76%)  135 | 42%  (73%)  210 | 95%  (42%)  298 |

Pair Rule Genes

| ***Diaphorina citri*** | **even skipped**  **QC (ID)  Bit Score** | **hairy**  **QC (ID)  E-value** | **odd paired**  **QC (ID)  E-value** | **runt**  **QC (ID)  Bit Score** |
| --- | --- | --- | --- | --- |
| ***Acyrthosiphon pisum*** | 38%  (91%)  160 | 92%  (48%)  223 | 44%  (79%)  320 | 35%  (76%)  208 |
| ***Halyomorpha halys*** | 24%  (88%)  149 | 59%  (53%)  202 | 42%  (83%)  316 | 56%  (64%)  254 |
| ***Cimex lectularius*** | 40%  (90%)  154 | 57%  (54%)  184 | ND | 52%  (70%)  268 |
| ***Anopheles gambiae*** | 24%  (90%)  152 | 68%  (55%)  234 | 47%  (70%)  332 | 54%  (66%)  253 |
| ***Aedes aegypti*** | 25%  (85%)  152 | 92%  (46%)  258 | 61%  (44%)  220 | 49%  (67%)  250 |
| ***Drosophila melanogaster*** | 24%  (88%)  149 | 57%  (56%)  196 | 46%  (76%)  328 | 49%  (69%)  253 |

**Table 5**. Represents Query Coverage (Identity) and E-value of the MCOT gene models pairwise aligned to orthologues in other species. Pairwise alignment was performed using NCBI blast.


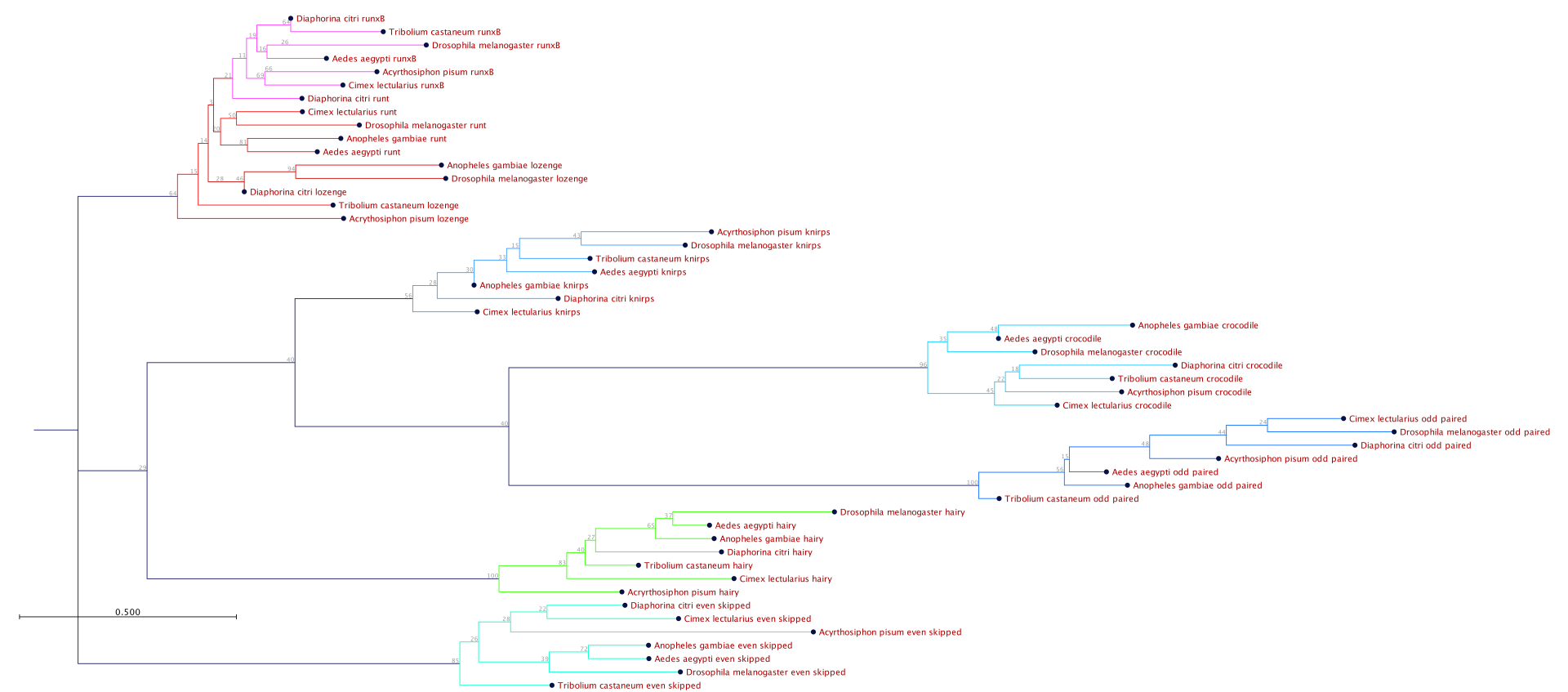


Figure1: Neighbor- Joining tree for early development genes genes in *D. citri* using 1000 bootstrap replicates, Poisson model, and complete deletion. *D. citri* consistently appears close to fellow hemipteran *A. pisum* and beetle *T. castaneum.*

**References**

1. El-Shesheny, I., et al., Silencing abnormal wing disc gene of the Asian citrus psyllid, *Diaphorina citri* disrupts adult wing development and increases nymph mortality. *PLoS One*., 2013. **8**(5): p. e65392.

2. Angelini, D.R. and T.C. Kaufman, Comparative developmental genetics and the evolution of arthropod body plans. *Annual Review of Genetics*, 2005. **39**: p. 95-119.

3. Attrill, H., et al., FlyBase: establishing a Gene Group resource for *Drosophila melanogaster*. *Nucleic Acids Research*., 2016. **44**(D1): p. D786-792. .

# Supplementary Note 36: Cuticle proteins

**Introduction**

The cuticle of arthropods provides the structural and mechanical support and protection that has contributed to the success of these organisms in diverse habitats (Neville, 1975). Arthropod cuticles are primarily composed of chitin fibers embedded in a proteinaceous matrix (Neville, 1975) that consists of a variety of cuticle proteins (reviewed in Willis et al., 2005). The number and types of cuticle proteins present are diverse among the arthropods with individual species containing some subset of approximately a dozen families of cuticle protein (Ioannidou et al, 2014; Willis, 2010). Families are generally classified by the presence of conserved motifs, with the CPR family, characterized by the “R&R Consensus” domain (Rebers and Riddiford, 1988), being the largest. Certain families, including CPR, CPAP1 and CPAP3, are common among arthropods whereas families such as CPCFC and CPLCW are restricted to certain orders and/or even smaller taxonomic groups (Ioannidou et al, 2014; Willis, 2010). However, the understanding of the diversity of cuticle proteins is currently evolving as more arthropod genomes are sequenced and expression patterns of cuticle protein genes are examined.

**Methods**

The two assembly versions for *Diaphorina citri* (diaci1.1 generated by the Baylor College of Medicine Human Genome Sequencing Center (BCM-HGSC**)**, and the NCBI GenBank- and RefSeq-processed version; NCBI-diaci1.1) were blasted (BLASTp; McGinnis and Madden, 2004) using sequence motifs that are characteristic of several families of cuticle proteins (Willis, 2002). Predicted cuticle proteins were further analyzed with CutProtFam-Pred, a cuticular protein family prediction tool described in Ioannidou et al. (2014), to assign genes to specific gene families of cuticle proteins. To find the closest putative homolog to cuticle protein genes from *D. citri*, genes were searched against the RefSeq protein sets downloaded from the National Center for Biotechnology Information (NCBI), or the official gene set for *Acyrthosiphon pisum* (Refseq), *Cimex lectularius* (maker v. 5.3, BCM-HGSC; Benoit et al., 2016), Gerris buenoi (maker v. 5.3, BCM-HGSC), *Oncopeltus fasciatus* (maker v. 5.3, BCM-HGSC), *Rhodnius prolixus* (CDC v. 3.1, downloaded from VectorBase), *Drosophila melanogaster* (Refseq), *Bombyx mori* (Refseq), *Tribolium castaneum* (Refseq), *Apis mellifera* (Refseq), and *Pediculus humanus corporis* (Refseq). The protein sequence with the lowest e-value was considered the closest putative homolog. Due to the relatively few number of cuticle protein genes found in the assembled genome, MCOT v1.0 transcriptome was also analyzed for putative cuticle proteins using the same methods described above.

| Table 1. Number of genes identified as putative cuticle proteins per family in the genome of *Diaphorina citri*. | | | | | | | |
| --- | --- | --- | --- | --- | --- | --- | --- |
|  | CPR^a^ |  |  |  |  |  |  |
| **RR-1** | **RR-2** | **Uncl** | **CPAP1** | **CPAP3** | **CPF** | **TWDL** | **Total** |
| 9 | 26 | 3 | 11 | 8 | 3 | 3 | 63 |
| ^a^Sequences that scored above the assigned cutoffs for the RR-1 and RR-2 models were classified as the corresponding type, whereas sequences with scores below the assigned cutoffs but above 0 were characterized as “unclassified” (Uncl). For more information, see Ioannidou et al. (2014). | | | | | | | |

| Table 1b. Number of genes identified as putative cuticle proteins per family in the genome of *Diaphorina citri*. | | | | | | | |
| --- | --- | --- | --- | --- | --- | --- | --- |
|  | CPR^a^ |  |  |  |  |  |  |
| **RR-1** | **RR-2** | **Uncl** | **CPAP1** | **CPAP3** | **CPF** | **TWDL** | **Total** |
| 11 | 33 | 20 | 16 | 7 | 2 | 3 | 92 |
| ^a^Sequences that scored above the assigned cutoffs for the RR-1 and RR-2 models were classified as the corresponding type, whereas sequences with scores below the assigned cutoffs but above 0 were characterized as “unclassified” (Uncl). For more information, see Ioannidou et al. (2014). | | | | | | | |

| Table 2. Number of genes identified as putative cuticle proteins per species in the genomes of several insect orders. | | | | | | | | | | |
| --- | --- | --- | --- | --- | --- | --- | --- | --- | --- | --- |
| **Order** | **Species** | **CPR** | **CPAP1** | **CPAP3** | **CPCFC** | **CPF** | **CPLCA** | **CPLCG** | **TWDL** | **Total** |
| Hemiptera | *Acyrthosiphon pisum* ^b^ | 117 | 11 | 10 | 1 | 3 | 0 | 0 | 3 | 145 |
|  | *Cimex lectularius* ^c^ | 121 | 15 | 6 | 0 | 5 | 0 | 0 | 3 | 149 |
|  | *Diaphorina citri* ^a^ | 38 | 11 | 8 | 0 | 3 | 0 | 0 | 3 | 63 |
|  | *Gerris buenoi* ^a^ | 126 | 10 | 6 | 0 | 3 | 0 | 0 | 10 | 155 |
|  | *Oncopeltus fasciatus* ^a^ | 133 | 11 | 7 | 0 | 7 | 0 | 0 | 3 | 173 |
|  | *Rhodnius prolixus* ^a^ | 91 | 10 | 6 | 1 | 2 | 0 | 0 | 1 | 111 |
| Diptera | *Drosophila melanogaster^b^* | 137 | 29 | 10 | 1 | 5 | 13 | 4 | 29 | 228 |
| Lepidoptera | *Bombyx mori^b^* | 144 | 13 | 6 | 1 | 1 | 2 | 0 | 4 | 171 |
| Coleoptera | *Tribolium castaneum^b^* | 110 | 13 | 7 | 2 | 5 | 1 | 1 | 3 | 142 |
| Hymenoptera | *Apis* *mellifera* ^b^ | 38 | 15 | 7 | 0 | 4 | 0 | 0 | 2 | 66 |
| Phthiraptera | *Pediculus humanus* ^b^ | 41 | 12 | 6 | 1 | 1 | 0 | 0 | 2 | 63 |
| ^a^ Cuticle protein numbers determined by analyzing gene sets with CutProtFam-Pred (Ioannidou et al., 2014).  ^b^ Cuticle protein numbers determined from Ioannidou et al. (2014).  ^c^ Cuticle protein numbers determined from Benoit et al. (2015). | | | | | | | | | | |

**Results**

Sixty-three genes encoding for putative cuticle proteins were identified in *D. citri* by searching the genome with sequence motifs characteristic of different cuticle protein families as established by Willis (2002). CutProtFam-Pred (Ioannidou et *al*., 2014) was employed to assign these genes to one of five families (CPR, CPAP1, CPAP3, CPF, and TWDL; Table 1). The total number of cuticle protein genes identified in *D. citri* (63) is much less than in other hemipterans (111-173; Ioannidou et al., 2014), but is within the range of that observed in other insects (Table 2). It is unclear if this reduced number accurately reflects the number of genes in *D. citri*, or is a reflection of a suboptimal genome assembly. As with other insects, the number of CPR genes (38) constituted the largest group of cuticle protein genes in the *D. citri* genome; however, this number is less than in other hemipterans and accounted for the reduced number of total cuticle protein genes. The number of genes in the protein families CPAP1, CPAP3, CPF, and TWDL was similar to the number in other insects (Ioannidou et al., 2014; Table 2). Within MCOT transcriptome 92 genes were identified as putative cuticle proteins. The greater number of cuticle protein transcripts was partially due to multiple isoforms of single genes; however, some were unique from the identified genes, suggesting the assembled genome may be lacking several expected genes.

**References**

1. Benoit JB, Adelman ZN, Reinhardt K, Dolan A, Jennings EC, Szuter EM, et al. (2016) Unique features of a global human ectoparasite identified through sequencing of the bed bug genome. *Nature Communications*. 7.
2. Ioannidou, Z. S., Theodoropoulou, M. C., Papandreou, N. C., Willis, J. H., & Hamodrakas, S. J. (2014). CutProtFam-Pred: Detection and classification of putative structural cuticular proteins from sequence alone, based on profile Hidden Markov Models. *Insect biochemistry and molecular biology*. 52, 51-59.
3. McGinnis S and Madden TL. (2004). BLAST: at the core of a powerful and diverse set of sequence analysis tools. *Nucleic Acids Research*. 32, W20-W25.
4. Neville, A.C. (1975). Biology of the Arthropod Cuticle. New York, Springer-Verlag
5. Rebers, J.E., Riddiford, L.M. (1988) Structure and expression of a *Manduca sexta* larval cuticle gene homologous to *Drosophila* cuticle genes. *Journal of Molecular Biology*, 203: 411–423.
6. Willis, J. H. (2010). Structural cuticular proteins from arthropods: annotation, nomenclature, and sequence characteristics in the genomics era. *Insect biochemistry and molecular biology*. 40, 189-204.
7. Willis, J.H., Iconomidou, V.A., Smith, R.F., Hamodrakas, S.J. (2005). Cuticular proteins. *Comprehensive Insect Science*. Eds. Gilbert, L.I., Iatrou, K., Gill, S. Oxford, Elsevier, 4: 79-109.

# Supplementary Note 37: Secretome

**Introduction**

Plant-feeding hemiptera secrete watery, enzymatic saliva, and sheath saliva which hardens to support stylet movement and reduce plant response to wounding (Moreno et al., 2012). Secreted proteins (including potential effectors) from insect plant pest thought to be involved in pathogenicity, are encoded in their genomes and can be identified by sequence analyses based on secretion signals (Rodriguez and Bos, 2013; Jaouannet et al., 2014). To date effectors from insect pest pathogens have been identified using bioinformatics analyses from transcript data obtained from insect salivary glands and other tissues (Bos et al., 2010).

**Materials and Methods**

The presence of signal peptide secretion signals is a universal feature of effectors plant, insect and mammalian parasitic microbes and can be used as an initial filtering step (Shang et al., 2016). We screened all predicted proteins (20,622) from version 1.1 under accession NCBI accession GCF0004751951 for the presence of signal peptides using SignalP version 2.0 (Nielsen et al., 1997) and for the absence of transmembrane domains with TMHMM version 2.0 (Krogh et al., 2001). Secreted proteins with a SignalP HMM score > 0.9, NN cleavage site within 10 and 40 amino acids (aa), and no transmembrane domains found at >40 amino acids away from the starting amino acid methionine, were selected. From the secretome list, we performed a search for proteins with 2% or higher % of cysteines and no more than 250 amino acids in length. We used the group of small cysteine rich proteins and described the presence of nuclear localization signals (NLSs) using NLStradamus (Nguyen Ba et al., 2009) and Chloroplast motifs using ChloroP v1.0 (Emanuelsson et al., 1999). The full-length protein sequences of the 246 predicted secreted proteins were used in a similarity search against each other with Blastp (Altschul et al., 1997) using e-value 10^-5^. Proteins were clustered with Tribe-MCL (Enright et al., 2002) using mcxload and mcl programs (van Dongen and Abreu-Goodger, 2012) and an inflation parameter I value of 0.5 to control cluster tightness. Sequences were extracted using custom scripts described by Saunders et al. (2012) . The sequence alignment and phylogenic neighbor joining tree were built with full-length amino acid sequences in ClustalX version 2.0 (Chenna et al., 2003).

Figure 1: Ejaculatory bulb-specific protein 3-like EbpII odorant binding like protein secreted protein family. A. Multiple sequence alignment of EbpII family (Tribe1) full-length amino acid sequences. B. Phylogeny of EbpII family (Tribe1) full-length amino acid sequences.

Table 1: Prediction and annotations for 246 putative secreted proteins in *Diaphorina citri*.

| **Gene ID** | **Description** |
| --- | --- |
| XP_008482042.1 | 15 kDa selenoprotein [Diaphorina citri] --HMM score = 0.963 --Signal peptide length = 24 PredHel = 0 Topology = o len = 176 |
| XP_017298172.1 | ABC transporter A family member 9-like [Diaphorina citri] --HMM score = 0.920 --Signal peptide length = 15 PredHel = 1 Topology = i2-24o len = 140 |
| XP_008482159.1 | acidic mammalian chitinase-like [Diaphorina citri] --HMM score = 1.000 --Signal peptide length = 24 PredHel = 0 Topology = o len = 84 |
| XP_017299188.1 | alpha-galactosidase A-like [Diaphorina citri] --HMM score = 1.000 --Signal peptide length = 22 PredHel = 0 Topology = o len = 146 |
| XP_017299189.1 | alpha-galactosidase A-like [Diaphorina citri] --HMM score = 1.000 --Signal peptide length = 22 PredHel = 0 Topology = o len = 146 |
| XP_008478669.1 | apolipoprotein D-like [Diaphorina citri] --HMM score = 0.971 --Signal peptide length = 32 PredHel = 0 Topology = o len = 189 |
| XP_008484002.1 | astakine-like, partial [Diaphorina citri] --HMM score = 0.999 --Signal peptide length = 20 PredHel = 0 Topology = o len = 72 |
| XP_008472105.1 | autophagy-related protein 2 homolog A-like [Diaphorina citri] --HMM score = 1.000 --Signal peptide length = 19 PredHel = 0 Topology = o len = 147 |
| XP_008477626.1 | basic salivary proline-rich protein 3-like [Diaphorina citri] --HMM score = 0.986 --Signal peptide length = 27 PredHel = 1 Topology = i7-29o len = 123 |
| XP_008475283.1 | cathepsin B-like [Diaphorina citri] --HMM score = 0.999 --Signal peptide length = 16 PredHel = 0 Topology = o len = 128 |
| XP_008480568.1 | chondroitin proteoglycan 2-like [Diaphorina citri] --HMM score = 1.000 --Signal peptide length = 20 PredHel = 0 Topology = o len = 247 |
| XP_008487969.1 | chorion class high-cysteine HCB protein 13-like [Diaphorina citri] --HMM score = 1.000 --Signal peptide length = 20 PredHel = 0 Topology = o len = 74 |
| XP_008485271.1 | clustered mitochondria protein homolog, partial [Diaphorina citri] --HMM score = 0.940 --Signal peptide length = 24 PredHel = 0 Topology = o len = 79 |
| XP_008472726.1 | connective tissue growth factor-like [Diaphorina citri] --HMM score = 0.993 --Signal peptide length = 22 PredHel = 0 Topology = o len = 159 |
| XP_008473317.1 | ctenidin-1-like isoform X1 [Diaphorina citri] --HMM score = 0.991 --Signal peptide length = 21 PredHel = 0 Topology = o len = 203 |
| XP_017303134.1 | cystatin-C-like isoform X1 [Diaphorina citri] --HMM score = 1.000 --Signal peptide length = 20 PredHel = 0 Topology = o len = 150 |
| XP_008481260.1 | cystatin-C-like isoform X2 [Diaphorina citri] --HMM score = 1.000 --Signal peptide length = 20 PredHel = 0 Topology = o len = 150 |
| XP_008481261.1 | cystatin-C-like isoform X3 [Diaphorina citri] --HMM score = 1.000 --Signal peptide length = 20 PredHel = 0 Topology = o len = 144 |
| XP_008487197.1 | cystatin-C-like, partial [Diaphorina citri] --HMM score = 1.000 --Signal peptide length = 20 PredHel = 0 Topology = o len = 140 |
| XP_008479809.1 | eclosion hormone-like [Diaphorina citri] --HMM score = 0.999 --Signal peptide length = 25 PredHel = 0 Topology = o len = 65 |
| XP_008484466.1 | eclosion hormone-like [Diaphorina citri] --HMM score = 0.999 --Signal peptide length = 25 PredHel = 0 Topology = o len = 80 |
| XP_017300318.1 | ejaculatory bulb-specific protein 3-like [Diaphorina citri] --HMM score = 0.992 --Signal peptide length = 21 PredHel = 0 Topology = o len = 156 |
| XP_008478140.1 | ejaculatory bulb-specific protein 3-like [Diaphorina citri] --HMM score = 0.993 --Signal peptide length = 22 PredHel = 0 Topology = o len = 137 |
| XP_008473947.1 | ejaculatory bulb-specific protein 3-like [Diaphorina citri] --HMM score = 0.998 --Signal peptide length = 23 PredHel = 0 Topology = o len = 135 |
| XP_008478451.1 | ejaculatory bulb-specific protein 3-like [Diaphorina citri] --HMM score = 0.998 --Signal peptide length = 23 PredHel = 1 Topology = o4-21i len = 75 |
| XP_008478860.1 | ejaculatory bulb-specific protein 3-like [Diaphorina citri] --HMM score = 0.999 --Signal peptide length = 19 PredHel = 0 Topology = o len = 124 |
| XP_008482474.1 | ejaculatory bulb-specific protein 3-like [Diaphorina citri] --HMM score = 0.999 --Signal peptide length = 19 PredHel = 0 Topology = o len = 88 |
| XP_008478138.1 | ejaculatory bulb-specific protein 3-like [Diaphorina citri] --HMM score = 0.999 --Signal peptide length = 20 PredHel = 0 Topology = o len = 128 |
| XP_017303149.1 | ejaculatory bulb-specific protein 3-like [Diaphorina citri] --HMM score = 0.999 --Signal peptide length = 20 PredHel = 0 Topology = o len = 136 |
| XP_017303150.1 | ejaculatory bulb-specific protein 3-like [Diaphorina citri] --HMM score = 0.999 --Signal peptide length = 20 PredHel = 0 Topology = o len = 136 |
| XP_008478141.1 | ejaculatory bulb-specific protein 3-like [Diaphorina citri] --HMM score = 0.999 --Signal peptide length = 20 PredHel = 0 Topology = o len = 67 |
| XP_008471452.1 | ejaculatory bulb-specific protein 3-like [Diaphorina citri] --HMM score = 1.000 --Signal peptide length = 18 PredHel = 0 Topology = o len = 124 |
| XP_008471453.1 | ejaculatory bulb-specific protein 3-like [Diaphorina citri] --HMM score = 1.000 --Signal peptide length = 18 PredHel = 0 Topology = o len = 124 |
| XP_008481200.1 | ejaculatory bulb-specific protein 3-like [Diaphorina citri] --HMM score = 1.000 --Signal peptide length = 18 PredHel = 0 Topology = o len = 67 |
| XP_008486851.1 | ejaculatory bulb-specific protein 3-like, partial [Diaphorina citri] --HMM score = 0.999 --Signal peptide length = 19 PredHel = 1 Topology = i5-24o len = 59 |
| XP_017301641.1 | epidermal growth factor-like protein 7 [Diaphorina citri] --HMM score = 0.949 --Signal peptide length = 24 PredHel = 0 Topology = o len = 103 |
| XP_017304908.1 | esterase 6-like, partial [Diaphorina citri] --HMM score = 1.000 --Signal peptide length = 20 PredHel = 1 Topology = o4-26i len = 63 |
| XP_008474275.1 | extensin-1-like [Diaphorina citri] --HMM score = 0.995 --Signal peptide length = 21 PredHel = 0 Topology = o len = 144 |
| XP_017304793.1 | Fanconi anemia group I protein homolog, partial [Diaphorina citri] --HMM score = 0.947 --Signal peptide length = 21 PredHel = 0 Topology = o len = 180 |
| XP_008468088.1 | folate receptor beta-like [Diaphorina citri] --HMM score = 0.958 --Signal peptide length = 17 PredHel = 0 Topology = o len = 211 |
| XP_008468232.1 | frizzled-2-like [Diaphorina citri] --HMM score = 0.997 --Signal peptide length = 23 PredHel = 1 Topology = i7-29o len = 117 |
| XP_008469244.1 | gamma-interferon-inducible lysosomal thiol reductase-like [Diaphorina citri] --HMM score = 0.999 --Signal peptide length = 23 PredHel = 0 Topology = o len = 212 |
| XP_008470659.1 | general odorant-binding protein 83a-like [Diaphorina citri] --HMM score = 0.989 --Signal peptide length = 23 PredHel = 0 Topology = o len = 140 |
| XP_017302279.1 | geranylgeranyl transferase type-2 subunit beta-like, partial [Diaphorina citri] --HMM score = 0.988 --Signal peptide length = 21 PredHel = 0 Topology = o len = 163 |
| XP_008469206.1 | GILT-like protein C02D5.2 [Diaphorina citri] --HMM score = 0.996 --Signal peptide length = 23 PredHel = 0 Topology = o len = 208 |
| XP_008469207.1 | GILT-like protein C02D5.2 [Diaphorina citri] --HMM score = 0.996 --Signal peptide length = 23 PredHel = 0 Topology = o len = 208 |
| XP_008469208.1 | GILT-like protein C02D5.2 [Diaphorina citri] --HMM score = 0.996 --Signal peptide length = 23 PredHel = 0 Topology = o len = 208 |
| XP_017297739.1 | glutaredoxin-C4-like [Diaphorina citri] --HMM score = 0.967 --Signal peptide length = 24 PredHel = 1 Topology = o4-26i len = 117 |
| XP_017297749.1 | glutaredoxin-like isoform X1 [Diaphorina citri] --HMM score = 0.992 --Signal peptide length = 20 PredHel = 0 Topology = o len = 122 |
| XP_008487182.1 | glycine-rich cell wall structural protein 1.0-like isoform X1 [Diaphorina citri] --HMM score = 0.991 --Signal peptide length = 21 PredHel = 0 Topology = o len = 199 |
| XP_008487183.1 | glycine-rich cell wall structural protein 1.0-like isoform X2 [Diaphorina citri] --HMM score = 0.991 --Signal peptide length = 21 PredHel = 0 Topology = o len = 196 |
| XP_008473318.1 | glycine-rich cell wall structural protein 1.0-like isoform X2 [Diaphorina citri] --HMM score = 0.991 --Signal peptide length = 21 PredHel = 0 Topology = o len = 200 |
| XP_008488257.1 | hypertrehalosaemic prohormone-like [Diaphorina citri] --HMM score = 0.983 --Signal peptide length = 24 PredHel = 1 Topology = i7-29o len = 74 |
| XP_017299390.1 | insulin gene enhancer protein isl-2a-like [Diaphorina citri] --HMM score = 0.971 --Signal peptide length = 18 PredHel = 0 Topology = o len = 146 |
| XP_008468127.1 | insulin-2-like [Diaphorina citri] --HMM score = 0.980 --Signal peptide length = 26 PredHel = 0 Topology = o len = 136 |
| XP_008473632.1 | jacalin-related lectin 34-like isoform X2 [Diaphorina citri] --HMM score = 1.000 --Signal peptide length = 18 PredHel = 0 Topology = o len = 228 |
| XP_008470248.1 | lachesin-like [Diaphorina citri] --HMM score = 1.000 --Signal peptide length = 24 PredHel = 0 Topology = o len = 147 |
| XP_008478723.1 | latex clearing protein-like, partial [Diaphorina citri] --HMM score = 0.934 --Signal peptide length = 31 PredHel = 0 Topology = o len = 225 |
| XP_017303321.1 | leucine-rich repeat and immunoglobulin-like domain-containing nogo receptor-interacting protein 1-B [Diaphorina citri] --HMM score = 0.998 --Signal peptide length = 21 PredHel = 0 Topology = o len = 124 |
| XP_017299004.1 | leucine-rich repeat-containing protein 26-like, partial [Diaphorina citri] --HMM score = 0.991 --Signal peptide length = 25 PredHel = 0 Topology = o len = 133 |
| XP_008468126.1 | LIRP-like [Diaphorina citri] --HMM score = 0.980 --Signal peptide length = 26 PredHel = 0 Topology = o len = 136 |
| XP_017297981.1 | LIRP-like [Diaphorina citri] --HMM score = 0.980 --Signal peptide length = 26 PredHel = 0 Topology = o len = 136 |
| XP_017302049.1 | lysozyme-like isoform X1 [Diaphorina citri] --HMM score = 0.944 --Signal peptide length = 22 PredHel = 1 Topology = i7-25o len = 165 |
| XP_008478360.1 | lysozyme-like isoform X2 [Diaphorina citri] --HMM score = 0.944 --Signal peptide length = 22 PredHel = 1 Topology = i7-25o len = 165 |
| XP_008485854.1 | malectin-A-like, partial [Diaphorina citri] --HMM score = 0.966 --Signal peptide length = 26 PredHel = 1 Topology = i9-31o len = 70 |
| XP_008474209.1 | mesencephalic astrocyte-derived neurotrophic factor homolog [Diaphorina citri] --HMM score = 0.982 --Signal peptide length = 24 PredHel = 0 Topology = o len = 176 |
| XP_017301153.1 | neprilysin-2-like [Diaphorina citri] --HMM score = 0.997 --Signal peptide length = 15 PredHel = 0 Topology = o len = 100 |
| XP_017304350.1 | neuroendocrine protein 7B2 isoform X1 [Diaphorina citri] --HMM score = 0.996 --Signal peptide length = 17 PredHel = 0 Topology = o len = 238 |
| XP_008484960.1 | neuroendocrine protein 7B2 isoform X2 [Diaphorina citri] --HMM score = 0.996 --Signal peptide length = 17 PredHel = 0 Topology = o len = 238 |
| XP_017304351.1 | neuroendocrine protein 7B2 isoform X3 [Diaphorina citri] --HMM score = 0.996 --Signal peptide length = 17 PredHel = 0 Topology = o len = 238 |
| XP_017302603.1 | neuroligin-4, Y-linked-like [Diaphorina citri] --HMM score = 0.999 --Signal peptide length = 22 PredHel = 0 Topology = o len = 150 |
| XP_008468890.1 | neuropeptide SIFamide-like [Diaphorina citri] --HMM score = 0.991 --Signal peptide length = 23 PredHel = 1 Topology = i5-23o len = 75 |
| XP_008468892.1 | neuropeptide SIFamide-like [Diaphorina citri] --HMM score = 0.991 --Signal peptide length = 23 PredHel = 1 Topology = i5-23o len = 75 |
| XP_008487361.1 | nucleoside diphosphate kinase-like, partial [Diaphorina citri] --HMM score = 0.982 --Signal peptide length = 23 PredHel = 0 Topology = o len = 75 |
| XP_008471862.1 | peroxiredoxin-4-like [Diaphorina citri] --HMM score = 0.992 --Signal peptide length = 22 PredHel = 0 Topology = o len = 208 |
| XP_008470698.1 | peroxiredoxin-5, mitochondrial-like [Diaphorina citri] --HMM score = 0.955 --Signal peptide length = 19 PredHel = 0 Topology = o len = 174 |
| XP_017298995.1 | phospholipase A2-like [Diaphorina citri] --HMM score = 0.994 --Signal peptide length = 23 PredHel = 1 Topology = o4-21i len = 171 |
| XP_017300972.1 | pleiotrophin-A-like isoform X1 [Diaphorina citri] --HMM score = 1.000 --Signal peptide length = 19 PredHel = 0 Topology = o len = 154 |
| XP_017300973.1 | pleiotrophin-A-like isoform X2 [Diaphorina citri] --HMM score = 1.000 --Signal peptide length = 19 PredHel = 0 Topology = o len = 149 |
| XP_008477640.1 | probable G-protein coupled receptor Mth-like 5, partial [Diaphorina citri] --HMM score = 0.993 --Signal peptide length = 30 PredHel = 0 Topology = o len = 158 |
| XP_008486968.1 | probable protein disulfide-isomerase A6 isoform X1 [Diaphorina citri] --HMM score = 0.998 --Signal peptide length = 18 PredHel = 0 Topology = o len = 83 |
| XP_008486974.1 | probable protein disulfide-isomerase A6 isoform X2 [Diaphorina citri] --HMM score = 0.999 --Signal peptide length = 18 PredHel = 0 Topology = o len = 83 |
| NP_001306216.1 | probable xyloglucan-specific endo-beta-1,4-glucanase A precursor [Diaphorina citri] --HMM score = 1.000 --Signal peptide length = 22 PredHel = 0 Topology = o len = 230 |
| XP_008474579.1 | prohormone-3 [Diaphorina citri] --HMM score = 0.966 --Signal peptide length = 21 PredHel = 0 Topology = o len = 179 |
| XP_008469102.1 | protein quiver-like [Diaphorina citri] --HMM score = 0.999 --Signal peptide length = 38 PredHel = 0 Topology = i len = 64 |
| XP_017299179.1 | protein slit-like [Diaphorina citri] --HMM score = 0.980 --Signal peptide length = 32 PredHel = 1 Topology = o15-37i len = 147 |
| XP_008481158.1 | protein twisted gastrulation-like [Diaphorina citri] --HMM score = 0.948 --Signal peptide length = 30 PredHel = 0 Topology = o len = 114 |
| XP_008470818.1 | protein-L-isoaspartate O-methyltransferase 1-like [Diaphorina citri] --HMM score = 0.996 --Signal peptide length = 23 PredHel = 0 Topology = o len = 199 |
| XP_008473927.1 | putative odorant-binding protein A10 [Diaphorina citri] --HMM score = 0.993 --Signal peptide length = 18 PredHel = 0 Topology = o len = 121 |
| XP_008473937.1 | putative odorant-binding protein A10 [Diaphorina citri] --HMM score = 0.995 --Signal peptide length = 22 PredHel = 0 Topology = o len = 127 |
| XP_008473631.1 | putative per-hexamer repeat protein 5 isoform X1 [Diaphorina citri] --HMM score = 1.000 --Signal peptide length = 18 PredHel = 0 Topology = o len = 239 |
| XP_008481743.1 | quinone oxidoreductase-like [Diaphorina citri] --HMM score = 0.999 --Signal peptide length = 22 PredHel = 0 Topology = o len = 191 |
| XP_017302036.1 | RNA polymerase II transcriptional coactivator-like isoform X1 [Diaphorina citri] --HMM score = 0.994 --Signal peptide length = 21 PredHel = 0 Topology = o len = 139 |
| XP_017302037.1 | RNA polymerase II transcriptional coactivator-like isoform X1 [Diaphorina citri] --HMM score = 0.994 --Signal peptide length = 21 PredHel = 0 Topology = o len = 139 |
| XP_017302038.1 | RNA polymerase II transcriptional coactivator-like isoform X1 [Diaphorina citri] --HMM score = 0.994 --Signal peptide length = 21 PredHel = 0 Topology = o len = 139 |
| XP_017302039.1 | RNA polymerase II transcriptional coactivator-like isoform X1 [Diaphorina citri] --HMM score = 0.994 --Signal peptide length = 21 PredHel = 0 Topology = o len = 139 |
| XP_017302041.1 | RNA polymerase II transcriptional coactivator-like isoform X1 [Diaphorina citri] --HMM score = 0.994 --Signal peptide length = 21 PredHel = 0 Topology = o len = 139 |
| XP_008478347.1 | RNA polymerase II transcriptional coactivator-like isoform X2 [Diaphorina citri] --HMM score = 0.994 --Signal peptide length = 21 PredHel = 0 Topology = o len = 119 |
| XP_008477322.1 | serine protease VLSP-1-like, partial [Diaphorina citri] --HMM score = 0.998 --Signal peptide length = 21 PredHel = 0 Topology = o len = 125 |
| XP_017301918.1 | Kazal-like SPARC-like protein 1 [Diaphorina citri] --HMM score = 1.000 --Signal peptide length = 22 PredHel = 0 Topology = o len = 190 |
| XP_008482273.1 | thioredoxin domain-containing protein 12-like [Diaphorina citri] --HMM score = 0.987 --Signal peptide length = 19 PredHel = 0 Topology = o len = 158 |
| XP_008482491.1 | thioredoxin domain-containing protein 12-like [Diaphorina citri] --HMM score = 0.998 --Signal peptide length = 20 PredHel = 0 Topology = o len = 148 |
| XP_008485322.1 | thioredoxin-2-like isoform X1 [Diaphorina citri] --HMM score = 0.942 --Signal peptide length = 22 PredHel = 0 Topology = o len = 115 |
| XP_017300397.1 | transmembrane 9 superfamily member 2-like [Diaphorina citri] --HMM score = 0.993 --Signal peptide length = 18 PredHel = 0 Topology = o len = 64 |
| XP_008487451.1 | tribbles homolog 3-like, partial [Diaphorina citri] --HMM score = 0.912 --Signal peptide length = 24 PredHel = 0 Topology = o len = 144 |
| XP_008475553.1 | turripeptide Lol9.1-like isoform X1 [Diaphorina citri] --HMM score = 0.999 --Signal peptide length = 23 PredHel = 1 Topology = o4-21i len = 89 |
| XP_008475554.1 | turripeptide Lol9.1-like isoform X2 [Diaphorina citri] --HMM score = 0.999 --Signal peptide length = 23 PredHel = 1 Topology = o4-21i len = 89 |
| XP_008481695.1 | tyrosine-protein kinase transmembrane receptor Ror-like [Diaphorina citri] --HMM score = 0.943 --Signal peptide length = 29 PredHel = 1 Topology = o4-26i len = 207 |
| NP_001284620.1 | uncharacterized LOC103519184 precursor [Diaphorina citri] --HMM score = 1.000 --Signal peptide length = 17 PredHel = 0 Topology = o len = 148 |
| XP_008486709.1 | uncharacterized monothiol glutaredoxin ycf64-like, partial [Diaphorina citri] --HMM score = 0.917 --Signal peptide length = 25 PredHel = 1 Topology = o10-29i len = 66 |
| XP_008467481.1 | uncharacterized protein LOC103504956 [Diaphorina citri] --HMM score = 0.999 --Signal peptide length = 21 PredHel = 0 Topology = o len = 120 |
| XP_008467997.1 | uncharacterized protein LOC103505443 [Diaphorina citri] --HMM score = 0.929 --Signal peptide length = 19 PredHel = 0 Topology = o len = 223 |
| XP_008468059.1 | uncharacterized protein LOC103505499 isoform X2 [Diaphorina citri] --HMM score = 0.994 --Signal peptide length = 22 PredHel = 0 Topology = o len = 83 |
| XP_008468200.1 | uncharacterized protein LOC103505626 isoform X1 [Diaphorina citri] --HMM score = 0.999 --Signal peptide length = 23 PredHel = 1 Topology = o4-26i len = 112 |
| XP_008468201.1 | uncharacterized protein LOC103505626 isoform X2 [Diaphorina citri] --HMM score = 0.999 --Signal peptide length = 23 PredHel = 1 Topology = o4-26i len = 78 |
| XP_008468499.1 | uncharacterized protein LOC103505904 [Diaphorina citri] --HMM score = 0.973 --Signal peptide length = 17 PredHel = 0 Topology = o len = 188 |
| XP_008468771.1 | uncharacterized protein LOC103506162 [Diaphorina citri] --HMM score = 0.998 --Signal peptide length = 22 PredHel = 0 Topology = o len = 169 |
| XP_017298314.1 | uncharacterized protein LOC103506345 [Diaphorina citri] --HMM score = 0.975 --Signal peptide length = 28 PredHel = 1 Topology = o4-26i len = 118 |
| XP_008469242.1 | uncharacterized protein LOC103506625 [Diaphorina citri] --HMM score = 0.967 --Signal peptide length = 20 PredHel = 0 Topology = o len = 129 |
| XP_008469286.1 | uncharacterized protein LOC103506671 [Diaphorina citri] --HMM score = 0.998 --Signal peptide length = 24 PredHel = 0 Topology = o len = 126 |
| XP_008469287.1 | uncharacterized protein LOC103506671 [Diaphorina citri] --HMM score = 0.998 --Signal peptide length = 24 PredHel = 0 Topology = o len = 126 |
| XP_008469288.1 | uncharacterized protein LOC103506672 [Diaphorina citri] --HMM score = 0.998 --Signal peptide length = 24 PredHel = 0 Topology = o len = 126 |
| XP_008469597.1 | uncharacterized protein LOC103506945 [Diaphorina citri] --HMM score = 0.925 --Signal peptide length = 25 PredHel = 0 Topology = o len = 145 |
| XP_008469915.1 | uncharacterized protein LOC103507240 [Diaphorina citri] --HMM score = 0.998 --Signal peptide length = 16 PredHel = 0 Topology = o len = 152 |
| XP_008470076.1 | uncharacterized protein LOC103507388 [Diaphorina citri] --HMM score = 0.995 --Signal peptide length = 25 PredHel = 1 Topology = i5-27o len = 132 |
| XP_008470083.1 | uncharacterized protein LOC103507391 [Diaphorina citri] --HMM score = 0.999 --Signal peptide length = 20 PredHel = 0 Topology = o len = 140 |
| XP_008470176.1 | uncharacterized protein LOC103507484 [Diaphorina citri] --HMM score = 1.000 --Signal peptide length = 20 PredHel = 0 Topology = o len = 138 |
| XP_008470863.1 | uncharacterized protein LOC103508106 [Diaphorina citri] --HMM score = 1.000 --Signal peptide length = 17 PredHel = 0 Topology = o len = 170 |
| XP_008470924.1 | uncharacterized protein LOC103508171 [Diaphorina citri] --HMM score = 0.999 --Signal peptide length = 22 PredHel = 0 Topology = o len = 199 |
| XP_008470940.1 | uncharacterized protein LOC103508185 [Diaphorina citri] --HMM score = 0.999 --Signal peptide length = 19 PredHel = 0 Topology = o len = 242 |
| XP_008471218.1 | uncharacterized protein LOC103508449 [Diaphorina citri] --HMM score = 0.988 --Signal peptide length = 31 PredHel = 1 Topology = i7-26o len = 134 |
| XP_017299347.1 | uncharacterized protein LOC103508661 isoform X1 [Diaphorina citri] --HMM score = 0.989 --Signal peptide length = 20 PredHel = 0 Topology = o len = 140 |
| XP_008471455.1 | uncharacterized protein LOC103508661 isoform X2 [Diaphorina citri] --HMM score = 0.989 --Signal peptide length = 20 PredHel = 0 Topology = o len = 130 |
| XP_008471535.1 | uncharacterized protein LOC103508742 [Diaphorina citri] --HMM score = 1.000 --Signal peptide length = 21 PredHel = 0 Topology = o len = 106 |
| XP_008471554.1 | uncharacterized protein LOC103508759 [Diaphorina citri] --HMM score = 0.925 --Signal peptide length = 25 PredHel = 0 Topology = o len = 102 |
| XP_008471577.1 | uncharacterized protein LOC103508782 isoform X4 [Diaphorina citri] --HMM score = 0.999 --Signal peptide length = 21 PredHel = 0 Topology = o len = 245 |
| XP_017299457.1 | uncharacterized protein LOC103508938 [Diaphorina citri] --HMM score = 0.972 --Signal peptide length = 30 PredHel = 1 Topology = o10-32i len = 133 |
| XP_008471753.1 | uncharacterized protein LOC103508946 [Diaphorina citri] --HMM score = 0.982 --Signal peptide length = 29 PredHel = 1 Topology = o10-32i len = 140 |
| XP_017299481.1 | uncharacterized protein LOC103508984 isoform X1 [Diaphorina citri] --HMM score = 0.986 --Signal peptide length = 19 PredHel = 0 Topology = o len = 153 |
| XP_008471797.1 | uncharacterized protein LOC103508984 isoform X2 [Diaphorina citri] --HMM score = 0.986 --Signal peptide length = 19 PredHel = 0 Topology = o len = 153 |
| XP_017299482.1 | uncharacterized protein LOC103508984 isoform X3 [Diaphorina citri] --HMM score = 0.986 --Signal peptide length = 19 PredHel = 0 Topology = o len = 152 |
| XP_017299682.1 | uncharacterized protein LOC103509026 [Diaphorina citri] --HMM score = 0.996 --Signal peptide length = 24 PredHel = 1 Topology = i7-29o len = 137 |
| XP_008471850.1 | uncharacterized protein LOC103509036 [Diaphorina citri] --HMM score = 0.963 --Signal peptide length = 19 PredHel = 0 Topology = o len = 132 |
| XP_008471928.1 | uncharacterized protein LOC103509115 [Diaphorina citri] --HMM score = 0.993 --Signal peptide length = 23 PredHel = 0 Topology = o len = 134 |
| XP_008471929.1 | uncharacterized protein LOC103509116 [Diaphorina citri] --HMM score = 0.969 --Signal peptide length = 24 PredHel = 1 Topology = i7-26o len = 134 |
| XP_017299608.1 | uncharacterized protein LOC103509257 [Diaphorina citri] --HMM score = 0.942 --Signal peptide length = 21 PredHel = 0 Topology = o len = 136 |
| XP_008472279.1 | uncharacterized protein LOC103509439 [Diaphorina citri] --HMM score = 0.998 --Signal peptide length = 19 PredHel = 0 Topology = o len = 85 |
| XP_008472722.1 | uncharacterized protein LOC103509867 [Diaphorina citri] --HMM score = 0.927 --Signal peptide length = 31 PredHel = 0 Topology = o len = 193 |
| XP_008472811.1 | uncharacterized protein LOC103509953 [Diaphorina citri] --HMM score = 0.996 --Signal peptide length = 25 PredHel = 0 Topology = i len = 102 |
| XP_008473711.1 | uncharacterized protein LOC103510798 [Diaphorina citri] --HMM score = 0.991 --Signal peptide length = 22 PredHel = 0 Topology = o len = 244 |
| XP_008474025.1 | uncharacterized protein LOC103511092 [Diaphorina citri] --HMM score = 0.961 --Signal peptide length = 30 PredHel = 0 Topology = o len = 102 |
| XP_008474120.1 | uncharacterized protein LOC103511179 [Diaphorina citri] --HMM score = 0.976 --Signal peptide length = 21 PredHel = 0 Topology = o len = 119 |
| XP_017300687.1 | uncharacterized protein LOC103511954 [Diaphorina citri] --HMM score = 0.912 --Signal peptide length = 23 PredHel = 1 Topology = o6-28i len = 139 |
| XP_017300752.1 | uncharacterized protein LOC103512119 isoform X2 [Diaphorina citri] --HMM score = 0.991 --Signal peptide length = 28 PredHel = 1 Topology = i9-31o len = 83 |
| XP_008475221.1 | uncharacterized protein LOC103512248 [Diaphorina citri] --HMM score = 0.997 --Signal peptide length = 19 PredHel = 0 Topology = o len = 106 |
| XP_008475227.1 | uncharacterized protein LOC103512253 [Diaphorina citri] --HMM score = 0.951 --Signal peptide length = 22 PredHel = 0 Topology = o len = 164 |
| XP_008475409.1 | uncharacterized protein LOC103512422 [Diaphorina citri] --HMM score = 0.998 --Signal peptide length = 17 PredHel = 0 Topology = o len = 197 |
| XP_008475418.1 | uncharacterized protein LOC103512433 [Diaphorina citri] --HMM score = 0.972 --Signal peptide length = 25 PredHel = 0 Topology = o len = 115 |
| XP_008475639.1 | uncharacterized protein LOC103512644 isoform X2 [Diaphorina citri] --HMM score = 0.967 --Signal peptide length = 21 PredHel = 0 Topology = o len = 137 |
| XP_008475641.1 | uncharacterized protein LOC103512645 isoform X2 [Diaphorina citri] --HMM score = 0.967 --Signal peptide length = 21 PredHel = 0 Topology = o len = 137 |
| XP_017301155.1 | uncharacterized protein LOC103513080 [Diaphorina citri] --HMM score = 0.999 --Signal peptide length = 22 PredHel = 0 Topology = o len = 227 |
| XP_008476191.1 | uncharacterized protein LOC103513160 [Diaphorina citri] --HMM score = 1.000 --Signal peptide length = 25 PredHel = 1 Topology = i5-27o len = 112 |
| XP_008476330.1 | uncharacterized protein LOC103513290 [Diaphorina citri] --HMM score = 0.998 --Signal peptide length = 19 PredHel = 0 Topology = o len = 143 |
| XP_008476658.1 | uncharacterized protein LOC103513591 [Diaphorina citri] --HMM score = 0.997 --Signal peptide length = 22 PredHel = 0 Topology = o len = 228 |
| XP_008476773.1 | uncharacterized protein LOC103513701 [Diaphorina citri] --HMM score = 0.936 --Signal peptide length = 19 PredHel = 0 Topology = o len = 194 |
| XP_008477393.1 | uncharacterized protein LOC103514302 [Diaphorina citri] --HMM score = 0.997 --Signal peptide length = 22 PredHel = 1 Topology = o4-23i len = 130 |
| XP_008478010.2 | uncharacterized protein LOC103514871 [Diaphorina citri] --HMM score = 0.999 --Signal peptide length = 22 PredHel = 0 Topology = o len = 173 |
| XP_008478017.1 | uncharacterized protein LOC103514875 [Diaphorina citri] --HMM score = 0.992 --Signal peptide length = 21 PredHel = 0 Topology = o len = 230 |
| XP_017302085.1 | uncharacterized protein LOC103515145 [Diaphorina citri] --HMM score = 1.000 --Signal peptide length = 19 PredHel = 0 Topology = o len = 168 |
| XP_017302015.1 | uncharacterized protein LOC103515150, partial [Diaphorina citri] --HMM score = 0.990 --Signal peptide length = 28 PredHel = 0 Topology = o len = 179 |
| XP_008478400.1 | uncharacterized protein LOC103515249 [Diaphorina citri] --HMM score = 0.999 --Signal peptide length = 20 PredHel = 0 Topology = o len = 147 |
| XP_008478473.1 | uncharacterized protein LOC103515309 isoform X1 [Diaphorina citri] --HMM score = 0.999 --Signal peptide length = 17 PredHel = 0 Topology = o len = 132 |
| XP_008478472.1 | uncharacterized protein LOC103515309 isoform X2 [Diaphorina citri] --HMM score = 0.999 --Signal peptide length = 17 PredHel = 0 Topology = o len = 132 |
| XP_008478649.1 | uncharacterized protein LOC103515486 [Diaphorina citri] --HMM score = 1.000 --Signal peptide length = 23 PredHel = 0 Topology = o len = 172 |
| XP_008478682.1 | uncharacterized protein LOC103515524, partial [Diaphorina citri] --HMM score = 0.976 --Signal peptide length = 18 PredHel = 0 Topology = o len = 113 |
| XP_008478727.1 | uncharacterized protein LOC103515567 [Diaphorina citri] --HMM score = 0.986 --Signal peptide length = 23 PredHel = 0 Topology = o len = 137 |
| XP_008478809.2 | uncharacterized protein LOC103515652, partial [Diaphorina citri] --HMM score = 0.977 --Signal peptide length = 22 PredHel = 0 Topology = o len = 178 |
| XP_017302433.1 | uncharacterized protein LOC103516168 isoform X1 [Diaphorina citri] --HMM score = 1.000 --Signal peptide length = 23 PredHel = 0 Topology = o len = 176 |
| XP_017302434.1 | uncharacterized protein LOC103516168 isoform X2 [Diaphorina citri] --HMM score = 1.000 --Signal peptide length = 23 PredHel = 0 Topology = o len = 176 |
| XP_017302499.1 | uncharacterized protein LOC103516340 [Diaphorina citri] --HMM score = 1.000 --Signal peptide length = 19 PredHel = 0 Topology = o len = 134 |
| XP_008479535.1 | uncharacterized protein LOC103516350 [Diaphorina citri] --HMM score = 0.998 --Signal peptide length = 22 PredHel = 0 Topology = o len = 159 |
| XP_017302718.1 | uncharacterized protein LOC103516847 [Diaphorina citri] --HMM score = 0.999 --Signal peptide length = 20 PredHel = 0 Topology = o len = 203 |
| XP_008480359.1 | uncharacterized protein LOC103517116 [Diaphorina citri] --HMM score = 0.998 --Signal peptide length = 20 PredHel = 0 Topology = o len = 216 |
| XP_008480375.1 | uncharacterized protein LOC103517129 [Diaphorina citri] --HMM score = 0.966 --Signal peptide length = 29 PredHel = 0 Topology = o len = 192 |
| XP_008480572.1 | uncharacterized protein LOC103517322 [Diaphorina citri] --HMM score = 0.979 --Signal peptide length = 19 PredHel = 0 Topology = o len = 102 |
| XP_008481275.1 | uncharacterized protein LOC103518002 [Diaphorina citri] --HMM score = 0.984 --Signal peptide length = 22 PredHel = 0 Topology = o len = 177 |
| XP_008481410.1 | uncharacterized protein LOC103518133 [Diaphorina citri] --HMM score = 1.000 --Signal peptide length = 25 PredHel = 1 Topology = i7-29o len = 168 |
| XP_008481488.1 | uncharacterized protein LOC103518205 [Diaphorina citri] --HMM score = 0.999 --Signal peptide length = 21 PredHel = 0 Topology = o len = 129 |
| XP_008481512.1 | uncharacterized protein LOC103518230 [Diaphorina citri] --HMM score = 0.962 --Signal peptide length = 15 PredHel = 0 Topology = o len = 105 |
| XP_008481831.1 | uncharacterized protein LOC103518532 [Diaphorina citri] --HMM score = 0.978 --Signal peptide length = 23 PredHel = 0 Topology = o len = 126 |
| XP_008481832.1 | uncharacterized protein LOC103518533 [Diaphorina citri] --HMM score = 0.981 --Signal peptide length = 23 PredHel = 1 Topology = o5-27i len = 126 |
| XP_008482380.1 | uncharacterized protein LOC103519077 [Diaphorina citri] --HMM score = 0.974 --Signal peptide length = 34 PredHel = 1 Topology = i12-34o len = 120 |
| XP_008482381.1 | uncharacterized protein LOC103519077 [Diaphorina citri] --HMM score = 0.974 --Signal peptide length = 34 PredHel = 1 Topology = i12-34o len = 120 |
| XP_017303539.1 | uncharacterized protein LOC103519077 [Diaphorina citri] --HMM score = 0.974 --Signal peptide length = 34 PredHel = 1 Topology = i12-34o len = 120 |
| XP_008482489.1 | uncharacterized protein LOC103519182 [Diaphorina citri] --HMM score = 1.000 --Signal peptide length = 17 PredHel = 0 Topology = o len = 149 |
| XP_008482531.1 | uncharacterized protein LOC103519226 [Diaphorina citri] --HMM score = 0.998 --Signal peptide length = 20 PredHel = 0 Topology = o len = 127 |
| XP_008483107.1 | uncharacterized protein LOC103519797 [Diaphorina citri] --HMM score = 0.968 --Signal peptide length = 25 PredHel = 0 Topology = o len = 152 |
| XP_008483348.1 | uncharacterized protein LOC103520032, partial [Diaphorina citri] --HMM score = 0.989 --Signal peptide length = 25 PredHel = 1 Topology = i5-27o len = 107 |
| XP_008483947.1 | uncharacterized protein LOC103520623 isoform X1 [Diaphorina citri] --HMM score = 0.963 --Signal peptide length = 21 PredHel = 0 Topology = o len = 156 |
| XP_008483946.1 | uncharacterized protein LOC103520623 isoform X2 [Diaphorina citri] --HMM score = 0.957 --Signal peptide length = 21 PredHel = 0 Topology = o len = 156 |
| XP_008484013.1 | uncharacterized protein LOC103520692 [Diaphorina citri] --HMM score = 0.999 --Signal peptide length = 25 PredHel = 0 Topology = o len = 100 |
| XP_008484243.1 | uncharacterized protein LOC103520923, partial [Diaphorina citri] --HMM score = 0.989 --Signal peptide length = 25 PredHel = 1 Topology = i7-29o len = 86 |
| XP_008484322.1 | uncharacterized protein LOC103520998, partial [Diaphorina citri] --HMM score = 0.996 --Signal peptide length = 22 PredHel = 0 Topology = o len = 191 |
| XP_008484331.1 | uncharacterized protein LOC103521004 [Diaphorina citri] --HMM score = 0.998 --Signal peptide length = 21 PredHel = 0 Topology = o len = 125 |
| XP_008484388.1 | uncharacterized protein LOC103521057, partial [Diaphorina citri] --HMM score = 0.995 --Signal peptide length = 17 PredHel = 0 Topology = o len = 143 |
| XP_008485033.1 | uncharacterized protein LOC103521704 [Diaphorina citri] --HMM score = 0.981 --Signal peptide length = 23 PredHel = 0 Topology = o len = 234 |
| XP_008485252.1 | uncharacterized protein LOC103521922 [Diaphorina citri] --HMM score = 0.997 --Signal peptide length = 24 PredHel = 0 Topology = o len = 123 |
| XP_008485511.1 | uncharacterized protein LOC103522187 isoform X1 [Diaphorina citri] --HMM score = 0.984 --Signal peptide length = 24 PredHel = 0 Topology = o len = 134 |
| XP_008485512.1 | uncharacterized protein LOC103522187 isoform X2 [Diaphorina citri] --HMM score = 0.984 --Signal peptide length = 24 PredHel = 0 Topology = o len = 134 |
| XP_017304464.1 | uncharacterized protein LOC103522187 isoform X3 [Diaphorina citri] --HMM score = 0.984 --Signal peptide length = 24 PredHel = 0 Topology = o len = 134 |
| XP_008485549.1 | uncharacterized protein LOC103522225, partial [Diaphorina citri] --HMM score = 0.997 --Signal peptide length = 22 PredHel = 0 Topology = o len = 140 |
| XP_008485803.1 | uncharacterized protein LOC103522478, partial [Diaphorina citri] --HMM score = 0.927 --Signal peptide length = 32 PredHel = 0 Topology = o len = 113 |
| XP_008485893.1 | uncharacterized protein LOC103522571 [Diaphorina citri] --HMM score = 0.988 --Signal peptide length = 28 PredHel = 0 Topology = o len = 156 |
| XP_008486403.1 | uncharacterized protein LOC103523112 [Diaphorina citri] --HMM score = 0.996 --Signal peptide length = 22 PredHel = 0 Topology = o len = 128 |
| XP_008486405.1 | uncharacterized protein LOC103523114 [Diaphorina citri] --HMM score = 0.992 --Signal peptide length = 21 PredHel = 0 Topology = o len = 167 |
| XP_017304775.1 | uncharacterized protein LOC103523676, partial [Diaphorina citri] --HMM score = 0.979 --Signal peptide length = 23 PredHel = 0 Topology = o len = 231 |
| XP_008487473.1 | uncharacterized protein LOC103524243 [Diaphorina citri] --HMM score = 0.921 --Signal peptide length = 20 PredHel = 0 Topology = o len = 108 |
| XP_008487527.1 | uncharacterized protein LOC103524291 [Diaphorina citri] --HMM score = 0.997 --Signal peptide length = 22 PredHel = 0 Topology = o len = 193 |
| XP_017305073.1 | uncharacterized protein LOC103524376 [Diaphorina citri] --HMM score = 0.916 --Signal peptide length = 16 PredHel = 0 Topology = o len = 110 |
| XP_008487632.1 | uncharacterized protein LOC103524398 [Diaphorina citri] --HMM score = 0.997 --Signal peptide length = 25 PredHel = 1 Topology = o4-26i len = 131 |
| XP_008487825.1 | uncharacterized protein LOC103524576 [Diaphorina citri] --HMM score = 0.997 --Signal peptide length = 23 PredHel = 0 Topology = o len = 145 |
| XP_008488071.1 | uncharacterized protein LOC103524816 [Diaphorina citri] --HMM score = 0.972 --Signal peptide length = 20 PredHel = 0 Topology = o len = 173 |
| XP_008488275.1 | uncharacterized protein LOC103525009 [Diaphorina citri] --HMM score = 1.000 --Signal peptide length = 19 PredHel = 0 Topology = o len = 204 |
| XP_017298578.1 | uncharacterized protein LOC108251836 [Diaphorina citri] --HMM score = 0.992 --Signal peptide length = 22 PredHel = 0 Topology = o len = 210 |
| XP_017298676.1 | uncharacterized protein LOC108252130 [Diaphorina citri] --HMM score = 0.996 --Signal peptide length = 18 PredHel = 0 Topology = o len = 144 |
| XP_017298791.1 | uncharacterized protein LOC108252162 [Diaphorina citri] --HMM score = 0.982 --Signal peptide length = 28 PredHel = 0 Topology = o len = 146 |
| XP_017298792.1 | uncharacterized protein LOC108252163 [Diaphorina citri] --HMM score = 0.982 --Signal peptide length = 28 PredHel = 0 Topology = o len = 146 |
| XP_017298994.1 | uncharacterized protein LOC108252218 [Diaphorina citri] --HMM score = 0.996 --Signal peptide length = 23 PredHel = 1 Topology = o4-21i len = 135 |
| XP_017299295.1 | uncharacterized protein LOC108252313 [Diaphorina citri] --HMM score = 1.000 --Signal peptide length = 20 PredHel = 0 Topology = o len = 114 |
| XP_017300042.1 | uncharacterized protein LOC108252542 [Diaphorina citri] --HMM score = 0.995 --Signal peptide length = 24 PredHel = 1 Topology = i7-29o len = 64 |
| XP_017301248.1 | uncharacterized protein LOC108252888 [Diaphorina citri] --HMM score = 0.997 --Signal peptide length = 23 PredHel = 0 Topology = o len = 122 |
| XP_017302258.1 | uncharacterized protein LOC108253225 isoform X1 [Diaphorina citri] --HMM score = 0.998 --Signal peptide length = 21 PredHel = 0 Topology = o len = 106 |
| XP_017302259.1 | uncharacterized protein LOC108253225 isoform X2 [Diaphorina citri] --HMM score = 0.998 --Signal peptide length = 21 PredHel = 0 Topology = o len = 95 |
| XP_017302492.1 | uncharacterized protein LOC108253299 [Diaphorina citri] --HMM score = 0.983 --Signal peptide length = 24 PredHel = 0 Topology = o len = 143 |
| XP_017302533.1 | uncharacterized protein LOC108253312 [Diaphorina citri] --HMM score = 0.987 --Signal peptide length = 28 PredHel = 0 Topology = o len = 101 |
| XP_017302851.1 | uncharacterized protein LOC108253431 [Diaphorina citri] --HMM score = 0.994 --Signal peptide length = 24 PredHel = 1 Topology = o10-32i len = 113 |
| XP_017302907.1 | uncharacterized protein LOC108253446 [Diaphorina citri] --HMM score = 0.963 --Signal peptide length = 21 PredHel = 0 Topology = o len = 193 |
| XP_017303819.1 | uncharacterized protein LOC108253753 isoform X1 [Diaphorina citri] --HMM score = 0.949 --Signal peptide length = 24 PredHel = 1 Topology = i7-29o len = 124 |
| XP_017303820.1 | uncharacterized protein LOC108253753 isoform X2 [Diaphorina citri] --HMM score = 0.949 --Signal peptide length = 24 PredHel = 1 Topology = i7-29o len = 124 |
| XP_017304064.1 | uncharacterized protein LOC108253867 [Diaphorina citri] --HMM score = 0.999 --Signal peptide length = 22 PredHel = 0 Topology = o len = 127 |
| XP_008483080.1 | UPF0729 protein AAEL015238 [Diaphorina citri] --HMM score = 0.938 --Signal peptide length = 21 PredHel = 0 Topology = o len = 92 |
| XP_008488184.1 | vasotab [Diaphorina citri] --HMM score = 0.999 --Signal peptide length = 21 PredHel = 1 Topology = o4-26i len = 101 |
| XP_017303584.1 | viresin-like [Diaphorina citri] --HMM score = 0.999 --Signal peptide length = 19 PredHel = 1 Topology = i5-24o len = 67 |
| XP_008482275.1 | waprin-Thr1 [Diaphorina citri] --HMM score = 0.999 --Signal peptide length = 20 PredHel = 0 Topology = o len = 107 |
| XP_017303632.1 | waprin-Thr1 [Diaphorina citri] --HMM score = 0.999 --Signal peptide length = 20 PredHel = 0 Topology = o len = 107 |
| XP_008473202.1 | yrdC domain-containing protein, mitochondrial [Diaphorina citri] --HMM score = 0.958 --Signal peptide length = 20 PredHel = 0 Topology = o len = 245 |

**Results**

Our goal was to identify secreted proteins, linked to salivation events from genomic data of *D. citri*. We used all predicted gene encoding proteins from version 1.1 to conduct an in-depth analyses search of proteins with N-terminal signal peptides. The predicted *Diaphorina citri* secretome contains a total of 1,247 proteins which is equivalent to 6% of the proteome (20,622 proteins). We identified a group of 246 small secreted protein (SCR) effector candidates out of the total 1,247 set by filtering out secreted proteins with more than 200 amino acids length. The full-length sequences of 246 secreted proteins were grouped in 11 tribes with more than 2 members and 202 singleton tribes. The largest tribe was annotated as “ejaculatory bulb-specific protein 3-like or EbpII family” (pfam domain IPR005055) which shares features common vertebrate odorant binding proteins (OBPs) with potential roles in insect immunity (Tsujimoto et al., 2017). OBP are conserved in the insect phylum these are not restricted to olfaction and are likely to be involved in broader physiological functions (Foret and Maleszka, 2006), as they can be expressed in non-olfactory tissues accessory glands tissues that form part of the salivary gland organs in insects (Paesen and Happ, 1995). The singleton tribes included a small cysteine rich (SCR) secreted protein annotated as “basic salivary proline-rich protein 3-like (XP_008480568.1)” which is in accordance with the hypothesis that secreted proteins are associated with salivary gland tissues. Five secreted proteins were annotated as inhibitors with putative catalytic functions of proteolytic enzymes of host proteases (Polanowski and Wilusz, 1996; Boigegrain et al., 2000). Among the annotated inhibitors proteins, there were identified cysteine inhibitors with cystatin-like domains (XP_017303134.1, XP_008481260.1, XP_008481261.1 and XP_008487197.1) (pfam00031, cd00042) and serine inhibitors with Kazal-like domain proteins (XP_017301918.) (Follistatin-like SPARC, cd01328). Also a secreted chitin-binding domain protein (CBP) (XP_008480568.1) “chondroitin proteoglycan 2-like” which belong to family present in various species with roles in chitin synthesis and degradation (Zhu et al., 2008) was identified. CPBs have been shown to participate in growth, maturation and repair during mechanical injuries or in immune responses against pathogens. Parasitic wasps encode for CBP-like proteins, that may be injected into the host to help wound healing/repair of the host exoskeleton particularly it is suggested a role in the flexible peritrophic matrix or membrane (PM) which protects the midgut epithelium from mechanical and biological injuries caused by dietary components and pathogens, and also serves as a digestive chamber, regulating the permeability and localization of enzymes and digestion products (Zhu et al., 2015).

**References**

1. Boigegrain, R.A., Pugniere, M., Paroutaud, P., Castro, B., and Brehelin, M. (2000). Low molecular weight serine protease inhibitors from insects are proteins with highly conserved sequences. *Insect Biochem Mol Biol* 30**,** 145-152.
2. Bos, J.I., Prince, D., Pitino, M., Maffei, M.E., Win, J., and Hogenhout, S.A. (2010). A functional genomics approach identifies candidate effectors from the aphid species *Myzus persicae* (green peach aphid). *PLoS Genet* 6**,** e1001216.
3. Chenna, R., Sugawara, H., Koike, T., Lopez, R., Gibson, T.J., Higgins, D.G., and Thompson, J.D. (2003). Multiple sequence alignment with the Clustal series of programs. *Nucleic Acids Res* 31**,** 3497-3500.
4. Emanuelsson, O., Nielsen, H., and Von Heijne, G. (1999). ChloroP, a neural network-based method for predicting chloroplast transit peptides and their cleavage sites. *Protein Sci* 8**,** 978-984.
5. Enright, A.J., Van Dongen, S., and Ouzounis, C.A. (2002). An efficient algorithm for large-scale detection of protein families. *Nucleic Acids Res* 30**,** 1575-1584.
6. Foret, S., and Maleszka, R. (2006). Function and evolution of a gene family encoding odorant binding-like proteins in a social insect, the honey bee (*Apis mellifera*). *Genome Res* 16**,** 1404-1413.
7. Jaouannet, M., Rodriguez, P.A., Thorpe, P., Lenoir, C.J., Macleod, R., Escudero-Martinez, C., and Bos, J.I. (2014). Plant immunity in plant-aphid interactions. *Front Plant Sci* 5**,** 663.
8. Krogh, A., Larsson, B., Von Heijne, G., and Sonnhammer, E.L. (2001). Predicting transmembrane protein topology with a hidden Markov model: application to complete genomes. *J Mol Biol* 305**,** 567-580.
9. Moreno, A., Garzo, E., Fernandez-Mata, G., Kassem, M., Aranda, M.A., and Fereres, A. (2012). Aphids secrete watery saliva into plant tissues from the onset of stylet penetration. *Entomol. Exp. Appl.* 139**,** 145–153.
10. Nguyen Ba, A.N., Pogoutse, A., Provart, N., and Moses, A.M. (2009). NLStradamus: a simple Hidden Markov Model for nuclear localization signal prediction. *BMC Bioinformatics* 10**,** 202.
11. Nielsen, H., Engelbrecht, J., Brunak, S., and Von Heijne, G. (1997). A neural network method for identification of prokaryotic and eukaryotic signal peptides and prediction of their cleavage sites. *Int J Neural Syst* 8**,** 581-599.
12. Paesen, G.C., and Happ, G.M. (1995). The B proteins secreted by the tubular accessory sex glands of the male mealworm beetle, *Tenebrio molitor,* have sequence similarity to moth pheromone-binding proteins. *Insect Biochem Mol Biol* 25**,** 401-408.
13. Polanowski, A., and Wilusz, T. (1996). Serine proteinase inhibitors from insect hemolymph. *Acta Biochim Pol* 43**,** 445-453.
14. Rodriguez, P.A., and Bos, J.I. (2013). Toward understanding the role of aphid effectors in plant infestation. *Mol Plant Microbe Interact* 26**,** 25-30.
15. Shang, Y., Xiao, G., Zheng, P., Cen, K., Zhan, S., and Wang, C. (2016). Divergent and convergent evolution of fungal pathogenicity. *Genome Biol Evol* 8**,** 1374-1387.
16. Tsujimoto, H., Hanley, K.A., Sundararajan, A., Devitt, N.P., Schilkey, F.D., and Hansen, I.A. (2017). Dengue virus serotype 2 infection alters midgut and carcass gene expression in the Asian tiger mosquito, *Aedes albopictus*. *PLoS One* 12**,** e0171345.
17. Zhu, Q., Arakane, Y., Banerjee, D., Beeman, R.W., Kramer, K.J., and Muthukrishnan, S. (2008). Domain organization and phylogenetic analysis of the chitinase-like family of proteins in three species of insects. *Insect Biochem Mol Biol* 38**,** 452-466.
18. Zhu, Y., Ye, X.H., Liu, Y., Yan, Z.C., Stanley, D., Ye, G.Y., and Fang, Q. (2015). A venom gland extracellular chitin-binding-like protein from pupal endoparasitoid wasps, Pteromalus puparum, selectively binds chitin. *Toxins (Basel)* 7**,** 5098-5113.

# Supplementary Note 38: Heat shock proteins

**Introduction**

Heat shock proteins (hsps) are molecular chaperones that facilitates protein folding and stabilization, act as proteases, and aiding the degradation of damaged proteins (Gerardo et al 2010; Pockley 2003). In various organisms, hsps are produced in response to stress and may serve as signaling proteins during immune responses (Henderson 2006; Pockley 2003).

Hsps are grouped into several families, including small hsps, hsp60, hsp70, hsp90, and hsp100 (Pockley, 2003). These hsp families involve many different functions. Heat shock protein 90 (hsp90) was shown to be upregulated in *Galleria mellonella* after a mild heat shock and microbial infection, suggesting that Hsp90 may be involved in both insect immunity and heat shock pathways (Wojda and Jakubowicz 2007). However, compared to the symbiotic relationship between *Acyrthosiphon pisum* and *Serratia symbiotica*, *A. pisum* hsp gene expression induces little change (Burke and Moran, 2011), showing hsps may not affect insect immunity in this example.

**Methods**

Hemipteran hsps orthologs were collected from NCBI and i5k Blast. The orthologs were used to blat against the *Diaphorina citri*, Asian citrus psyllid (ACP), genome. Identification and annotation of hsps was performed in WebApollo where RNA sequence data (adult, nymph, and egg) supported the manually curated gene models. BLASTp was used to confirm accuracy of the annotated hsps and ExPASy was used to identify molecular weight.

Hsps orthologs were collected through i5k Blast and a limited NCBI search to include different insect orders. Local pairwise amino acid sequence alignments were done in BLASTp between *D. citri*, *Acyrthosiphon pisum, Oncopeltus fasciatus, Halyomorpha halys, Cimex lectularius, Gerris buenoi, Homalodisca vitripennis,* and *Pachypsylla venusta.* Multiple sequence alignments of ACP and orthologous insect hsps sequences was performed in MEGA6 using ClustalW (Tamura et al 2003). The phylogenetic analysis of the hsps included, constructing a midpoint rooted neighbor-joining tree in MEGA6 using p-distance to show evolutionary distance and 1,000 bootstrap replicates.

Table 1: All the hsps found in *D. citri* genome. Table shows all hsp, names based on NCBI Gnomon (^A^) with BLASTp matches and domain organization, functional description from Uniprot (^B^) for each gene, and corresponding molecular weight and protein length.

| ***D. citri* Hsps ^A^** | **BLASTP match (organism, bit score)** | **Function^B^** | **MW (kDa)** | **Protein Length** | **Domains** |
| --- | --- | --- | --- | --- | --- |
| 10 kDa Hsp,  mitochondrial (2) | 11.1 kDa Hsp  (*Oxya chinensis*, 114) | Chaperone that binds to hsp60 to enhance protein folding | 12 | 111 residues | CPN10, GroES, oligomerization interface, mobile loop, roof hairpin |
| Hsp 23 | Heat shock protein  (*Aedes aegypt,* 67) | No known function | 17 | 113 residues | Metazoan_ACD, HSP20, IbpA |
| Hsp beta-1  (2 isoforms) | Hsp beta-1  *(Musca domestica,* 253) | Involved in stress resistance and actin organization | 21 | RA= 187 residues RB= 151 residues | metazoan ACD, IbpA, hsp20, ACD hsps p23 superfamily, putative dimer interface |
| Alpha-crystallin A chain | Alpha-crystallin A chain  (*Halyomorpha halys*, 204) | has chaperone-like activity | 21 | 189 residues | metazoan ACD, IbpA, hsp20, ACD hsps p23 superfamily |
| Hsp 60 kDa,  mitochondrial | 60 kDa Hsp, mitochondrial  (*Lasius niger*, 720) | Folds/transports newly imported proteins into the mitochondria | 45 | 421 residues | GroEL, chaperonin like superfamily, thermosome_arch |
| Hsp 68 (partial) | - | Stabilizes existing proteins against aggregation and mediates the folding of newly translated proteins | - | - | - |
| Hsp 70 A1 (4) | Hsp 70  (*Bemisia tabaci*, 1118) |  | 69 | 626,629, 629, 629 residues | NBD of HSPA1, NBD of sugar kinase hsp 70 actin superfamily, hsp70, DnaK, SBD interface, BAG/hsp70 and EF/hsp70 interaction site |
| Hsp 70 kDa  protein 14 (partial) | Hsp 70 kDa protein 14  (*A. pisum*, 84.7) |  | 23 | 213 residues | NBD of sugar kinase hsp 70 actin superfamily |
| Hsp 75 kDa,  mitochondrial (partial) | Hsp 75 kDa, mitochondrial  (*Athalia rosae*, 577) | Chaperone with ATP activity | 46 | 402 residues | HSP90 |
| Hsp 83 | Hsp 90  (*Laodelphax striatella,* 1226) | Molecular chaperone of specific proteins of the cell cycle and signal transduction | 83 | 723 residues | Hsp90 superfamily, HATPase_c, ATP binding site, Mg2+ binding site, G-X-P motif |
| Hsp 81-3 | Hsp 83  (*H. saltator*, 444) |  | 74 | 642 residues | Hsp90 superfamily, HATPase_c, ATP binding site, Mg2+ binding site, G-X-P motif |
| Hsp 83 | Hsp90 (Nilaparvata lugens, 903) |  | 64 | 553 residues | Hsp90 and superfamily |
| Hsp 90 (partial) | - | - | - | - | - |
| 97 kDa Hsp | 97 kDa Hsp  (*Amyelois transitella*, 733) | No known function | 85 | 763 residues | Hsp70, DnaK, NBD sugar kinase hsp 70 superfamily, SBD interface, hsp70 interaction site |
| Hsp 105 kDa (partial) | - | Chaperone with ATP activity | - | - | - |

Table 2: Pairwise Alignment of *D. citri* hsps with related organisms in Hemiptera. Sequence % identity, (sequence % similiarity), and [bit score]. No sequence found for the organism represented by ( - ), and values for alpha-crystallin represent query coverage of the domain only.

| ***Diaphorina citri*** | **Hsp 10** | **Hsp 23** | **Hsp β / α-crystallin** | **Hsp 60** | **Hsp 70** | **Hsp 83** |
| --- | --- | --- | --- | --- | --- | --- |
| ***Acyrthosiphon pisum*** | 45% (76%) [96.3] | - | - / 72% (82%) [261] | 77% (88%) [633] | 84% (91%) [1107] | 82% (91%) [1156] |
| ***Oncopeltus fasciatus*** | 58% (81%) [112] | 31% (58%) [50.4] | - / 63% (73%) [143] | 87% (92%) [712] | 79% (88%) [1085] | 84% (93%) [1185] |
| ***Halyomorpha halys*** | 58% (80%) [112] | 34% (57%) [46.6] | - / 87% (94%) [193] | 88% (93%) [719] | 84% (92%) [1118] | 84% (92%) [1174] |
| ***Cimex lectularius*** | 58% (79%) [111] | 34% (63%) [52] | - / 68% (79%) [240] | 87% (94%) [712] | 84% (92%) [1114] | 84% (92%) [1161] |
| ***Gerris buenoi*** | 55% (80%) [113] | 30% (47%) [48.9] | - / 60% (74%) [136] | 85% (93%) [701] | 82% (91%) [1084] | 79% (87%) [952] |
| ***Homalodisca vitripennis*** | 40% (61%) [93.6] | 33% (58%) [49.7] | - / 94% (97%) [123] | 74% (80%) [679] | 85% (93%) [1130] | 84% (92%) [1174] |
| ***Pachypsylla venusta*** | - | 30% (61%) [42] | - / 40% (70%) [65.9] | 79% (83%) [644] | 89% (94%) [1065] | 89% (96%) [1247] |

Table 3: Number of heat shock proteins in *D. citri* and related organisms.

| Organism | # of hsps |
| --- | --- |
| *Diaphorina citri* | 17 |
| *Acyrthosiphon pisum* | 15 |
| *Cimex lectularius* | 15 |
| *D. melanogaster* | 24 |
| *Aedes aegypti* | 23 |
| *Anopheles gambiae* | 27 |
| *Culex quinquefasciatus* | 22 |

**Results and Discussion**

A total of 17 hsps, representing five hsp families, were identified and manually curated within the ACP genome (Table 1). Only 15 hsps were found in the closest relative of *D. citri*, *A. pisum* (Gerardo et al 2010) (Table 3). The naming convention is based on NCBI Gnomon. Misassembly within the ACP genome prevented annotation of a couple hsps, including hsp 68 and hsp 105 kDa. All hsp 70 genes were found in a cluster together in a particular scaffold (WebApollo accession: gi|645503904|ref|NW_007378480.1|), the gene structure were similar between them containing three exons. Two hsp 10 were found in the ACP genome and were 100% identical. *A. pisum* also has two hsp 10 but differ in only two amino acids (NCBI accessions: NP_001119666 & XP_003245465).

In the pairwise alignment analysis (Table 2), hsps 60, 70, and 83 have an identity of 74% or greater between the ACP and related hemipterans compared to hsps 10 and 23 which have an identity of 58% or less. Hsp β-1 was not found in other hemipterans based on BLASTp searches. However, BLASTp searches limited to hemipterans did return close matches to α-crystallin. Pairwise alignment of Hsp B-1 and α-crystallin showed a greater than 70% similarity, but only within the α-crystallin domain only.

*D. citri* hsps 10, 70, and 90 were analyzed with related insects hsps from the orders Isoptera, Hymenoptera, Coleoptera, and Diptera by constructing neighbor-joining trees (Figure 1, 2, 3). The psyllid hsps were consistently grouped with other hemipteran hsps, further supporting the manually curated hsps.


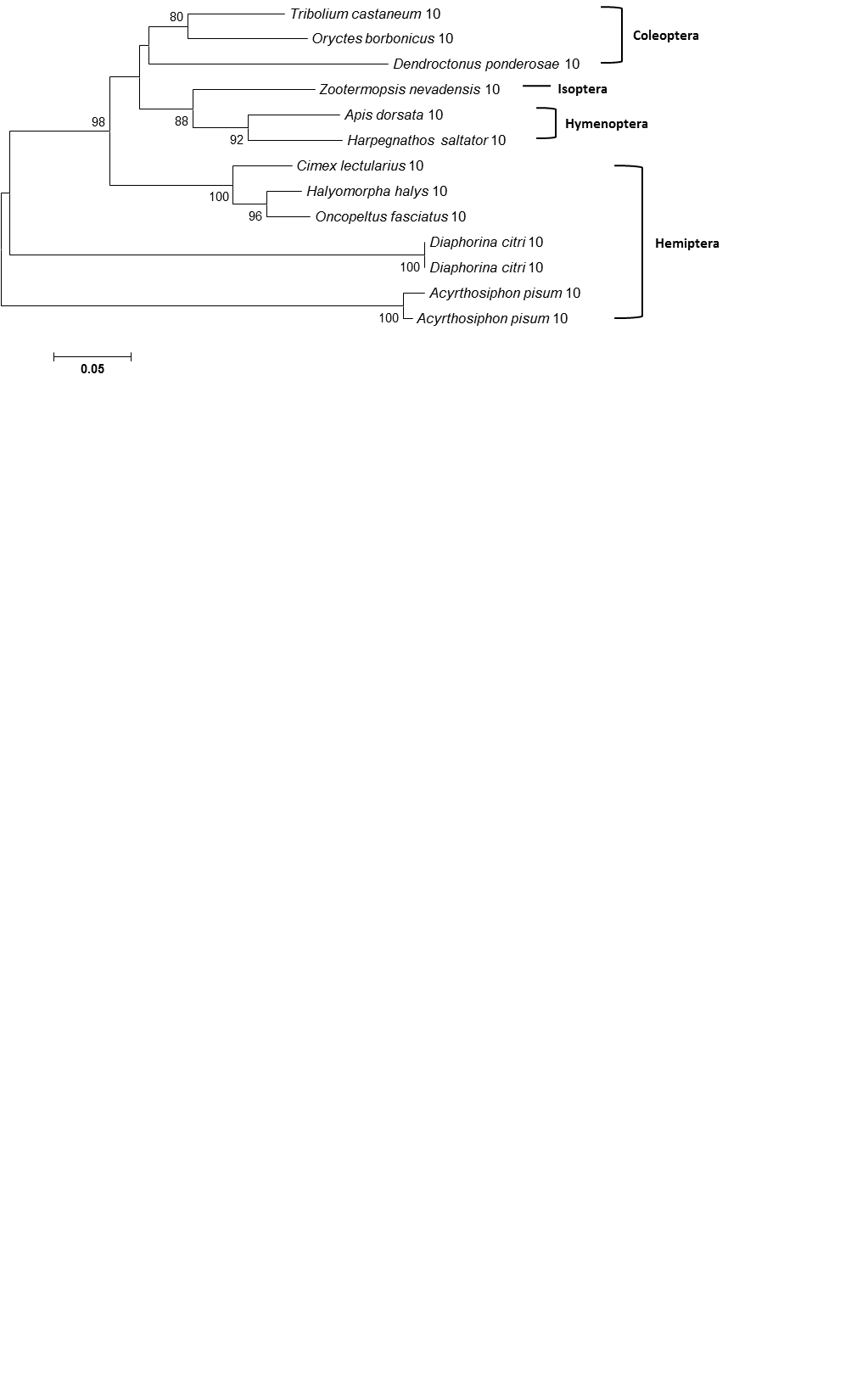


Figure 1: Neighbor-joining tree of 13 hsps 10 was constructed in MEGA6 using 1000 bootstrap replications showing 80% and greater, and the p-distance method was used to show evolutionary distance. A total of 100 amino acid positions were analyzed for the tree. NCBI accession: *T. castaneum* [XP_975179.2], *O. borbonicus* [KRT79990.1], *D. ponderosae* [ENN71973], *Z. nevadensis* [KDR14059.1], *A. dorsata* [XP_006622783.1], *H. saltator* [EFN79770.1], *C. lectularius* [XP_014258406.1], *H. halys* [XP_014273034.1], *A. pisum* [XP_003245465.1 & NP_001119666.1]. i5k accession: *O. fasciatus* [OFAS007006-RA].


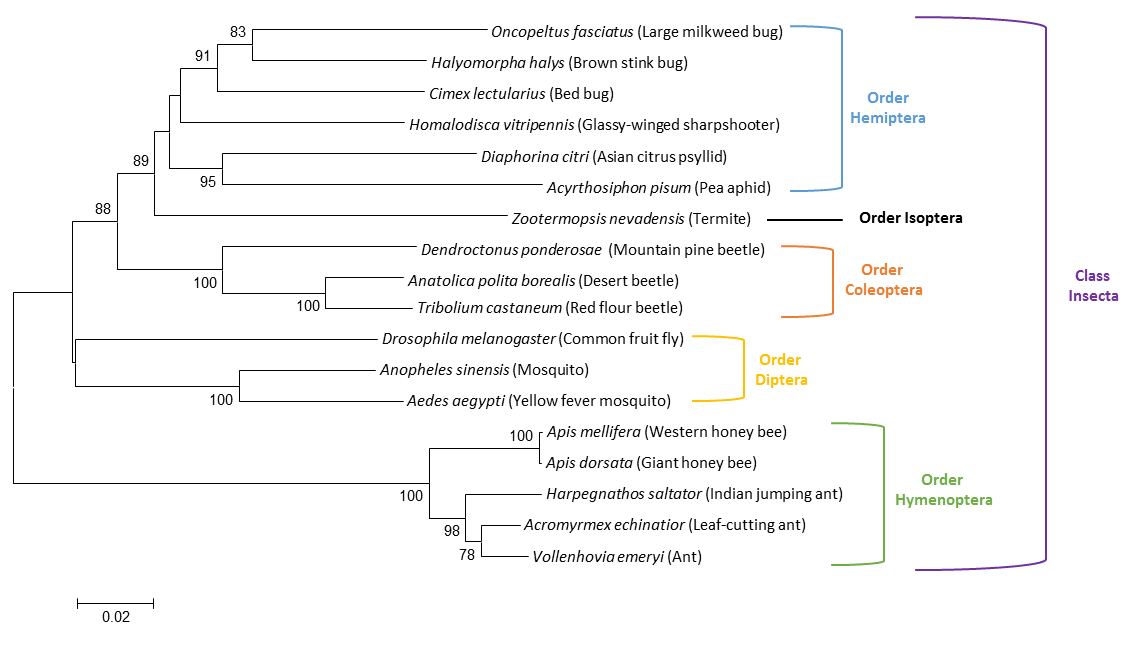


Figure 2: Neighbor-joining tree of the 18 hsps 70 was constructed in MEGA6 using 1000 bootstrap replications showing 78% and greater, and the p-distance method was used to show evolutionary distance. A total of 621 amino acid positions were analyzed for the tree. The organisms are accompanied with common name. NCBI accession: *H. halys* [XP_014293117.1], *C. lectularius* [XP_014239635.1], *A. pisum* [XP_001945786.2], *Z. nevadensis* [KDR08926.1], *D. ponderosae* [ENN76738.1], *A. polita borealis* [ABQ39970.1], *D. melanogaster* [AAG26893.1], *A. sinensis* [KFB46250.1], *A. aegypti* [ACJ64194.1], *A. mellifera* [NP_001153544.1], *A. dorsata* [XP_006620321.1], *H. saltator* [XP_011153497.1], *A. echinatior* [XP_011059681.1], *V. emeryi* [XP_011862691.1]. i5k accession: *O. fasciatus* [OFAS004738-RA], *H. vitripennis* [HVIT001451-PA].

**
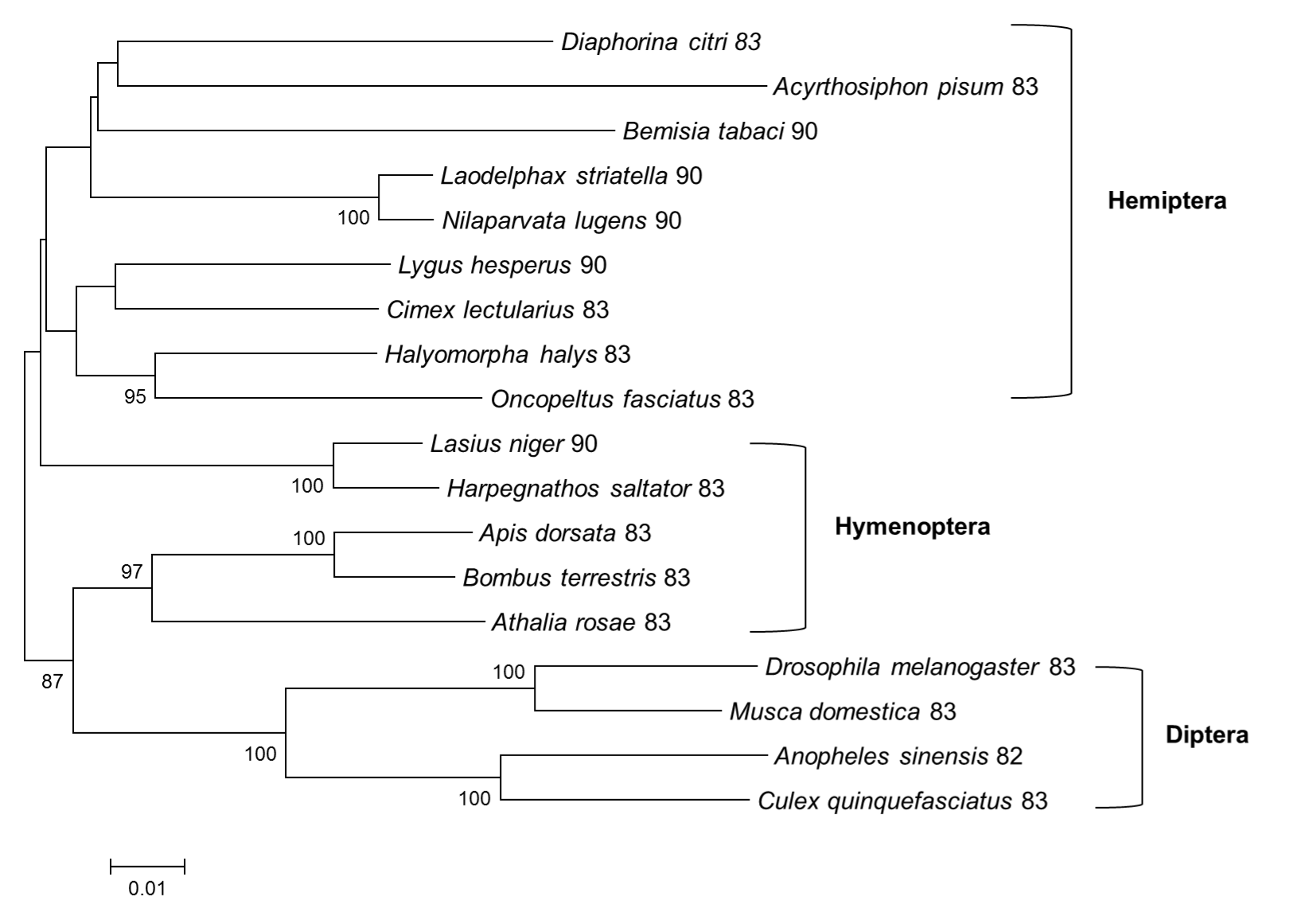
**

Figure 3: Neighbor-joining tree of the 18 hsps 83/90 was constructed in MEGA6 using 1000 bootstrap replications showing 87% and greater, and the p-distance method was used to show evolutionary distance. A total of 684 amino acid positions were analyzed for the tree. NCBI accession: *A. pisum* [XP_001943172.1], *B. tabaci* [AAZ17403.1], *L. striatella* [AHB63833.1], *N. lugens* [ADE34169.1], *L. hesperus* [AFX84559.1], *C. lectularius* [XP_014239772.1], *H. halys* [XP_014284945.1], *L. niger* [KMQ95531.1], *H. saltator* [XP_011154034.1], *A. dorsata* [XP_006616995.1], B. terrestris [XP_003393129.2], A. rosae [XP_012250991.1], *D. melanogaster* [NP_523899.1], *M. domestica* [XP_005176932], *A. sinensis* [KFB48798.1], *C. quinquefasciatus* [EDS36267.1]. i5k accession: *O. fasciatus* [OFAS001265-RA].

**References**

1. Gerardo NM, et al. 2010 Feb. Immunity and other defenses in pea aphids, *Acyrthosiphon pisum.* Genome Biology [Internet]. [cited 2016 May 17];1-17. PMID: 20178569
2. Tamura K., Stecher G., Peterson D., Filipski A., and Kumar S. (2013). MEGA6: Molecular Evolutionary Genetics Analysis version 6.0. *Molecular Biology and Evolution* 30: 2725-2729.
3. Pockley AG. 2003. Heat shock proteins as regulators of the immune response. The Lancet [Internet]. 362;469-476
4. Henderson B, Allan E, Coate S ARM: Stress wars: the direct role of host and bacterial molecular chaperones in bacterial infection. Infect Immun 2006, 74:3693-3706.
5. Wojda I, Jakubowicz T. Humoral immune response upon mild heat-shock conditions in Galleria mellonella larvae. J. Insect Physiol. 53: 1134- 1144, 2007.
6. Burke GR, Moran NA. Responses of the pea aphid transcriptome to infection by facultative symbionts. Insect Mol. Biol. 20: 357-365, 2011.

# Supplementary Note 39: Rab proteins

**Introduction**

Rabs are a gene family of small GTPases that act as molecular switches which regulate vesicle formation, movement, and merging (Pereira-Leal and Seabra 2001). They are involved in many key processes within all cells. They play a part in the transportation of material to and from the plasma membrane (exocytosis and endocytosis) (Seabra at al. 2002). They participate in the transport v between organelles, like the ER, Golgi apparatus, endosome, or phagosome, as seen during phagocytosis and gene expression (Seabra at al. 2002). The Rabs are phylogenetically assorted into functional groups based on their conservation (Pereira-Leal and Seabra 2001). What makes the sequence specific for Rabs are two switch regions, G box motifs (1-5), Family motifs (F1-5), and Subfamily motifs (SF1-5) (Pereira-Leal and Seabra 2000). Rabs typically consist of a domain called a GTP- binding domain. Rabs are suspected to be conserved in all branches of eukaryotes in one way or another since the last eukaryotic common ancestor (LECA) (Klopper et al. 2012).

**Methods**

The annotation process began with using at least three Rab sequences from closely related insects collected from the NCBI database and screening the psyllid’s genome for any predicted models. The first search tool used was BLAT from Web Apollo then BLAST from I5K and the MCOT database for the psyllid. Using the available evidence tracks in Web Apollo the found predictions were manually annotated, resulting in 21 different Rabs from *D. citri* being identified including isoforms.

Following manual annotation, the models were verified using a reciprocal BLASTP search in NCBI. Orthologs from *H. sapiens, D. melanogaster, C. elegans, S. cerevisiae, and S. pombe* were collected from the supplementary materials of Pereira-Leal and Seabra 2001. MUSCLE with default settings was used to make multiple sequence alignments of the Rab protein sequences from the previously mentioned orthologs and *D. citri*. MEGA7 was used to construct a phylogenetic tree. The tree was neighbor-joining and used the Poisson Model, pairwise deletion, and 1,000 bootstrap replicate settings. Functional groups were labeled based on Figure 4 from Pereira-Leal and Seabra 2001 and Figure 3 from Klopper et al. 2012.

Table 1: This is a list of the Rab genes found in *D.* *citri* and the functional group they fall into, as well as the ungrouped, based on phylogeny. The purpose associated with the groups is also given (Pereira-Leal and Seabra 2001; Ackers et al. 2005).

| **Rab genes found in *Diaphorina citri* and their functional groups** | | | |
| --- | --- | --- | --- |
| **Functional Group** | **Group members** | ***D. citri* Rab family members** | **Function** |
| I | 1,35 | 1A | Exocytosis |
| II | 2,4,11,14,25 | 2,11A,14 | Endocytosis and recycling |
| III | 3,26,27,37 | 3,27A | Regulated exocytosis |
| IV | 19,30 | 19,30 | Golgi related |
| V | 5,21,22 | 21,22B | Endocytosis |
| VI | 6,41 | 6 | Retrogade Golgi transport |
| VII | 7,9 | NA | Late endocytosis |
| VIII | 8,10,13 | 10, 13A, 13B, 13C, 13D | Golgi related |
| Ungrouped | - | L3,23,28,32, 40 | unknown |

Table 2: Rab genes founds in *D. citri* (column 1), highest BLASTP result other than *D. citri* (column 2), corresponding E values (column 3), and their functional groups (column4).

| **Rab genes found in *Diaphorina citri*** | | | |
| --- | --- | --- | --- |
| **Gene** | **BLAST result** | | **Functional Group** |
|  | Name | E value |  |
| DcRab1A | ras-related protein Rab-1A [Tribolium castaneum] | 3.00E-85 | I |
| DcRab11A | ras-related protein Rab-11A [Trichogramma pretiosum] | 3.00E-115 | II |
| DcRab14 | Rab14 [Macrobrachium rosenbergii] | 4.00E-125 | II |
| DcRab2 | ras-related protein Rab-2A isoform X2 [Bactrocera oleae] | 3.00E-92 | II |
| DcRabL3 | rab-like protein 3 isoform X1 [Athalia rosae] | 4.00E-88 | III |
| DcRab27A | ras-related protein Rab-27A [Cerapachys biroi] | 4.00E-38 | III |
| DcRab3 | ras-related protein Rab-3 isoform X1 [Halyomorpha halys] | 7.00E-145 | III |
| DcRab30 | Ras-related protein Rab-30 [Zootermopsis nevadensis] | 2.00E-122 | IV |
| DcRab21 | Ras-related protein Rab-21 [Zootermopsis nevadensis] | 9.00E-122 | V |
| DcRab19 | putative Rab-43-like protein ENSP00000330714 [Ceratitis capitata] | 2.00E-84 | VI |
| DcRab6 | ras-related protein Rab6 isoform X2 [Acromyrmex echinatior] | 2.00E-100 | VI |
| DcRab10 | ras-related protein Rab-10 [Halyomorpha halys] | 6.00E-77 | VIII |
| DcRab13A | Ras-related protein Rab-8B [Zootermopsis nevadensis] | 6.00E-97 | VIII |
| DcRab22B | ras-related protein Rab-21 isoform X2 [Halyomorpha halys] | 1.00E-97 | Ungrouped |
| DcRab23 | Ras-related protein Rab-23 [Zootermopsis nevadensis] | 5.00E-77 | Ungrouped |
| DcRab28 | RAB-28, putative [Pediculus humanus corporis] | 7.00E-25 | Ungrouped |
| DcRab32 | ras-related protein Rab-32 isoform X4 [Bombus terrestris] | 9.00E-112 | Ungrouped |
| DcRab40 | Ras-related protein Rab-40C [Zootermopsis nevadensis] | 2.00E-149 | Ungrouped |


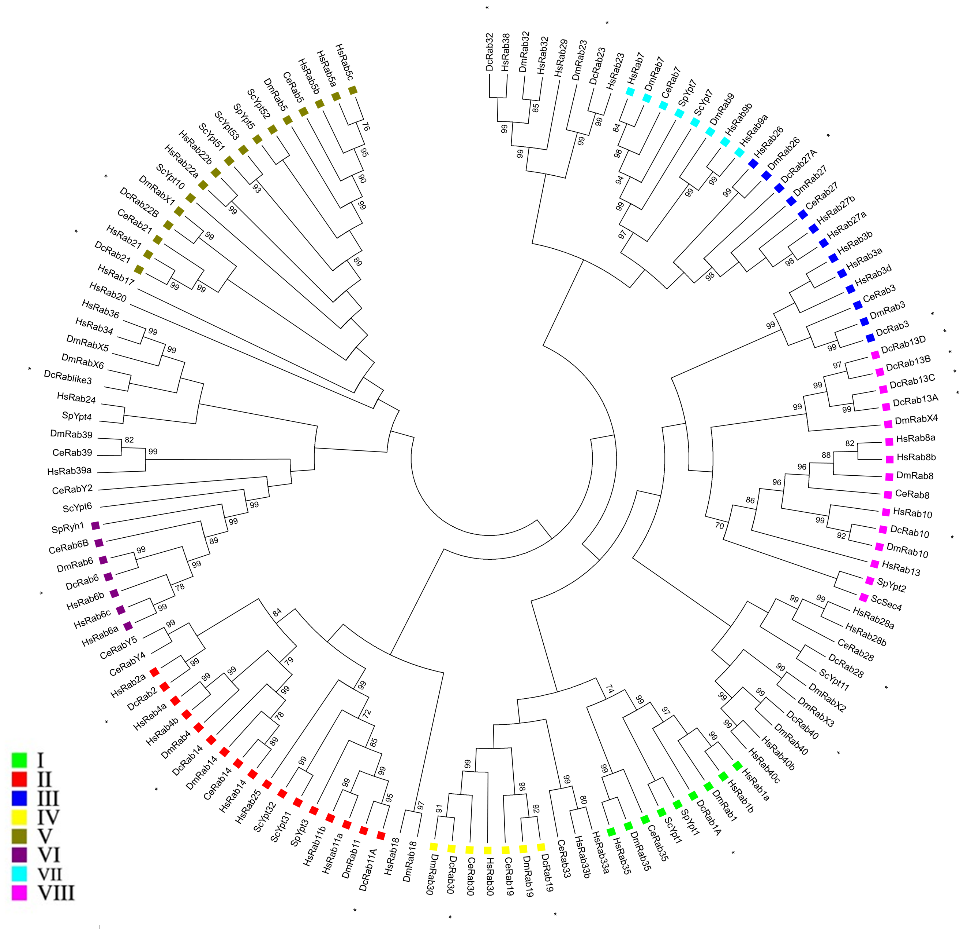


Figure 1: Phylogenetic analysis of 133 Rab amino acid sequences from *D. citri* (Dc), *C. elegans* (Ce), *D. melanogaster* (Dm), *H. sapiens* (Hs), *S. cerevisiae* (Sc), and *S. pombe* (Sp). Most Rabs grouped together in functional groups I-VIII. Groups are color-coded as shown in the legend and *D. citri* sequences are identified by an asterisk. Trees were constructed using the neighbor joining methods and bootstrap test of phylogeny. Bootstrap values >70 are placed at nodes based on 1000 replicates.


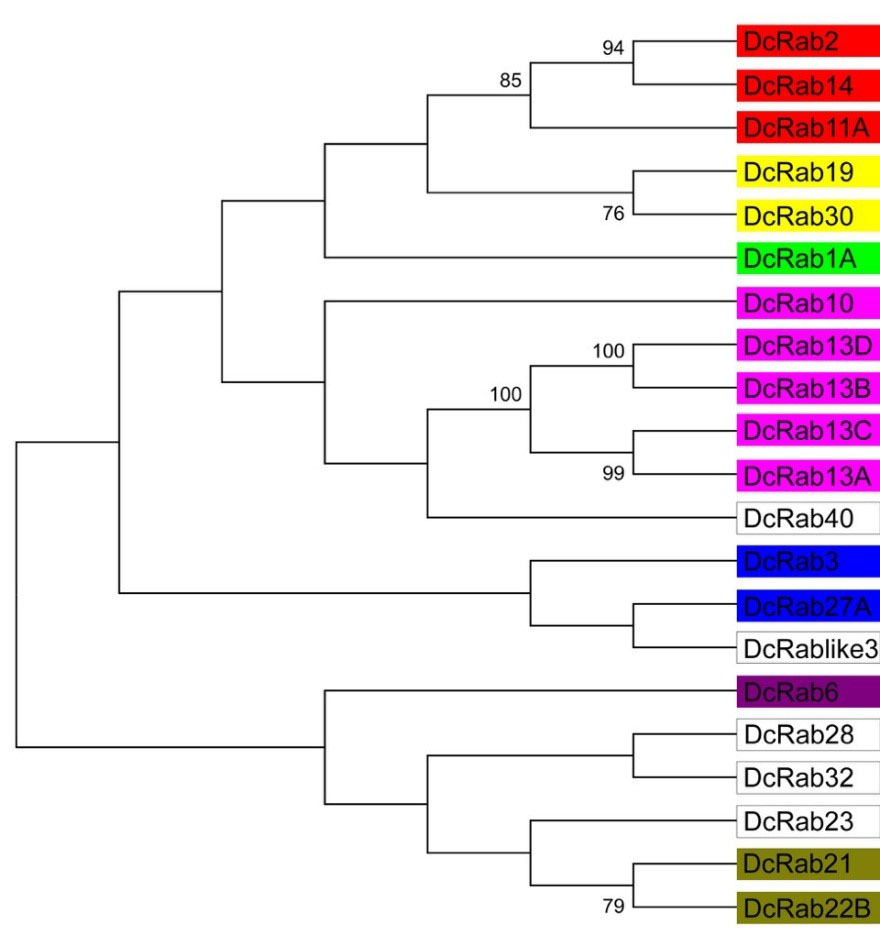


Figure 2: Phylogenetic analysis of 21 *D. citri* Rab amino acid sequences. Tree was constructed using the neighbor joining method and bootstrap test of phylogeny. Bootstrap values >70 (%) are placed at nodes based on 1000 replicates. Separation into functional groups still occurs. II, III, IV, and VIII are color coded per the same legend as in Fig.1. Clear boxes indicate ungrouped Rabs.

Table 3: This table shows the individual Rabs, their amino acid sequence length, and a general description generated during the time of manual annotation. It also shows the pairwise comparison between the *D. citri* Rabs and the sequences from *A. pisum and C. lectularius.* They have mostly high Query coverage and high bit scores.

| ***Diaphorina citri* RABs** | | | | | | | | | | | |
| --- | --- | --- | --- | --- | --- | --- | --- | --- | --- | --- | --- |
|  |  |  |  | Pea aphid | | | | Bedbug | | | |
| Gene | aa | Description | Domains | Description | Accession | query | Bit Score | Description | Accession | query | Bit Score |
| Rab1A | 204 | key regulatory component for the transport of vesicles from the ER to the Golgi apparatus | Rab1_Ypt1 | PREDICTED: ras-related protein ORAB-1 [Acyrthosiphon pisum] | XP_001951763.1 | 86% | 585 | PREDICTED: ras-related protein Rab-1A-like [Cimex lectularius] | XP_014260675.1 | 96% | 647 |
| Rab2 | 243 | Rab2 is localized on cis-Golgi membranes and interacts with Golgi matrix proteins. Rab2 is also implicated in the maturation of vesicular tubular clusters (VTCs), which are microtubule-associated intermediates in transport between the ER and Golgi apparatus. | Rab2 | PREDICTED: ras-related protein Rab-2A [Acyrthosiphon pisum] | XP_001951908.1 | 94% | 684 | PREDICTED: ras-related protein Rab-2 [Cimex lectularius] | XP_014245059.1 | 97% | 704 |
| Rab3 | 221 | Controls Ca2+-regulated exocytosis. Possibly involved in hormone and neurotransmitter secretion. | Rab3 | PREDICTED: ras-related protein Rab-3 [Acyrthosiphon pisum] | XP_001944695.1 | 100% | 1034 | PREDICTED: ras-related protein Rab-3 [Cimex lectularius] | XP_014257053.1 | 98% | 1028 |
| Rab like 3 | 217 | NA | RabL3 | PREDICTED: rab-like protein 3 [Acyrthosiphon pisum] | XP_016660492.1 | 91% | 522 | PREDICTED: rab-like protein 3 [Cimex lectularius] | XP_014242421.1 | 94% | 563 |
| Rab6 | 378 | Rab6 is involved in microtubule-dependent transport pathways through the Golgi and from endosomes to the Golgi. | Rab6 | rab6-like [Acyrthosiphon pisum] | NP_001155439.1 | 100% | 764 | PREDICTED: ras-related protein Rab6 isoform X2 [Cimex lectularius] | XP_014260763.1 | 100% | 779 |
| Rab10 | 270 | involved in post-Golgi transport to the plasma membrane | Rab8, Rab10, Rab13 like | PREDICTED: ras-related protein Rab-10 [Acyrthosiphon pisum] | XP_001951463.1 | 100% | 602 | PREDICTED: ras-related protein Rab-10 [Cimex lectularius] | XP_014248382.1 | 100% | 576 |
| Rab11A | 275 | regulate recycling pathways from endosomes to the plasma membrane and to the trans-Golgi network | Rab11A, Rab11B, Rab25 | PREDICTED: ras-related protein Rab-11A isoform X2 [Acyrthosiphon pisum] | XP_001950094.1 | 100% | 824 | PREDICTED: ras-related protein Rab-11A [Cimex lectularius] | XP_014245308.1 | 99% | 837 |
| Rab13A | 253 | involved in post-Golgi transport to the plasma membrane | Rab | PREDICTED: ras-related protein Rab-13-like isoform X1 [Acyrthosiphon pisum] | XP_001944801.1 | 87% | 677 | PREDICTED: ras-related protein Rab-13-like [Cimex lectularius] | XP_014246087.1 | 80% | 710 |
| Rab13B | 259 | involved in post-Golgi transport to the plasma membrane | Rab | PREDICTED: ras-related protein Rab-13-like isoform X1 [Acyrthosiphon pisum] | XP_001944801.1 | 85% | 674 | PREDICTED: ras-related protein Rab-13-like [Cimex lectularius] | XP_014246087.1 | 78% | 708 |
| Rab13C | 243 | involved in post-Golgi transport to the plasma membrane | Rab | PREDICTED: ras-related protein Rab-8A, partial [Acyrthosiphon pisum] | XP_003248501.2 | 82% | 659 | PREDICTED: ras-related protein Rab-13-like [Cimex lectularius] | XP_014246087.1 | 97% | 718 |
| Rab13D | 237 | involved in post-Golgi transport to the plasma membrane | Rab | PREDICTED: ras-related protein Rab-8A, partial [Acyrthosiphon pisum] | XP_003248501.2 | 83% | 661 | PREDICTED: ras-related protein Rab-13-like [Cimex lectularius] | XP_014246087.1 | 92% | 710 |
| Rab14 | 215 | Rab14 GTPases are localized to biosynthetic compartments, including the rough ER, the Golgi complex, and the trans-Golgi network, and to endosomal compartments, including early endosomal vacuoles and associated vesicles. Rab14 is believed to function in both the biosynthetic and recycling pathways between the Golgi and endosomal compartments. Rab14 has been suggested to help regulate GLUT4 translocation and play a role in the regulation of phagocytosis. | Rab14 | Rab-protein 14 isoform b [Acyrthosiphon pisum] | NP_001155935.1 | 90% | 832 | PREDICTED: ras-related protein Rab-14 [Cimex lectularius] | XP_014253287.1 | 99% | 907 |
| Rab19 | 234 | associated with Golgi stacks | Rab19 | PREDICTED: ras-related protein Rab-40C [Acyrthosiphon pisum] | XP_001945092.1 | 69% | 624 | PREDICTED: ras-related protein Rab-1A-like [Cimex lectularius] | XP_014248369.1 | 92% | 437 |
| Rab21 | 223 | Rab21 has been reported to localize in the ER. It has also been shown to colocalize with and affect the morpology of endosomes. When it is GTP-bound it has been shown to regulate phagocytosis. | Rab21 | ras-related protein Rab-21-like [Acyrthosiphon pisum] | NP_001191951.1 | 94% | 823 | PREDICTED: ras-related protein Rab-21 [Cimex lectularius] | XP_014245117.1 | 98% | 909 |
| Rab22B | 228 | Rab31, also called Rab22B, is involved in the trafficking of the trans-Golgi network. | Rab | PREDICTED: ras-related protein RABF2a-like isoform X1 [Acyrthosiphon pisum] | XP_001944237.1 | 75% | 450 | PREDICTED: ras-related protein Rab-31-like isoform X2 [Cimex lectularius] | XP_014246038.1 | 75% | 717 |
| Rab23 | 375 | Rab23 has been shown to regulate the sonic hedgehog pathway in mammals. | Rab23 like | GTP-binding protein YPTM1-like [Acyrthosiphon pisum] | NP_001156195.1 | 74% | 558 | PREDICTED: ras-related protein Rab-23 [Cimex lectularius] | XP_014255402.1 | 84% | 631 |
| Rab27A | 145 | Rab27A helps regulate exocytosis for different molecules and in different tissues depending on what effector molecules it has been paired up with. | Rab27A | PREDICTED: ras-related protein Rab-3 [Acyrthosiphon pisum] | XP_001944695.1 | 62% | 225 | PREDICTED: ras-related protein Rab-27A [Cimex lectularius] | XP_014244051.1 | 93% | 347 |
| Rab28 | 231 | Rab28 is a late embryogenesis-abundant (Lea) protein that is regulated by the plant hormone abcisic acid (ABA). | Rab | PREDICTED: ras-related protein Rab-28-like [Acyrthosiphon pisum] | XP_003245469.1 | 73% | 236 | PREDICTED: ras-related protein Rab-28-like isoform X2 [Cimex lectularius] | XP_014252593.1 | 73% | 269 |
| Rab30 | 203 | Rab30 appears to be associated with the Golgi stack and is expressed in a wide variety of tissue types. | Rab30 | PREDICTED: ras-related protein Rab-30 [Acyrthosiphon pisum] | XP_001945306.2 | 99% | 835 | PREDICTED: ras-related protein Rab-30-like [Cimex lectularius] | XP_014250576.1 | 98% | 914 |
| Rab32 | 318 | Rab32 functions as an A-kinase anchoring protein (AKAP). | Rab32 Rab28 | PREDICTED: ras-related protein Rab-38-like isoform X2 [Acyrthosiphon pisum] | XP_003241872.1 | 64% | 774 | PREDICTED: ras-related protein Rab-32 [Cimex lectularius] | XP_014246796.1 | 70% | 797 |
| Rab40 | 359 | Rab40 may function as an E3 ubiquitin ligase and mediate the degradation of proteins, and it may be involved in the transport of myelin components. | Rab40; SOCS Rab40 (supressor of cytokine signaling) | PREDICTED: ras-related protein Rab-40C [Acyrthosiphon pisum] | XP_001947621.1 | 69% | 1052 | PREDICTED: ras-related protein Rab-40C [Cimex lectularius] | XP_014255234.1 | 74% | 1091 |

Table 4: Rab gene family gene number in *Diaphorina citri, Drosophila melanogaster, Homo sapiens, Saccharomyces cerevisiae, Caenorhabditis elegans,* and *Trypanosoma brucei*. (Pereira-Leal e Seabra, 2001; Ackers *et al.*, 2005; Zhang *et al.*, 2007)

| **Rab gene family member count in *D. citri* and other species** | |
| --- | --- |
| *Diaphorina citri* | 21 |
| *Drosophila melanogaster* | 33 |
| *Homo sapiens* | 66 |
| *Saccharomyces cerevisiae* | 11 |
| *Caenorhabditis elegans* | 28 |
| *Trypanosoma brucei* | 16 |

**Results and Discussion**

With manual annotation twenty-one individual Rab models were completed within the Web Apollo editor of the *D. citri* genome. Two Rab gene-models were removed during quality control. A BLASTP search of the *D. citri* MCOT database with default settings found 60 hits above the 1e-10 e-value threshold, which includes duplicated results and sequences. When comparing the number of Rabs found in *D. citri* (21) and other organisms, like *D. melanogaster* (33), *H. sapiens* (66), *C. elegans* (28)*, S. cerevisiae* (11), and *T. burcei* (16) you see a wide range of Rab members accounted for (Table 4)*.* Since in *D. melanogaster* at least 10 more Rab genes were found it should be expected that more could be found *D. citri*. Comparison of the amino acid sequences of all Rabs in a multiple alignment revealed evolutionary conservation of the Rabs and their domains. Using BLASTP, it was shown that there was a significant match with orthologs of other insects (Table 2). Most of the *D. citri* Rabs were grouped with orthologs forming seven of eight functional groups (Figure 1) (Table 1). A pattern of inheritance helps support the identity of the sequence. The forming of the functional groups in the phylogenetic tree allows for the general function assignment to their individual group members (Pereira-Leal and Seabra 2001) (Ackers et al. 2005). Because an analysis of the Rab family has been performed we now have support of the idea that these sequences could be used for future analysis and possible therapies to help fight the spread of citrus greening. Understanding the evolution and purpose of the Rabs would help us determine where they would be best used in the process of saving citrus.

**References**

1. Ackers JP, Dhir V, and Field MC. 2005. A bioinformatic analysis of the RAB genes of *Trypanosoma brucei*. Mol Biochem Parasitol [Internet] [cited 25 March 2015]; 141:89-97. Available from: <http://www.sciencedirect.com/science/article/pii/S0166685105000605>
2. Brighouse A, Dacks JB, and Field MC. 2010. Rab protein evolution and the history of the eukaryotic endomembrane system. Cell Mol Life Sci [Internet] [cited 25 March 2015]; 67:3449-3465. Available from: <http://resolver.flvc.org/FLCC1200?sid=google&auinit=A&aulast=Brighouse&atitle=Rab+protein+evolution+and+the+history+of+the+eukaryotic+endomembrane+system&id=doi:10.1007/s00018-010-0436-1&title=Cellular+and+Molecular+Life+Sciences&volume=67&issue=20&date=2010&spage=3449&issn=1420-682X>
3. Chua CEL and Tang BL. 2015. Role of Rab GTPases and their interacting proteins in mediating metabolic signalling and regulation. Cell Mol Life Sci [Internet] [cited 25 March 2015]; 72:2289-2304. Available from: <http://resolver.flvc.org/FLCC1200?sid=google&auinit=CEL&aulast=Chua&atitle=Role+of+Rab+GTPases+and+their+interacting+proteins+in+mediating+metabolic+signalling+and+regulation&id=doi:10.1007/s00018-015-1862-x&title=Cellular+and+Molecular+Life+Sciences&volume=72&issue=12&date=2015&spage=2289&issn=1420-682X>
4. Diekmann Y, Seixas E, Gouw M, Tavares-Cadete F, Seabra MC, and Pereira-Leal JB. 2011. Thousands of Rab GTPases for the Cell Biologist. PLos Comput Biol [Internet] [cited 25 March 2015]; 7(10):1-15. Available from: <http://resolver.flvc.org/FLCC1200?sid=google&auinit=Y&aulast=Diekmann&atitle=Thousands+of+rab+GTPases+for+the+cell+biologist&id=doi:10.1371/journal.pcbi.1002217&title=PLoS+Computational+Biology&volume=7&issue=10&date=2011&spage=e1002217&issn=1553-734X>
5. Klopper TH, Kienle N, Fasshauer D, and Munro S. 2012. Untangling the evolution of Rab G proteins: implications of a comprehensive genomic analysis. BMC Biology [Internet] [cited 25 March 2015]; 10(71):1-17. Available from: <http://bmcbiol.biomedcentral.com/articles/10.1186/1741-7007-10-71>
6. Pereira-Leal JB and Seabra MC. Evolution of the Rab Family of Small GTP-binding Proteins. 2001. J Mol Biol [Internet] [cited 25 March 2015]; 313:889-901. Available from: <http://www.sciencedirect.com/science/article/pii/S0022283601950727>
7. Pereira-Leal JB and Seabra MC. The Mammalian Rab Family of the Small GTPases: Definition of Family and Subfamily Sequence Motifs Suggests a Mechanism for Functional Specificity in the Ras Superfamily. 2000. J Mol Biol [Internet] [cited 25 March 2015]; 301:1077-1087. Available from: <http://www.sciencedirect.com/science/article/pii/S0022283600940105>
8. Seabra MC, Mules EH, and Hume AN. Jan 2002. Rab GTPases, intracellular traffic and disease. Trends Mol Med [Internet] [cited 25 March 2015]; 8(1):23-30. Available from: <http://www.sciencedirect.com/science/article/pii/S1471491401022274>
9. Zhang J, Schulze KL, Hiesinger R, Suyama K, Wang S, Fish M, Acar M, Hoskins RA, Bellen HJ, ans Scott MP. 2007. Thirty-one flavors of Drosophila Rab Proteins. Genetics [Internet]. [cited 5 December, 2016]; 176:1307-1322. Available from: <http://www.genetics.org/content/176/2/1307>
10. ACKERS, J. P.; DHIR, V.; FIELD, M. C. A bioinformatic analysis of the RAB genes of Trypanosoma brucei. **Molecular and biochemical parasitology,** v. 141, n. 1, p. 89-97, 2005. ISSN 0166-6851.
11. PEREIRA-LEAL, J. B.; SEABRA, M. C. Evolution of the Rab family of small GTP-binding proteins. **Journal of molecular biology,** v. 313, n. 4, p. 889-901, 2001. ISSN 0022-2836.
12. ZHANG, J. et al. Thirty-one flavors of Drosophila rab proteins. **Genetics,** v. 176, n. 2, p. 1307-1322, 2007. ISSN 0016-6731.
