# Supplementary material for: Improved annotation of the insect vector of citrus greening disease: biocuration by a diverse genomics community
Source: Database (Oxford). 2017 Jun 30;2017:bax032. doi: 10.1093/database/bax032 (PMC5502364; doi:10.1093/database/bax032)
Supplement: Supplementary Data [file bax032_supp.zip › Annotation Guidelines for D citri.pdf]

BERKELEY LAB
LAWRENCE BERKELEY NATIONAL LABORATORY

U.S. DEPARTMENT OF ENERGY

Apollo Annotation Guidelines for i5k Projects

Diaphorina citri

Monica Munoz-Torres | @monimunozto

Berkeley Bioinformatics Open-Source Projects (BBOP)
Environmental Genomics & Systems Biology Division
Lawrence Berkeley National Laboratory

i5k Pilot Project Species | 16 February, 2016

http://GenomeArchitect.org

General process of curation

1. Select or find a **region of interest**, e.g. scaffold.
2. Select appropriate **evidence** tracks to review the gene model.
3. Determine whether a feature in an existing evidence track will provide a reasonable **gene model to start** working.
4. If necessary, **adjust** the gene model.
5. Check your edited gene model for **integrity and accuracy** by comparing it with available homologs.
6. **Comment.**

Collaborative curation at i5k

1. The Apollo User Guide is available at http://genomearchitect.org/web_apollo_user_guide
2. Specific guidelines for i5k Pilot Species Projects are available at the i5k Worskpace@NAL https://goo.gl/9R0J4E
3. A computationally predicted consensus gene set has been generated using multiple lines of evidence. E.g. *HVIT_v0.5.3-Models* or *MAKER gene predictions*.
4. In some cases algorithms and metrics used to generate consensus sets may actually reduce the accuracy of the gene's representation. Use your judgment, try choosing a different model to begin the annotation.
5. If an annotation needs to be removed from the consensus set, drag it to the '***User-created Annotations***' area and label as '***Delete***' using the radio button in the the *Information Editor*.
6. **Annotate isoforms when possible:** drag the original model and also annotate all alternatively spliced forms in '***User-created Annotations***' area. '**Replace Models**' rules apply.
7. i5k Projects will integrate consensus computational predictions with manual annotations to produce an updated Official Gene Set (OGS):

   Warning!

   - If an annotation is on either the consensus or manual annotations tracks and it shouldn't, it will make the OGS!
   - If annotation is not on either track, it won't make the OGS!
8. Collaborate to reach agreement on overlapping interests.

What does it take to initiate an annotation?

After locating your gene or region of interest, add as many gene prediction and evidence tracks as you consider necessary to inform your annotation by clicking on and off from the list of '*Available Tracks*' on the left. Scroll through the different tracks of gene predictions and choose one that you consider most closely reflects the actual structure of the gene. You may base your decision on prior knowledge of the reliability of each gene prediction track (e.g., select an evidence-based gene model instead of an *ab initio* gene prediction). Alternatively, you may compare the gene prediction tracks to a BLAST alignment or other aligned data (e.g.: alignments of protein homologs, cDNAs and, RNAseq reads). Drag the highlighted model or all pieces of evidence into the '*User-created Annotations*' area.

You may then download the amino acid sequence (*Right Click Menu / Get Sequence*) to query a public database and help you determine if the selected gene model is, biologically speaking, an accurate approximation to the gene. For example, you may perform a protein sequence search of UniProt or NCBI's non-redundant peptide database, NR. If you have knowledge of protein domains in your gene of interest, you may perform a protein domain search of the InterPro databases or NCBI's Conserved Domain Database to verify that your selected gene model contains the expected domains.

Once a gene model is selected as the best starting point for annotation, the annotator must decide whether it needs further modification. Protein or domain database searches may have already informed this decision. Scroll down the evidence tracks to see if splice sites in transcript alignments agree with the selected gene model, or if evidence suggests addition or modification of an exon is necessary. Transcript alignments (e.g. cDNA/EST/RNASeq tracks) that are significantly longer than the gene model may indicate the presence of additional coding sequence or untranslated regions (UTRs). Keep in mind that transcript alignments may be shorter than the gene model due to the fragmented nature of current transcript sequencing technologies. Similarly, protein alignments may not reflect the entire length of the coding region because divergent regions may not align well, resulting in a short protein alignment or one with gaps. Protein and transcript alignments in regions with tandem, closely related genes might also be problematic, with partial alignments to one gene, then skipping over to align the rest to a second gene. More at http://genomearchitect.org/web_apollo_user_guide

Checklist

for manual annotation of a gene model

1. Check, correct or add ‘***Start***’ and ‘***Stop***’ sites.
2. Check **splice sites**: most splice sites display these residues **5**’-**…exon]GT…**intron**…AG[exon…-3**’
3. Check if you can annotate **UTRs**, for example using RNA-Seq data:
    - align it against relevant genes/gene family
    - blastp against NCBI’s RefSeq or NCBI nr
4. Check and note **gaps** in the assembly.
5. Add supporting details to the **Annotation Information Editor.** (See next page).

Annotation Information Editor

- Add supporting details to each box in the Information Editor.

  - Begin adding information by clicking the '*Add*' button and typing.
  - A click outside the box saves this information automatically.
  - Another click on the box allows you to edit the information.
  - The '*Delete*' button removes the entry.

1. Add comments documenting how you modified the automated gene predictions. E.g.:
   - **merging** 2 gene predictions in the same scaffold
   - gene prediction spans **across 2 or more scaffolds**
   - **splitting** a gene prediction
   - annotating **frameshifts**, annotating selenocysteines, correcting single-base and other assembly errors, etc.
2. Also add comments with important project information.
3. Add gene symbol(s), common name(s), synonyms on the top boxes.
4. Use '**DBXRef**' to add IDs from public databases (e.g. NCBI's GenBank) for the gene of interest in this species and also for orthologs from other species, top BLAST hits, etc.
5. Add any appropriate functional assignments obtained via BLAST, RNA-Seq data, literature searches, etc., using '**Gene Ontology IDs**'.
6. Follow the rules for '**Replaced Models**', when applicable.

Annotation Information Editor

Example

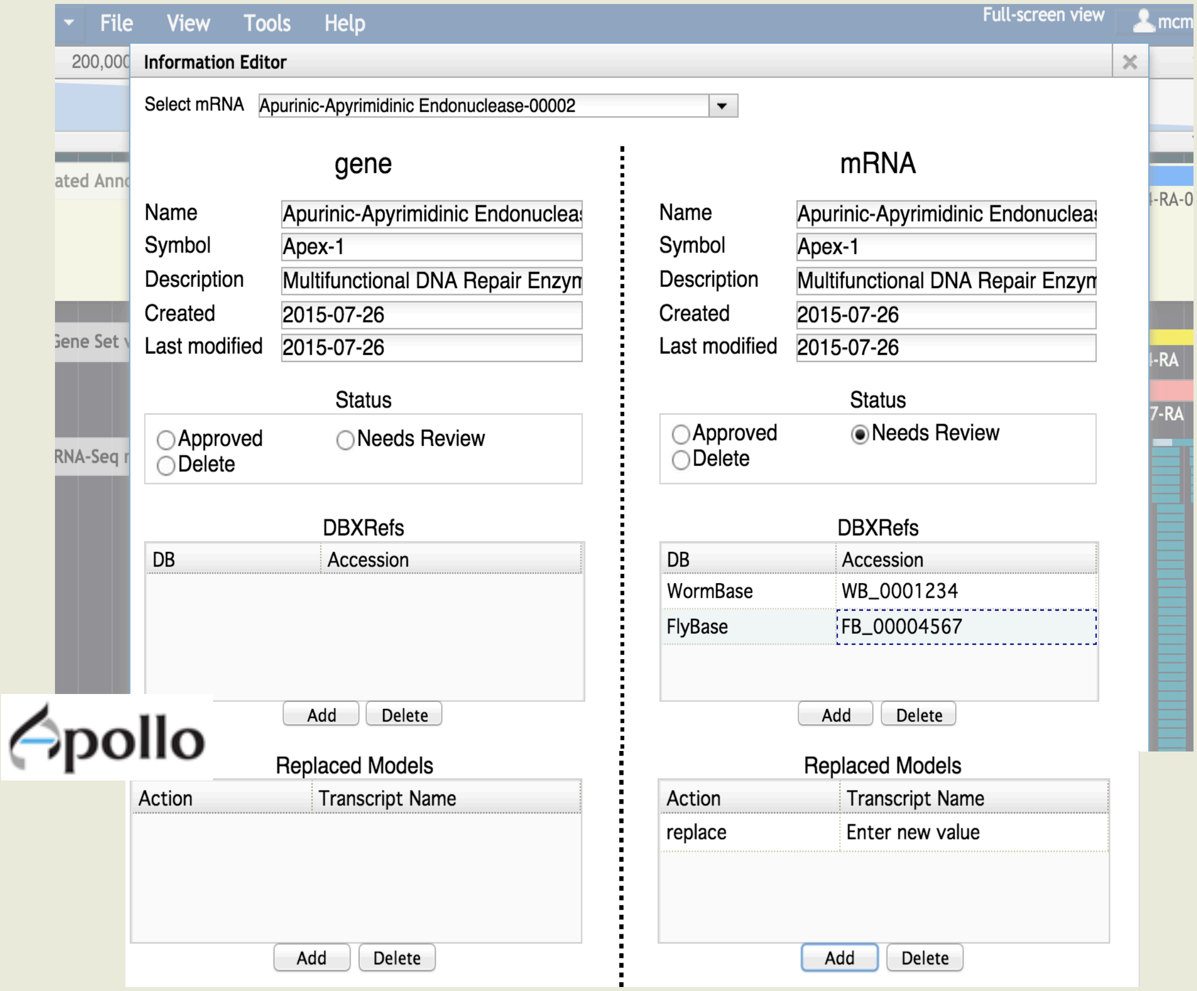

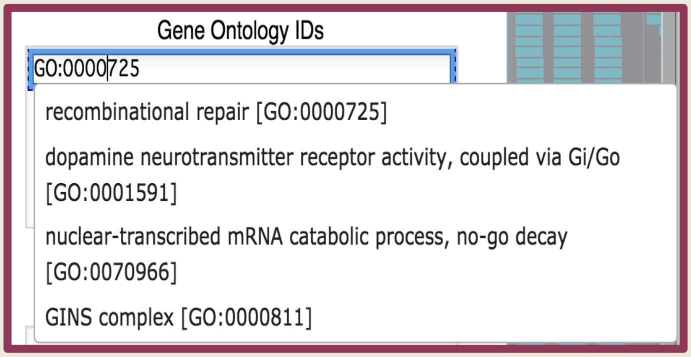

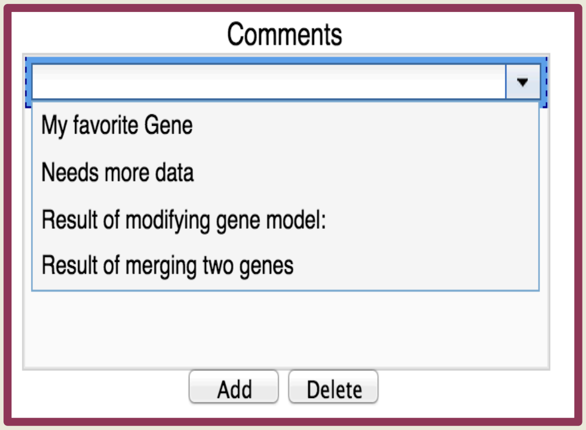

Remember:

- Add **PubMed IDs** from publications used to support your annotations.
- Include **Gene Ontology** terms as appropriate from any of the three ontologies.
- Write comments stating how you have validated each model.

The ‘Replace Models’ rules

Explanations and examples for how to enter information in the ‘**Replace Models**’ field are available at i5k Workspace@NAL. Visit http://tinyurl.com/apollo-i5k-replace

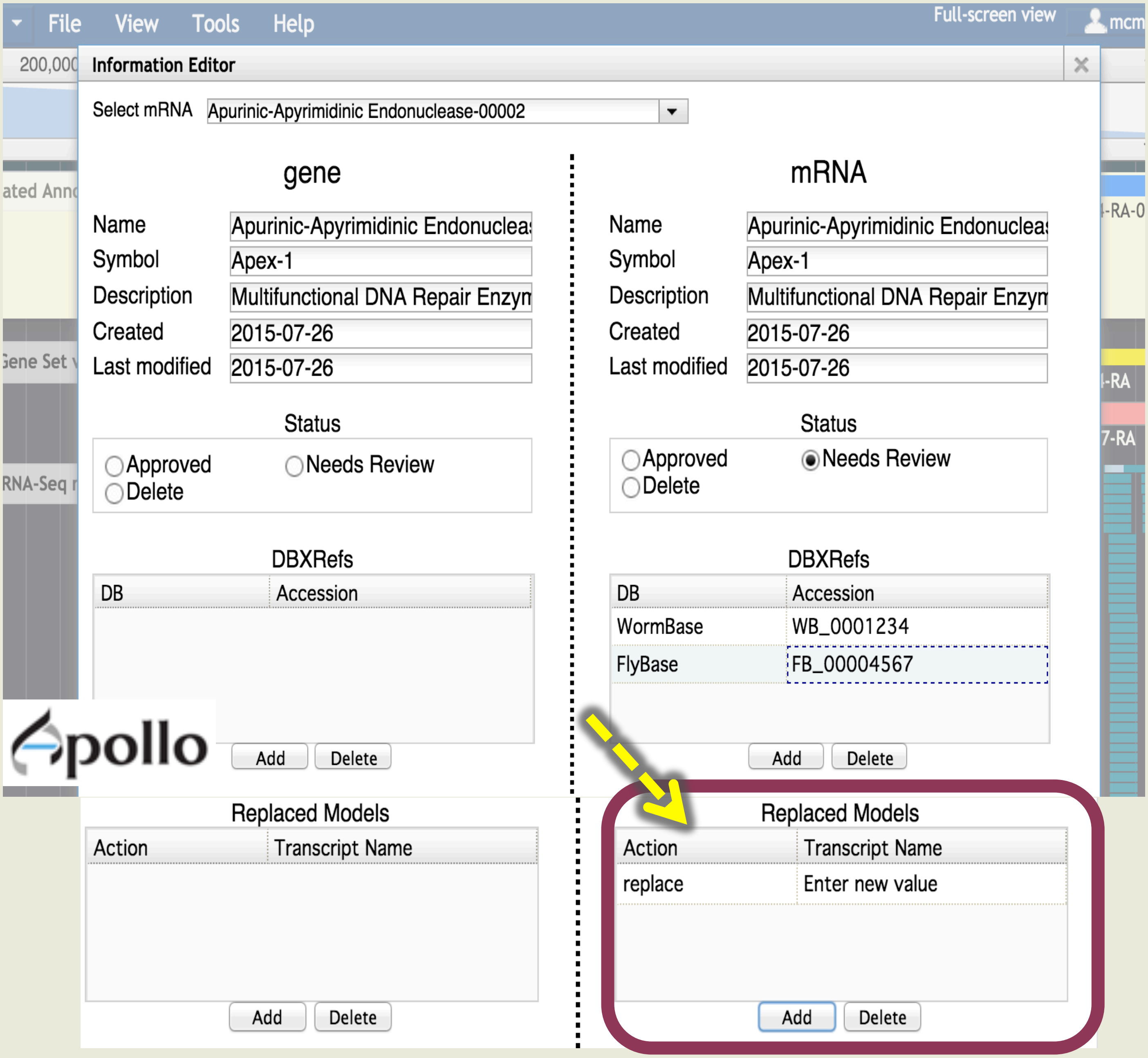

The *Diaphorina citri* research community

- **Apollo instance for *Diaphorina citri*** at i5k Workspace@NAL: https://apollo.nal.usda.gov/diacit/sequences

- All annotations from Apollo should also be added to the **Citrus Psyllid Gene Annotation Workbook**, our shared spreadsheet available on Google Drive at https://goo.gl/dd7c2U

- *Diaphorina citri* **Project Page** at i5k Workspace@NAL: https://i5k.nal.usda.gov/Diaphorina_citri

- Details about the the USDA NIFA citrus greening project are available at http://goo.gl/H41CxI

- Our **Documentation Management System** – Basecamp is available at https://goo.gl/I0agUK
Please request access from Ms. Kascha Bohnenblust at kascha@ksu.edu
