# Supplementary material for: Improved annotation of the insect vector of citrus greening disease: biocuration by a diverse genomics community
Source: Database (Oxford). 2017 Jun 30;2017:bax032. doi: 10.1093/database/bax032 (PMC5502364; doi:10.1093/database/bax032)
Supplement: Supplementary Data [file bax032_supp.zip › Annotation Tutorial.pdf]

Citrus Greening Solutions
A USDA NIFA Project

Annotation Tutorial: *Diaphorina citri* genome

Indian River State College, FL

Websites you will need

Basecamp: https://basecamp.com/

Apollo: https://apollo.nal.usda.gov/diacit/sequences

i5k Blast or hmmer: https://i5k.nal.usda.gov/webapp/blast/

https://i5k.nal.usda.gov/webapp/hmmer/

NCBI Blast: https://blast.ncbi.nlm.nih.gov/Blast.cgi

MCOT database: https://citrusgreening.org/tools/blast?db_id=27

Chose Gene or Gene family from Basecamp

Basecamp 2 | Projects | Calendar | Everything | Progress | Everyone | Me

New features Sign out

Jump to a project, person, label, or search...

Psyllid Genome Annotation

See all text documents

Example:

SODs annotation

Superoxide Dismutatses

Please select a gene family for annotation
SODs Mn-Fe (6 Orthologs)- Chris Cordola
SODs Cu-Zn (14 Orthologs) -Krystal Villalobos
Now with BedBug *Cimex lectularius* genes

You can find protein sequence fasta files at
ftp://ftp.citrusgreening.org/annotation/curated_orthologs/SODs/

Annotated *Dm-Ag-Aa-Cp* Trees
http://cegg.unige.ch/files/insecta/immunodb/1/FAMILY_DATA/SOD/SODs_gapless N...

Add your annotated psyllid proteins and location from WebApollo to this Google doc in fasta format (please follow the Dcitr naming convention)
https://docs.google.com/document/d/1EPmQsH9OT-o30pttDSALdBOEaKFhYLZ9GlqFgA8...

Source:

- Expert annotations for D. melanogaster, A. gambiae, A. aegypti and C. quinquefasciatus http://cegg.unige.ch/Insecta/immunodb

Want to make changes?

Edit this document

Prashant Hosmani was the last person to edit this document on Oct 8, 2015 (See what changed).

Version history

Prashant H. Oct 8 at 12:47pm
Prashant H. Sep 18 at 3:46pm
Prashant H. Sep 18 at 3:44pm
Prashant H. Sep 18 at 3:44pm
Christopher C. Sep 18 at 11:56am
Krystal V. Sep 17 at 11:22pm
Prashant H. Sep 16 at 12:16pm
Prashant H. Sep 16 at 12:14pm

Delete...

Name	Size	Date Modified
[parent directory]		
SOD_Cu-Zn.faa	4.3 kB	10/8/15, 12:00:00 AM
SOD_Mn-Fe.faa	2.1 kB	10/8/15, 12:00:00 AM
SODs_readme.txt	1.1 kB	9/16/15, 12:00:00 AM

This page will take you to orthologous sequences & Gene family description

Orthologs

```
>Flyb:SOD2 Flyb:N1308 CG11793-PA Sod Cu-Zn Dm_Sod 'SOD-Cu-Zn'
MVVKAVCVINGDAKGTVFFEQESSGTPVKVSGEVCGLAKGLHGFHVHEFGDNTNGCMSSG
PHFNPYGKEHGAPVDENRHLGDLGNIEATGDCPTKVNITDSKITLFGADSIIGRTVVVHA
DADDLGQGGHELSKSTGNAGARIGCGVIGIAKV

>Flyb:SOD3 Flyb:N12721 CG9027-PA Cu-Zn Dm_no_name 'SOD-Cu-Zn'
MMQYLVVSLALCATICSAAQTRNMPIQAIAYLIGPVQSDNTQVKGNVTFTQNDCGQNVHV
RVQLEGLKEGKHGFHIHEKGDLTNGCISMGAHYNPDKVDHGGPDHEVRHVGDLGNLEANS
TGIIDVTYTDQVITLTGKLGIIGRGVVVHELEDDLGLGNHTDSKKTGNAGGRIACGVIGI
K

>Flyb:SOD4 Flyb:N10352 CG5948-PA Cu-Zn Dm_no_name 'SOD-Cu-Zn'
MLIKLILALVIGYGGIFAPGSAQNLLDRLRPVNMTRFDDGIKVVSGTKVKRYERLTVPLG
GPVGIPGAAGLLGPLLPPQPSYLGYTYQLVPQWQAGAKLMGDGEGAGVAGMISFVQLPYN
SDIRVTINVTGLPPGKHALHIHTFGDLSDGCKSTGGQFPNNFLGNVDTKDDGSISAVFQS
IYLQLFGINGIVGRSIVIHSKAIDLNTALNAEVFSSSLQAMPNPLAYQNEENSLGPAIAC
GVISIMSTAASSSGMATAAPAPPAMENPEI

>Agam:SOD2 Agam:N13914 ENSANGG00000013675 ENSANGP00000016164 SOD3a Ag_CuSOD3-A 'SOD-Cu-Zn'
MPLKAVCVLNGEVKGTIFFEQSGESDPVKVTGSVTGLKPGDHGFHIHEFGDNTNGCMSTG
AHFNPYAKTHGGPDAEERHAGDMGNIVADENGEAKVDLTATQIALSGALNVVGRSLVVHA
DPDDLGVGGHELSKTTGNAGARLACGVIGLCKI

>Agam:SOD3 Agam:N13914 ENSANGG00000013675 ENSANGP00000032094 SOD3b Ag_CuSOD3-B 'SOD-Cu-Zn'
MPLKAVCVLNGEVKGTIFFEQSGSSVAVTGALEGLRPGKHGCHIHEFGDFSRGCLSTGPH
YNPDGNDHGAPDDANCHVGDLGNIVAYSTGLAKIQIANKKLTLVGDRSIIGRTLSISEYE
DDLGRGKHDYSKTTGNSGNCIACAIIGVAREEYFAERLHLTTDE

>Agam:SOD4 Agam:N22525 ENSANGP00000015824 gene:ENSANGG00000013335 transcript:ENSANGT00000015824 S
MKVLIALSTVLCVVLAKDQPRKAIVYLQGTSGVSGNVTISQPSCTEPVFIDINVVGLTPG
KHGFHIHEKGDLTDGCASTGGHYNPDKVSHGAPNDQVRHVGDLGNIAADENGIAKTSYSD
TVVSLYGARSVIGRAIVIHAEVDDLGKTNHPDSLKTGNAGGRVACGVIGILEPFDEPDSE
CSSGQQGLLPAAVTVVFSLLLTRSWVL

>Agam:SOD5 Agam:N22962 ENSANGP00000019828 gene:ENSANGG00000017339 transcript:ENSANGT00000019828 A
MRRCPAVVGTLCLVPEPSWKVGATLVADNPEQQIMGTISFRQWAPGHVQTAINVTGLPVG
KHAVHVHAFGDMREGCKSTGPHFRSSIIGNIEVKEDGNAMIDFHSPYINLFGFAGIVGRS
IVIHEKPSEVYRFPDLSINNPISFQGEEDTVGARIACGLITILDNVAP

>Aaeg:SOD3 Aaeg:N31871(supercontig_1.5:1342653-1363914)=Aaeg:N34696(supercontig_1.584:422035-4224
MPAKAVCVLSGDVKGTIFFQQNGDSDPVKVTGEVTGLKPGNHGFHIHEFGDNTNGCTSAG
PHFNPHGKEHGGPDAAERHAGDLGNVVADGSGVAKVDISDSQISLSGPLSILGRTVVVHA
DPDDLGLGGHELSKSTGNAGARLACGVIGICKA
```

Test **ALL** orthologous sequences one by one in Apollo (Next slide)

“Blat” sequences in Apollo

Menu bar: Click Tools > Search Sequence

- Enter orthologous sequence (not in FASTA)
- Indicate nucleotide or protein
- Click Search

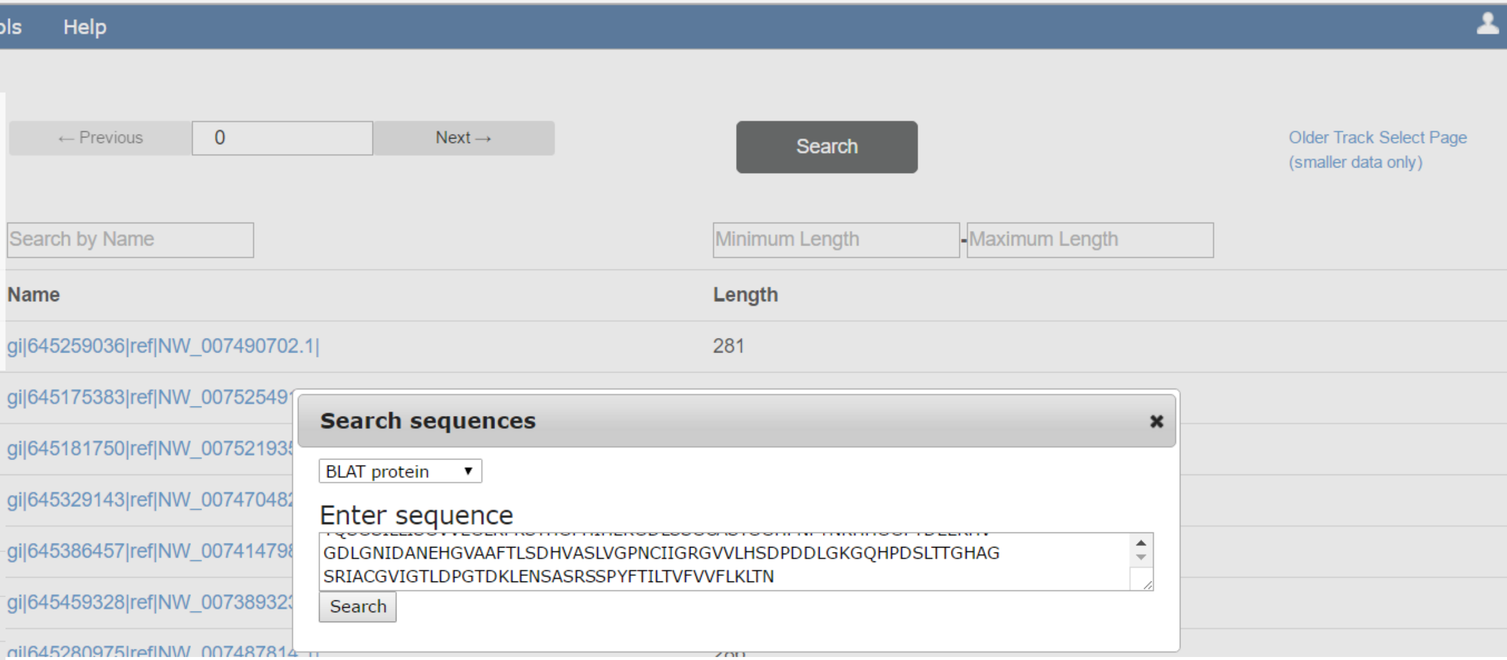

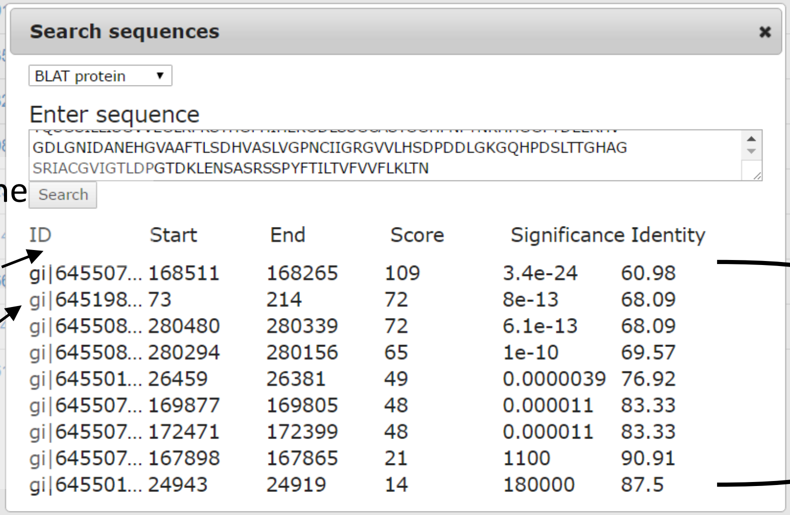

ID	Start	End	Score	Significance	Identity
gi|645507...	168511	168265	109	3.4e-24	60.98
gi|645198...	73	214	72	8e-13	68.09
gi|645508...	280480	280339	72	6.1e-13	68.09
gi|645508...	280294	280156	65	1e-10	69.57
gi|645501...	26459	26381	49	0.0000039	76.92
gi|645507...	169877	169805	48	0.000011	83.33
gi|645507...	172471	172399	48	0.000011	83.33
gi|645507...	167898	167865	21	1100	90.91
gi|645501...	24943	24919	14	180000	87.5

This is one scaffold

This is another scaffold, etc.

Results in different scaffolds

Change to BLAT protein if using a protein sequence (most often).
Be sure to check “search all genome sequences” if you want anything not already on your page.
Insert the letter sequence only. Do not use the > or any spaces.

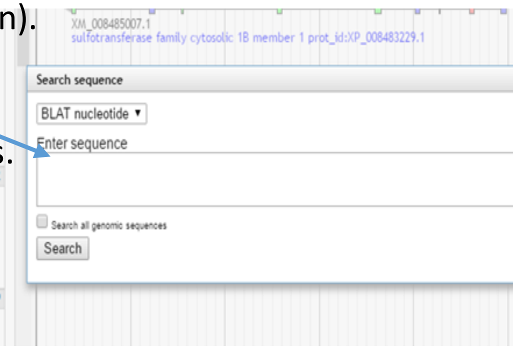

- Look at all scaffolds
- Significance that is very low and Score that is high is ideal
- *Remember* Test all orthologs given from basecamp

Apollo Search Results

Score: A number that the BLAT results "scores" the hit. The higher is often better, but not always. There is a balance with score and Significance.

Significance: The "E-value" given by the BLAT results. The smaller the number is often better, but not always. There is a balance with Score and Significance.

ID: The scaffold location

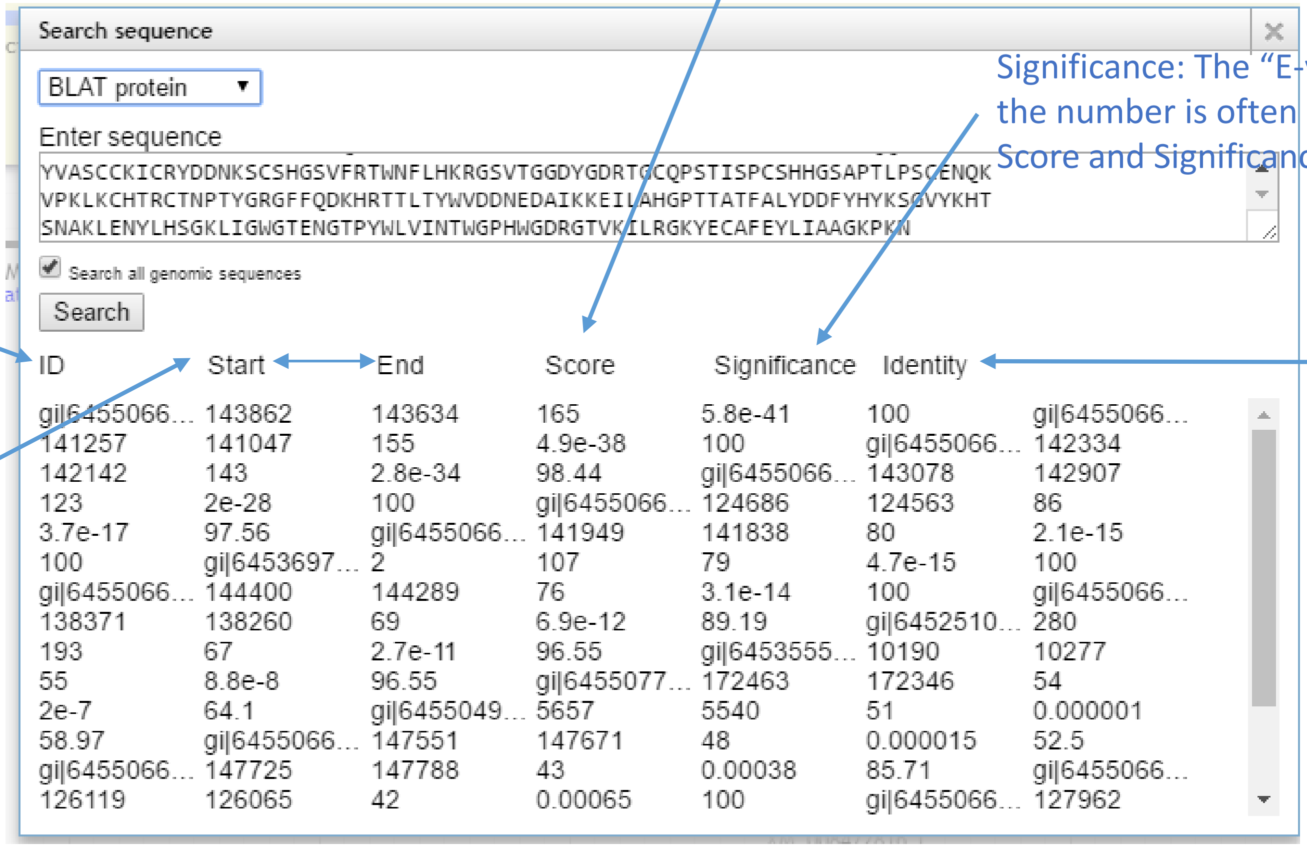

Identity: How well the sequence entered matches the location of the scaffold.

Start or End : The number where the sequence starts or ends

This example shows the columns out of place, this may happen, The ID section starts with gi.

Choose a scaffold. By clicking on it. You can then X out of the dialog box.

If Apollo says “No matches found”

Use i5k Blast

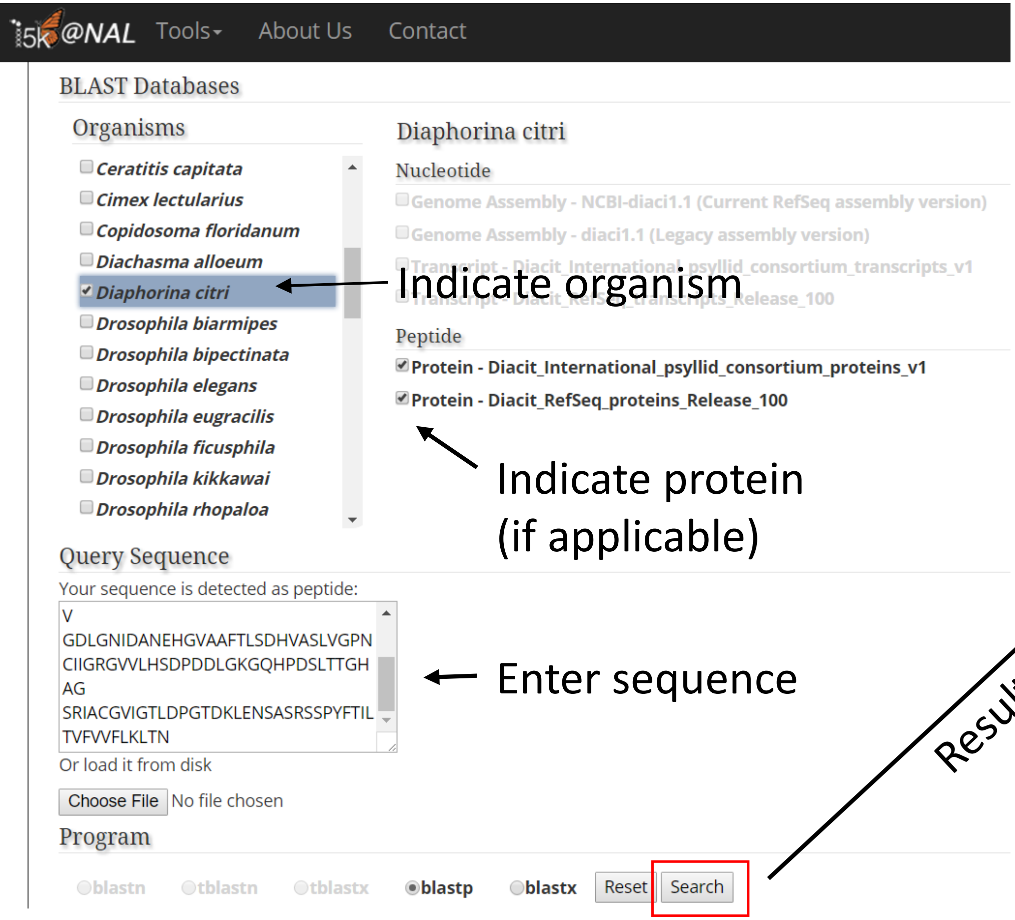

Results

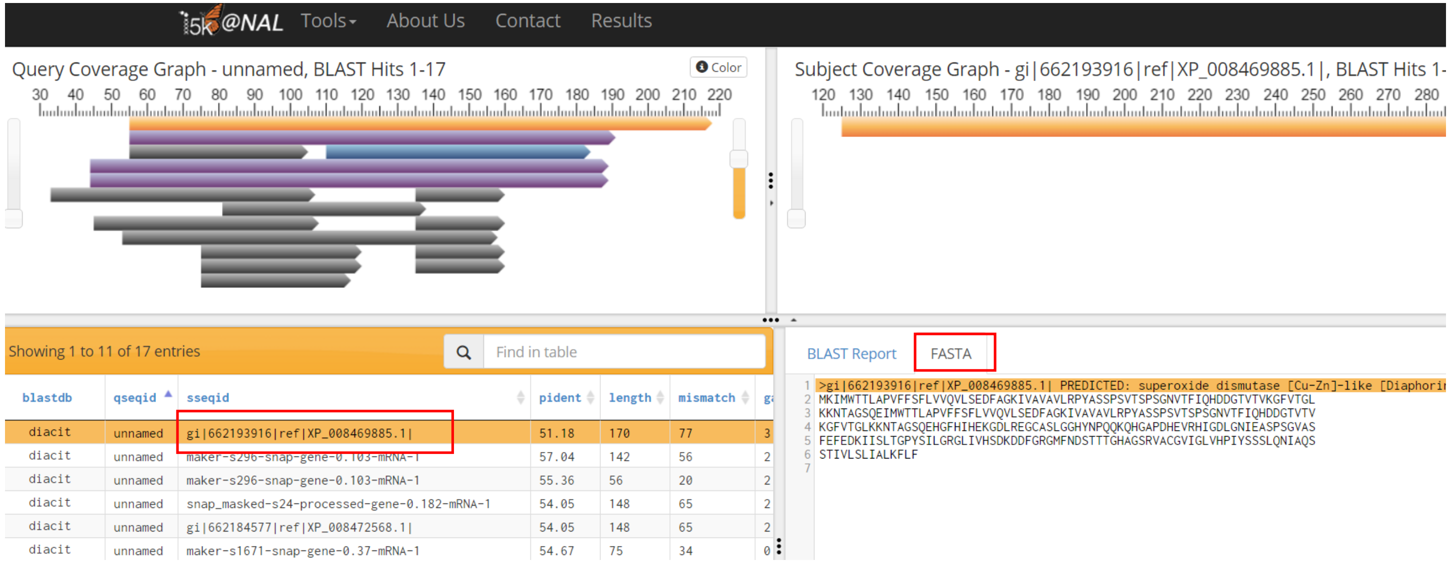

- Chose best result(s) (Low e-value, best coverage, etc.)
- Copy sequence and Blat in Apollo.

Apollo Blat vs i5k Blast

- Apollo Blatt is more “sensitive” than i5k Blast.
- i5k Blast will broaden the search.

If Apollo says “No matches found”

Search your sequence on *D. citri* MCOT database

https://citrusgreening.org/tools/blast?db_id=27 **BLAST**

Input parameters

**Categories**	Psyllid Databases
**Database**	Diaphorina citri MCOT proteins — db details
**Program**	blastp (protein to protein db)
**Query**	autodetect — Show example

Indicate MCOT protein DB

Indicate blastp, blastn, etc.

MTSESYKLFVESHPRFTTFSNYSLLKSYERPRCHFVFTLAQEHRAVAKLVGPNVSGNITF
TQSGSILLISGVVEGLKPKSTHGFHIHEKGDLSSGCASTGGHFNPYNKHHGGPTDEERHV
GDLGNIDANEHGVAAFTLSDHVASLVGPNCIIGRGVVLHSDPDDLGKGQHPDSLTTGHAG
SRIACGVIGTLDPGTDKLENSASRSSPYFTILTVFVVFLKLTN

Your sequence

BLAST

Results

Results

Untitled_sequence vs Diaphorina citri MCOT proteins

SubjectId	id%	Aln	evalue	Score	Description
MCOT10235.0.MM	57.04	81/142	1e-48	163	| Superoxide dismutase [Cu-Zn] | Similar to C4WTR6 | *-*- | PANTHER PTHR10003 | Pfam PF00080 Length = 274
MCOT13840.0.CO	55.10	81/147	2e-46	157	| Superoxide dismutase [Cu-Zn] | Similar to A0A023FAY3 | ***- | PANTHER PTHR10003 | Pfam PF00080 Length = 238
MCOT16518.0.CC	54.05	80/148	5e-44	148	| Superoxide dismutase [Cu-Zn] | Similar to R4V538 | ***- | PANTHER PTHR10003 | Pfam PF00080 Length = 155

Click for sequence, and blat on Apollo

If Apollo says "No matches found"

Search your sequence in i5k blast hmmer (hidden Markov model) created from a multiple sequence alignment of the ortholog proteins

https://i5k.nal.usda.gov/webapp/hmmer/

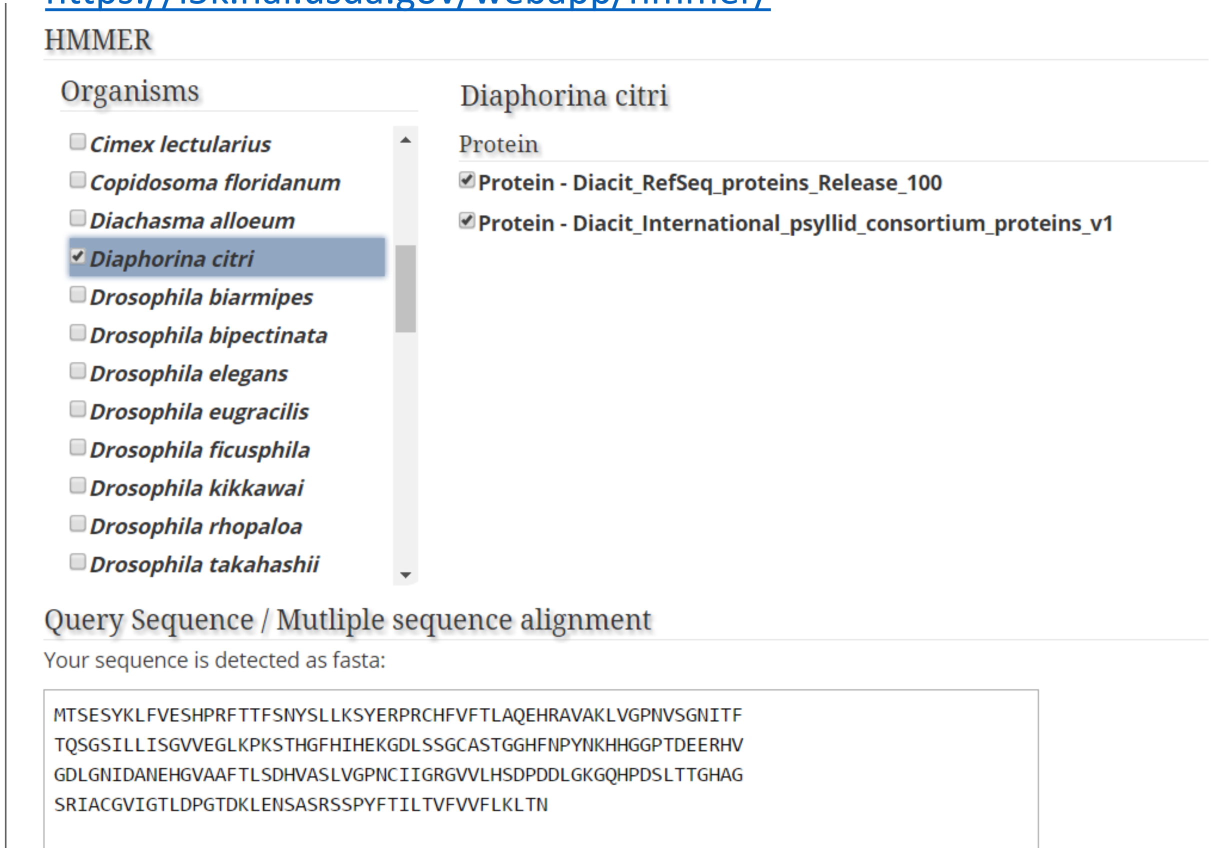

Tutorial

https://i5k.nal.usda.gov/webapp/hmmer/manual/

On Apollo, once you have selected a scaffold.

Be sure that the items you want to view are chosen on the left side of the screen. For the psyllid, we start to base our models off of NCBI predictions. Be sure the following gene sets are chosen.

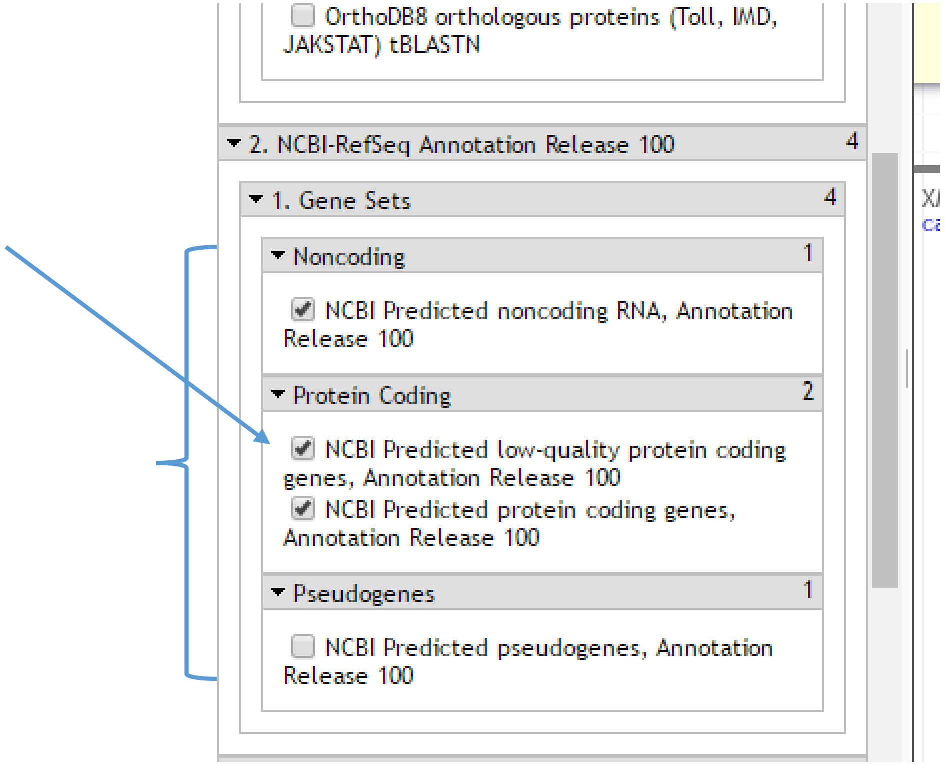

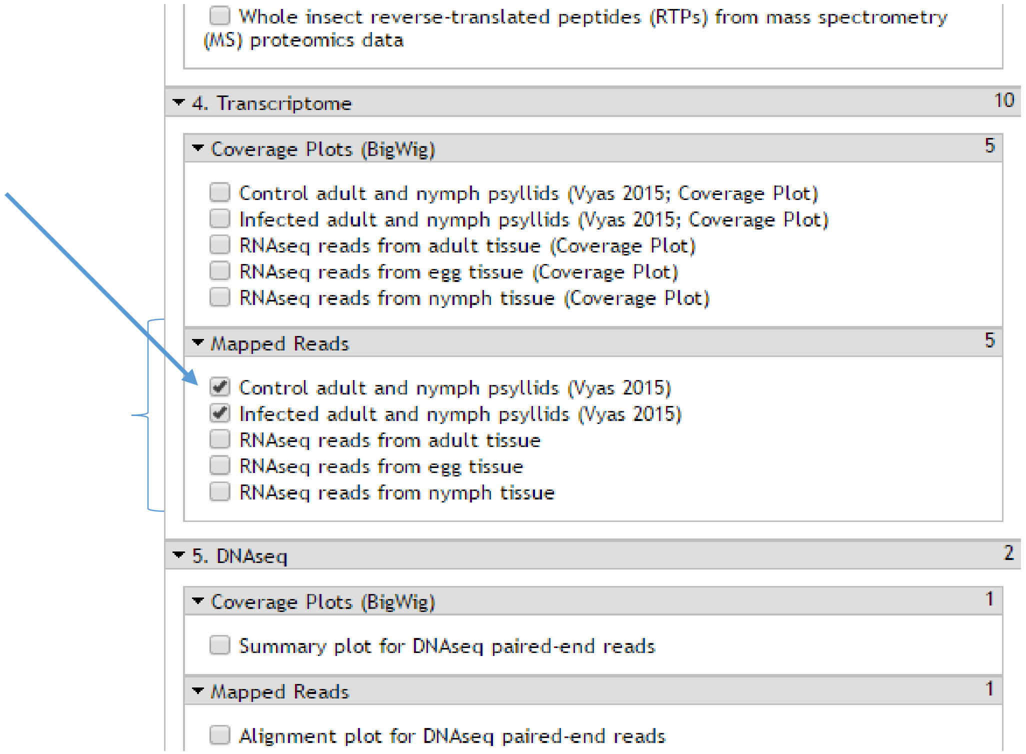

We will base our models off of RNAseq data, so be sure to choose some tracks under **Mapped Reads** as well.

Note: Be sure to check all RNAseq data, but it may overload the system if they are all turned on at once.

Predicted Gene Models

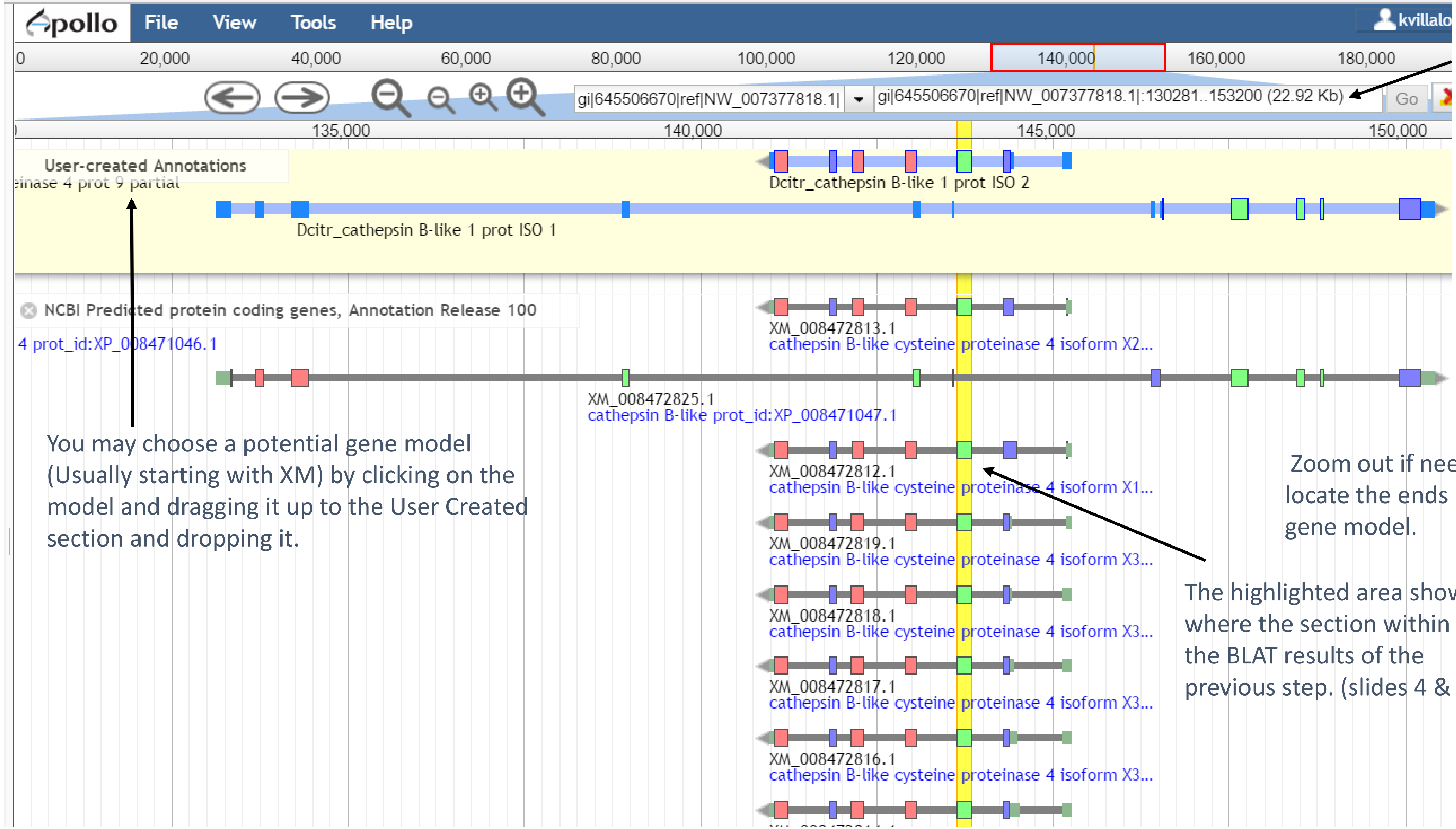

Useful tip: You can use this as an "URL" in Apollo. As you need to look at all scaffolds (BLAT results), use this URL to organize all your potential gene models and to get back to them.

You may choose a potential gene model (Usually starting with XM) by clicking on the model and dragging it up to the User Created section and dropping it.

Zoom out if need be and locate the ends of a potential gene model.

The highlighted area shows where the section within the BLAT results of the previous step. (slides 4 & 5)

Check Gene Models

Right click on the model in the User Created section and choose "Get sequence"

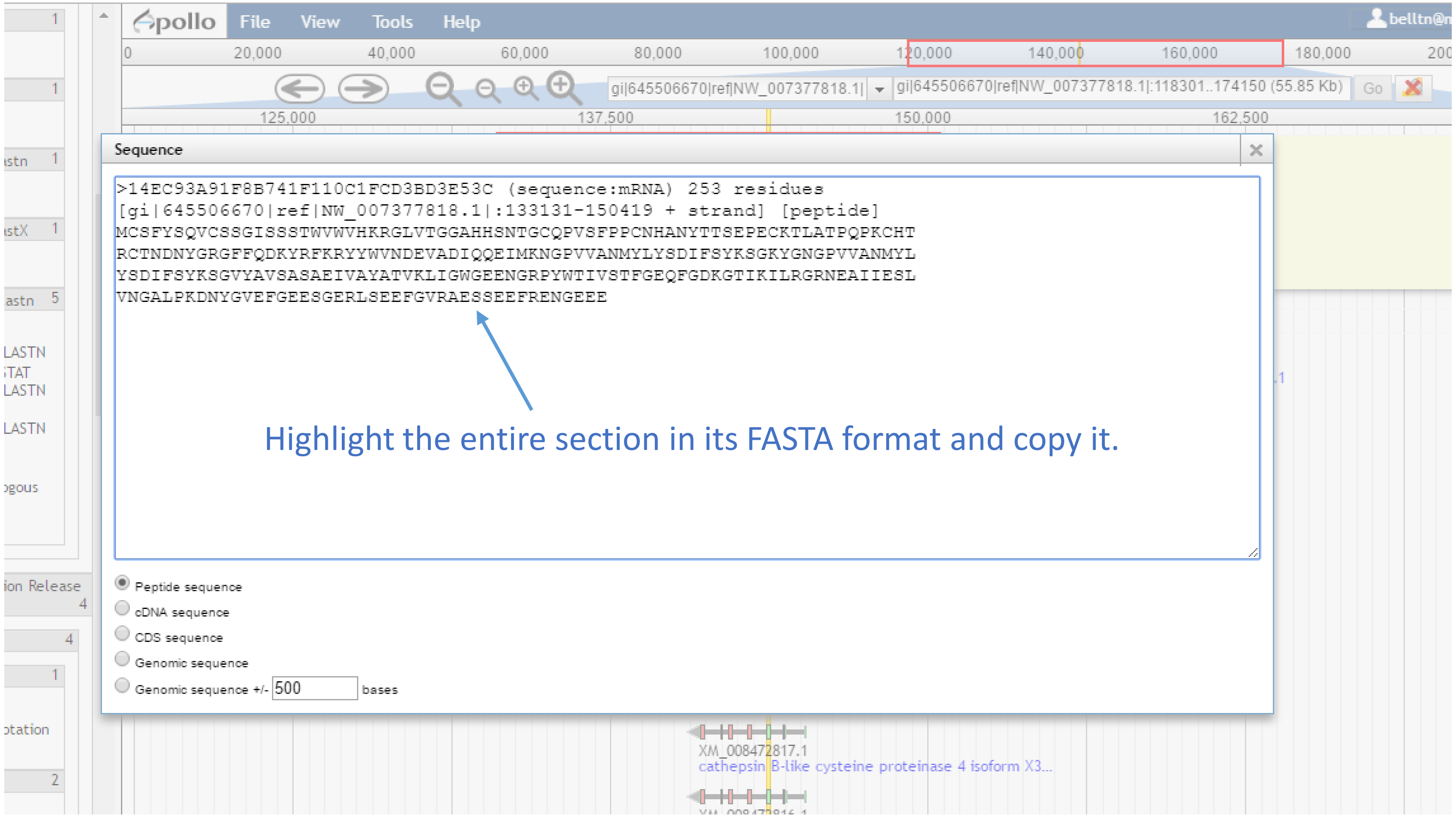

Protein BLAST

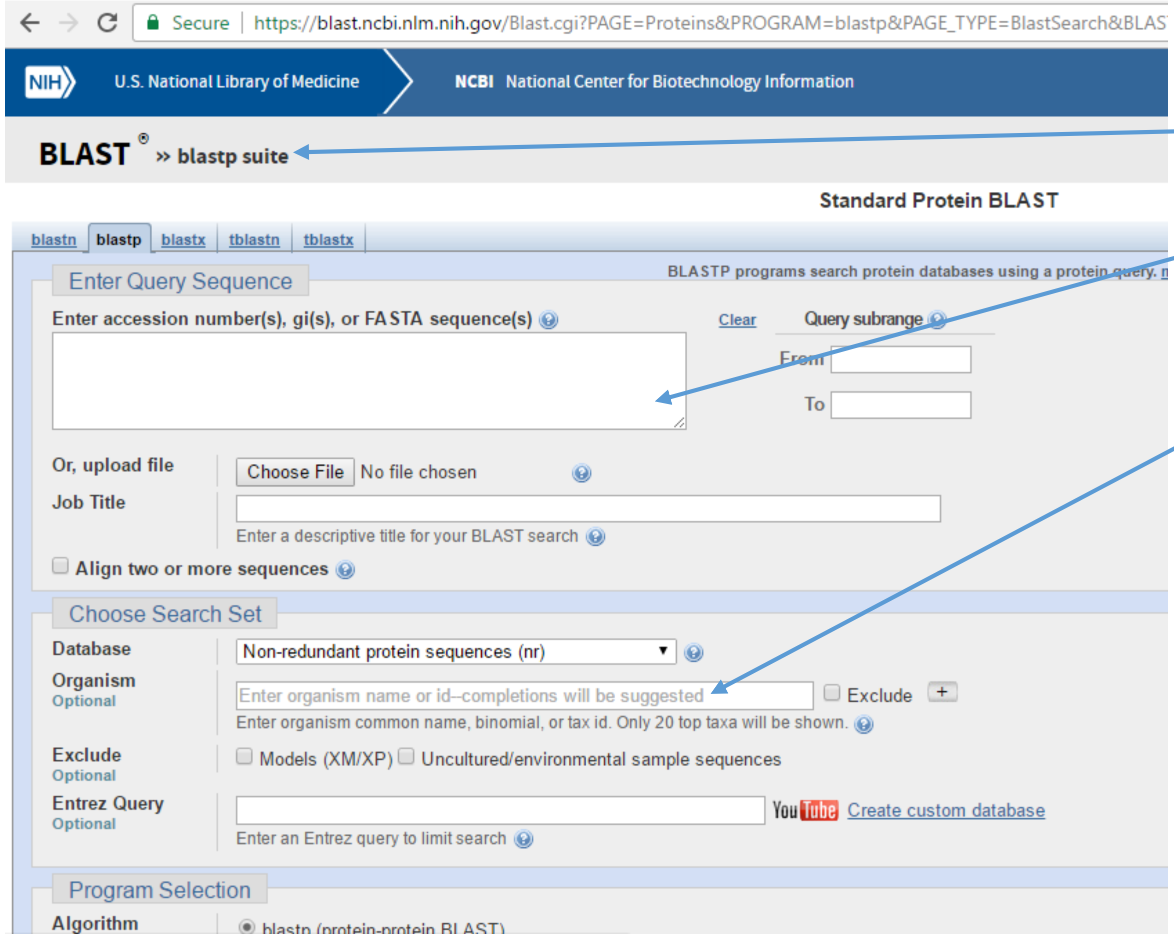

Use blastp for protein BLAST

Enter the FASTA sequence into the appropriate box, choose any selection / parameters. And press BLAST at the bottom.

Indicate "Insecta". This will limited your search to insects only.

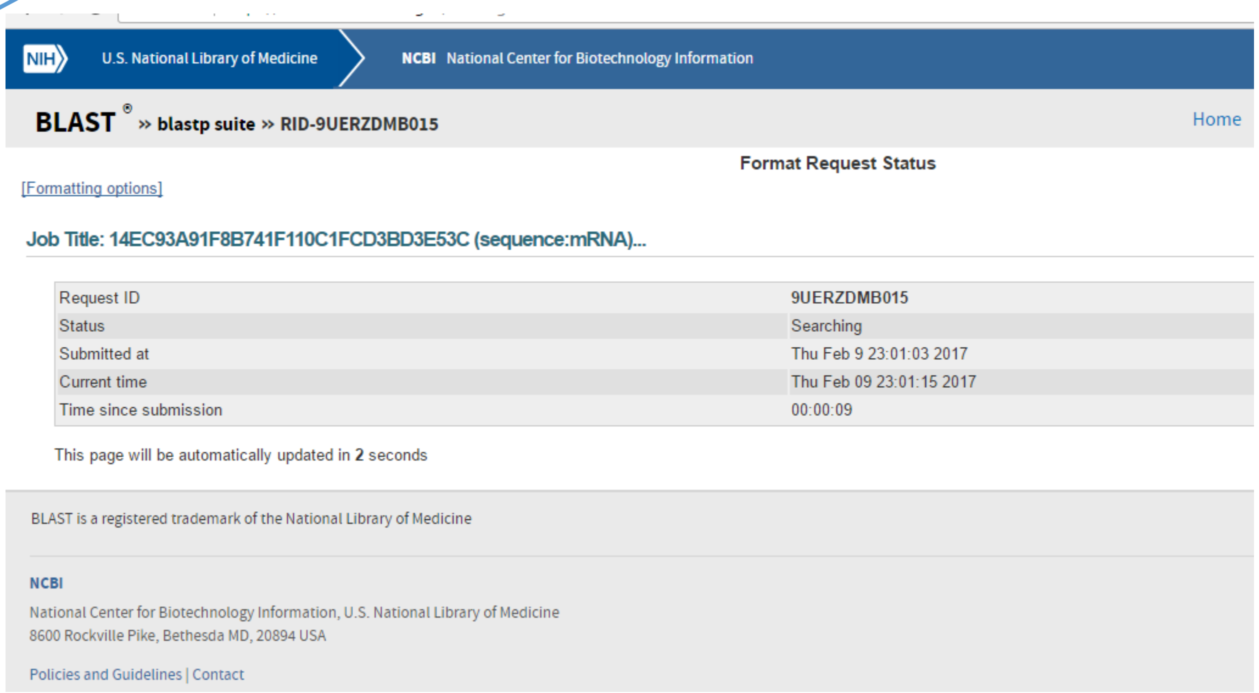

The NCBI Results Page

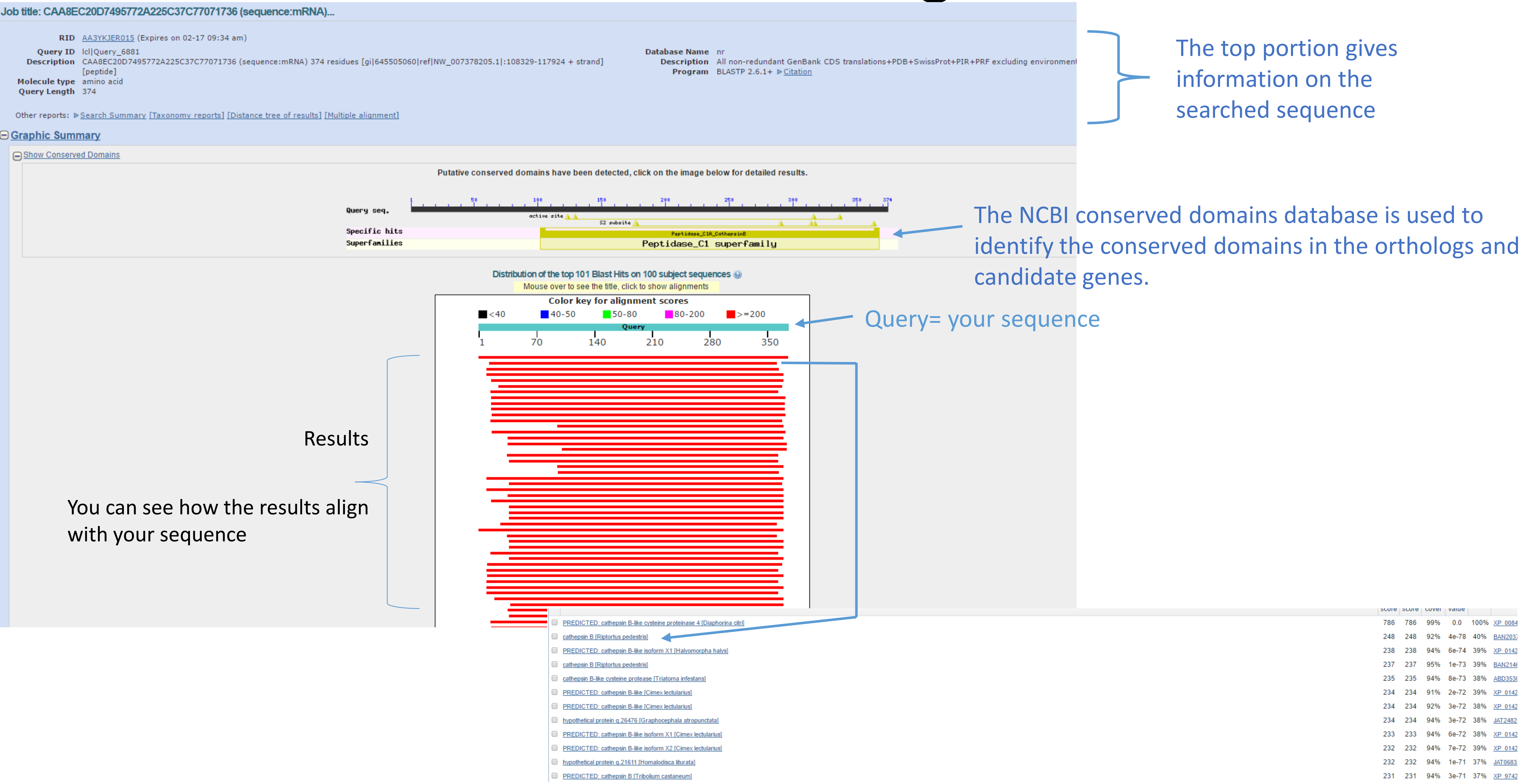

The top portion gives information on the searched sequence

The NCBI conserved domains database is used to identify the conserved domains in the orthologs and candidate genes.

Query= your sequence

Results

You can see how the results align with your sequence

The NCBI Results Page cont'd

Pairwise Alignment

cathepsin B-348 precursor [Acyrthosiphon pisum]

Sequence ID: ref|NP_001119608.1| **Length:** 342 **Number of Matches:** 1

▶ See 1 more title(s)

Range 1: 9 to 341 GenPept Graphics ▼ Next Match ▲ Previous Match

Score	Expect	Method	Identities	Positives	Gaps
229 bits(583)	5e-68	Compositional matrix adjust.	132/346(38%)	180/346(52%)	17/346(4%)

```
Query  1     MIHILVFLLG---CTLVRGELYKFSDAYIDQINREANTWTAGRNFPANLSEEYLRQFLIA  57
             ++ +L+F  G      VR +L   SD +ID IN     W+AGRNF  +    Y++  +
Sbjct  9     LVGLLIFSFGRVDGATVRVDLNPLSDEFIDHINSIQYYWSAGRNFHKDTPISYIKGLMGV  68

Query  58    DAKYFDQSDRPLPGDRKTYDPEYSATVPDRFDAREQWPNCGTIGHVPDTGACAAPHIFAA  117
               K    ++ P      TY+ + S  +P+ FDARE+WPNC TI  V D G+C +   F A
Sbjct  69    HEK---NAEYPKLEQLLTYN-DASTDLPETFDARERWPNCPTIREVRDQGSCGSCWAFGA  124

Query  118   VGAFSDRRCIKSKGQQNRPLSTEYVASCCKICRYDDNKSCSHGSVFRTWNFLHKRGSVTG  177
             V A SDR CI S G +N   S E + SCC  C +     C+ G     WN+   +G V+G
Sbjct  125   VEAMSDRVCIHSNGTKNFHFSAENLVSCCWTCGF----GCNGGFPGAAWNYWKTKGIVSG  180

Query  178   GDYGDRTGCQPSTISPCSHHGSAPTLPSCENQKVPKLKCHTRCTNPTYGRGFFQDKHRTT  237
             G YG   GC P  I+PC HH +    P  E  K P   C  +C    Y   + QD H
Sbjct  181   GPYGSNMGCIPYEIAPCEHHVNGTRGPCKEGGKTP--TCVKKCEE-GYKVPYAQDLHHGK  237

Query  238   LTYWVDDNEDAIKKEILAHGPTTATFALYDDFYHYKSGVYKHTSNAKLENYLHSGKLIGW  297
               Y + ++ D I++EI  +GP    F +Y+DF  Y++GVYKH +   L    H+ +++GW
Sbjct  238   SAYSIRNDVDQIRQEIYTNGPVEGAFTVYEDFIAYRAGVYKHVAGKALGG--HAIRILGW  295

Query  298   GTENG-TPYWLVINTWGPHWGDRGTVKILRGKYECAFEYLIAAGKP  342
             G +NG  PYWLV N+W   WG  G  KILRG  EC  E  I AG P
Sbjct  296   GVQNGEIPYWLVANSWNTDWGSDGFFKILRGSDECGIEGQINAGLP  341
```

You can see how similar your gene model is to other similar genes in related organism(s), by clicking on each result (previous slide).

Example: The gene model is 38% identical and 52% similar to gene in *A. pisum*.

Or check gene model by using

NCBI Smart BLAST

https://blast.ncbi.nlm.nih.gov/smartblast/?LINK_LOC=BlastHomeLinkn

Smart BLAST will provide a phylogenetic tree with model organisms.

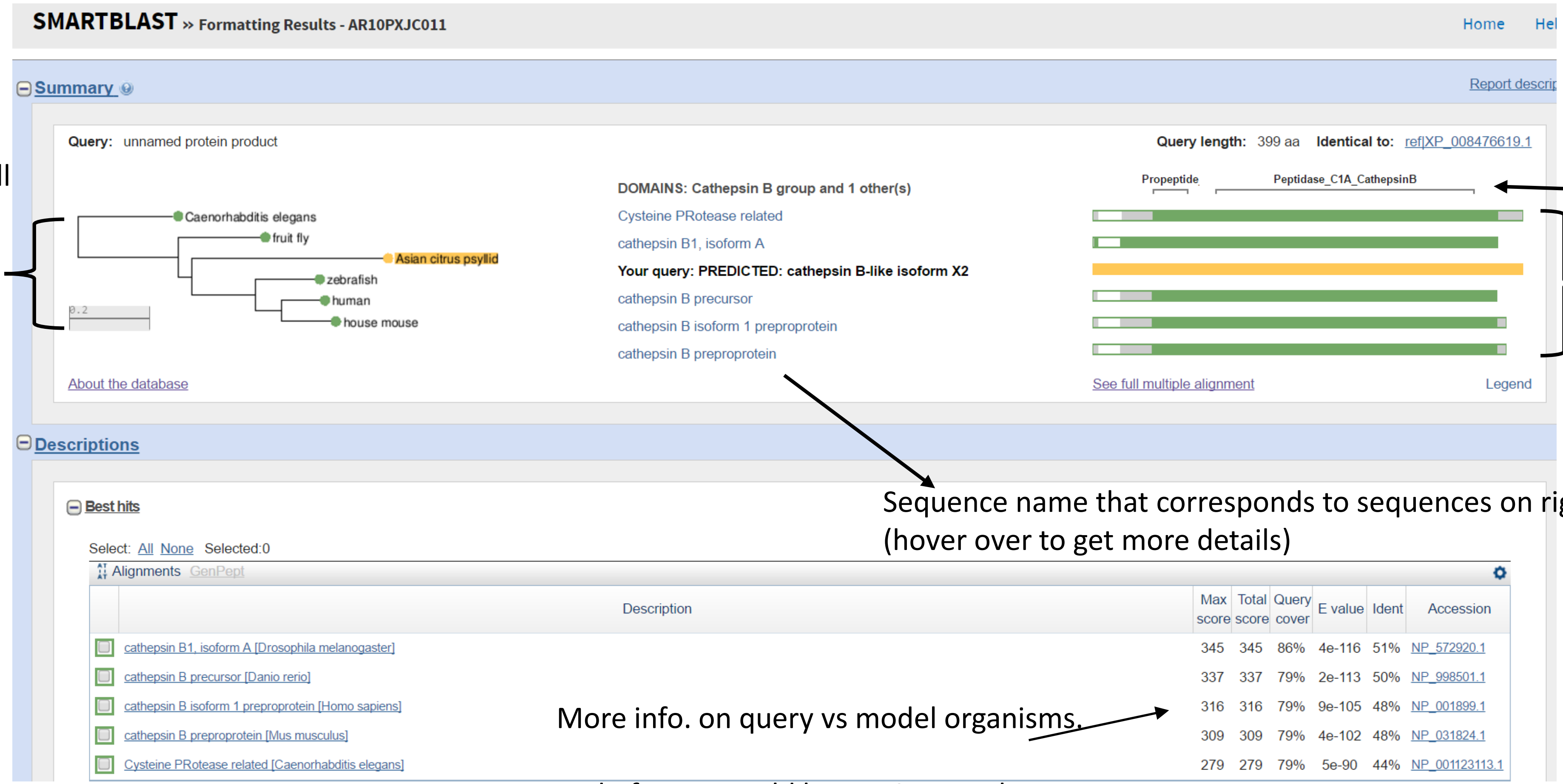

Conserved domains within the sequences

Green: subject sequences

Yellow: your sequence (query)

Sequence name that corresponds to sequences on right. (hover over to get more details)

More info. on query vs model organisms.

End of page would be BLASTp results.

RNASeq Data

To view gene model in different colors indicating the diff. frames: (As seen) Click View, Color by CDS frame.

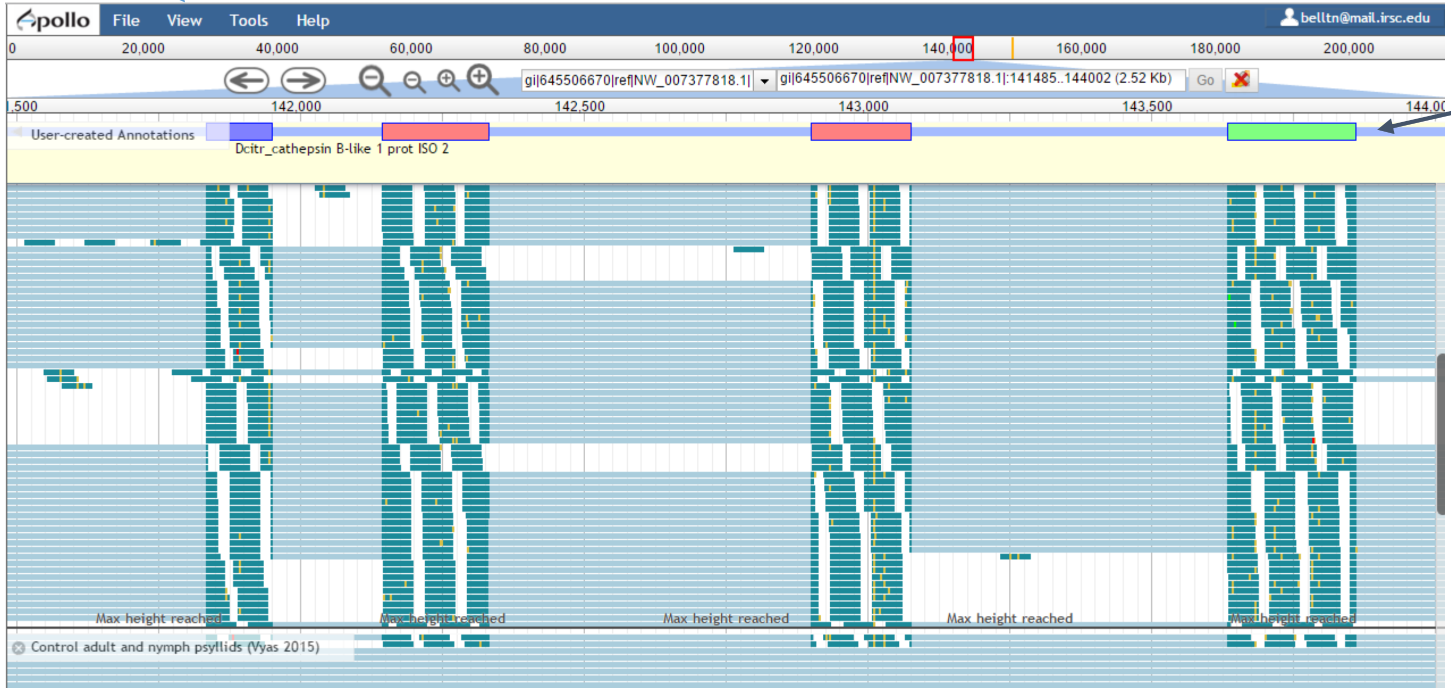

The colored outlined boxes represent exons, while the thin blue lines connecting them represent introns. The blue boxes without an outline represent UTRs

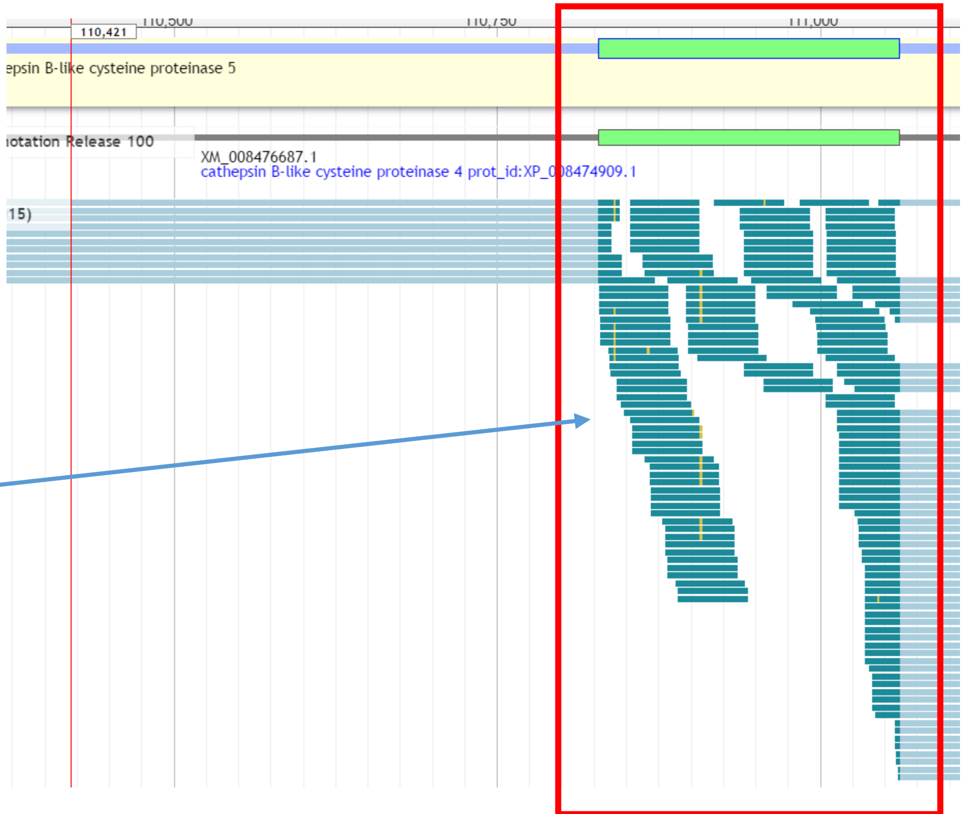

Look closely at the results and see how they line up with RNASeq data. You may have to zoom in close and look by sections.

Dark blue RNA seq reads indicate evidence for exons and light blue for introns.

RNASeq Data

More details

You can also view the RNAseq Coverage Plots.
(On the left side on Apollo (Available Tracks))

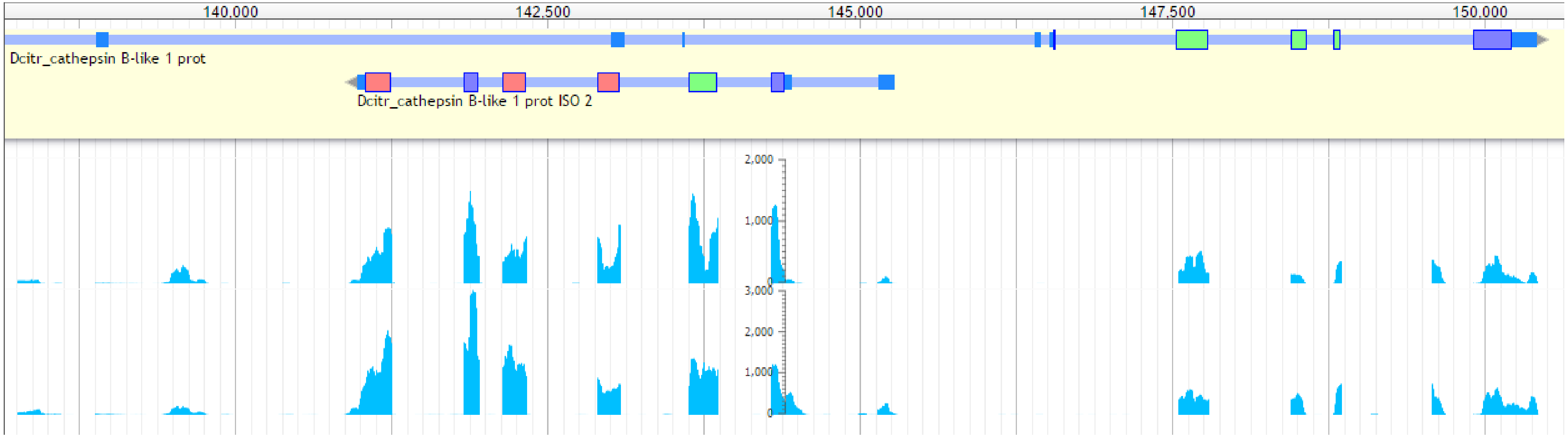

If you zoom in and see letters, this is the reference sequence, as shown here. You can view stops on the different frames by an *.

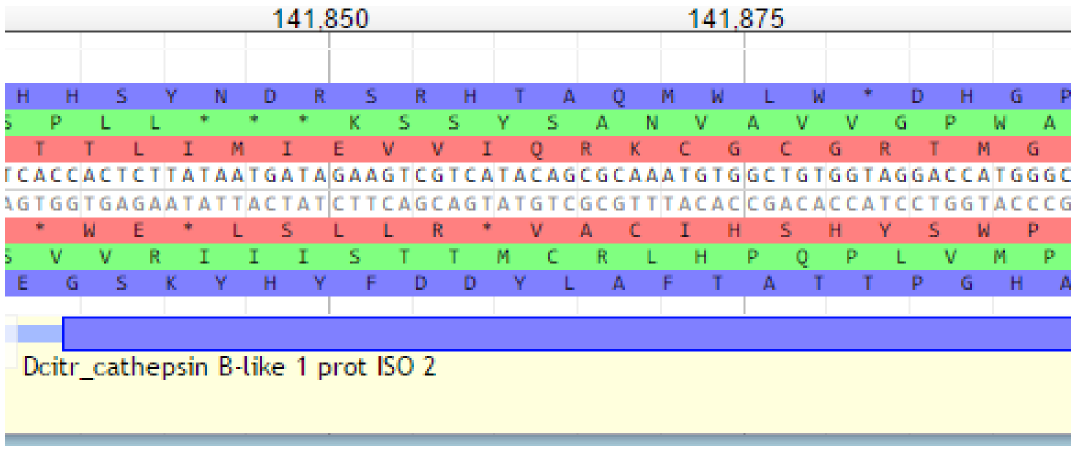

Making Edits

UTR's are dark blue and are on the 5' and 3' ends of the gene model.

Example: This is the 3' UTR of the gene model

Matching Data

Delete exons, or UTR (Untranslated region)

Merge exons, or gene models

Split

Duplicate

Make Intron

Set to Downstream Splice Donor

Set to Upstream Splice Donor

Set to Downstream Splice Acceptor

Set to Upstream Splice Acceptor

View History

Indicates which direction (Upstream or Downstream) the sequence is reading. In this case = downstream (-) strand

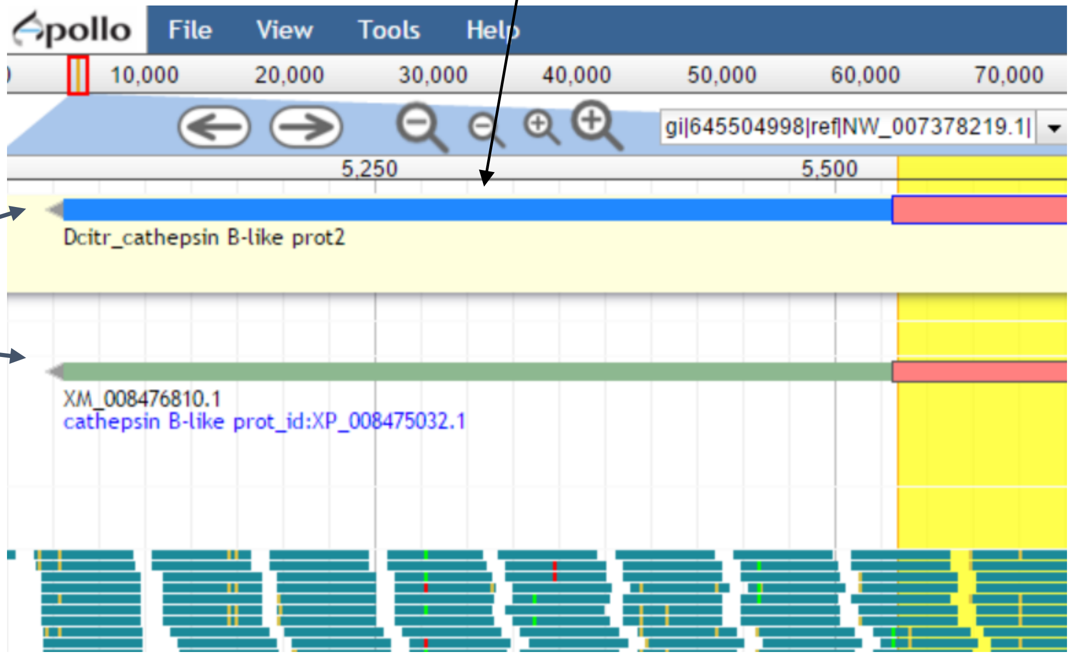

Important:

Splice sites:
Reverse (-) strand gene model:
3' acceptor of intron, GA
5' donor of intron, TG, CG
Positive (+) strand gene model:
3' acceptor of intron, AG
5' donor of intron, GT, GC

Make sure your gene model starts with ATG (M) and ends with TAG, TGA, or TAA (stop codons).

Fixing splice sites

Splice sites:
Reverse (-) strand gene model:
3' acceptor of intron, GA
5' donor of intron, TG, CG
Positive (+) strand gene model:
3' acceptor of intron, AG
5' donor of intron, GT, GC

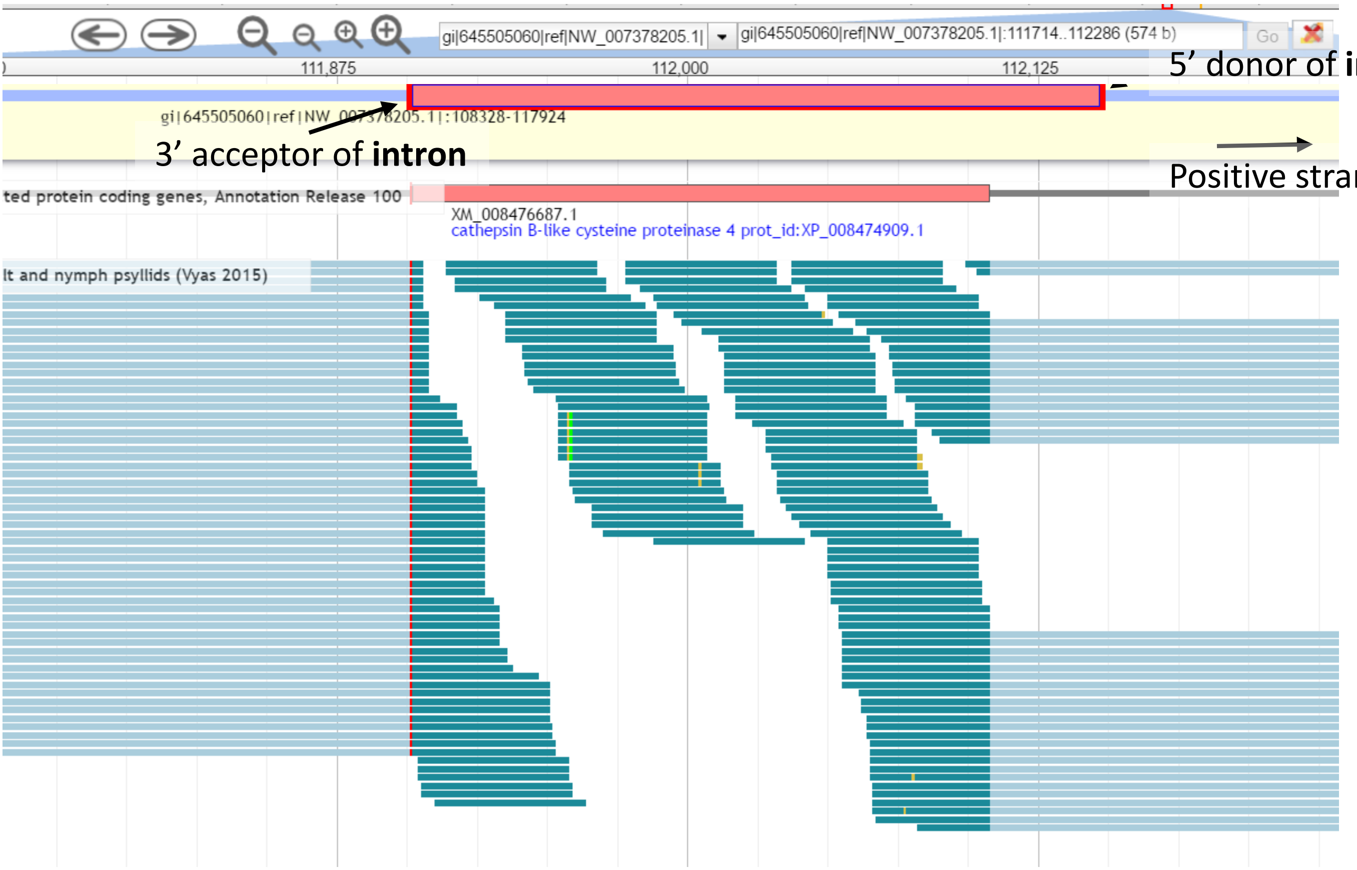

For this example, the donor would be moved upstream. (RNAseq evidence to do so)
Right click exon, Set to upstream splice donor.

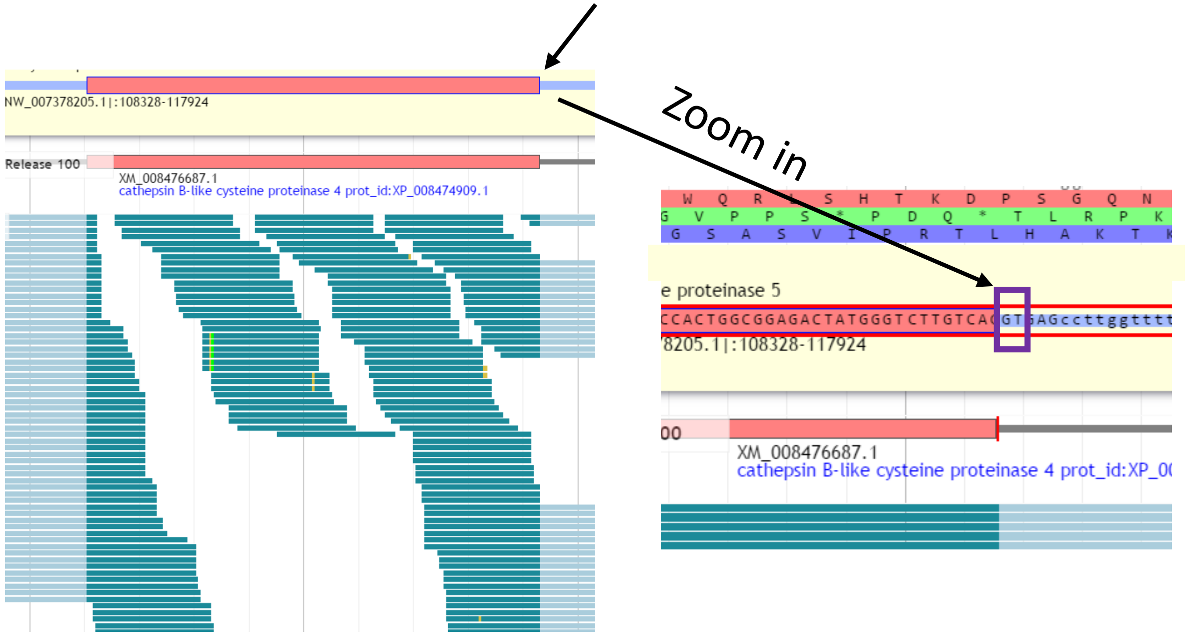

Adding an exon

Other gene predictions can be used, not limited to, Maker, augustus, snap.

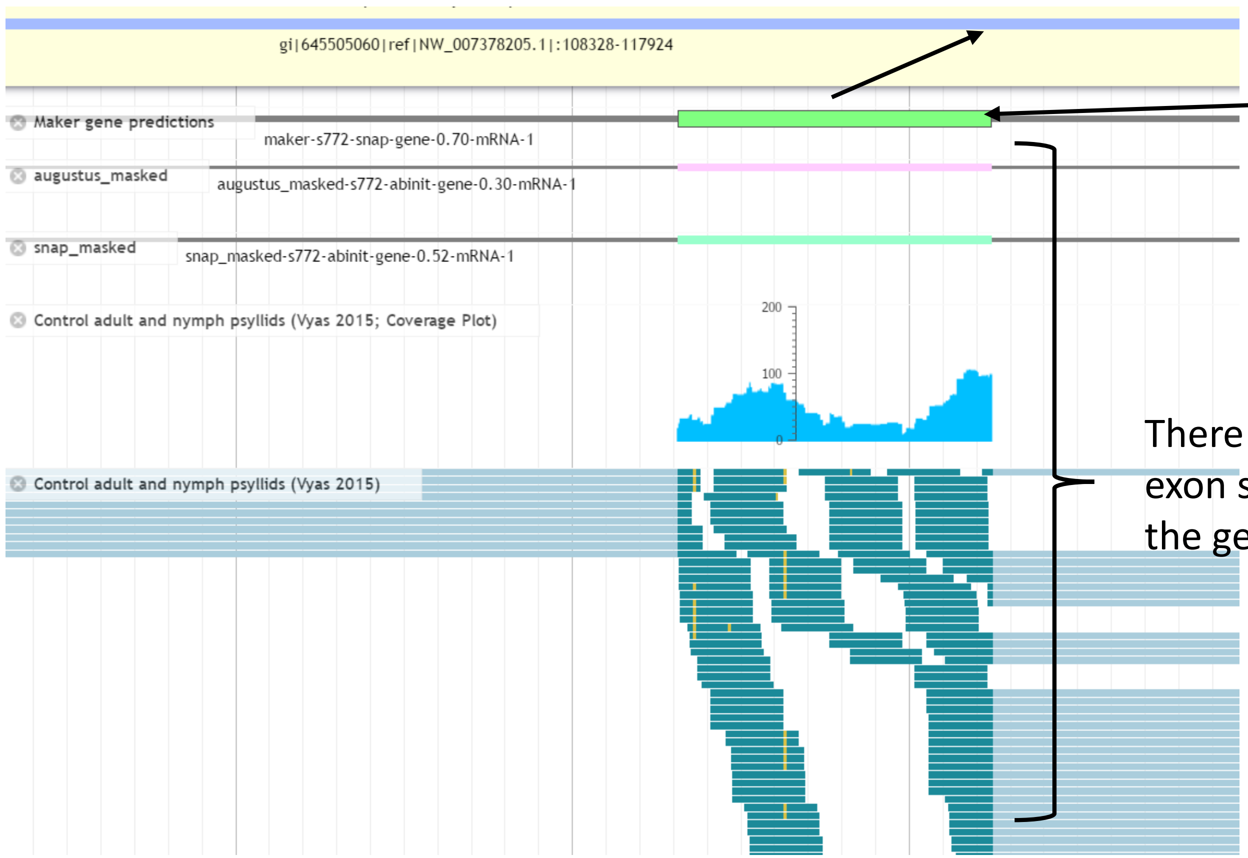

Click and drag exon on top of gene model and drop.

There is evidence that an exon should be added to the gene model

After

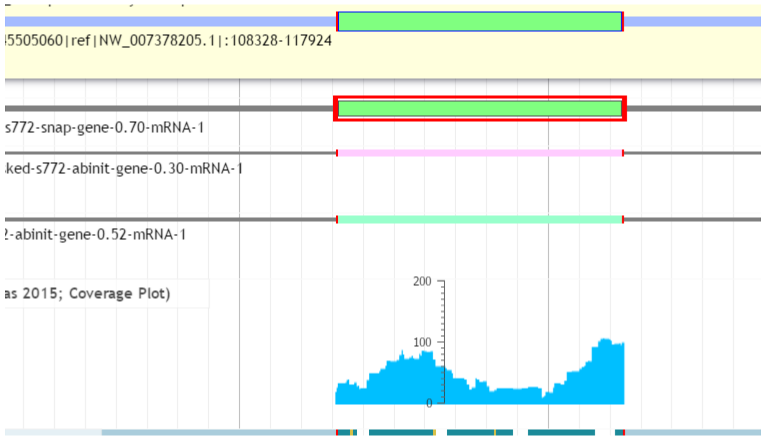

Isoforms

When the gene can undergo alternative splicing

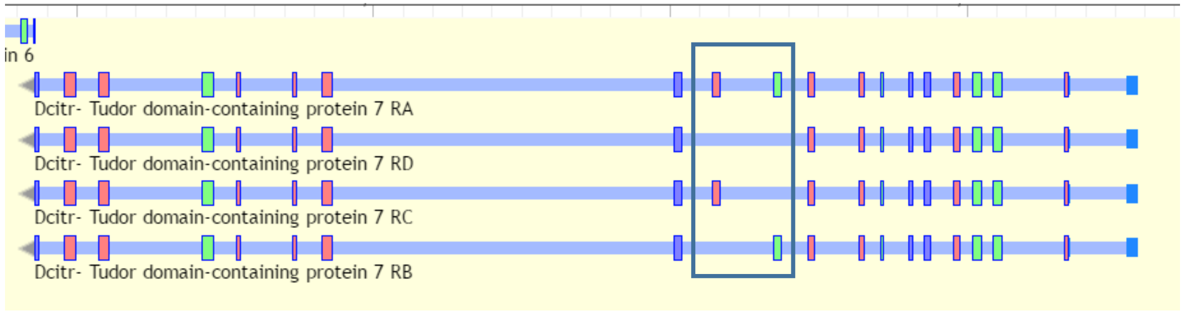

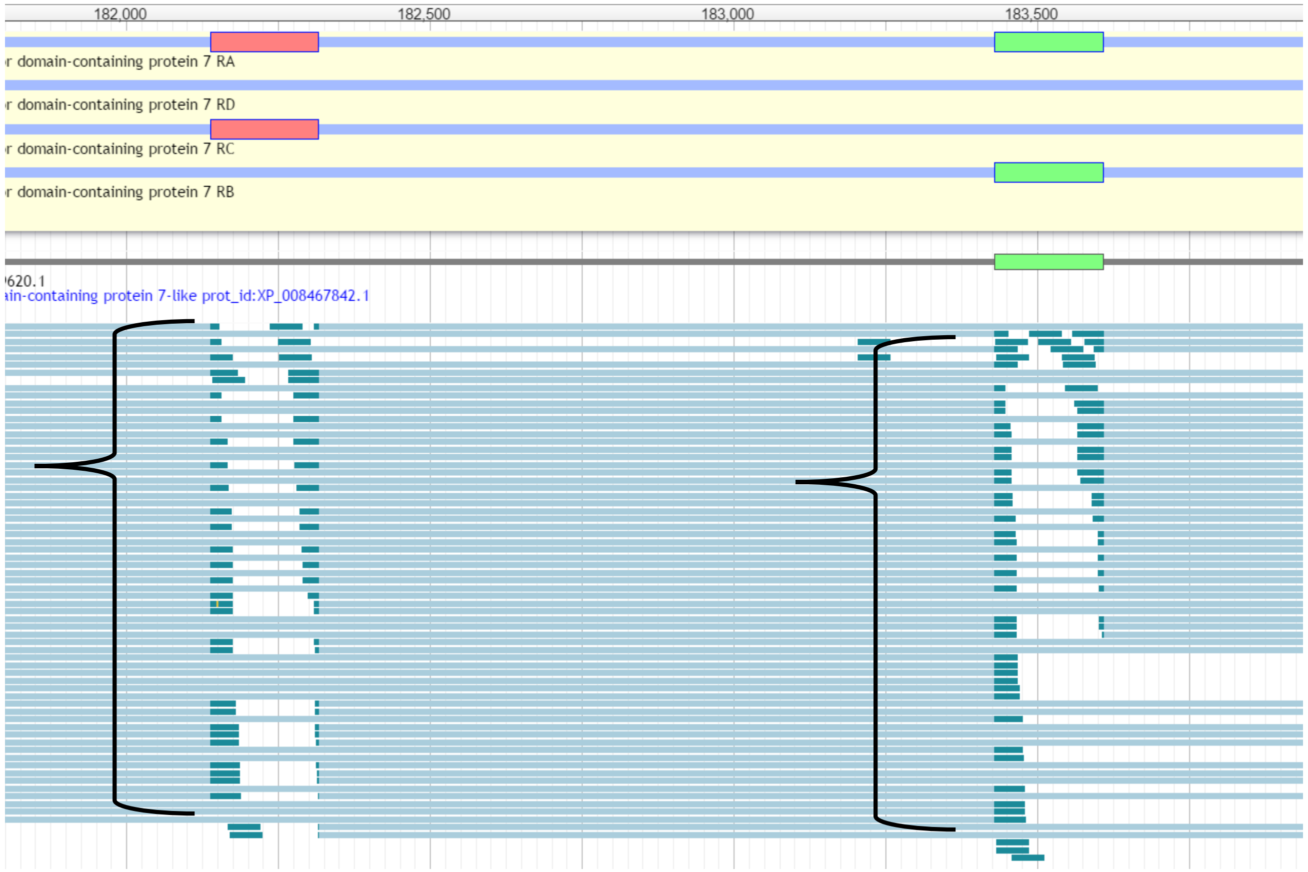

RNAseq reads indicate intron and exon evidence for the same part of the model.

If there were many results containing isoforms when the gene model was initially blasted on NCBI, this is good evidence for isoforms as well.

If you have evidence for isoforms, right click your gene model in the “user-created annotations” space, and duplicate.

Edited Gene Models

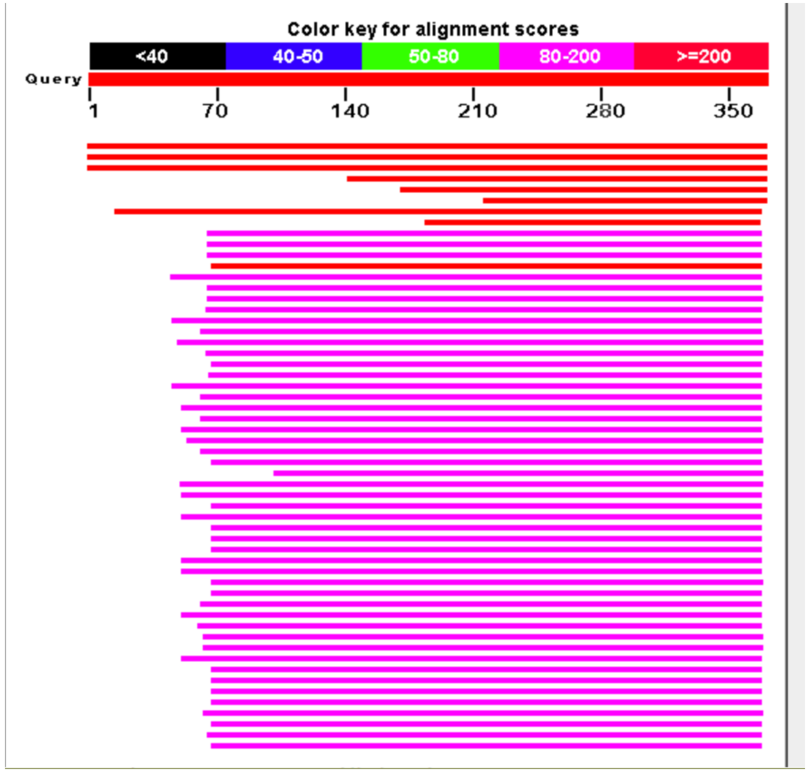

Predicted Model Conserved domain results

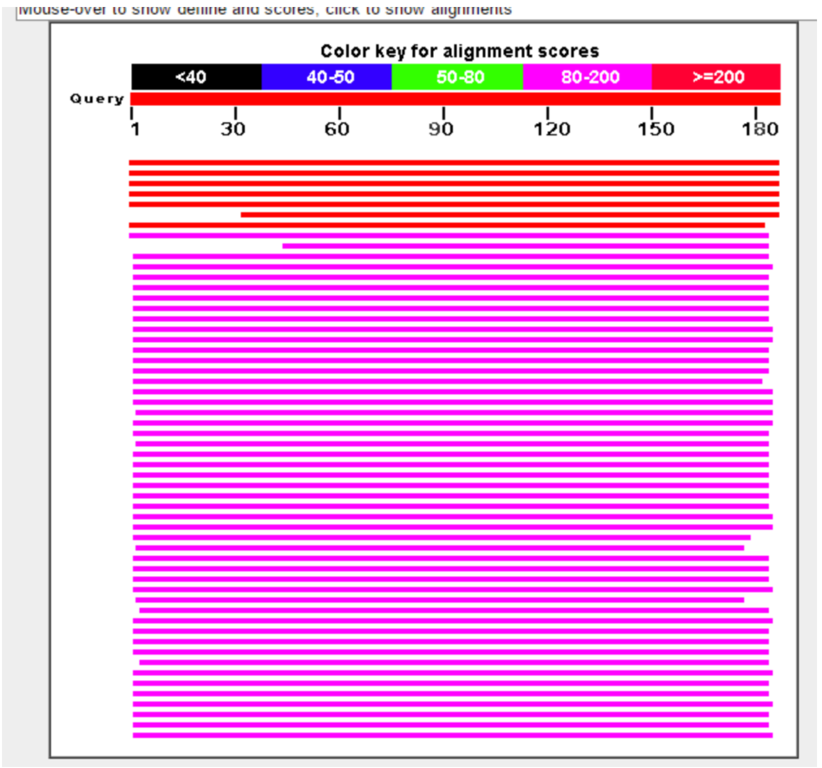

Conserved domain results after matching your gene model with RNASeq data

After a change is made, check your results using the BLASTp or Smart BLAST again

Information editor

Right click on the model in the User Created section and choose "Edit Information"

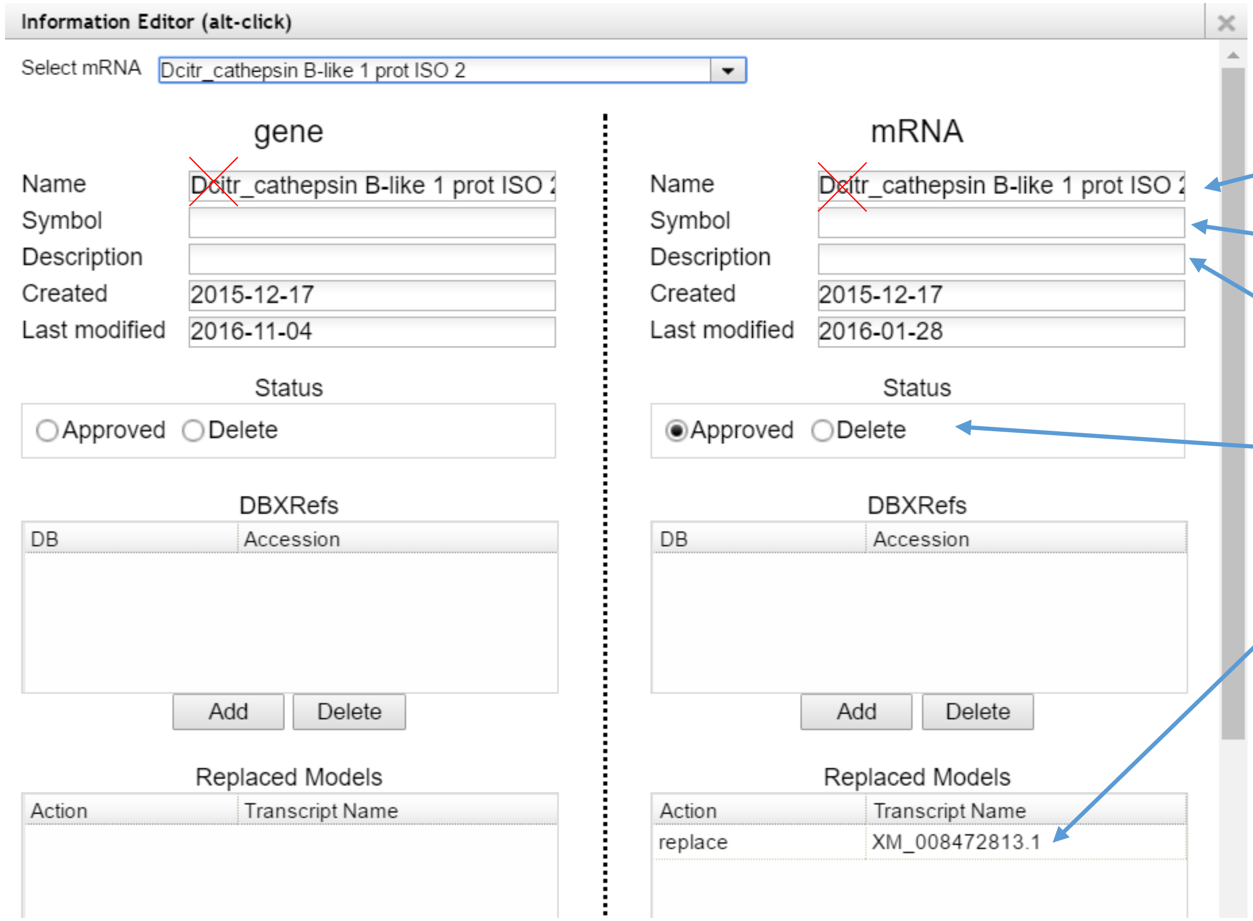

Name of the gene. Do NOT use Dcitr

Symbol or abbreviation of the gene

Description of the gene, usually function. Uniprot is a good source.

Click approved once all edits are made and your gene model is complete

You will need the name of the original model that your annotation will be replacing

Example:

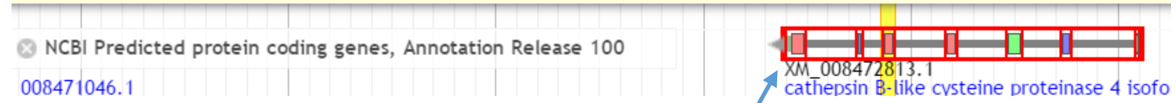

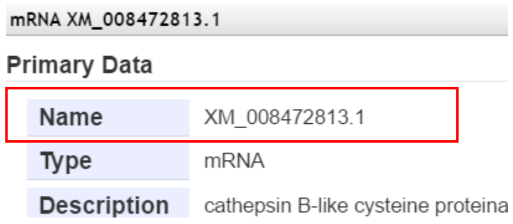

Right click on model > View details

Fill in both sections: Gene and mRNA panels

Information editor cont'd

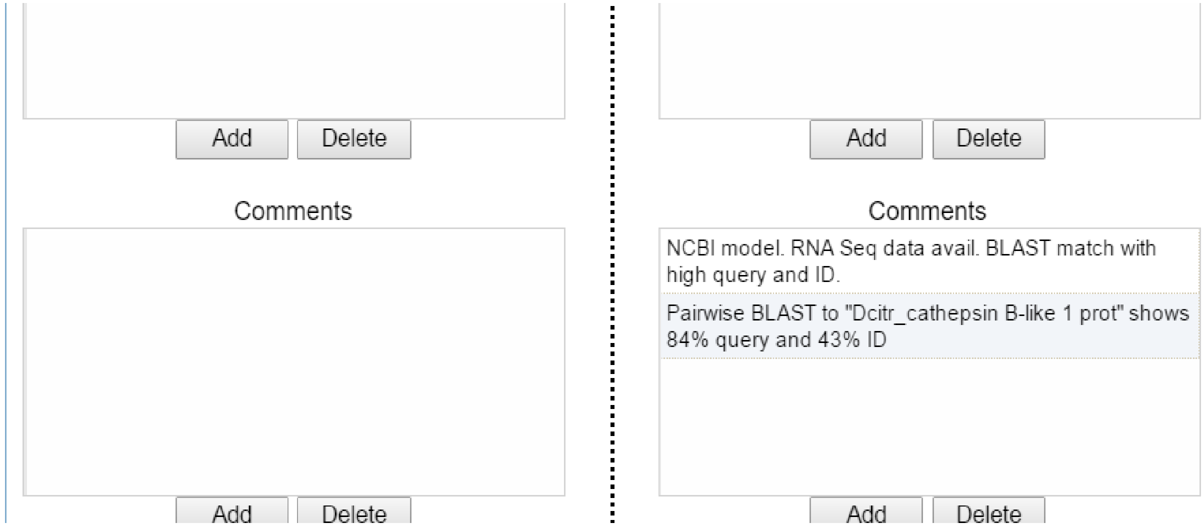

Here you will enter comments about all the edits (splice sites moved, added exons, deleted exons, etc.) made to your gene model and other relevant info.

Naming isoforms:

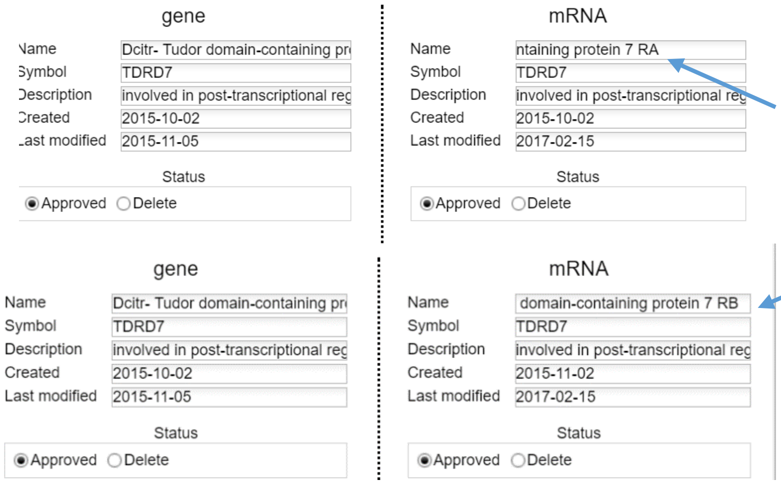

Naming convention for isoforms should have RA, RB, RC, etc. at the end for each isoform you have, respectively. (look at slide 20)

The gene section should be the same for all isoforms.

But the mRNA section should be different for all isoforms, comments should contain the edits made for the particular isoform clicked on.

Gene Family Report

Once your gene or gene family is annotated, the gene report should include:

- Introduction to the gene or gene family including the pathway (use literature)
- Methods of annotation and phylogenetic analysis
- Results and Discussion (use literature)
- References cited

Tables to include in report

Table with gene count in:

- *Drosophila melanogaster*
- *Anopheles gambiae*
- *Tribolium castaneum*
- *Apis mellifera*
- *Nasonia vitripennis*
- *Acyrthosiphon pisum*
- *Bemicia tabaci*

Table with blast match:

- Indicate organism and name of gene
- Query coverage
- % identity
- Bit score

Include legend for each table.

Comparative and Phylogenetic Analysis

When your gene model(s) are completed, perform analysis in MEGA.

1. Construct tree with *D. citri* gene models only.
2. Construct tree with *D. citri* gene models and orthologs. Use related sequences from, but not limited to,
    - *Drosophila melanogaster*
    - *Anopheles gambiae*
    - *Tribolium castaneum*
    - *Apis mellifera*
    - *Nasonia vitripennis*
    - *Acyrthosiphon pisum*
    - *Bemicia tabaci*

Can find orthologous sequences in NCBI, Ensembl, Uniprot, etc.

Multiple Sequence Alignments (MSA)

Multiple sequence alignments should be generated using MUSCLE, tcoffee, or clustal to compare the ACP gene model to the query gene set. The final model should be refined in Apollo using homology, RNAseq and proteomics evidence tracks.

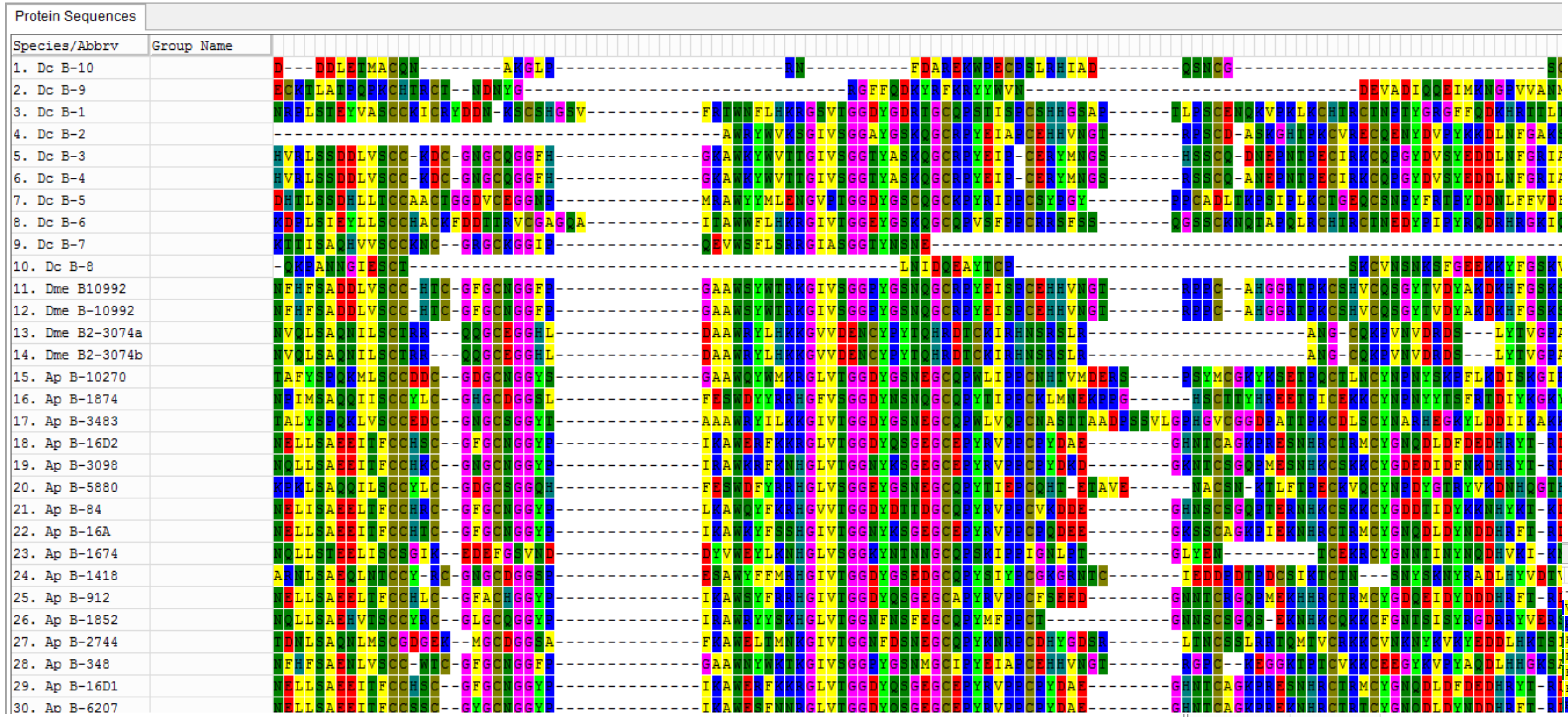

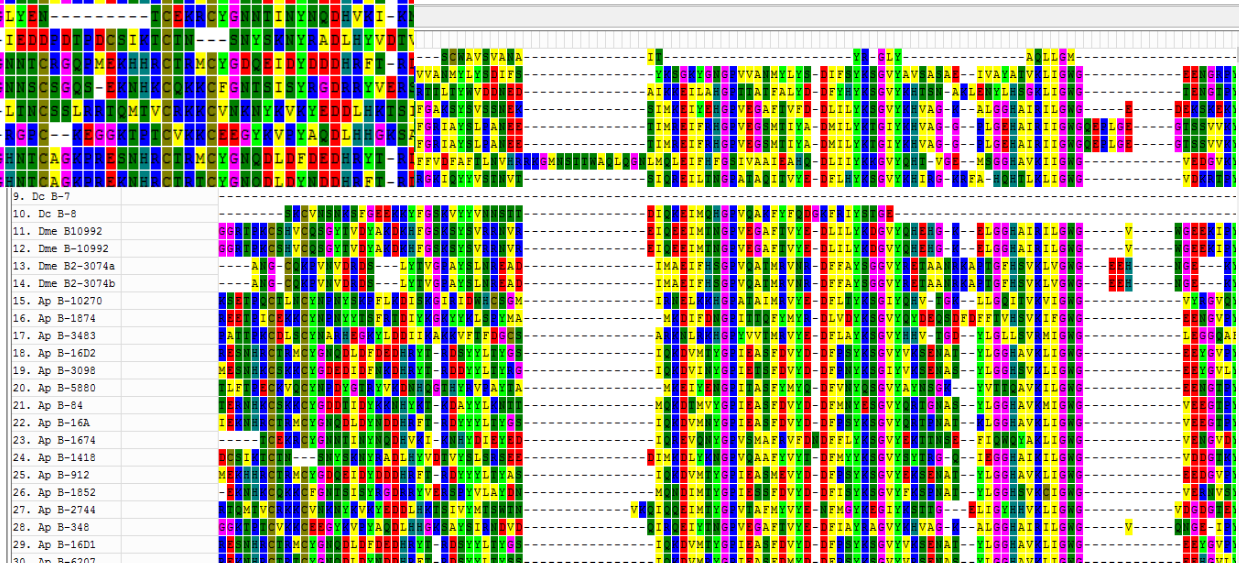

Tree of all *D. citri* gene models

This would include all genes (paralogs) for the particular family annotated in the genome.

Example:

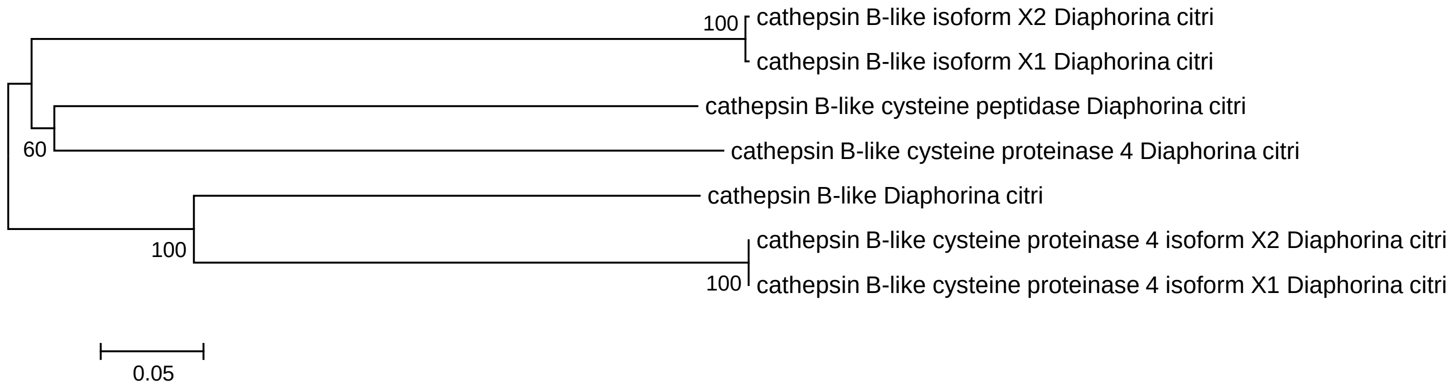

Tree should be included in report with legend.

Tree of all *D. citri* gene models and other orthologs

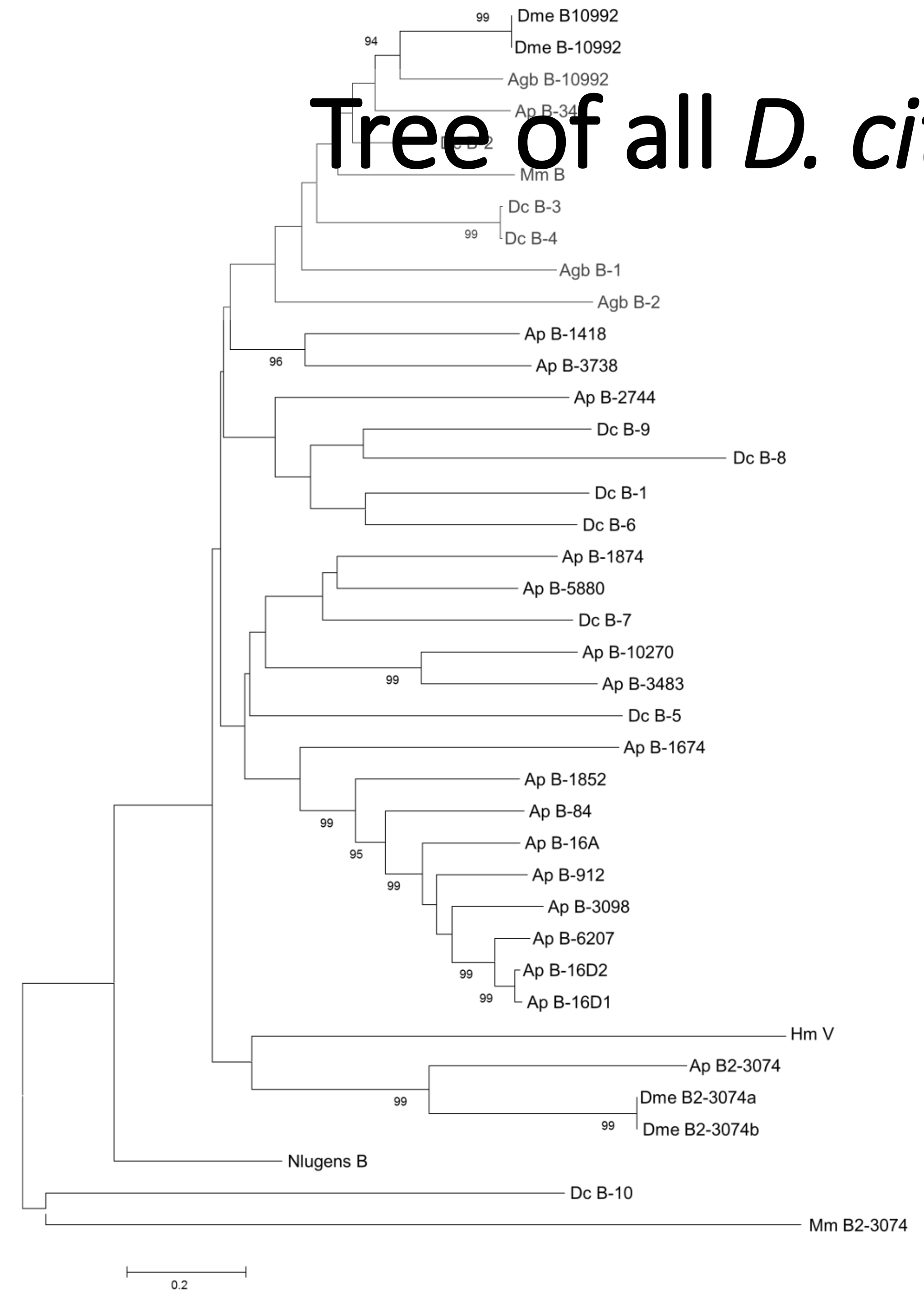

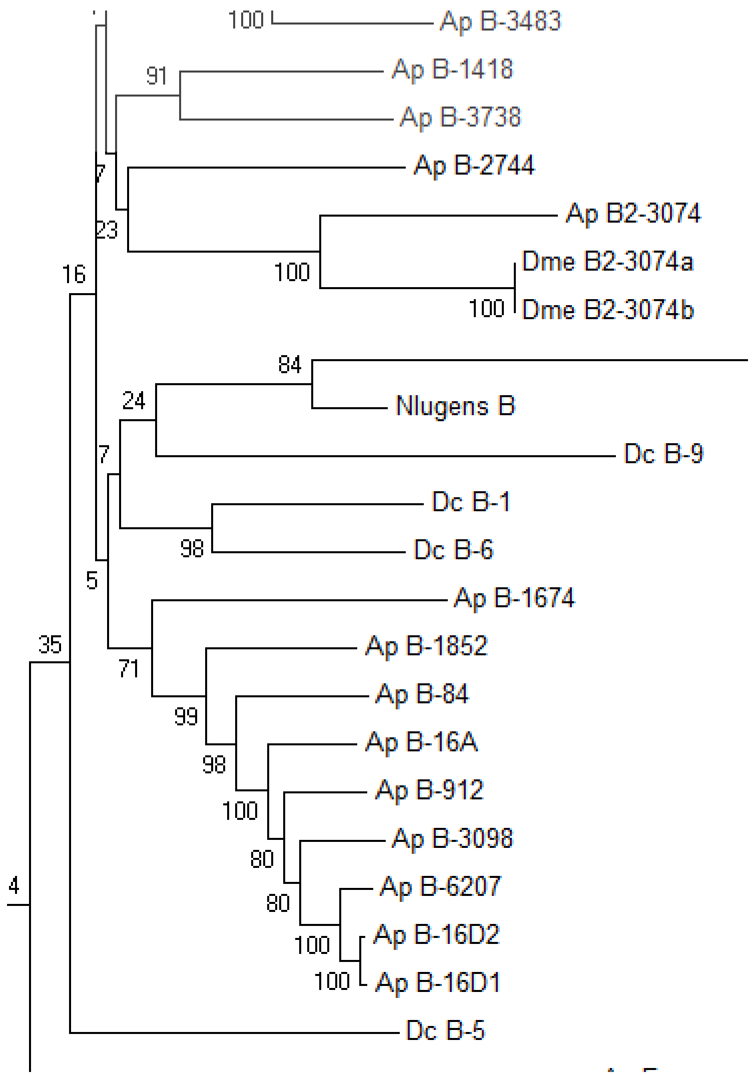

Tree should be included in report with legend

Krystal Villalobos Ayala
Chris Cordola
Tracey Bell
Hannah Mann
Daniel DeAvila
Gabe DeAvila
Tom D’elia

Indian River State College, FL
